# Synthesis of C*sp* ^3^ Chlorinated Compounds from Cyclopropanes, Olefins, and C–H Bonds via Photolysis of Willgerodt-Type Reagents

**DOI:** 10.1021/jacsau.5c01226

**Published:** 2026-01-12

**Authors:** Tin V. T. Nguyen, Thanh V. Q. Nguyen, Trinh T. H. Tran, Qui-Hien Nguyen, Jerome Waser

**Affiliations:** † School of Pharmacy, 249295 University of Medicine and Pharmacy at Ho Chi Minh City, 41 Dinh Tien Hoang, Sai Gon Ward, Ho Chi Minh City 710000, Vietnam; ‡ Laboratory of Catalysis and Organic Synthesis, Institute of Chemical Sciences and Engineering, 27218 École Polytechnique Fédérale de Lausanne, Lausanne CH-1015, Switzerland; § Medicinal Chemistry, Research and Early Development, Respiratory and Immunology, BioPharmaceuticals R&D, AstraZeneca, Gothenburg, Mölndal 431 83, Sweden

**Keywords:** chlorination, cyclopropanes, hypervalent iodine, direct excitation, photocatalysis

## Abstract

C*sp* ^3^–Cl bonds are essential as diversification handles in organic synthesis and are found in many natural products and bioactive molecules. In this work, we introduce a general protocol for the selective chlorination of aryl cyclopropanes, olefins, and activated C–H bonds using direct photoexcitation of Willgerodt-type reagents to generate chlorine radicals. Preliminary results for an iodine­(I/III) catalytic process starting from abundant chloride salts are also presented. Furthermore, a one-pot protocol has been developed for the telescoped functionalization of benzylic chlorides with C-, N-, O-, and S-nucleophiles. Especially, this approach provides a platform to access 1,1-diaryl motifs, which are important building blocks for the synthesis of pharmacophores.

## Graphical abstract

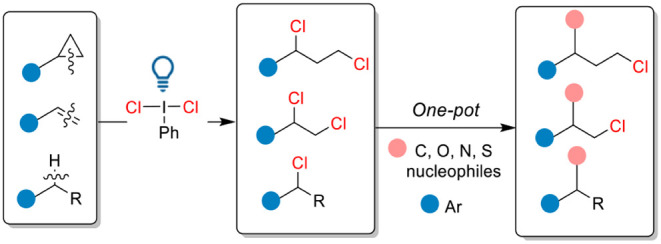

Chlorinated compounds are found in various natural products such as Atpenin A5 (**1**), Perforenone B (**2**), or Clionastatin B (**3**); active pharmaceutical ingredients such as Quinfamide (**4**) or Chloramphenicol (**5**); and are essential intermediates used in chemical manufacturing (Scheme 1A).^1^ Chlorine-based chemistry has been used in the synthesis of around 20% of small-molecule drugs and 30% of agrochemical products.^1a^ Among organochlorides, chloroalkanes serve as important synthetic precursors to access several functional groups, including alcohols, amines, and thioethers.^1^ The classical methods for synthesizing chloroalkanes involve electrophilic addition of formal [Cl^+^] intermediates to an olefin (Scheme 1B, eq 1),^2^ nucleophilic substitution with chlorides [Cl^−^] (Scheme 1B, eq 2),^3^ and the use of chlorine radicals [Cl^•^] (Scheme 1B, eq 3). As they are difficult to control, chlorine radicals have long been regarded as less attractive for fine chemical synthesis. However, recent progress in radical chemistry has rekindled interest in this approach.^4^ Different transformations have been developed, including radical additions to olefins^4a,4b^ hydrogen atom transfer processes onto C*sp* ^3^–H bonds,^5,6^ or oxidative cleavage of C–C σ bonds.^7^

**Scheme 1:**
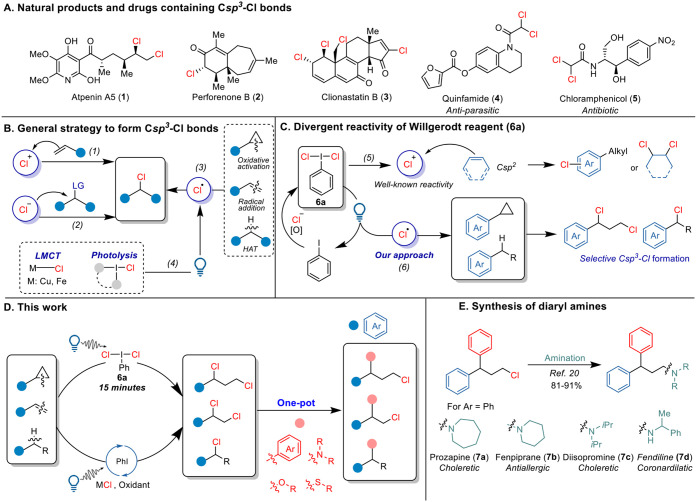
(A) Natural Products and Drugs Containing C*sp* ^3^–Cl Bonds. (B) General Strategy to Form C*sp* ^3^–Cl Bonds. (C) Divergent Reactivity of Willgerodt Reagent (**6a**) to Give Cl^+^ or Cl^•^. (D) Our Work on Photoactivation of Willgerodt Reagent (**6a**). (E) Synthesis of Amine Drugs from Diaryl Chlorides

With an emphasis on sustainable chemistry in the past few decades, several photocatalytic protocols have been developed to convert electrophilic or nucleophilic chlorine sources to chlorine radicals.^4,5,6,7,8,9^ These methods include reductive activation,^5d^ oxidative activation,^5b,5c,5e,5i,8^ and, more recently, ligand-to-metal charge transfer (LMCT) processes for the homolytic cleavage of metal chlorides.^4b,9^

Hypervalent iodine reagents (HIRs) have similar properties to heavy metals in organic transformations.^10^ In particular, the homolytic fission of the I–Cl bond can generate chlorine radicals analogous to LMCT processes (Scheme 1B, eq 4). Indeed, the photolysis of HIRs to give chlorine radicals has been described using iodine trichloride ICl_3_,^11^ Willgerodt reagent (**6a**)^12^ and recently chlorinated cyclic HIRs.^13^ Among them, **6a** stands out as it is more stable and easier to handle than ICl_3_ and also more atom-economic than cyclic HIRs. **6a** has been extensively applied for the electrophilic chlorination of C*sp* ^2^ centers, such as olefins or aromatic rings (Scheme 1C, eq 5),^14^ as well as for the oxidation of alcohols^15^ and thioethers.^16^ These reactions are potentially competitive with radical chlorination, suggesting that **6a** would not be a good choice to favor this pathway. We hypothesized, however, that the irradiation of **6a** would lead to rapid homolytic fission of the I–Cl bond, producing chlorine radicals fast enough to suppress electrophilic chlorination pathways. As a result, highly selective radical chlorination of alkane C–H and cyclopropane C–C bonds to form C*sp* ^3^–Cl bonds could be achievable even in the presence of arenes or alcohols (Scheme 1C, eq 6). Although the use of **6a** for chlorination under photochemical conditions was initially introduced by Banks,^12a^ it has been applied only to the chlorination of steroids by Breslow, Wicha, and coworkers in the 70s and 80s,^17^ and the potential of this approach in other chlorination reactions has not been further explored. Given that reagent **6a** can be easily accessed via the reaction of chloride anions with iodosylbenzene,^18^ it can potentially be generated in situ using a catalytic amount of iodobenzene in the presence of an external oxidant. Gilmour and Wirth recently reported catalytic iodine­(I/III) chlorinations from CsCl or TMSCl, focusing on the electrophilic chlorination of olefins.^14b,14f^ A radical approach would allow us to target not only olefins but also cyclopropanes C–C and alkanes C–H bonds.

Herein, we report the use of **6a** for the formation of various C*sp* ^3^–Cl bonds, including 1,3-, 1,2-, and C–H-chlorinated products starting from cyclopropanes, alkenes, and alkanes, respectively (Scheme 1D). The most efficient protocol was established using a stoichiometric amount of **6a**, but preliminary results for a catalytic method were also obtained. Additionally, we introduce a one-pot process to further functionalize benzylic chlorides with aryl, N-, O-, and S-nucleophiles, providing a fast and modular approach to 1,1-diaryl compounds, important building blocks in the synthesis of various pharmacophores.^19,20^ Especially, 1,1-diphenyl-3-chloropropanes were reported as the precursors to synthesize a wide range of commercial drugs such as Prozapine (**7a**), Fenpiprane (**7b**), Diisopromine (**7c**), or Fendiline (**7d**) (Scheme 1E).^20^

We started our investigation by irradiating a mixture of phenylcyclopropane (**8a**) and chlorobenziodoxolone reagent **6b**, which we had previously used as a chlorine radical source in the oxidative activation of cyclopropanes (Scheme 2A).^13c^ After 4 h of irradiation with three equivalents of **6b**, we observed an 86% NMR yield of the 1,3-dichlorinated product **9a**. However, further attempts to improve the yield by increasing the amount of **6b** or prolonging the reaction time were unsuccessful. We then examined Willgerodt-type reagents **6a,c–e**. Interestingly, a quantitative yield of **9a** was formed after 15 min of irradiation, and only 1.2 equiv of **6a** were needed to achieve complete conversion. In contrast to electrophilic chlorination,^14b^ the electronic structure of the aromatic ring did not affect the reactivity of **6**, giving **9a** quantitatively with either electron-rich (**6c**) or electron-poor reagents (**6d**–**e**). Further screening of other common solid-nitrogen-based chlorinating reagents **6f**–**h** did not deliver the product, demonstrating the unique properties of the Willgerodt-type reagents under photolysis. Further screening of solvents showed that chloroform, acetonitrile, and ethyl acetate could also be used with a slight decrease in yield. The reaction was less efficient in acetone, methanol, water/acetonitrile mixtures, or THF. Under blue LED irradiation (λ = 450, 440, or 427 nm), the reaction in DCM proceeded to full conversion, yielding the product quantitatively. In comparison, replacing the light source with a green LED strip (525 nm) resulted in only trace amounts of the product. This result is in good accordance with the reported absorption spectra of **6a**, showing significant absorbance up to 500 nm.^12c^

**Scheme 2:**
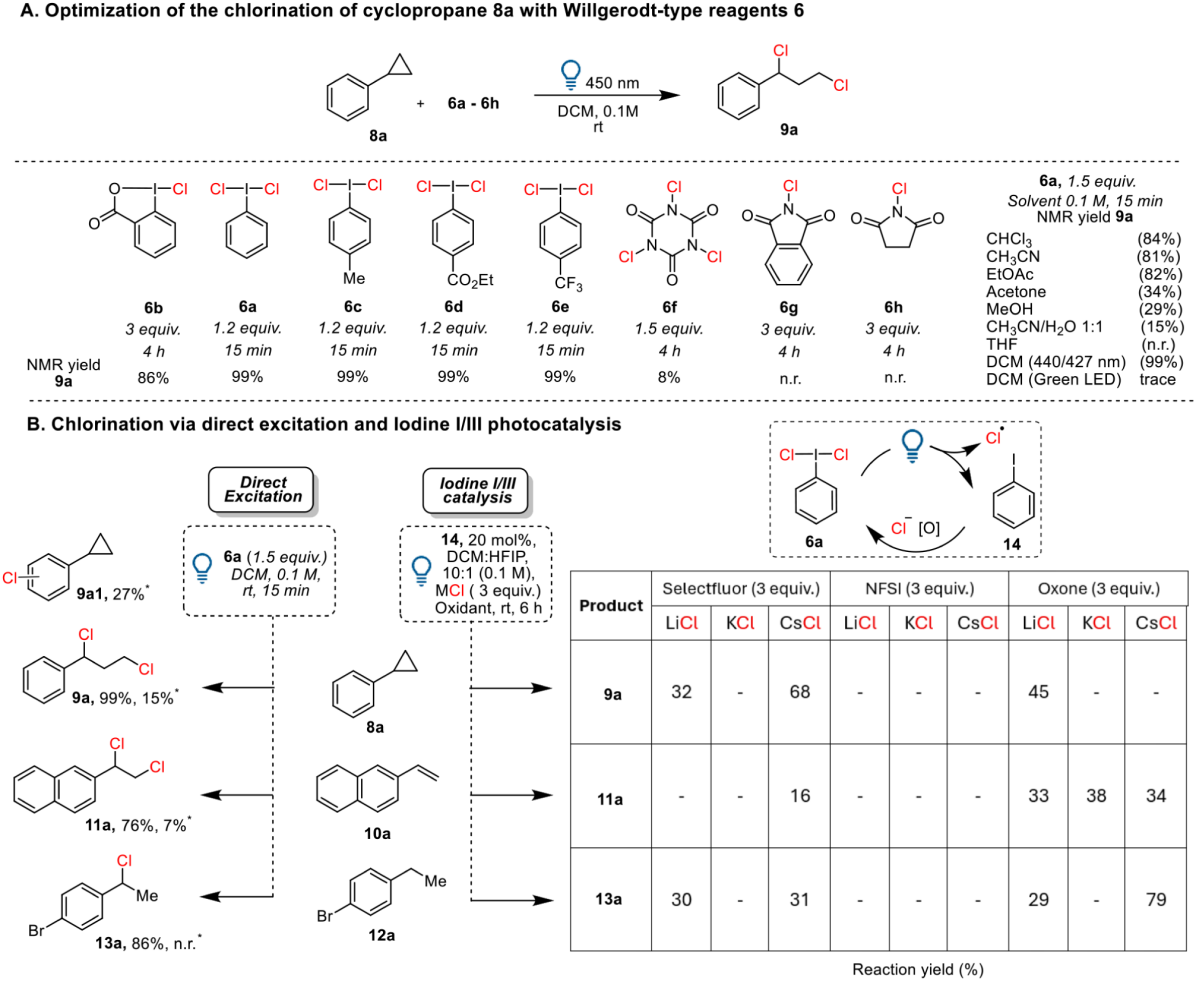
Reaction Optimization and Control Experiments Using 450 nm Blue LED—30 W^a^

a NMR yield is reported using CH_2_Br_2_ as an internal standard. n.r. = no reaction. ^*^NMR yields when performing reactions in the dark for 1 h.

To our delight, the same conditions could be applied for the 1,2-chlorination of vinyl naphthalene **10a** and the C–H chlorination of ethyl arene **12a**, delivering 76% of **11a** and 86% of **13a** (Scheme 2B). In the case of **10a** and **12a**, 1.5 equiv of **6a** were required to reach complete conversion. Performing a control experiment in the dark for the chlorination of **8a** for 1 h resulted in 27% of aromatic chlorination **9a1**, 15% of **9a**, and 54% of recovered cyclopropane **8a**. When heated to 40 °C in the dark, **9a** was obtained in 12% yield together with **9a1**, whereas irradiating at 0 °C still led to the formation of **11a** in 95% NMR yield. Only 7% of **11a** was observed starting from **10a**, and no product was detected with **12a**. These results demonstrated the dominance and higher efficiency of the radical pathway under irradiation. Furthermore, the dichlorination of **8a** did not take place in the presence of TEMPO, and a TEMPO chlorination adduct was detected by mass spectroscopy. These results support a speculative mechanism involving the formation of a benzylic radical, probably via an intermediate arylcyclopropyl radical cation (see Supporting Information Section S9 for details).

We consistently observed a large amount of iodobenzene (**14**) remaining after the irradiation of **6a**. Therefore, we envisioned that a catalytic process could be achieved by adding chlorine salt and an oxidant to regenerate 6a (Scheme 2C). Following a protocol reported by Gilmour and coworkers,^14b^ we screened reactions in parallel with different oxidants, including Selectfluor, NFSI, oxone, and chlorine salts (LiCl, KCl, CsCl) under visible-light irradiation with 20 mol % iodobenzene (**14**) as the catalyst. We observed the formation of products **9a**, **11a**, and **13a** when either Selectfluor or Oxone was used as the oxidant. NFSI did not result in product formation for any of the three substrates. We found that the best conditions for C–C chlorination were using CsCl with Selectfluor as the oxidant, yielding 68% product **9a**. Oxone proved to be the best oxidant for chlorination of **10a** and **12a**, delivering 38% **11a** and 79% **13a**. However, several background reactions were observed and affected the reaction efficiency, such as fluorination in the case of **8a**, polymerization of **10a**, and aromatic chlorination on **12a**. Overall, the possibility of performing chlorinations directly from chloride salts under iodine­(I/III) photocatalysis has been demonstrated. Nevertheless, the use of stoichiometric **6a** still delivered better results without side reactions and required a shorter reaction time than the catalytic protocol. Given that **6a** is an easily accessible reagent from iodobenzene (**14**),^18^ we decided to continue to explore the substrate scope using stoichiometric amounts of **6a**.

The substrate scope of aryl cyclopropanes **8** for the 1,3-chlorination is shown in Scheme 3A. The desired products were obtained cleanly, and no aromatic chlorination was observed regardless of the electronic properties of the phenyl ring (products **9a–h**). The isolated yield of **9a** was only 80% compared to the quantitative NMR yield due to its instability during column chromatography. We encountered similar issues with products **9b** and **9c**. Product **9b** was even fully hydrolyzed by column chromatography to give the corresponding benzylic alcohol in 51% yield. The reaction tolerated various functionalities, including a bromo (**9d**), a cyanide (**9e**), an acetyl (**9f**), a pinacol boronate (**9g**), and a naphthyl group (**9h**), with isolated yields ranging from 63% to 91%. A diphenyl-substituted cyclopropane resulted in a 79% yield of product **9i**. A donor–acceptor cyclopropane with a diester functional group delivered 68% of products **9j**. Interestingly, the reaction tolerated a carboxylic acid and a pyridine derivative, giving 91% yield of **9k** and 74% yield of **9l**. It has been reported that pyridines can substitute for chloride on **6a**,^21^ and carboxylic acids are usually incompatible with reactions involving electrophilic HIRs.^22^ The products observed demonstrated that photolysis was fast enough to promote the radical pathway over other reactions. The protocol also allowed the late-stage chlorination of heterocyclic bioactive molecules, yielding chlorinated products **9m**, derived from the drug Lesinurad, in 42% yield and **9n** in 59% yield. It is worth mentioning that the thioether in **9m** was tolerated, even if it could be sensitive to oxidation.

**Scheme 3:**
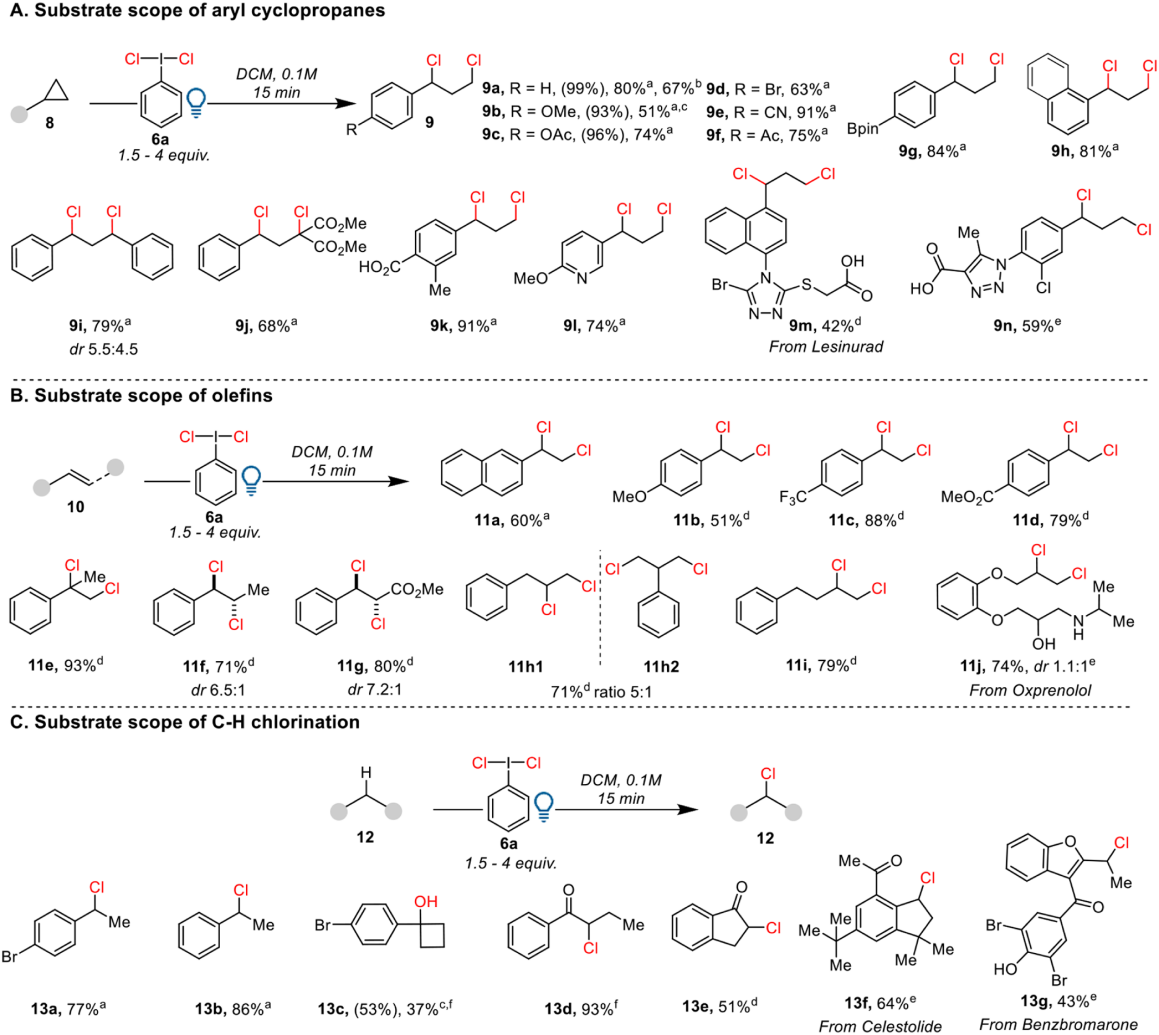
Substrate Scope of Chlorination Using 450 nm Blue LED—30 W[Fn sch3-fn1]

i Yields in brackets are NMR yields with CH_2_Br_2_ as an internal standard. Scope at 0.2 mmol scale. ^a^1.5 equiv of **6a**. ^b^At 1 mmol scale. ^c^The chlorinated compound was hydrolyzed at the benzylic position on reverse-phase chromatography, and the corresponding alcohol was isolated. ^d^2.0 equiv of **6a**. ^e^2.5 equiv of **6a**. ^f^4.0 equiv of **6a**.

We then explored the substrate scope of the olefins (Scheme 3B). In general, styrene derivatives having an electron-donating group (products **11a**,**b**) gave lower yields than those with an electron-withdrawing group (**11c**,**d**), likely due to instability of the products during column chromatography. 1,1-Disubstituted or 1,2- disubstituted styrene derivatives delivered 1,2-dichlorination products in high yields (**11e**: 93%, **11f**: 71%, **11g**: 80%). Despite having opposite electronic properties, both **11f** and **11g** can be obtained in good yields, demonstrating the generality of radical chlorination compared to classical electrophilic chlorination. The protocol was also successfully applied to nonactivated olefins (products **11h**–**j**). In the case of allyl benzene, an *ipso* rearrangement was observed, giving a 5:1 mixture of **11h1** and **11h2** in 71% yield. Product **11i** was isolated in 79% yield starting from 4-phenyl-1-butene. Although it was reported that **6a** can be used as an efficient reagent to oxidize alcohols,^15^ the reaction shows good tolerance to both alcohols and amines, yielding 74% of product **11j** starting from the drug Oxprenolol.

Further exploration of the substrate scope for C–H chlorination revealed excellent yields with 1-bromo-4-ethylbenzene and ethylbenzene (products **13a** and **13b**) (Scheme 3C). When starting from an aryl cyclobutane, we obtained C–H functionalization instead of C–C cleavage, resulting in cyclobutyl alcohol **13c** after isolation by reverse-phase column chromatography. We were also able to afford C–H chlorination of α-carbonyl C–H bonds, yielding 93% of isolated product **13d**, although four equivalents of **6a** were necessary to complete the reaction. Starting from the substrate having both secondary benzylic and α-carbonyl C–H bonds, **13e** was isolated as the major product. Only a trace amount of benzylic chlorination was observed in the crude mixture. Interestingly, we obtained product **13f** in 64% yield without chlorination of the acetyl group, likely due to the unfavorable formation of a primary radical on the acetyl group.^23,24^ The protocol was successful in the case of Benzbromarone, giving 43% yield of product **13g.** C–H chlorination products can also be obtained from cyclic substrates such as α-tetralone and the drug ibuprofen (see Supporting Information).

The simplicity of the protocol allowed us to use benzylic chlorides as intermediates for a telescoped functionalization (Scheme 4A). We focused on 1,1-diaryl motifs, which are frequently encountered in bioactive molecules (Scheme 1E).^19,20^ Following modified reported conditions for Friedel–Crafts arylation,^200^ we successfully performed a one-pot chlorination/arylation sequence, resulting in 1,1-diaryl-3-chloro scaffolds **15** starting from cyclopropanes **8**. The nucleophilic substitution happened at room temperature in the presence of iron­(III) chloride^200^ and a base, and purification of the chlorinated intermediate was not required. Exclusive substitution at the benzylic position, better suited to stabilizing positive charges, was observed. Cyclopropane **8a** was converted to **15a** in 77% yield after two steps. The reaction was successful for both bromo- and boron-substituted substrates (products **15b**,**c**). However, the nucleophilic substitution is not efficient with cyclopropanes bearing electron-withdrawing groups, as exemplified by product **15d** obtained in only 26% yield, likely due to the difficulty of forming the benzylic carbocation. Using phenol as a nucleophile exclusively delivered aromatic substitution over *O*-alkylation, giving 52% yield of **15f** as a *para/ortho* mixture in a 7:1 ratio. Products **15g** and **15h** were obtained in 76% and 66% yields, respectively, using 1,3-benzodioxole and benzothiophene as nucleophiles. Arylation product **15i** was obtained in 45% yield starting from Indomethacin methyl ester. The drugs Nimesulide and Estrone can also be used for aromatic substitution (products **15j** and **15k**), demonstrating the possibility for late-stage functionalization of bioactive compounds. We also explored the same reaction conditions with sulfonamides as nucleophiles, obtaining good yields from *para-*methoxyphenyl sulfonamide (product **15l**, 75%) and the drug Celecoxib (**15m**, 70%). In this case, one equivalent of iron chloride was required to accelerate the reaction. Alcohol and thiol nucleophiles can also be used under the same reaction conditions, giving products **15n**–**p** in 65–74% yield. Additionally, C–H arylation and 1,2-chloro-arylation of styrene derivatives can be achieved, yielding 61% of product **15q** and 60% of product **15r**.

**Scheme 4:**
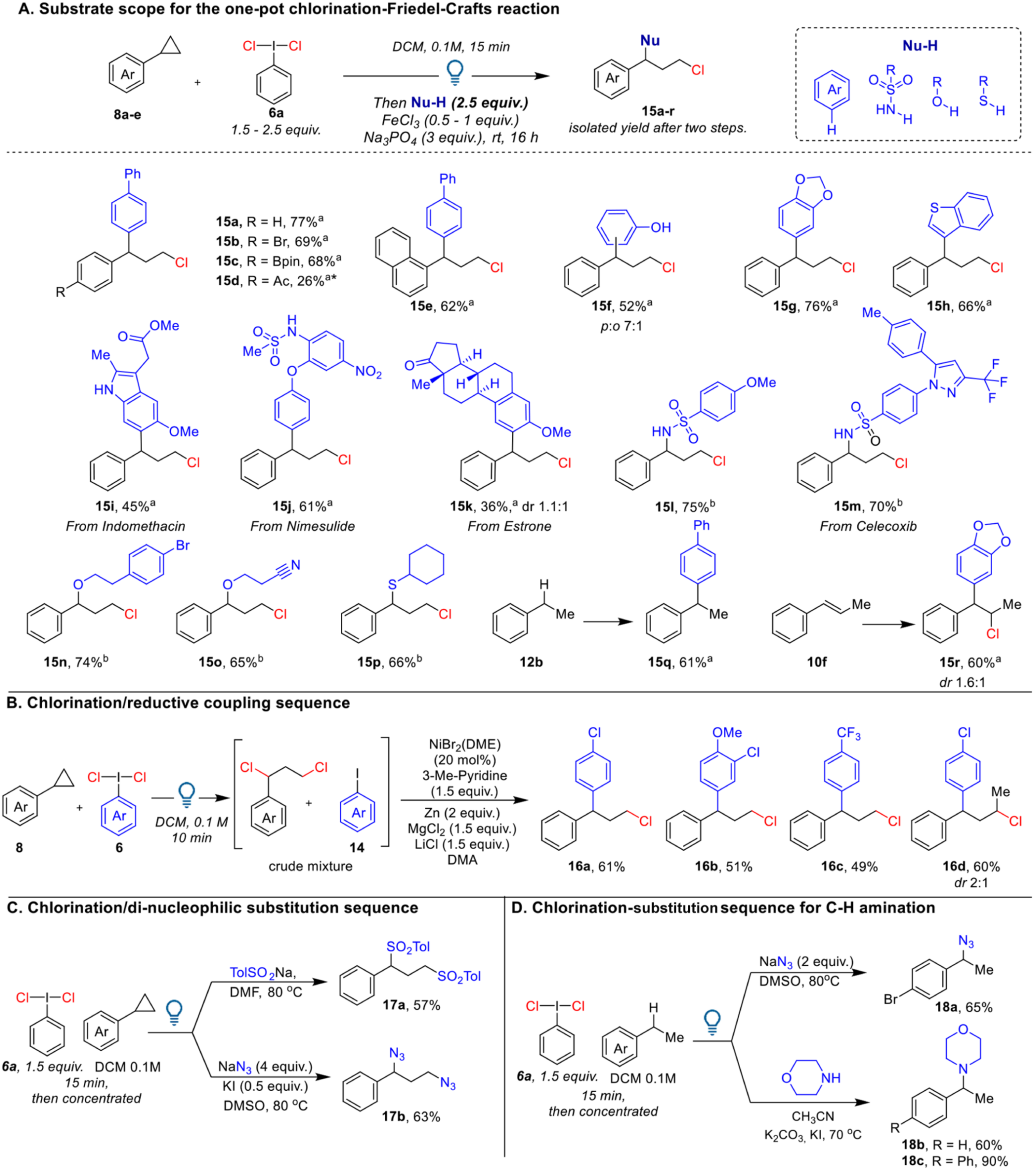
Two-Step Protocol for Benzylic Functionalization^a^

a Scope on 0.2 mmol scale using 450 nm Blue LED—30 W. ^a^0.5 equiv of FeCl_3_. ^b^1.0 equiv of FeCl_3_. ^*^72 h of reaction.

During scope exploration, we often noticed the presence of a byproduct resulting from benzylic arylation with iodobenzene **14.** This suggests the possibility of reusing **14** as a substrate in the C–C bond-forming step. Since **14** has low reactivity in Friedel–Crafts-type arylation, we envisaged the use of a nickel-catalyzed reductive coupling of **14** with the benzylic chloride intermediate to give a 1,1-diaryl scaffold in an atom-economic manner (Scheme 4B). Following modified reported conditions^25^ (see SI for detailed optimization), we carried out the reductive coupling directly from the concentrated crude mixture after ring-opening chlorination. After two steps, we successfully obtained chloro-arylation products **16a**–**d** with yields ranging from 49% to 61%. This approach is suitable for both electron-poor and electron-rich aromatic partners, making it complementary to the Friedel–Crafts-type reaction.

We expected that 1,3-chlorinated compounds could also serve as dielectrophilic intermediates (Scheme 4C). A double substitution was conducted with sodium sulfonate and sodium azide, resulting in a 57% yield of disulfonate **17a** and 63% yield of diazidation product **17b** over two steps. Lastly, we used the same method to perform a telescoped C–H benzylic functionalization with azide and morpholine nucleophiles, producing C–H amination products **18a**–**c** (Scheme 4D). All of the reactions were conducted without purification of the chlorinated intermediates, demonstrating the practicability of our method.

In conclusion, we have developed a general protocol for chlorinating cyclopropanes, olefins, and activated C–H bonds using direct photoexcitation of the Willgerodt reagent (**6a**). The conditions are mild, and the reaction is practical to set up, with a wide range of functional groups tolerated. Additionally, we demonstrated that photomediated chlorination using Iodine­(I/III) catalysis is possible, which serves as a complementary approach to the stoichiometric use of the Willgerodt reagent (**6a**). Taking advantage of the easily accessible benzylic chlorides, we have developed a one-pot protocol for further substitution with C, N, O, and S nucleophiles, and for repurposing the aryl iodide byproduct in a reductive cross-coupling. Our work provides a practical approach to functionalize cyclopropanes, olefins, and activated C–H bonds via the formation of organo-chlorine intermediates, and we believe it will, therefore, be of interest for accessing useful building blocks in synthetic and medicinal chemistry.

## Supplementary Material



## Data Availability

The data that support the findings of this study are available in the Supporting Information of this article.

## Notes

General methods, synthetic procedures, optimization details, and characterization data for all synthesized compounds; a copy of NMR spectra for new compounds; raw data for compound characterization made at UMP and EPFL (PDF)
