# Supplementary material for: Synthesis of Csp 3 Chlorinated Compounds from Cyclopropanes, Olefins, and C–H Bonds via Photolysis of Willgerodt-Type Reagents
Source: JACS Au. 2026 Jan 12;6(1):290–8. doi: 10.1021/jacsau.5c01226 (PMC12848673; doi:10.1021/jacsau.5c01226)

Supporting Information

Synthesis of C*sp*^{3} Chlorinated Compounds from Cyclopropanes, Olefin and C-H Bonds via photolysis of Willgerodt-type reagents.

Tin V. T. Nguyen, Thanh V. Q. Nguyen, Trinh T. H. Tran, Qui-Hien Nguyen, and Jerome Waser.

Contents

1. General methods 2
2. Synthesis of hypervalent iodine reagent....................4
3. Cyclopropanes synthesis 7
4. Protocol for chlorination. 10
5. One-pot functionalization. 30
6. Sequence chlorination/reductive coupling 43
7. Sequence of chlorination/dielectrophilic substitution 48
8. Sequence for C-H amination 49
9. Preliminary mechanistic study 52
10. NMR spectrum. 53

1. General methods

1D and 2D NMR was recorded on Bruker 400 MHz, 500 MHz or 600 MHz spectrometers and chemical shifts are given in parts per million (ppm) relative to residual solvent peaks: 2.50 ppm (^{1}H) and 39.52 ppm (^{13}C) for DMSO-d6, 7.26 ppm (^{1}H) and 77.16 ppm (^{13}C) for $CDCl_3$. Data are presented as follows: chemical shift (ppm), multiplicity (s = singlet, d = doublet, t = triplet, q = quartet, m = multiplet), coupling constant J (Hz) and integration. High resolution mass spectrometric measurements were performed by the mass spectrometry service of ISIC at the EPFL on a MICROMASS (ESI) Q-TOF Ultima API. Analysis: TLC was performed on pre-cut glass-based silica plate. LCMS was carried on a Water Acquity UPLC system equipped with an autosampler (2 µL loop), a thermo-stated column compartment, a PAD detector and a QDa detector. The separation was performed on a HSS C18 column (50 × 2.1 mm, 1.8 µm) at 60 °C with a flow rate of 1 mL/min, using a gradient from 10 to 99% (v/v) of eluent A in eluent B over 3.5 min. Eluent A: MeCN/water = 95/5; eluent B: pH3 buffer (aqueous solution containing 10 mM HCO_2H and 1 mM NH_3). Purification: Column chromatography was performed on Biotage Selekt using Biotage Sfär HC D columns (normal phase) or Biotage Sfär C18 D columns (reversed phase) and 254 nm as the collection wavelength. Gradient condition was given for each compound in the experimental procedure. All chemicals were purchased and used as received unless stated otherwise.

High-throughput photochemistry was performed using standards Para-dox Photoredox Golden Plate (purchased from Analytical Sales and Services, SKU 96973, top left). Reaction mixtures were prepared in 1 mL clear glass shell vials (SKU 84001-CASE) equipped with 6x3 mm magnetic stirring bars. The vials were inserted in 24 positions in the middle of the plate (right) to minimize differences in stirring and temperature. The plate was placed on top of a Lumidox II LED 470 nm array (SKU LUM296LS470, middle left). The use of Lumidox II Controller (SKU LUM2CON, bottom left) manipulated the energy input of 150 mW per well (stage 2/5). The whole system was cooled using 3 TTDs (SKU 266540, one under the LED array, two along the long sides of the plate). These TTDs were kept cold with flowing mixtures of propylene glycol and water (pre-cooled at 10 °C in a circulation chiller). AstraZeneca team used a HepatoChem Photo RedOx Box (US patent #10,906,022) equipped with a 450 nm EvoluChem Blue LED – 30W. At EPFL, Kessil lamps (440 nm, 44 W) were used. The hood was free and coated with aluminum foil for personal protection. The reactions were cooled by built-in fan. Long radiation resulted in temperature increasing up to 50 °C during overnight reactions unless a fan was used, in which case the temperature raised to 30-35 °C.

Photochemical set-up

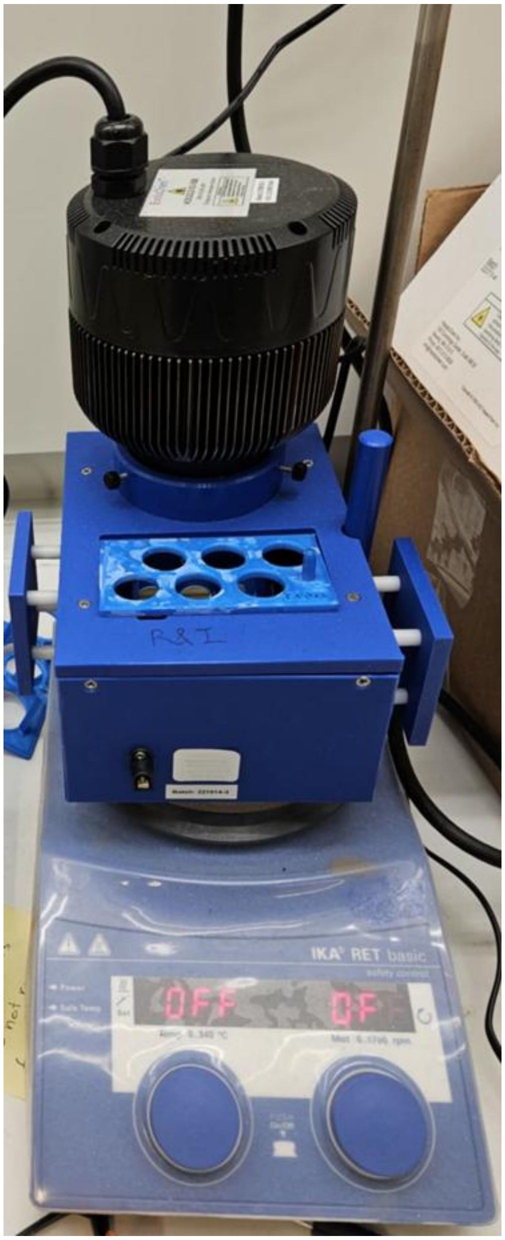

2. Synthesis of hypervalent iodine reagent

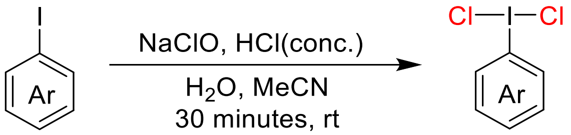

General procedure A: according to the reported procedure,[1] concentrated HCl (20 mL) was added dropwise over 2 min to a stirring solution of iodobenzene (10 mmol) in aq 13-14% NaOCl soln (20 mL) at r.t, 40 mL of water and 20mL of MeCN as co-solvent. A yellow solid precipitated immediately and the reaction was complete after 30 minutes – 2 hours. The resulting yellow solid was collected by filtration, washed with H_2O and light petroleum ether, and dried at r.t. in the dark overnight. The yellow solid was identified to be $ArICl_2$ by comparison of its ^{1}HNMR with that reported in the literature.

1-Chloro-1,2- benziodoxol-3(1H)-one (6a)

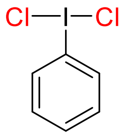

Following the general procedure A, from iodobenzene (1.120 mL, ~10.0 mmol), obtaining **6a** (2.45 g, 8.91 mmol, 89%) as a yellow solid. **^{1}H NMR** (400 MHz, $CDCl_3$) δ 8.23 – 8.15 (m, 2H, Ar*H*), 7.65 – 7.56 (m, 1H, Ar*H*), 7.52 – 7.45 (m, 2H, Ar*H*). Consistent with reported value.[1]

Dichloro(*p*-tolyl)-λ^3-iodane (6c)

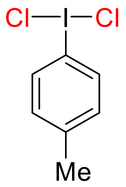

Following the general procedure A, using *p*-iodotoluene (2.19 g, 10.0 mmol, 1.00 equiv), obtaining dichloro(*p*-tolyl)-λ^3-iodane **6c** as a yellow solid (2.93 g, 10.0 mmol, 99%). **^{1}H NMR** (400 MHz, $CDCl_3$) δ 8.08 – 8.00 (m, 2H, Ar*H*), 7.27 (d, *J* = 7.9 Hz, 2H, Ar*H*), 2.47 (s, 3H, C*H*$_3$). Consistent with reported value.[1]

[1] Sarie, J. C.; Neufeld, J.; Daniliuc, C. G.; Gilmour, R. Willgerodt-Type Dichloro(aryl)-λ3-Iodanes: A Structural Study. *Synthesis* **2019**, *51*, 4408.

Ethyl 4-(dichloro-λ³-iodaneyl)benzoate (6d)

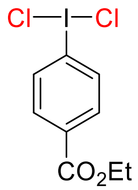

Following the general procedure A, using ethyl 4-iodobenzoate (1.17 mL, 10.0 mmol, 1.00 equiv), yielding ethyl 4-(dichloro-λ³-iodaneyl)benzoate **(6d)** as a yellow solid (2.79 g, 8.10 mmol, 81%). **^{1}H NMR** (400 MHz, $CDCl_3$) δ 8.32 – 8.24 (m, 2H, Ar*H*), 8.16 – 8.08 (m, 2H, Ar*H*), 4.43 (q, *J* = 7.1 Hz, 2H, CH_2CH_3), 1.41 (t, *J* = 7.1 Hz, 3H, CH_2CH_3). Consistent with reported value.[1]

Dichloro[4-(trifluoromethyl)phenyl]-λ³-iodane (6e)

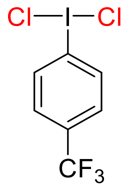

Following the general procedure A, using 1-iodo-4-(trifluoromethyl)benzene (1.47mL, 10.0 mmol, 1.0 equiv), yielding dichloro[4-(trifluoromethyl)phenyl]-λ³-iodane **(6e)** as a yellow solid (2.85 g, 8.45 mmol, 85%). **^{1}H NMR** (400 MHz, $CDCl_3$) δ 8.35 (dd, *J* = 9.0, 0.8 Hz, 2H, Ar*H*), 7.78 – 7.70 (m, 2H, Ar*H*). Consistent with reported value.[1]

Dichloro(4-chlorophenyl)-λ³-iodane (6i)

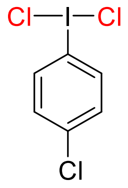

Following the general procedure A, using 1-chloro-4-iodobenzene (2.40 g, 10.0 mmol, 1.0 equiv), yielding dichloro(4-chlorophenyl)-λ³-iodane **(6i)** as a yellow solid (2.91 g, 9.40 mmol, 94%). **^{1}H NMR** (400 MHz, $CDCl_3$) δ 8.16 – 8.07 (m, 2H, Ar*H*), 7.49 – 7.40 (m, 2H, Ar*H*). Consistent with reported value.[1]

Dichloro(3-chloro-4-methoxyphenyl)- λ^3-iodane (6j)

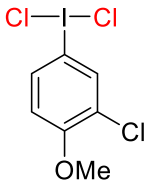

Following the general procedure A using 1-iodo-4-methoxybenzene (2.34 g, 10.0 mmol, 1.00 equiv), yielding dichloro(4-methoxyphenyl)-λ^3-iodane (**6j**) as a yellow solid (881 mg, 2.40 mmol, 24%). **^{1}H NMR** (400 MHz, $CDCl_3$) δ 8.15 (d, *J* = 2.5 Hz, 1H, Ar*H*), 8.03 (dd, *J* = 9.0, 2.5 Hz, 1H, Ar*H*), 6.96 (d, *J* = 9.1 Hz, 1H, Ar*H*), 3.99 (s, 3H, CH_3).

*Starting materials: **8a**, **8d – 8k**, **8m**, **8n**, **10a – 10j**, **12a – 12j**, are commercially available and were used as received without further purification*

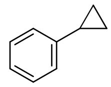
8a

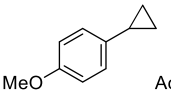
8b

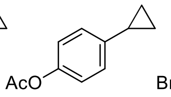
8c

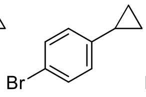
8d

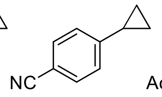
8e

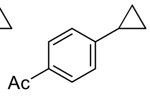
8f

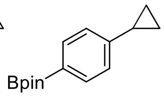
8g

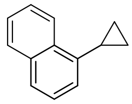
8h

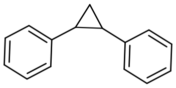
8i

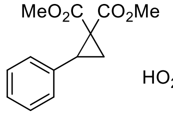
8j

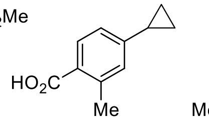
8k

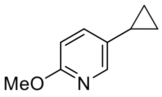
8l

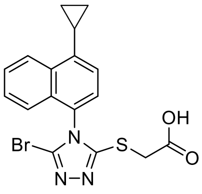
8m, *Lesinurad*

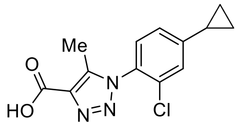
8n

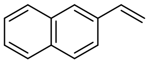
10a

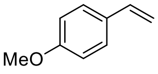
10b

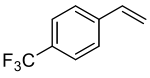
10c

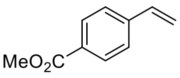
10d

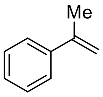
10e

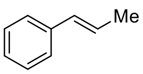
10f

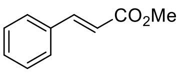
10g

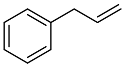
10h

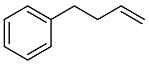
1oi

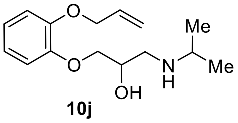
10j

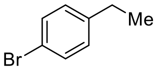
12a

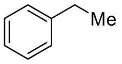
12b

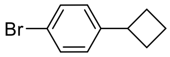
12c

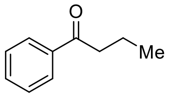
12d

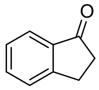
12e

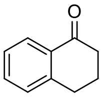
12f

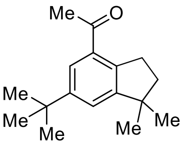
12g, *Celestolide*

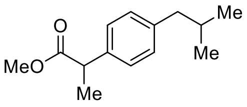
12h, *buprofen*

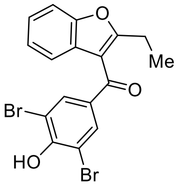
12i, *Benzbromarone*

3. Cyclopropanes synthesis

General procedure B

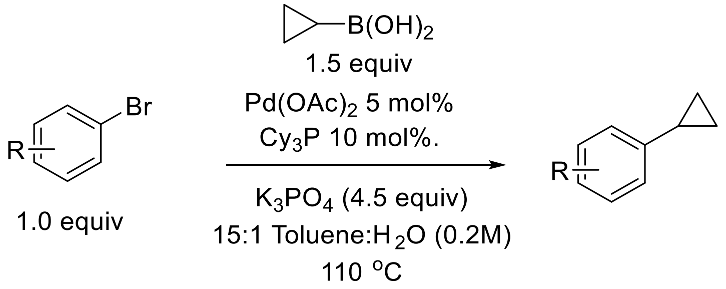

Following a reported procedure,[2] to a solution of aryl bromide (5.00 mmol, 1.00 equiv) in 15:1 toluene:water (32 mL) was added potassium phosphate tribasic (4.78 g, 22.5 mmol, 4.50 equiv), cyclopropylboronic acid (644 mg, 7.50 mmol, 1.50 equiv), tricyclohexylphosphine (0.5 ml, 1M solution in toluene, 0.010 equiv.) and palladium(II) diacetate (56.1 mg, 250 µmol, 0.050 equiv). The resulting mixture was heated to 110 °C and was stirred at this temperature overnight. The reaction mixture was then cooled to room temperature, diluted with DCM (100 mL) and washed with water (100 mL). The aqueous layer was extracted with DCM (3 × 100 mL). The combined organic layers were washed with brine (50 mL), dried over Na_2SO_4, filtered and concentrated under reduced pressure. The crude mixture was purified by column chromatography on Biotage (Büchi flashpure cartridge 40 g, gradient of pentane: ethyl) to give the product.

1-Cyclopropyl-4-methoxybenzene (8b)

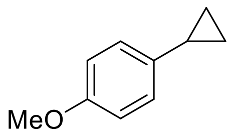

The cyclopropane was already synthesized in our previous project,[2] following general procedure B, starting from 1-bromo-4-methoxybenzene (935 mg, 5.00 mmol, 1.00 equiv), 1-cyclopropyl-4-methoxybenzene **8b** (580 mg, 3.91 mmol, 78% yield) was obtained as a colorless liquid. **^{1}H NMR** (400 MHz, $CDCl_3$) δ 7.09 – 6.97 (m, 2H, Ar*H*), 6.90 – 6.77 (m, 2H, Ar*H*), 3.78 (s, 3H, OC*H*$_3$), 1.86 (m, 1H, C*H*CH$_2$), 0.98 – 0.84 (m, 2H, CHC*H*$_2$), 0.71 – 0.57 (m, 2H, CHC*H*$_2$). **^{13}C NMR** (101 MHz, $CDCl_3$**)** δ 157.7, 136.0, 127.0, 113.9, 55.4, 14.7, 8.6.

4-cyclopropylphenyl acetate (8c)

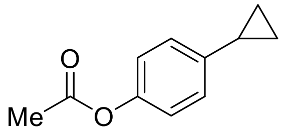

Following general procedure B, starting from 1-bromo-4-acetoxybenzene (1075 mg, 5.000 mmol, 1.00 equiv), 4-cyclopropylphenyl acetate **8c** (625 mg, 3.55 mmol, 71% yield) was obtained as a pale-yellow liquid. **^{1}H NMR** (400 MHz, $CDCl_3$) δ 7.13 – 7.02 (m, 2H, Ar*H*), 7.02 – 6.92 (m, 2H, Ar*H*), 2.28 (s, 3H, C*H*$_3$), 1.97 – 1.77 (m, 1H, CH$_2$C*H*), 1.02 – 0.88 (m, 2H, C*H*$_2$), 0.74 – 0.58 (m,

2H, CH_2). **^{13}C NMR** (101 MHz, $CDCl_3$) δ 169.9, 148.5, 141.7, 126.8, 121.5, 21.3, 15.1, 9.2. NMR data consistent with reported value.[2]

5-cyclopropyl-2-methoxypyridine (8l)

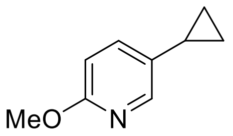

Following general procedure B, starting from 5-bromo-2-methoxypyridine (940 mg, 5.00 mmol, 1.00 equiv), 5-cyclopropyl-2-methoxypyridine **8l** (507 mg, 3.40 mmol, 68% yield) was obtained as a pale-yellow liquid. **^{1}H NMR** (400 MHz, $CDCl_3$) δ 7.97 (d, J = 2.5 Hz, 1H, ArH), 7.23 (dd, J = 8.5, 2.5 Hz, 1H, ArH), 6.64 (dd, J = 8.5, 0.7 Hz, 1H, ArH), 3.90 (s, 3H, OCH_3), 1.91 – 1.74 (m, 1H, CH), 0.99 – 0.83 (m, 2H, CH_2), 0.72 – 0.49 (m, 2H, CH_2). **^{13}C NMR** (101 MHz, $CDCl_3$) δ 162.7, 144.9, 136.4, 131.8, 110.5, 53.5, 12.3, 8.1. NMR data consistent with reported value.[3]

[2] Gieuw, M. H.; Ke, Z.; Yeung, Y. Lewis Base-Promoted Ring-Opening 1,3-Dioxygenation of unactivated cyclopropanes using a hypervalent iodine reagent, *Angew. Chem. Int. Ed.* **2018**, *57*, 3782–3786.
[3] Molander, G. A.; Gormisky, P. E. Cross-Coupling of Cyclopropyl- and Cyclobutyltrifluoroborates with Aryl and Heteroaryl Chlorides. *J. Org. Chem.* **2008**, *73*, 7481–7485.

4. Protocol for chlorination.

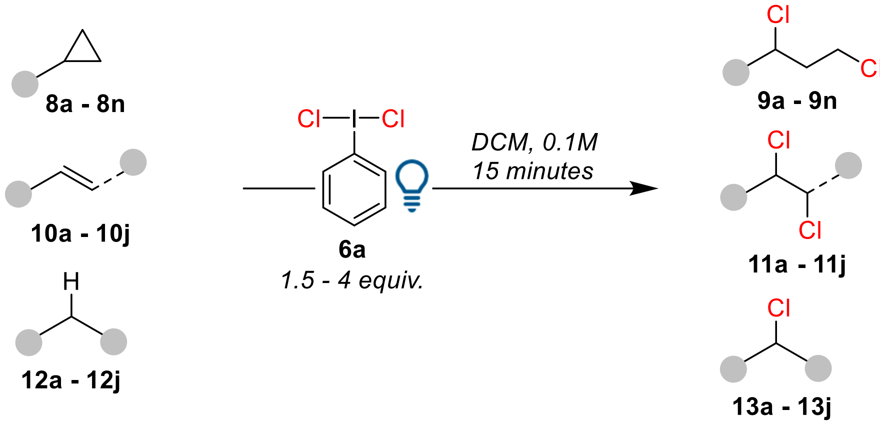

General procedure C. In a 4 mL Vial, dichloro(phenyl)-λ3-iodane (1.5 equiv – 4.0 equiv.) was added. Then the starting material was added (0.20 mmol, 1.0 equiv) if as solid, followed by addition of DCM (2.0 mL, 0.1 M). The starting material as liquid was added after DCM addition. The vial was flushed with nitrogen for 10 seconds before sealing with a cap. The reaction mixture was stirred at room temperature under irradiation of Evolution chem 30 W LED (I_{max} = 450 nm) for 15 minutes. The reaction was monitored by NMR with CH_2Br_2 as internal standard using signal of ArC*H*Cl on the products. Upon completion the mixture was concentrated in vacuo and purified by column chromatography on Biotage.

(1,3-Dichloropropyl)benzene (9a)

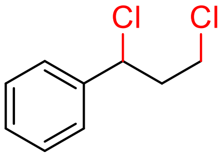

Following the general procedure C, starting from cyclopropylbenzene **8a** (23.6 mg, 0.200 mmol, 1.0 equiv) and **6a** (82 mg, 0.30 mmol, 1.5 equiv), NMR yield was analysed after 15 minutes using CH_2Br_2 (14 μl, 0.20 mmol, 1 equiv) as internal standard. Product was purified by column chromatography on Biotage (heptane 100%) to obtain product **9a** (30.3, 0.160 mmol, 80%) as colorless oil. **^{1}H NMR** (400 MHz, $CDCl_3$) δ 7.47 – 7.29 (m, 5H, Ar*H*), 5.14 (dd, *J* = 9.0, 5.4 Hz, 1H, ArC*H*Cl), 3.82 – 3.71 (m, 1H, ArCHCH_2CH_2Cl), 3.63 – 3.52 (m, 1H, ArCHCH_2CH_2Cl), 2.63 – 2.49 (m, 1H, ArCHCH_2), 2.48 – 2.33 (m, 1H, ArCHCH_2).**^{13}C NMR** (101 MHz, $CDCl_3$) δ ^{13}C NMR

(101 MHz, $CDCl_3$) δ 140.7, 129.0, 128.8, 127.1, 77.5, 77.2, 76.8, 60.1, 42.4, 42.0. The analytical data matched that previously reported.[4]

1 mmol scale reaction

Starting from cyclopropylbenzene **8a** (128 mg, 1.00 mmol, 1.00 equiv), **6a** (410 mg, 1.50 mmol, 1.50 equiv), 10 ml of DCM. The reaction was conducted in a 25 ml Schlenk tube under irradiation for 30 minutes. Product was purified by column chromatography (SiO_2, hexane 100%) to obtain product **9a** (126 mg, 0.667 mmol, 67%) as colorless oil.

3-Chloro-1-(4-methoxyphenyl)propan-1-ol (9b)

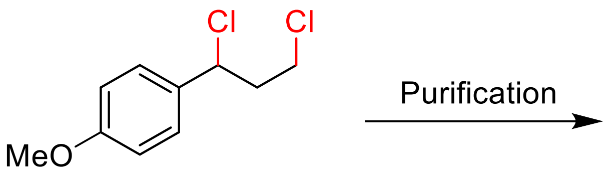

Following general procedure C, starting from cyclopropane **8b** (29.2 mg, 0.200 mmol, 1.00 equiv) and **6a** (82.0 mg, 0.300 mmol, 1.50 equiv), NMR yield was analysed after 15 minutes using CH_2Br_2 (14 µl, 0.20 mmol, 1 equiv) as internal standard.

[4] Lopez, M. A.; Buss, J. A.; Stahl, S. S. Cu-Catalyzed Site-Selective Benzylic Chlorination Enabling Net C–H Coupling with Oxidatively Sensitive Nucleophiles. *Org. Lett.* **2021**, *24*, 597–601.

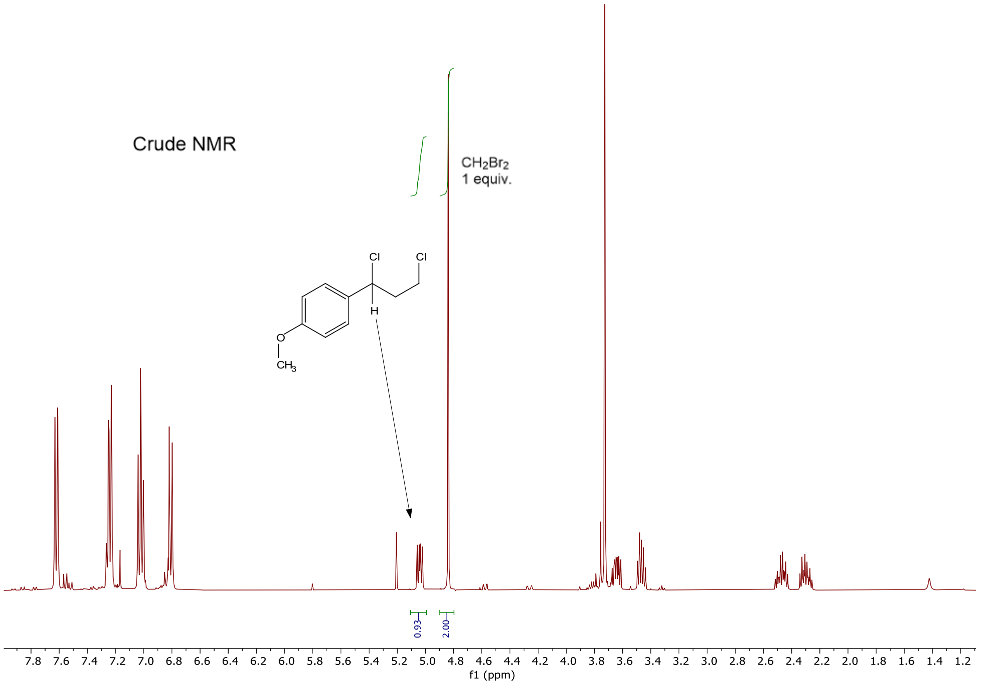

The product is unstable and hydrolyzed on reverse phase column chromatography on Biotage (gradient 5% to 95% of CH_3CN – water 0.1% formic acid) to give alcohol **9b** (20.5 mg, 0.103 mmol, 51 %) as colorless oil. TLC (Pentane/Et_2O = 4/1) Rf = 0.25. **^{1}H NMR** (400 MHz, $CDCl_3$) δ 7.33 – 7.26 (m, 2H), 6.92 – 6.87 (m, 2H), 4.89 (dd, *J* = 8.3, 4.9 Hz, 1H, PhC*H*), 3.81 (s, 3H, OC*H₃*), 3.76 – 3.67 (m, 1H, ClCH_2CH₂), 3.59 – 3.49 (m, 1H, ClCH_2CH₂), 2.30 – 2.18 (m, 1H, ClCH₂CH_2), 2.12 – 2.01 (m, 1H, ClCH₂CH_2). 1.96 (s, 1H, O*H*). **^{13}C NMR** (101 MHz, $CDCl_3$) δ 159.4, 135.9, 127.2, 114.2, 71.1, 55.4, 41.9, 41.5. **HRMS** (Sicrit plasma/LTQ-Orbitrap) m/z: Calcd for $C_{10}H_{12}ClO^+$ 183.0571; Found 183.0572. NMR matched the reported value.[5]

4-(1,3-Dichloropropyl)phenyl acetate (9c)

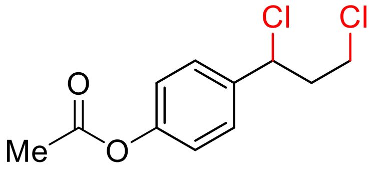

Following general procedure C, starting from cyclopropane **8c** (35.2 mg, 0.200 mmol, 1.00 equiv) and **6a** (82.0 mg, 0.300 mmol, 1.5 equiv). NMR yield was analysed after 15 minutes

[5] Sheng, W.; Huang, X.; Cai, J.; Zheng, Y.; Wen, Y.; Song, C.; Li, J. Electrochemical oxidation enables regioselective 1,3-Hydroxyfunctionalization of cyclopropanes. *Org. Lett.* **2023**, *25*, 6178–6183.

using CH_2Br_2 (14 μl, 0.20 mmol, 1.0 equiv) as internal standard

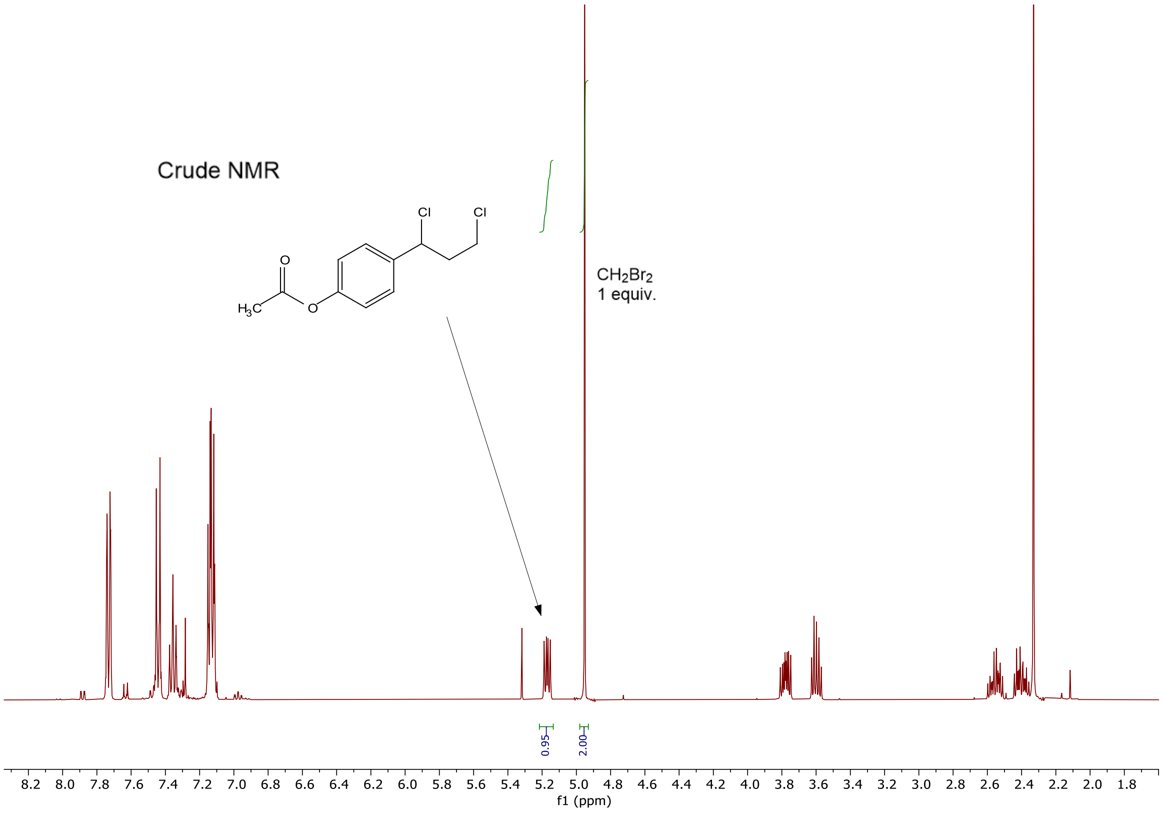

The mixture was purified by prepTLC (Pentane:Et_2O 8:1) to give product **9c** (36.5 mg, 0.148 mmol, 74 %) as colorless oil. **TLC** (Pentane/Et_2O = 8/1) Rf = 0.32. **^{1}H NMR** (400 MHz, $CDCl_3$) δ 7.46 – 7.37 (m, 2H, Ar*H*), 7.14 – 7.07 (m, 2H, Ar*H*), 5.14 (dd, *J* = 9.1, 5.3 Hz, 1H, ArClC*H*), 3.80 – 3.66 (m, 1H, CH_2ClCH_2), 3.61 – 3.51 (m, 1H, CH_2ClCH_2), 2.59 – 2.44 (m, 1H, $ClCH_2CH_2$), 2.44 – 2.34 (m, 1H, $ClCH_2CH_2$), 2.30 (s, 3H, $COCH_3$). **^{13}C NMR** (101 MHz, $CDCl_3$) δ 169.4, 150.8, 138.2, 128.3, 122.1, 59.4, 42.4, 41.9, 21.3. **HRMS** (Sicrit plasma/LTQ-Orbitrap) m/z: $[M + H]^+$ Calcd for $C_{11}H_{13}Cl_2O_2^+$ 247.0287; Found 247.0286.

1-Bromo-4-(1,3-dichloropropyl)benzene (9d)

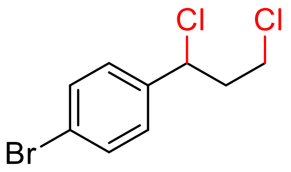

Following general procedure C, starting from 4-bromo-phenyl cyclopropane **8d** (39.4 mg, 0.200 mmol, 1.00 equiv). **6a** (82.0 mg, 0.300 mmol, 1.50 equiv). The mixture was purified by column

chromatography on Biotage (heptane 100%) to obtain the product **9d** (34.0 mg, 0.127 mmol, 63 %) as colorless oil.

TLC (Heptane/EtOAc 20/1) Rf = 0.50. **^{1}H NMR** (500 MHz, $CDCl_3$) δ 7.49–7.32 (m, 2H, ArC*H*), 7.23–7.15 (m, 2H, ArC*H*), 5.01 (dd, *J* = 9.0, 5.3 Hz, 1H, ArClC*H*), 3.71–3.62 (m, 1H, CH_2ClCH_2), 3.52–3.41 (m, 1H, CH_2ClCH_2), 2.5–2.36 (m, 1H, $ClCH_2CH_2$), 2.36–2.19 (m, 1H, $ClCH_2CH_2$), **^{13}C NMR** (126 MHz, $CDCl_3$) δ 139.7, 132.1, 128.8, 122.7, 59.2, 42.3, 41.7. NMR matched reported value.[6]

4-(1,3-Dichloropropyl)benzonitrile (9e)

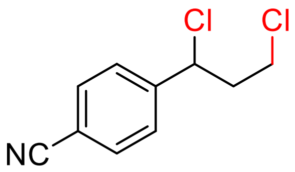

Following general procedure C, starting from cyclopropane **8e** (28.6 mg, 0.200 mmol, 1.00 equiv), **6a** (82.0 mg, 0.300 mmol, 1.50 equiv). The mixture was purified by column chromatography on Biotage (gradient heptane/EtOAc 100/0 to 80/20) to obtain the product **9e** (38.9 mg, 0.182 mmol, 91%) as colorless oil. **TLC** (Heptane/EtOAc 20/1) Rf = 0.22. **^{1}H NMR** (500 MHz, $CDCl_3$) δ 7.72–7.63 (m, 2H, Ar*H*), 7.59–7.41 (m, 2H, Ar*H*), 5.16 (dd, *J* = 9.3, 5.0 Hz, 1H, ArClC*H*), 3.85–3.72 (m, 1H, CH_2ClCH_2), 3.66–3.53 (m, 1H, CH_2ClCH_2), 2.6–2.43 (m, 1H, $ClCH_2CH_2$), 2.42–2.29 (m, 1H, $ClCH_2CH_2$). **^{13}C NMR** (126 MHz, $CDCl_3$) δ 145.7, 132.8, 128.0, 118.4, 112.7, 58.8, 42.2, 41.5. **HRMS** (APCI/QTOF) m/z: $[M + H]^+$ Calcd for $C_{10}H_{10}Cl_2N^+$ 214.0185; Found 214.0180.

1-(4-(1,3-Dichloropropyl)phenyl)ethan-1-one (9f)

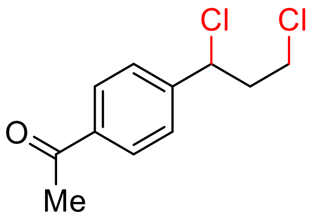

Following general procedure C, starting from cyclopropane **8f** (32.0 mg, 0.200 mmol, 1.00 equiv), **6a** (82.0 mg, 0.300 mmol, 1.50 equiv). The mixture was purified by column chromatography on

[6] Zyk, N. V.; Gavrilova, A. Yu.; Bondarenko, O. B.; Mukhina, O. A.; Tikhanushkina, V. N. Reactions of cyclopropanes with potassium dihaloiodates. *Russ. J. Org. Chem.* **2011**, *47*, 340–354.

Biotage (gradient heptane/EtOAc 100/0 to 80/20) to obtain the product **9f** (34.8 mg, 0.151 mmol, 75 %) as pale-yellow oil. **TLC** (Heptane/EtOAc 20/1) Rf = 0.17. **^{1}H NMR** (500 MHz, $CDCl_3$) δ 8.01-7.93 (m, 2H, Ar*H*), 7.55-7.46 (m, 2H, Ar*H*), 5.18 (dd, *J* = 9.1, 5.2 Hz, 1H, ArClC*H*), 3.8-3.73 (m, 1H, CH_2ClCH_2), 3.6–3.54 (m, 1H, CH_2ClCH_2), 2.61 (s, 3H, $COCH_3$), 2.59–2.49 (m, 1H, $ClCH_2CH_2$), 2.43–2.34 (m, 1H, $ClCH_2CH_2$). **^{13}C NMR** (126 MHz, $CDCl_3$) δ 197.5, 145.6, 137.4, 129.0, 127.4, 59.2, 42.2, 41.7, 26.8. **HRMS** (APCI/QTOF) m/z: $[M + H]^+$ Calcd for $C_{11}H_{13}Cl_2O^+$ 231.0338; Found 231.0345.

2-(4-(1,3-Dichloropropyl)phenyl)-4,4,5,5-tetramethyl-1,3,2-dioxaborolane (9g)

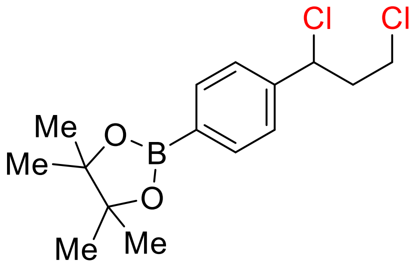

Following general procedure C, starting from cyclopropane **8g** (48.8 mg, 0.200 mmol, 1.00 equiv), **6a** (82.0 mg, 0.300 mmol, 1.50 equiv). The mixture was purified by column chromatography on Biotage (gradient heptane/EtOAc 100/0 to 80/20) to obtain the product **9g** (53.0 mg, 0.168 mmol, 84 %) as white solid. **TLC** (Heptane/EtOAc 20/1) Rf = 0.23. **^{1}H NMR** (500 MHz, $CDCl_3$) δ 7.82 (d, *J* = 7.8 Hz, 2H, Ar*H*), 7.41 (d, *J* = 7.9 Hz, 2H, Ar*H*), 5.14 (dd, *J* = 9.0, 5.3 Hz, 1H, ArClC*H*),3.82-3.68 (m, 1H, CH_2ClCH_2), 3.59– 3.49 (m, 1H, CH_2ClCH_2), 2.59–2.45 (m, 1H, $ClCH_2CH_2$), 2.43–2.36 (m, 1H, $ClCH_2CH_2$), 1.34 (s, 12H, $OCCH_3$). **^{13}C NMR** (126 MHz, $CDCl_3$) δ 143.5, 135.4, 126.4, 84.1, 60.0, 42.3, 41.9, 25.00, 24.99, 1 carbon is unresolved. **HRMS** (APCI/QTOF) m/z: $[M]^+$ Calcd for $C_{15}H_{21}BClO_2^+$ 279.1318; Found 279.1309.

2-(1,3-Dichloropropyl)naphthalene (9h)

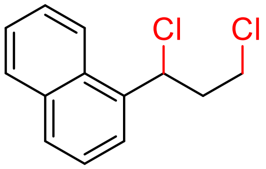

Following general procedure C, starting from cyclopropane **8h** (33.6 mg, 0.200 mmol, 1.00 equiv), **6a** (82.0 mg, 0.300 mmol, 1.50 equiv). The mixture was purified by column chromatography on

Biotage (heptane) to obtain the product **9h** (38.9 mg, 0.163 mmol, 81%) as yellow oil. TLC: (Heptane/EtOAc 20/1) Rf = 0.46. **^{1}H NMR** (500 MHz, $CDCl_3$) δ 8.19 (d, *J* = 8.5 Hz, 1H, Ar*H*), 7.90 (d, *J* = 8.1 Hz, 1H, Ar*H*), 7.85 (dd, *J* = 8.2, 1.1 Hz, 1H, Ar*H*), 7.72 (d, *J* = 7.2 Hz, 1H, Ar*H*), 7.64–7.58 (m, 1H, Ar*H*), 7.57–7.47 (m, 2H, Ar*H*), 6.02 (dd, *J* = 9.9, 4.1 Hz, 1H, ArClC*H*), 3.97–3.89 (m, 1H, CH_2ClCH_2), 3.81–3.71 (m, 1H, CH_2ClCH_2), 2.79–2.68 (m, 1H, $ClCH_2CH_2$), 2.68–2.58 (m, 1H, $ClCH_2CH_2$). **^{13}C NMR** (126 MHz, $CDCl_3$) δ 136.1, 134.0, 130.4, 129.5, 129.2, 126.9, 126.1, 125.5, 124.8, 122.9, 56.4, 42.4, 41.5. **HRMS** (LTQ-Orbitrap) m/z: $[M]^+$ Calcd for $C_{13}H_{12}Cl_2^+$ 238.0311; Found 238.0303.

1,3-Dichloro-1,3-diphenylpropane (9i)

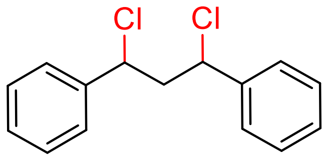

Following general procedure C, starting from cyclopropane **8i** (38.9 mg, 0.200 mmol, 1.00 equiv), **6a** (82.0 mg, 0.300 mmol, 1.50 equiv). The mixture was purified by column chromatography on Biotage (heptane) to obtain the product **9i** (42.0 mg, 0.158 mmol, 79 %) as white solid, a mixture of two diastereomer (integrating the peak of ArCH major 5.26–5.19ppm, minor 4.85–4.76 ppm) dr 5.5 : 4.5 was analysed in crude mixture with CH_2Br_2 as internal standard. **TLC** (Heptane/EtOAc 20/1) Rf = 0.50

Major. ^{1}H NMR (500 MHz, $CDCl_3$) δ, 7.44–7.31 (m, 10H, Ar*H*), 5.26–5.19 (m, 2H, C*H*Cl), 2.73–2.67 (m, 2H, C*H*$_2$). **Minor ^{1}H NMR** (500 MHz, $CDCl_3$) δ 7.44 7.31 (m, 10H, Ar*H*), 4.85–4.76 (m, 2H, C*H*Cl), 3.03–2.93 (m, 1H, C*H*$_2$), 2.75–2.69 (m, 1H, C*H*$_2$), **^{13}C NMR** (126 MHz, $CDCl_3$) δ (for both diastereomers) 140.9, 140.3, 129.03, 128.98, 128.9, 128.8, 127.22, 127.16, 60.9, 60.3, 49.8, 49.6. NMR data matched reported value.[7]

Dimethyl 2-chloro-2-(2-chloro-2-phenylethyl)malonate (9j)

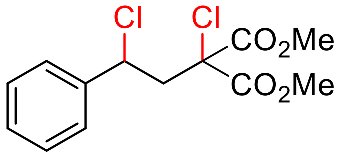

[7] Kabalka, G. W.; Wu, Z.; Ju, Y.; Yao, M.-L. Lewis Acid Mediated Reactions of Aldehydes with Styrene Derivatives: Synthesis of 1,3-Dihalo-1,3-diarylpropanes and 3-Chloro-1,3-diarylpropanols. *J. Org. Chem.* **2005**, *70*, 10285–10291.

Following general procedure C, starting from cyclopropane **8j** (46.8 mg, 0.200 mmol, 1.00 equiv), **6a** (82.0 mg, 0.300 mmol, 1.50 equiv). The mixture was purified by column chromatography on Biotage (heptane) to obtain the product **9j** (41.2 mg, 0.135 mmol, 68 %) as pale-yellow oil. **TLC**: (Heptane/Et_2O 6/1) Rf = 0.21. **^{11}H NMR** (500 MHz, $CDCl_3$) δ 7.48–7.28 (m, 5H, ArC*H*). 5.25 (dd, *J* = 7.7, 5.6 Hz, 1H, ArClC*H*), 3.84 (s, 3H, OC*H₃*), 3.55 (s, 3H, OC*H₃*), 3.22 (dd, *J* = 15.4, 7.8 Hz, 1H, CHClC*H₂*), 3.06 (dd, *J* = 15.4, 5.7 Hz, 1H, CHClC*H₂*). **^{13}C NMR** (126 MHz, $CDCl_3$) δ 166.7, 166.3, 140.3, 128.9, 128.8, 127.5, 68.6, 58.1, 54.2, 54.0, 46.7. **HRMS** (Sicrit plasma/LTQ-Orbitrap) m/z: $[M + H]^+$ Calcd for $C_{13}H_{15}Cl_2O_4^+$ 305.0342; Found 305.0341.

4-(1,3-Dichloropropyl)-2-methylbenzoic acid (9k)

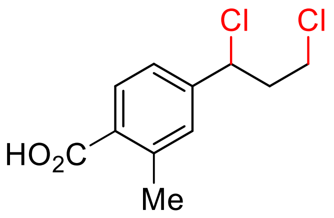

Following general procedure C, starting from cyclopropane **8k** (35.2 mg, 0.200 mmol, 1.00 equiv), **6a** (82.0 mg, 0.300 mmol, 1.50 equiv). The mixture was purified by reverse phase column chromatography on Biotage (gradient 5% to 95% of CH_3CN – water 0.1% formic acid) to obtain the product **9k** (45.2 mg, 0.183 mmol, 91 %) as white solid. TLC: (EtOAc/Heptane 2/3) Rf = 0.3. **^{1}H NMR** (500 MHz, CD_2Cl_2) δ 8.10 – 8.05 (m, 1H, Ar*H*), 7.38 – 7.33 (m, 2H, Ar*H*), 5.16 (dd, *J* = 9.1, 5.2 Hz, 1H, ArClC*H*), 3.83 – 3.71 (m, 1H, CH_2ClC*H₂*), 3.67 – 3.53 (m, 1H, CH_2ClC*H₂*), 2.67 (s, 3H, ArC*H₃*), 2.64 – 2.50 (m, 1H, ClCH_2C*H₂*), 2.48 – 2.37 (m, 1H, ClCH_2C*H₂*). **^{13}C NMR** (126 MHz, CD_2Cl_2) δ 172.8, 145.8, 142.5, 132.5, 131.0, 128.8, 124.9, 59.6, 42.3, 42.2, 22.3. **HRMS** (ESI/QTOF) m/z: $[M + H\text{-}1]^-$ Calcd for $C_{11}H_{12}Cl_2O_2$ 245.0142; Found 245.0132.

5-(1,3-Dichloropropyl)-2-methoxypyridine (9l)

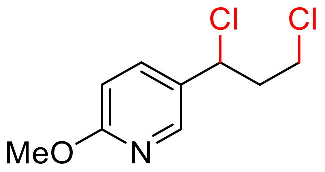

Following general procedure C, starting from cyclopropane **8l** (29.8 mg, 0.200 mmol, 1.00 equiv), **6a** (82.0 mg, 0.300 mmol, 1.50 equiv). The mixture was purified by column chromatography on Biotage (gradient pentane/Et_2O 100/0 to 75/25) to obtain the product **9l** (32.6 mg, 0.149 mmol, 74 %) as yellow oil. TLC (Et_2O/Pentane 1/8) Rf = 0.46. **^{1}H NMR** (400 MHz, $CDCl_3$) δ 8.16 (d, *J* =

2.6 Hz, 1H, Ar*H*), 7.63 (dd, *J* = 8.7, 2.6 Hz, 1H, Ar*H*), 6.77 (d, *J* = 8.6 Hz, 1H, Ar*H*), 5.13 (dd, *J* = 9.0, 5.4 Hz, 1H, ArClC*H*), 3.94 (s, 3H, OC*H₃*), 3.79 – 3.70 (m, 1H, CH_2ClCH_2), 3.63 – 3.50 (m, 1H, CH_2ClCH_2), 2.63 – 2.49 (m, 1H, $ClCH_2CH_2$), 2.41 – 2.30 (m, 1H, $ClCH_2CH_2$). **^{13}C NMR** (101 MHz, $CDCl_3$) δ 164.5, 145.6, 137.4, 129.2, 111.6, 57.4, 53.8, 42.0, 41.7. **HRMS** (ESI/QTOF) m/z: $[M + H]^+$ Calcd for $C_9H_{12}Cl_2NO^+$ 220.0290; Found 220.0293.

2-((5-Bromo-4-(4-(1,3-dichloropropyl)naphthalen-1-yl)-4H-1,2,4-triazol-3-yl)thio)acetic acid (9m)

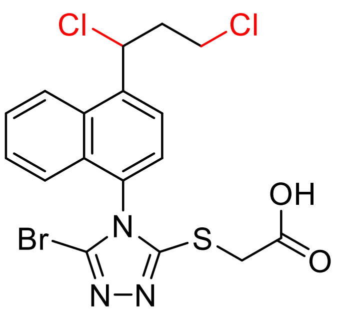

Following general procedure C, starting from cyclopropane **8m** (80.8 mg, 0.200 mmol, 1.00 equiv), **6a** (137 mg, 0.500 mmol, 2.50 equiv). The mixture was purified by reverse phase column chromatography on Biotage (gradient 5% to 95% of CH_3CN – water 0.1% formic acid) to obtain the product **9m** (40.2 mg, 0.0850 mmol, 42 %) as orange sticky oil. **TLC** (DCM/MeOH 3/1) Rf = 0.44. **^{1}H NMR** (500 MHz, $CDCl_3$) 8.30 (dd, *J* = 8.7, 3.8 Hz, 1H, Ar*H*). δ 8.06–7.90 (m, 1H, O*H*), 7.88 (dd, *J* = 7.8, 5.5 Hz, 1H, Ar*H*), 7.74–7.66 (m, 1H, Ar*H*), 7.62 (t, *J* = 7.7 Hz, 1H, Ar*H*), 7.52 (d, *J* = 7.7 Hz, 1H, Ar*H*), 7.28 (d, *J* = 8.5 Hz, 1H, Ar*H*), 6.12 – 5.98 (m, 1H, ArC*H*Cl), 4.11–3.91 (m, 3H, SCH_2 and CH_2ClCH_2), 3.81 (dt, *J* = 11.2, 4.7 Hz, 1H, CH_2ClCH_2), 2.75–2.53 (m, 2H, $ClCH_2CH_2$), **^{13}C NMR** (126 MHz, $CDCl_3$) δ 169.9, 154.7, 140.4, 131.4, 131.2, 129.4, 128.8, 128.7, 128.4, 126.3, 124.4, 123.7, 122.5, 55.4, 42.1, 41.4, 34.5. **HRMS** (ESI/QTOF) m/z: $[M + H\text{-}1]^-$ Calcd for $C_{17}H_{14}BrCl_2N_3O_2S$ 471.9294, found 471.9296

1-(2-Chloro-4-(1,3-dichloropropyl)phenyl)-5-methyl-1H-1,2,3-triazole-4-carboxylic acid (9n)

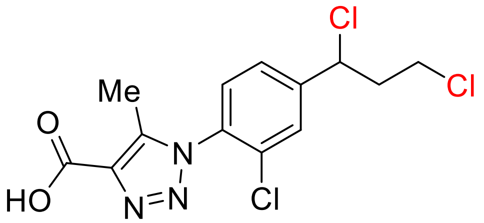

Following general procedure C, starting from cyclopropane **8n** (56.0 g, 0.200 mmol, 1.00 equiv), **6a** (219 mg, 0.80 mmol, 4.00 equiv.). The mixture was purified by reverse phase column chromatography Biotage (gradient 5% to 95% of CH_3CN – water 0.1% formic acid) to obtain the product **9n** (40.8 mg, 0.118 mmol, 59 %) as yellow solid. **TLC** (DCM/MeOH 5/1) Rf = 0.30. **^{1}H NMR** (500 MHz, d6-Acetone) δ 7.76 (d, *J* = 8.2 Hz, 1H, Ar*H*), 7.96 (d, *J* = 2.0 Hz, 1H, Ar*H*), 7.82 (dd, *J* = 8.2, 2.0 Hz, 1H, Ar*H*), 5.43 (dd, *J* = 9.2, 5.2 Hz, 1H, ArClC*H*), 3.91–3.83 (m, 1H, CH_2ClCH_2), 3.82–3.75 (m, 1H, CH_2ClCH_2), 2.75–2.65 (m, 1H, $ClCH_2CH_2$), 2.65–2.56 (m, 1H, $ClCH_2CH_2$), 2.44 (s, 3H, ArC*H*$_3$). **^{13}C NMR** (126 MHz, d6-Acetone) δ 162.7, 146.6, 141.7, 136.8, 134.1, 132.4, 131.0, 130.2, 128.3, 59.4, 42.4 (2 carbons), 9.5. **HRMS** (ESI/QTOF) m/z: [M + H-1]$^-$ Calcd for $C_{13}H_{12}C_{l3}N_3O_2$ 345.9922, found 345.9911

2-(1,2-Dichloroethyl)naphthalene (11a)

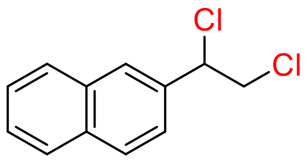

Following general procedure C, starting from olefin **10a** (30.8 mg, 0.200 mmol, 1.00 equiv), **6a** (82.0 mg, 0.300 mmol, 1.5 equiv). The mixture was purified by column chromatography on Biotage (pentane 100%) to obtain the product **11a (**26.8 mg, 0.119 mmol, 60 %) as brown oil. **TLC** (EtOAc/Heptane 1/20) Rf = 0.46. **^{1}H NMR** (500 MHz, $CDCl_3$) δ 7.91–7.85 (m, 4H, Ar*H*). 7.55–7.51 (m, 3H, Ar*H*), 5.18 (dd, *J* = 8.1, 6.5 Hz, 1H, ArC*H*Cl), 4.14–3.97 (m, 2H, C*H*$_2$Cl). **^{13}C NMR** (126 MHz, $CDCl_3$) δ 135.3, 133.7, 133.1, 129.1, 128.3, 127.9, 127.4, 127.0, 126.8, 124.3, 62.2, 48.3. NMR data matched reported value.[8]

1-(1,2-Dichloroethyl)-4-methoxybenzene (11b)

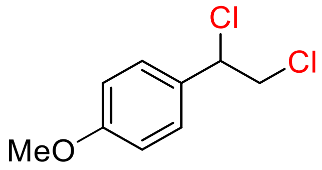

[8] Lian, P.; Long, W.; Li, J.; Zheng, Y.; Wan, X. Visible-Light-Induced Vicinal Dichlorination of Alkenes through LMCT Excitation of $CuCl_2$. *Angew. Chem. Int. Ed.* **2020,** *59*, 23603–23608.

Following general procedure C, starting from olefin **10b** (0.027 g, 0.200 mmol, 1.00 equiv), **6a** (110 mg, 0.400 mmol, 2.00 equiv). The mixture was purified by column chromatography on Biotage (gradient Heptane/EtOAc 100/0 – 90/10) to obtain the product **11b (**21.1 mg, 0.103 mmol, 51%) as colorless oil. **TLC**: (EtOAc/Heptane 1/20) Rf = 0.29. ^{1}**H NMR** (500 MHz, $CDCl_3$) δ, 7.39–7.29 (m, 2H, Ar*H*), 6.97–6.86 (m, 2H, Ar*H*), 4.98 (dd, *J* = 8.2, 6.5 Hz, 1H, ArC*H*Cl), 3.99 (dd, *J* = 11.3, 6.5 Hz, 1H, CH_2Cl), 3.91 (dd, *J* = 11.3, 8.2 Hz, 1H, CH_2Cl), 3.82 (s, 3H, OCH_3). ^{13}**C NMR** (126 MHz, $CDCl_3$) δ 160.3, 130.2, 128.8, 114.3,, 61.8, 55.5, 48.5. NMR data matched reported.[9]

1-(1,2-Dichloroethyl)-4-(trifluoromethyl)benzene (11c)

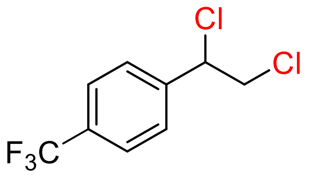

Following general procedure C, starting from olefin **10c** (34.4 mg, 0.200 mmol, 1.00 equiv), **6a** (110 mg, 0.400 mmol, 2.00 equiv). The mixture was purified by column chromatography on Biotage (Heptane) to obtain product **11c** (42.7 mg, 0.176 mmol, 88 %) as colorless oil. **TLC** (Heptane) Rf = 0.38. ^{1}**H NMR** (500 MHz, $CDCl_3$) δ 7.67 (d, *J* = 8.1 Hz, 2H, Ar*H*), 7.55 (d, *J* = 8.1 Hz, 2H, Ar*H*), 5.04 (dd, *J* = 8.5, 6.0 Hz, 1H, ArC*H*Cl), 4.02 (dd, *J* = 11.4, 6.0 Hz, 1H, CH_2Cl), 3.91 (dd, *J* = 11.4, 8.5 Hz, 1H, CH_2Cl). ^{13}**C NMR** (126 MHz, $CDCl_3$) δ 141.9, 131.4 (q, *J* = 32.6 Hz), 128.1, 126.0 (q, *J* = 3.8 Hz). 123.91 (q, J = 272.3 Hz), 60.5, 48.0. ^{19}**F NMR** (470 MHz, $CDCl_3$) δ -62.8. NMR data matches reported value.[10]

Methyl 4-(1,2-dichloroethyl)benzoate (11d)

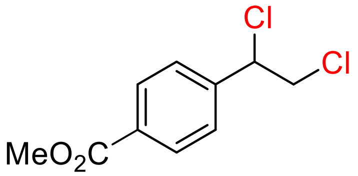

Following general procedure C, starting from olefin **10d** (32.4 mg, 0.200 mmol, 1.00 equiv), **6a** (110 mg, 0.400 mmol, 2.00 equiv). The mixture was purified by column chromatography on Biotage (gradient Heptane/EtOAc 100/0 – 80/20) to obtain product **11d** (36.6 mg, 0.157 mmol, 79

---

[9] Egami, H.; Yoneda, T.; Uku, M.; Ide, T.; Kawato, Y.; Hamashima, Y. Difunctionalization of alkenes using 1-Chloro-1,2-benziodoxol-3-(1H)-one. *J. Org. Chem.* **2016**, *81*, 4020–4030.

[10] Dong, X.; Roeckl, J. L.; Waldvogel, S. R.; Morandi, B. Merging shuttle reactions and paired electrolysis for reversible vicinal dihalogenations. *Science* **2021**, *371*, 507–514.

%) as colorless oil. **TLC** (Et_2O/Heptane 1/10) Rf = 0.27. **^{1}H NMR** (500 MHz, $CDCl_3$) δ 8.12–8.00 (m, 2H, ArC*H*). 7.56–7.42 (m, 2H, ArC*H*), 5.03 (dd, *J* = 8.3, 6.2 Hz, 1H, ArC*H*Cl), 4.00 (dd, *J* = 11.4, 6.2 Hz, 1H, CH_2Cl), 3.92 (s, 3H, OCH_3), 3.92–3.89 (m, 1H, CH_2Cl), **^{13}C NMR** (126 MHz, $CDCl_3$) δ 166.5, 142.8, 131.0, 130.2, 127.7, 60.8, 52.4, 48.1. NMR data matching reported value.[11]

(1,2-Dichloropropan-2-yl)benzene (11e)

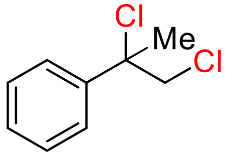

Following general procedure C, starting from olefin **10e** (23.6 mg, 0.200 mmol, 1.00 equiv), **6a** (110 mg, 0.400 mmol, 2.00 equiv). The mixture was purified by column chromatography on Biotage (Heptane) to obtain the product **11e** (35.1 mg, 0.186 mmol, 93%) as a colorless oil. **TLC** (heptane) Rf = 0.4. **^{1}H NMR** (500 MHz, $CDCl_3$) δ 7.58–7.54 (m, 2H, Ar*H*). 7.42–7.37 (m, 2H, Ar*H*), 7.36–7.31 (m, 1H, Ar*H*), 4.06 (d, *J* = 11.3 Hz, 1H, CH_2Cl), 4.00 (d, *J* = 11.2 Hz, 1H, CH_2Cl), 2.10 (s, 3H, CH_3). **^{13}C NMR** (126 MHz, $CDCl_3$) δ 141.6, 128.6, 128.5, 126.6, 70.7, 54.7, 28.4. NMR data matches reported value.[12]

(1,2-Dichloropropyl)benzene (11f)

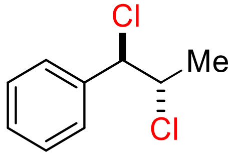

Following general procedure C, starting from *trans* olefin **10f** (23.6 mg, 0.200 mmol, 1.00 equiv), **6a** (110 mg, 0.400 mmol, 2.00 equiv). The mixture was purified by column chromatography on Biotage (Heptane) to obtain the product **11f** (26.8 mg, 0.142 mmol, 71%) as colorless oil, a mixture of two diastereomer dr 1:6.5 was analysed by crude mixture with signal of CH_3 Major: 1.72 ppm, minor 1.46 ppm. **TLC** heptane Rf = 0.33

[11] Li, M.; Qiu, M.; Ren, Y.; Li, H.; An, W. Light-Induced vicinal dichlorination of alkenes using FECL3 as the dichlorination reagent. *ChemistrySelect* **2024**, *9*.
[12] Fu, N.; Sauer, G. S.; Lin, S. Electrocatalytic Radical Dichlorination of Alkenes with Nucleophilic Chlorine Sources. *J. Am. Chem.* Soc. **2017**, *139*, 15548–15553.

Major. ^{1}**H NMR** (500 MHz, $CDCl_3$) δ 7.46–7.38 (m, 3H, Ar*H*). 7.38–7.33 (m, 2H, Ar*H*), 4.92 (d, *J* = 7.8 Hz, 1H, ArC*H*Cl), 4.45–4.35 (m, 1H, CH_3C*H*Cl), 1.72 (d, *J* = 6.5 Hz, 3H, C*H₃*). **Minor** ^{1}**H NMR** (500 MHz, $CDCl_3$) δ 7.46–7.38 (m, 3H, Ar*H*). 7.38–7.33 (m, 2H, Ar*H*), 5.00 (d, *J* = 6.0 Hz, 1H), 4.43 – 4.41 (m, 1H), 1.47 (d, *J* = 6.7 Hz, 3H). ^{13}**C NMR** (126 MHz, $CDCl_3$) for two diastereomers δ 138.8, 137.5, 129.0, 128.9, 128.7, 128.5, 128.2, 127.9, 67.6 (2 carbons), 61.3, 60.3, 22.3, 21.8. NMR data matches reported value.[12]

Methyl-2,3-dichloro-3-phenylpropanoate (11g)

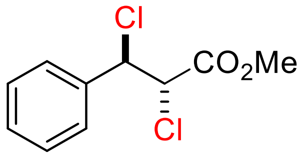

Following general procedure C, starting from olefin *trans* **10g** (32.4 mg, 0.200 mmol, 1.00 equiv), **6a** (110 mg, 0.400 mmol, 2.00 equiv). dr 1:7.2 was analysed by crude mixture with signal of OC*H₃* Major: 3.90 ppm, minor 3.62 ppm. The mixture was purified by column chromatography on Biotage (gradient Heptane/EtOAc 100/0 – 80/20) to obtain the product **11g**

Major (32.5 mg, 0.139 mmol, 70 %) as pale-yellow oil**. TLC** (Et_2O/Heptane 1/20) Rf = 0.27. ^{1}**H NMR** (500 MHz, $CDCl_3$) δ 7.47–7.34 (m, 5H, Ar*H*). 5.18 (d, *J* = 10.7 Hz, 1H, ArC*H*Cl), 4.62 (d, *J* = 10.7 Hz, 1H, C*H*Cl), 3.90 (s, 3H, OC*H₃*). ^{13}**C NMR** (126 MHz, $CDCl_3$) δ 168.1, 136.5, 129.6, 129.0, 128.2, 61.2, 59.0, 53.5.

Minor (4.60 mg, 0.0197 mmol, 10 %) as pale-yellow oil. **TLC** (EtOAc/Heptane 1/20) Rf = 0.46. ^{1}**H NMR** (500 MHz, $CDCl_3$) δ 7.42 (dd, *J* = 7.6, 2.2 Hz, 2H, Ar*H*). 7.40–7.33 (m, 3H, Ar*H*), 5.31 (d, *J* = 8.1 Hz, 1H, ArC*H*Cl), 4.66 (d, *J* = 8.0 Hz, 1H, C*H*Cl), 3.62 (s, 3H, OC*H₃*) ^{13}**C NMR** (126 MHz, $CDCl_3$) δ 167.2, 136.7, 129.6, 128.9, 127.9, 63.7, 62.7, 53.3.

NMR data matches reported value.[13]

(2,3-Dichloropropyl)benzene (11h1)

---

[13] Bian, K.-J.; Nemoto, D.; Chen, X.-W.; Kao, S.-C.; Hooson, J.; West, J. G. Photocatalytic, modular difunctionalization of alkenes enabled by ligand-to-metal charge transfer and radical ligand transfer. *Chem. Sci.* **2024**, *15*, 124–133.

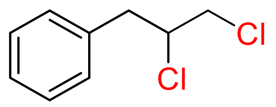

(1,3-Dichloropropan-2-yl)benzene (11h2)

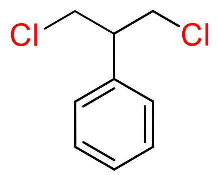

Following general procedure C, starting from olefin **10h** (23.6 mg, 0.200 mmol, 1.00 equiv), **6a** (110 mg, 0.400 mmol, 2.00 equiv). ratio 5:1 was analysed by crude mixture with signal of 4.27 ppm C*H*Cl **11h1** and 3.92 ppm C*H*$_2$Cl – 2H of minor **11h2**. The mixture was purified by column chromatography on Biotage (Heptane) to obtain the mixture of **11h1** and **11h2** (26.8 mg, 0.142 mmol, 71 %) as a colorless oil. TLC (Et_2O/Heptane 1/20) Rf = 0.57.

(11h1) ^{1}**H NMR** (500 MHz, $CDCl_3$,) 7.38–7.24 (m, 5H, Ar*H*). 4.3–4.24 (m, 1H, C*H*Cl), 3.73 (dd, *J* = 11.4, 4.8 Hz, 1H, ClC*H*$_2$), 3.66 (dd, *J* = 11.4, 6.8 Hz, 1H, ClC*H*$_2$), 3.31 (dd, *J* = 14.2, 5.7 Hz, 1H, ArC*H*), δ 3.09–3.05 (m, 1H, ArC*H*). ^{13}**C NMR** (126 MHz, $CDCl_3$) δ 136.4, 129.7, 128.7, 127.3, 61.1, 47.6, 41.2.

(11h2) ^{1}**H NMR** (500 MHz, $CDCl_3$,) δ 7.38–7.24 (m, 5H), 3.92 (dd, *J* = 11.1, 6.8 Hz, 2 H, C*H*$_2$Cl), 3.87 (dd, *J* = 11.1, 6.0 Hz, 2 H, C*H*$_2$Cl), 3.37 - 3.30 (m, 1 H, ArC*H*). ^{13}**C NMR** (126 MHz, $CDCl_3$) δ 138.9, 128.9, 128.0, 128.0, 49.5, 46.0

Data matches reported value.[14]

(3,4-Dichlorobutyl)benzene (11i)

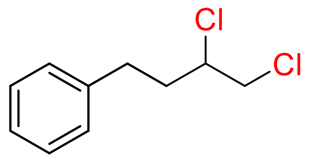

Following general procedure C, starting from olefin **10i** (26.4 mg, 0.200 mmol, 1.00 equiv), **6a** (110 mg, 0.400 mmol, 2.00 equiv). The mixture was purified by column chromatography on

[14] Ding, R.; Huang, S.; Wang, Q.; Liu, Y.; Sun, B.; Tian, H. Dichlorination of olefins with diphenyl sulfoxide/oxalyl chloride. *Synth. Commun.* **2020**, *50*, 2319–2330.

Biotage (Heptane) to obtain the product **11i** (32.1 mg, 0.158 mmol, 79%) as a colorless oil. **TLC** (Et_2O/Heptane = 1/20) Rf = 0.60. **^{1}H NMR** (500 MHz, $CDCl_3$) δ 7.37–7.29 (m, 2H, Ar*H*). 7.25–7.19 (m, 3H, Ar*H*), 4.07–3.93 (m, 1H, CH_2ClCH), 3.78 (dd, *J* = 11.4, 5.1 Hz, 1H, $ClCH_2$), 3.67 (dd, *J* = 11.3, 7.4 Hz, 1H, $ClCH_2$), 3.00–2.86 (m, 1H, $ArCH_2$), 2.86–2.71 (m, 1H, $ArCH_2$), 2.36–2.27 (m, 1H, $ArCH_2CH_2$), 2.08–1.96 (m, 1H, $ArCH_2CH_2$). **^{13}C NMR** (126 MHz, $CDCl_3$) δ 140.5, 128.73, 128.66, 126.4, 60.4, 48.4, 36.8, 32.2. NMR data matches reported value.[15]

1-(2-(2,3-Dichloropropoxy)phenoxy)-3-(isopropylamino)propan-2-ol (11j)

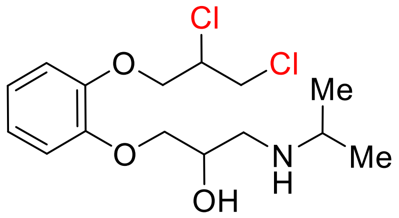

Following general procedure C, starting from Oxoprenolol.HCl **10j** (60.3 mg, 0.200 mmol, 1.00 equiv), **6a** (137 mg, 0.500 mmol, 2.50 equiv.). The mixture was purified by reverse phase column chromatography Biotage (gradient 5% to 95% of CH_3CN – water 0.1% formic acid) to obtain the product **11j** (49.8 mg, 0.148 mmol, 74 %) as pale yellow sticky solid dr: 1.1:1.0 (the ratio was determined by the signal of CH_3 1.40 ppm for minor diastereomer and 1.39 ppm for major diastereomer);. **TLC** (DCM/MeOH 10/1) Rf = 0.23**.** **^{1}H NMR** (500 MHz, $CDCl_3$) δ 8.56 (s, 1H, Ar*H*). 6.97–6.83 (m, 4H, Ar*H*), 4.56–4.46 (m, 1H CH_2O,), 4.44–4.36 (m, 1H, CH_2CHCl), 4.33–4.21 (m, 2H, 2x$ArCH_2O$), 4.15–4.06 (m, 1H, CH_2Cl), 4.03–3.90 (m, 3H, CH_2Cl, CH_2O, $CHOH$), 3.39–3.32 (m, 1H, $NHCH_2$), 3.32–3.24 (m, 1H, $NHCH_2$), 3.17–3.02 (m, 1H, $CHCH_3$), 1.40 (d, *J* = 7.3 Hz, 3H, CH_3), 1.39 (d, *J* = 6.6 Hz, 3H, CH_3). **^{13}C NMR** (126 MHz, $CDCl_3$) for both diastereomers δ 148.7, 148.1, 122.8, 122.2, 115.5, 114.8 (the aromatic signal for the other diastereomer is unresolved) 71.16, 71.14, 69.96, 69.92, 65.6(+1 carbon unresolved), 57.8(+1 carbon unresolved), 51.0 (+1 carbon unresolved), 48.27, 48.23, 45.29, 45.27 19.3, 19.0. **HRMS** (APCI/QTOF) m/z: $[M + H]^+$ Calcd for $C_{15}H_{24}Cl_2NO_3^+$ 336.1128; Found 336.1124.

Unsuccessful substrates

[15] Lubaev, A. E.; Rathnayake, M. D.; Eze, F.; Bayeh-Romero, L. Catalytic chemo-, regio-, diastereo-, and enantioselective bromochlorination of unsaturated systems enabled by Lewis Base-Controlled Chloride Release. *J. Am. Chem. Soc.* **2022**, *144*, 13294–13301.

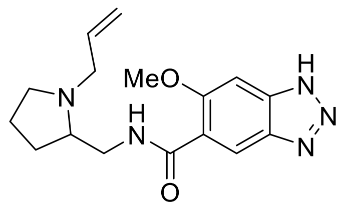

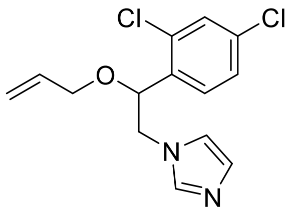

1-Bromo-4-(1-chloroethyl)benzene (13a)

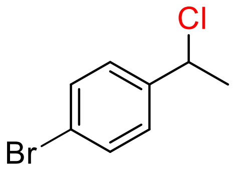

Following general procedure C, starting from **12a** (36.9 mg, 0.200 mmol, 1.00 equiv), **6a** (82.0 mg, 0.300 mmol, 1.50 equiv). The mixture was purified by column chromatography on PrepTLC (Heptane) to obtain the product **13a** (33.8 mg, 0.154 mmol, 77%) as a pale-yellow oil. TLC (Heptane) Rf = 0.38**.** ^{1}**H NMR** (500 MHz, $CDCl_3$) δ 7.47–7.38 (m, 2H, Ar*H*). 7.28–7.22 (m, 2H, Ar*H*), 5.00 (q, *J* = 6.8 Hz, 1H, ArC*H*Cl), 1.78 (d, *J* = 6.8 Hz, 3H, C*H*₃).^{13}**C NMR** (126 MHz, $CDCl_3$) δ 142.0, 131.9, 128.4, 122.2, 57.9, 26.6. NMR data matches reported value.[16]

(1-Chloroethyl)benzene (13b)

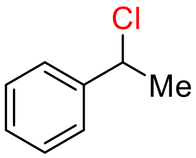

Following general procedure C, starting from **12a** (21.2 mg, 0.200 mmol, 1.00 equiv), **6a** (82.0 mg, 0.300 mmol, 1.50 equiv). The mixture was purified by column chromatography on Biotage (Heptane) to obtain the product **13b** (24.3 mg, 0.173 mmol, 86 %) as a colorless oil. TLC (Heptane) Rf = 0.43. ^{1}**H NMR** (500 MHz, $CDCl_3$) δ 7.33–7.29 (m, 1H, Ar*H*), 7.44–7.41 (m, 2H, Ar*H*). 7.39–7.34 (m, 2H, Ar*H*), 5.10 (q, *J* = 6.8 Hz, 1H, ArC*H*Cl), 1.86 (d, *J* = 6.8 Hz, 3H, CH_3). ^{13}**C NMR** (126 MHz, $CDCl_3$) δ 143.0, 128.8, 128.4, 126.6, 58.9, 26.7. NMR data matches reported value.[17]

[16] Ramachandran, P. V.; Alawaed, A. A.; Hamann, H. J. Balancing Lewis acidity and carbocation stability for the selective deoxyhalogenation of aryl carbonyls and carbinols. *Org. Lett.* **2023**, *25*, 4650–4655.
[17] Olivier, A.; Müller, D. S. Hydrochlorination of Alkenes with Hydrochloric Acid. *Org. Process Res. Dev.* **2023**, *28*, 305–309.

1-(4-Bromophenyl)cyclobutan-1-ol (13c)

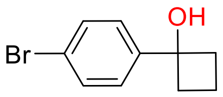

Following general procedure C, starting from cyclobutane **12c** (42.2 mg, 0.200 mmol, 1.00 equiv), **6a** (137 mg, 0.500 mmol, 2.50 equiv.). NMR yield 53% was analysed after 15 minutes using CH_2Br_2 (14 µl, 0.20 mmol, 1 equiv) as internal standard, integrating CH_2 peak (1.88 ppm).

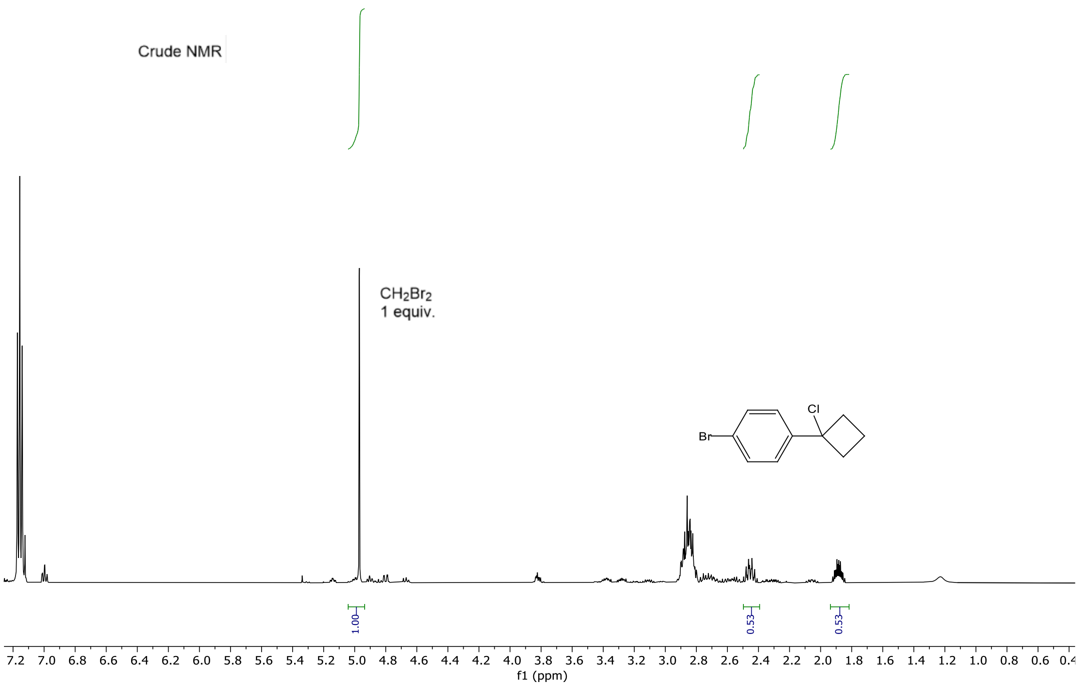

The crude mixture was purified by reverse phase column chromatography Biotage (gradient 5% to 95% of CH_3CN – water 0.1% formic acid) to obtain the product alcohol **13c** (17 mg, 0.0750 mmol, 37 %) as a colorless oil. TLC: (EtOAc/Heptane 1/20) Rf = 0.49. **^{1}H NMR** (500 MHz, $CDCl_3$) δ 7.52–7.44 (m, 2H, Ar*H*). 7.42–7.34 (m, 2H, Ar*H*), 2.59–2.47 (m, 2H, CClCH_2), 2.42–2.30 (m, 2H, CClCH_2), 2.09–1.99 (m, 1H, CClCH_2CH_2), 1.89 (s, 1H, O*H*), 1.74–1.62 (m, 1H, CClCH_2CH_2). **^{13}C NMR** (126 MHz, $CDCl_3$) δ 145.4, 131.6, 127.0, 121.3, 76.8, 37.2, 13.1. **HRMS** (APPI/LTQ-Orbitrap) m/z: $[M]^+$ Calcd for $C_{10}H_{10}Br^+$ 208.9960; Found 208.9966.

2-Chloro-1-phenylbutan-1-one (13d)

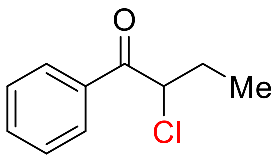

Following general procedure C, starting from **12d (**29.6 mg, 0.200 mmol, 1.00 equiv), **6a** (137 mg, 0.500 mmol, 2.50 equiv.). The mixture was purified by column chromatography on Biotage (Heptane) to obtain the product **13d** (33.9 mg, 0.186 mmol, 93%) as light-yellow oil. **TLC** (Et_2O/Heptane 1/20) Rf = 0.33. **^{1}H NMR** (500 MHz, $CDCl_3$) δ 8.04–7.99 (m, 2H, Ar*H*). 7.66–7.58 (m, 1H, Ar*H*), 7.53–7.47 (m, 2H, Ar*H*), 5.06 (t, *J* = 5.7 Hz, 1H, COC*H*), 2.29–2.10 (m, 1H, C*H₃*), 2.09–1.97 (m, 1H, C*H₂*), 1.09 (t, *J* = 7.4 Hz, 3H, C*H₃*). **^{13}C NMR** (126 MHz, $CDCl_3$) δ 193.8, 134.7, 133.8, 129.0, 128.9, 59.5, 27.2, 11.0. NMR data matches reported value.[18]

2-Chloro-2,3-dihydro-1H-inden-1-one (13e)

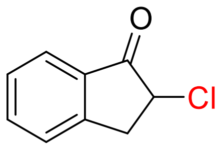

Following general procedure C, starting from **12e** (26.4 mg, 0.200 mmol, 1.00 equiv), **6a** (137 mg, 0.500 mmol, 2.50 equiv.). The mixture was purified by column chromatography on Biotage (gradient Heptane/EtOAc 100/0 to 80/20) to obtain the product **13e** (16.9 mg, 0.101 mmol, 51%) as colorless sticky oil. **TLC** (EtOAc /Heptane 1/10) Rf = 0.21. **^{1}H NMR** (500 MHz, $CDCl_3$) δ 7.47–7.44 (m, 2H, Ar*H*). 7.68–7.62 (m, 1H, Ar*H*), 7.48–7.40 (m, 1H, Ar*H*), 4.56 (dd, *J* = 7.9, 3.9 Hz, 1H, ClC*H*), 3.78 (dd, *J* = 17.5, 7.8 Hz, 1H, ArCH_2), 3.30 (dd, *J* = 17.5, 4.0 Hz, 1H, ArCH_2). **^{13}C NMR** (126 MHz, $CDCl_3$) δ 199.4, 150.9, 136.2, 134.0, 128.5, 126.5, 125.2, 55.9, 37.7. NMR data matches reported value.[19]

1-(6-(*Tert*-butyl)-3-chloro-1,1-dimethyl-2,3-dihydro-1H-inden-4-yl)ethan-1-one (13f)

[18] Tang, S.; Zhao, W.; Chen, T.; Liu, Y.; Zhang, X.; Zhang, F. A Simple and Efficient Method for the Preparation of α-Halogenated Ketones Using Iron(III) Chloride and Iron(III) Bromide as Halogen Sources with Phenyliodonium Diacetate as Oxidant. *Adv. Synth. Catal.* **2017**, *359*, 4177–4183.
[19] Xiang, J.-C.; Wang, J.-W.; Yuan, P.; Ma, J.-T.; Wu, A.-X.; Liao, Z.-X. Switching Over of the Chemoselectivity: I2-DMSO-Enabled α,α-Dichlorination of Functionalized Methyl Ketones. *J. Org. Chem.* **2022**, *87*, 15101–15113.

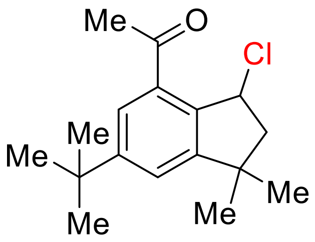

Following general procedure C, starting from **12g** (48.9 mg, 0.200 mmol, 1.00 equiv), **6a** (137 mg, 0.500 mmol, 2.50 equiv.). The mixture was purified by column chromatography on Biotage (gradient Heptane/EtOAc 100/0 to 80/20) to obtain the product **13f** (35.6 mg, 0.128 mmol, 64 %) as colorless oil. **TLC** (EtOAc /Heptane 1/20) Rf = 0.22. **^{1}H NMR** (500 MHz, $CDCl_3$) δ 7.73 (d, *J* = 1.8 Hz, 1H, ArC*H*), 7.37 (d, *J* = 1.8 Hz, 1H, ArC*H*), 6.04 (dd, *J* = 6.9, 1.4 Hz, 1H, ArC*H*Cl), 2.64 (s, 3H, COCH_3), 2.45 (dd, *J* = 14.6, 6.8 Hz, 1H, CHClCH_2), 2.39 (dd, *J* = 14.6, 1.4 Hz, 1H, CHClCH_2), 1.45 (s, 3H, ArCCH_3), 1.37 (s, 9H, C(CH_3)$_3$), 1.34 (s, 3H, ArCCH_3). **^{13}C NMR** (126 MHz, $CDCl_3$) δ 199.9, 154.3, 153.5, 138.7, 133.7, 126.1, 123.7, 60.4, 51.2, 43.2, 35.2, 31.5, 30.7, 29.4, 28.6. NMR data matches reported value.[20]

(2-(1-Chloroethyl)benzofuran-3-yl)(3,5-dibromo-4-hydroxyphenyl)methanone (13g)

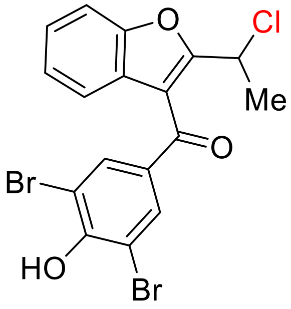

Following general procedure C, starting from **12i** (84.8 mg, 0.200 mmol, 1.00 equiv), **6a** (137 mg, 0.500 mmol, 2.50 equiv.). The mixture was purified by reverse phase column chromatography Biotage (gradient 5% to 95% of CH_3CN – water 0.1% formic acid to obtain the product **13g** (39.1 mg, 0.0850 mmol, 43 %) as white solid. **TLC** (EtOAc/ heptane 1/3) Rf = 0.30. **^{1}H NMR** (500 MHz, $CDCl_3$) δ 8.05 (s, 2H, Ar*H*), 7.45–7.40 (m, 2H, Ar*H*), 7.61–7.58 (m, 1H, Ar*H*), 7.32–7.27 (m, 1H, Ar*H*), 6.41 (s, 1H, O*H*), 5.36 (q, *J* = 6.8 Hz, 1H, ArC*H*Cl), 1.96 (d, *J* = 6.8 Hz, 3H, CH_3). **^{13}C NMR** (126 MHz, $CDCl_3$) δ 187.1, 160.0, 154.1, 153.9, 133.8, 133.0, 126.3, 125.8, 124.5, 121.8, 116.2, 112.0, 110.4, 48.8, 23.4. **HRMS** (APCI/QTOF) m/z: [M + Na]$^+$ Calcd for $C_{17}H_{11}Br_2ClNaO_3^+$ 478.8656; Found 478.8650.

[20] Fawcett, A.; Keller, M. J.; Herrera, Z.; Hartwig, J. F. Site Selective Chlorination of C(sp3)−H Bonds Suitable for Late-Stage Functionalization. *Angew. Chem. Int. Ed.* **2021**, *60*, 8276–8283.

2-Chloro-3,4-dihydronaphthalen-1(2H)-one (13h)

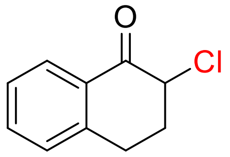

Following general procedure C, starting from **12f** (29.2 mg, 0.200 mmol, 1.00 equiv), **6a** (137 mg, 0.500 mmol, 2.50 equiv.). The mixture was purified by column chromatography on Biotage (gradient Heptane/EtOAc 100/0 to 80/20) to obtain the product **13h** (19.3 mg, the product contained impurities that could not be completely removed; therefore, the isolated yield is not reported) as yellow sticky oil. **TLC** (EtOAc /Heptane 1/10) Rf = 0.28. **^{1}H NMR** (500 MHz, $CDCl_3$) δ 8.05–8.12 (m, 1H, Ar*H*). 7.56–7.50 (m, 1H, Ar*H*), 7.39–7.32 (m, 1H, Ar*H*), 7.30–7.26 (m, 1H, Ar*H*), 4.64 (dd, *J* = 7.7, 3.9 Hz, 1H, ArCOClCH_2), 3.35–3.24 (m, 1H, ArCH_2), 3.04–2.95 (m, 1H, ArCH_2), 2.62–2.51 (m, 1H, CHClCH_2), 2.51–2.39 (m, 1H, CHClCH_2). **^{13}C NMR** (126 MHz, $CDCl_3$) δ 191.0, 143.3, 134.3, 130.6, 128.9, 128.7, 127.3, 59.9, 32.6, 26.4. NMR data matches reported value.[19]

Methyl 2-(4-(1-chloro-2-methylpropyl)phenyl)propanoate (13i)

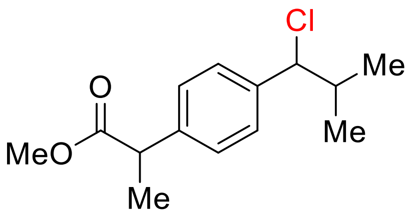

Following general procedure C, starting from **12h** (44.0 mg, 0.200 mmol, 1.00 equiv), **6a** (137 mg, 0.500 mmol, 2.50 equiv.). The mixture was purified by column chromatography on Biotage (gradient Heptane/EtOAc 100/0 to 80/20) to obtain the product **13i** (38.3 mg, the product contained impurities that could not be completely removed; therefore, the isolated yield is not reported) as colorless oil. **TLC** (EtOAc /Heptane 1/20) Rf = 0.18. **^{1}H NMR** (500 MHz, $CDCl_3$) δ 7.32–7.27 (m, 2H, Ar*H*), 7.27–7.23 (m, 2H, Ar*H*), 4.61 (d, *J* = 7.6 Hz, 1H, ArC*H*Cl), 3.71 (q, *J* = 7.2 Hz, 1H, ArC*H*CH_3), 3.65 (s, 3H, OCH_3), 2.26–2.19 (m, 1H, CHClC*H*), 1.48 (d, *J* = 7.2 Hz, 3H, ArCHCH_3), 1.09 (d, *J* = 6.6 Hz, 3H, CClCHCH_3), 0.86 (d, *J* = 6.7 Hz, 3H, CClCHCH_3). **^{13}C NMR** (126 MHz, $CDCl_3$) δ 175.0, 140.3, 140.0, 127.9, 127.6, 52.2, 45.2, 36.7, 20.3, 19.7, 19.2, 18.7. NMR data matches reported value.[4]

5. One-pot functionalization.

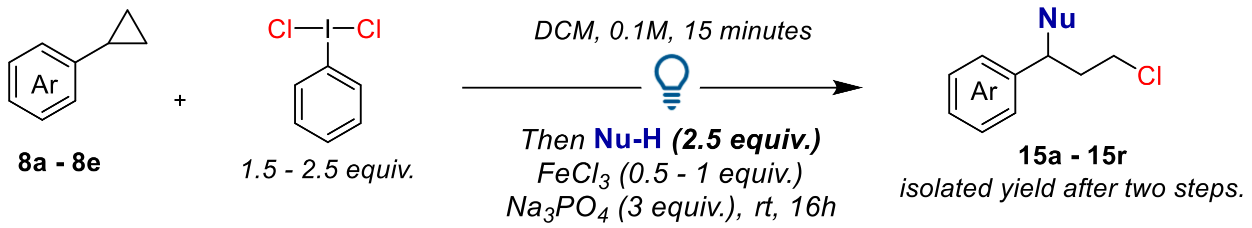

General procedure D: In a 4 mL Vial, dichloro(phenyl)-λ3-iodane (1.5 equiv.) was added followed by addition of the starting material (0.20 mmol, 1.0 equiv) if as solid, then DCM (2.0 mL, 0.1 M). The starting material as liquid was added after DCM addition. The vial was flushed with nitrogen for 10 seconds before sealing with a cap. The reaction mixture was stirred at room temperature under irradiation of a Evolution chem 30 W LED (l_{max} = 450 nm) for 15 minutes. After that, the nucleophile (2.5 equiv.) and Na_3PO_4 (0.098 g, 0.60 mmol, 3.0 equiv.) were added, then the mixture was stirred for 10 seconds before the addition of $FeCl_3$ (0.5 equiv. – 1.0 equiv.). It is important that $FeCl_3$ is added at the end to ensure reproducibility. The mixture was stirred at room temperature for 18 hours. The resulting crude mixture was then concentrated under vacuo and purified by column chromatography.

4-(3-Chloro-1-phenylpropyl)-1,1'-biphenyl (15a)

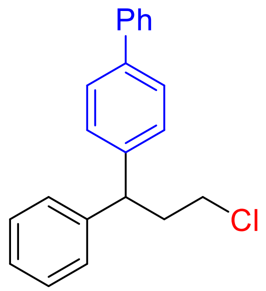

Following general procedure D, starting from **8a** (0.024 g, 0.20 mmol, 1.0 equiv), biphenyl (0.077 g, 0.50 mmol, 2.5 equiv.) and $FeCl_3$ (16.2 mg, 0.100 mmol, 0.500 equiv.the crude mixture was purified on reverse phase column chromatography Biotage (gradient 5% to 95% of CH_3CN – water 0.1% formic acid) to give **15a** (47.1 mg, 0.154 mmol, 77 %) as colorless solid. TLC (Et_2O/heptane 1/20): Rf = 0.42. **^{1}H NMR** (500 MHz, $CDCl_3$) δ 7.59–7.55 (m, 2H, Ar*H*). 7.55–7.51 (m, 2H, Ar*H*), 7.47–7.38 (m, 2H, Ar*H*), 7.36–7.27 (m, 7H, Ar*H*), 7.24–7.19 (m, 1H, Ar*H*), 4.27 (t, *J* = 7.8 Hz, 1H, ArC*H*), 3.50 (t, *J* = 6.6 Hz, 2H, CH_2CH_2Cl), 2.54 (dt, *J* = 7.9, 6.6 Hz, 2H, CH_2CH_2Cl). **^{13}C NMR** (126 MHz, $CDCl_3$) δ 143.6, 142.8, 140.9, 139.6, 128.9, 128.8, 128.4, 128.0, 127.5, 127.3, 127.1,

126.8, 47.7, 43.4, 38.3. **HRMS** (Sicrit plasma/LTQ-Orbitrap) m/z: $[M]^+$ Calcd for $C_{21}H_{19}Cl^+$ 306.1170; Found 306.1169.

4-(1-(4-Bromophenyl)-3-chloropropyl)-1,1'-biphenyl (15b)

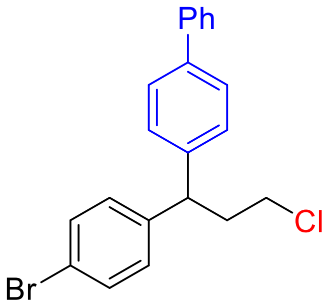

Following general procedure D, starting from **8d** (39.4 mg, 0.200 mmol, 1.00 equiv), biphenyl (0.077 g, 0.50 mmol, 2.5 equiv.) and $FeCl_3$ (16.2 mg, 0.100 mmol, 0.500 equiv.) the crude mixture was purified on reverse phase column chromatography Biotage (gradient 5% to 95% of CH_3CN – water 0.1% formic acid) to give **15b** (53.1 mg, 0.138 mmol, 69 %) as pale-yellow sticky oil. **TLC** (Et_2O/heptane 1/20): Rf = 0.43. **^{1}H NMR** (500 MHz, CD_2Cl_2) δ 7.61 – 7.53 (m, 4H, Ar*H*), 7.48 – 7.40 (m, 4H, Ar*H*), 7.36 – 7.30 (m, 3H, Ar*H*), 7.22 – 7.17 (m, 2H, Ar*H*), 4.24 (t, *J* = 7.8 Hz, 1H, ArC*H*), 3.49 (t, *J* = 6.5 Hz, 2H, CH_2ClCH_2), 2.59 – 2.47 (m, 2H, $ArCHCH_2$). **^{13}C NMR** (126 MHz, CD_2Cl_2) δ 143.2, 142.6, 141.0, 140.0, 132.1, 130.1, 129.2, 128.6, 127.8, 127.7, 127.3, 120.7, 47.4, 43.5, 38.2. **HRMS** (APPI/LTQ-Orbitrap) m/z: $[M]^+$ Calcd for $C_{21}H_{18}BrCl^+$ 384.0275; Found 384.0260.

2-(4-(1-([1,1'-Biphenyl]-4-yl)-3-chloropropyl)phenyl)-4,4,5,5-tetramethyl-1,3,2-dioxaborolane (15c)

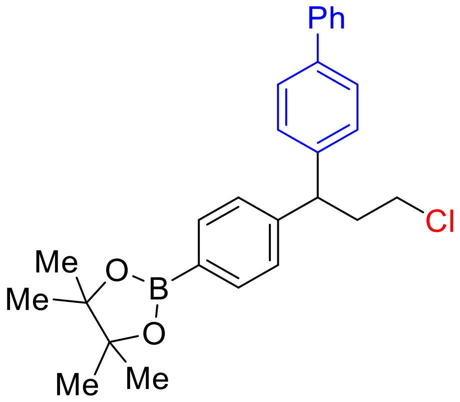

Following general procedure D, starting from **8g** (48.8 mg, 0.200 mmol, 1.00 equiv), biphenyl (77.0 g, 0.500 mmol, 2.50 equiv.) and $FeCl_3$ (16.2 mg, 0.100 mmol, 0.500 equiv.) isolated on column chromatography on Biotage (gradient Heptane/EtOAc 100/0 to 80/20) to obtain **15c** (59.1 mg, 0.137 mmol, 68 %) as white solid. **TLC** (Et_2O/heptane 1/20) Rf = 0.24. ^{1}**H NMR** (500 MHz, $CDCl_3$) δ 7.82–7.76 (m, 2H, Ar*H*). 7.60–7.52 (m, 4H, Ar*H*), 7.47–7.41 (m, 2H, Ar*H*), 7.38–7.30 (m, 5H, Ar*H*), 4.30 (t, *J* = 7.8 Hz, 1H, ArC*H*), 3.50 (td, *J* = 6.6, 2.5 Hz, 2H, CH_2CH_2Cl), 2.56 (dt, *J* = 7.9, 6.6 Hz, 2H, $ArCHCH_2$), 1.35 (s, 12H, 4xCH_3). ^{13}**C NMR** (126 MHz, $CDCl_3$) δ 146.8, 142.5, 140.9, 139.6, 135.4, 128.9, 128.4, 127.5, 127.3, 127.1, 83.9, 47.8, 43.3, 38.0, 24.99, 24.97. (2 aromatic carbons are unresolved). **HRMS** (APCI/QTOF) m/z: $[M + Na]^+$ Calcd for $C_{27}H_{30}BClNaO_2^+$ 455.1920; Found 455.1915.

1-(4-(1-([1,1'-Biphenyl]-4-yl)-3-chloropropyl)phenyl)ethan-1-one (15d)

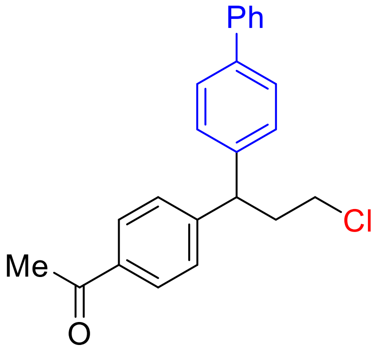

Following general procedure D, starting from **8f** (48.8 mg, 0.200 mmol, 1.00 equiv), biphenyl (0.077 g, 0.500 mmol, 2.50 equiv.) and $FeCl_3$ (16.2 mg, 0.100 mmol, 0.500 equiv.), stirred for 72 hours. The mixture was isolated on column chromatography on Biotage (gradient Heptane/EtOAc 100/0 to 80/20) to obtain **15d** (18.3 mg, 0.0520 mmol, 26 %) as a white sticky oil. **TLC** (Et_2O/heptane 1/7) Rf = 0.14. ^{1}**H NMR** (500 MHz, $CDCl_3$) δ 7.94–7.89 (m, 2H, Ar*H*). 7.58–7.52 (m, 4H, Ar*H*), 7.45–7.37 (m, 4H, Ar*H*), 7.36–7.28 (m, 3H, Ar*H*), 4.35 (t, *J* = 7.8 Hz, 1H, ArC*H*), 3.49 (td, *J* = 6.3, 1.6 Hz, 2H, CH_2Cl), 2.58 (s, 3H, $COCH_3$), 2.57–2.52 (m, 2H, $ArCHCH_2$). ^{13}**C NMR** (126 MHz, $CDCl_3$) δ 197.8, 149.2, 141.7, 140.7, 140.0, 135.8, 129.0, 128.9, 128.4, 128.3, 127.7, 127.5, 127.1, 47.6, 43.0, 37.9, 26.7. **HRMS** (nanochip-ESI/LTQ-Orbitrap) m/z: $[M + H]^+$ Calcd for $C_{23}H_{22}ClO^+$ 349.1354; Found 349.1370.

2-(1-([1,1'-Biphenyl]-4-yl)-3-chloropropyl)naphthalene (15e)

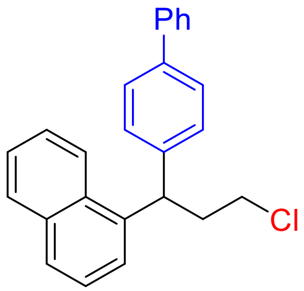

Following general procedure D, starting from **8d** (33.6 mg, 0.200 mmol, 1.0 equiv), biphenyl (0.077 g, 0.50 mmol, 2.5 equiv.) and $FeCl_3$ (16.2 mg, 0.100 mmol, 0.50 equiv.) isolated on reverse phase column chromatography Biotage (gradient 5% to 95% of CH_3CN – water 0.1% formic acid) to obtain **15e** (44.3 mg, 0.124 mmol, 62 %) as pale-yellow sticky oil. **TLC** (Et_2O/heptane 1/20) Rf = 0.42. **^{1}H NMR** (500 MHz, CD_2Cl_2) δ 8.24 – 8.18 (m, 1H, Ar*H*), 7.91 – 7.84 (m, 1H, Ar*H*), 7.79 (dt, *J* = 7.7, 1.2 Hz, 1H, Ar*H*), 7.60 – 7.45 (m, 8H, Ar*H*), 7.43 – 7.38 (m, 4H, Ar*H*), 7.35 – 7.28 (m, 1H, Ar*H*), 5.10 (dd, *J* = 8.4, 6.8 Hz, 1H, ArC*H*), 3.68 – 3.55 (m, 2H, CH_2Cl), 2.75 – 2.60 (m, 2H, ArCHCH_2). **^{13}C NMR** (126 MHz, CD_2Cl_2) δ 143.1, 141.0, 139.8, 139.5, 134.6, 132.2, 129.3, 129.1, 128.9, 127.8, 127.7, 127.6, 127.3, 126.5, 126.0, 125.8, 124.6, 124.0, 43.9, 43.1, 38.9. **HRMS** (APPI/LTQ-Orbitrap) m/z: $[M]^+$ Calcd for $C_{25}H_{21}Cl^+$ 356.1326; Found 356.1318.

4-(3-Chloro-1-phenylpropyl)phenol (15f1)

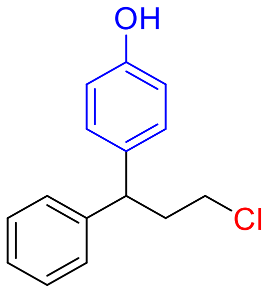

2-(3-Chloro-1-phenylpropyl)phenol (15f2)

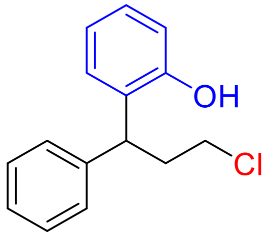

Following general procedure D, starting from **8a** (23.6 mg, 0.200 mmol, 1.00 equiv), phenol (0.077 g, 0.500 mmol, 2.50 equiv.) and $FeCl_3$ (16.2 mg, 0.100 mmol, 0.500 equiv.) isolated on reverse phase column chromatography Biotage (gradient 5% to 95% of CH_3CN – water 0.1% formic acid) to obtain products as two regioisomers (total 52% yield) as an orange oil.

Para isomer **15f1** (22.4 mg, 0.0910 mmol, 45%). **TLC** (Et_2O/heptane 2/3) Rf = 0.55. **^{1}H NMR** (500 MHz, $CDCl_3$) δ 7.34–7.27 (m, 2H, Ar*H*). 7.24–7.17 (m, 3H, Ar*H*), 7.14–7.09 (m, 2H, Ar*H*), 6.82–6.71 (m, 2H, Ar*H*), 4.72 (s, 1H, O*H*), 4.16 (t, *J* = 7.8 Hz, 1H, ArC*H*), 3.45 (t, *J* = 6.6 Hz, 2H, CH_2Cl), 2.56–2.34 (m, 2H, ArCHCH_2). **^{13}C NMR** (126 MHz, $CDCl_3$) δ 154.2, 144.0, 136.0, 129.2, 128.8, 127.9, 126.6, 115.6, 47.2, 43.4, 38.4. **HRMS** (APPI/LTQ-Orbitrap) m/z: $[M]^+$ Calcd for $C_{15}H_{15}ClO^+$ 246.0806; Found 246.0811**.**

Ortho isomer **15f2** (3.2 mg, 0.0130 mmol, 7%). **TLC** (Et_2O/heptane 2/3) Rf = 0.63. **^{1}H NMR (**500 MHz, $CDCl_3$) δ 7.34 – 7.28 (m, 4H, Ar*H*), 7.25 – 7.18 (m, 2H, Ar*H*), 7.15 – 7.09 (m, 1H, Ar*H*), 6.94 (td, *J* = 7.5, 1.2 Hz, 1H, Ar*H*), 6.74 (dd, *J* = 8.0, 1.3 Hz, 1H, Ar*H*), 4.66 (s, 1H, O*H*), 4.50 (t, *J* = 7.8 Hz, 1H, ArC*H*), 3.58 – 3.44 (m, 2H, CH_2Cl), 2.64 – 2.42 (m, 2H, ArCHCH_2). **^{13}C NMR** (126 MHz, $CDCl_3$) δ 153.5, 142.8, 129.8, 128.9, 128.2, 128.2, 127.9, 126.9, 121.3, 116.3, 43.4, 41.5, 37.3. **HRMS** (APPI/LTQ-Orbitrap) m/z: $[M]^+$ Calcd for $C_{15}H_{15}ClO^+$ 246.0806; Found 246.0812.

5-(3-Chloro-1-phenylpropyl)benzo[*d*][1,3]dioxole (15g)

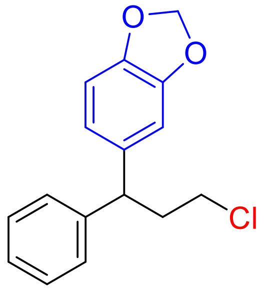

Following general procedure D, starting from **8a** (23.6 mg, 0.200 mmol, 1.00 equiv), benzo[*d*][1,3]dioxole (61.1 mg, 0.500 mmol, 2.50 equiv.) and $FeCl_3$ (16.2 mg, 0.100 mmol, 0.500 equiv.) isolated on reverse phase column chromatography Biotage (gradient 5% to 95% of CH_3CN – water 0.1% formic acid) to obtain product **15g** (41.9 mg, 0.153 mmol, 76%) as a pale yellow oil. **TLC** (Et_2O/heptane 1/10) Rf = 0.43. **^{1}H NMR** (500 MHz, $CDCl_3$) δ 7.32–7.27 (m, 2H, Ar*H*). 7.25–7.17 (m, 3H, Ar*H*), 6.74 (s, 2H, Ar*H*), 6.70 (s, 1H, Ar*H*), 5.91 (s, 2H, OCH_2), 4.14 (t, *J* = 7.8 Hz, 1H, ArC*H*), 3.45 (t, *J* = 6.6 Hz, 2H, CH_2Cl), 2.48–2.36 (m, 2H, ArCHCH_2). **^{13}C NMR** (126 MHz,

$CDCl_3$) δ 148.0, 146.3, 143.8, 137.6, 128.8, 127.8, 126.7, 121.0, 108.4, 101.1, 47.6, 43.3, 38.3, 1 carbon is unresolved. **HRMS** (APPI/LTQ-Orbitrap) m/z: $[M]^+$ Calcd for $C_{16}H_{15}ClO_2^+$ 274.0755; Found 274.0751.

3-(3-Chloro-1-phenylpropyl)benzo[b]thiophene (15h)

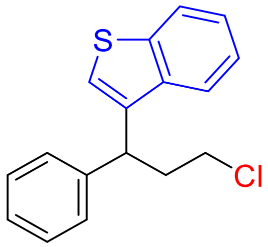

Following general procedure D, starting from **8a** (23.6 mg, 0.200 mmol, 1.00 equiv), benzo[b]thiophene (66.5 mg, 0.500 mmol, 2.50 equiv.) and $FeCl_3$ (16.2 mg, 0.100 mmol, 0.500 equiv.) isolated on reverse phase column chromatography Biotage (gradient 5% to 95% of CH_3CN – water 0.1% formic acid) to obtain product **15h** (38.0 mg, 0.132 mmol, 66 %) as an orange oil. **TLC** (Et_2O/heptane 1/20) Rf = 0.44. **^{1}H NMR** (500 MHz, $CDCl_3$) δ 7.90–7.82 (m, 1H, Ar*H*). 7.76–7.67 (m, 1H, Ar*H*), 7.40-7.29 (m, 5H, Ar*H*), 7.28–7.20 (m, 3H, Ar*H*), 4.66–4.62 (m, 1H, ArC*H*), 3.65–3.56 (m, 1H, C*H*$_2$Cl), 3.55–3.45 (m, 1H, C*H*$_2$Cl), 2.73–2.60 (m, 1H, 1H, ArCHC*H*$_2$), 2.60–2.49 (m, 1H, ArCHC*H*$_2$). **^{13}C NMR** (126 MHz, $CDCl_3$) δ 142.0, 140.6, 138.5, 138.3, 128.8, 128.0, 126.9, 124.4, 124.0, 122.8, 122.3, 121.5, 43.1, 42.0, 38.4. **HRMS** (APPI/LTQ-Orbitrap) m/z: $[M]^+$ Calcd for $C_{17}H_{15}ClS^+$ 286.0578; Found 286.0586

Methyl 2-(6-(3-chloro-1-phenylpropyl)-5-methoxy-2-methyl-1H-indol-3-yl)acetate (15i)

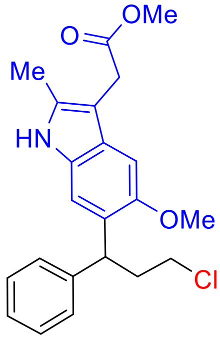

Following general procedure D, starting from **8a** (23.6 mg, 0.200 mmol, 1.00 equiv), Indomethacin methyl ester (186 mg, 0.500 mmol, 2.50 equiv.) and $FeCl_3$ (16.2 mg, 0.100 mmol, 0.500 equiv.)

isolated on reverse phase column chromatography Biotage (gradient 5% to 95% of CH_3CN – water 0.1% formic acid) to obtain product **15i** (35.6 mg, 0.0910 mmol, 45%) as an orange oil. **TLC** (EtOAc/heptane 1/3) Rf = 0.28. **^{1}H NMR** (500 MHz, $CDCl_3$) δ 7.67 (s, 1H, N*H*), 7.29–7.23 (m, 4H, Ar*H*), 7.18–7.12 (m, 1H, Ar*H*), 7.04 (s, 1H, Ar*H*), 6.93 (s, 1H, Ar*H*), 4.65 (t, *J* = 7.8 Hz, 1H, ArC*H*CH$_2$), 3.80 (s, 3H, ArOC*H$_3$*), 3.66 (s, 3H, CO$_2$C*H$_3$*), 3.64 (s, 2H, ArC*H$_2$*CO$_2$Me), 3.54–3.42 (m, 2H, C*H$_2$*Cl), 2.59–2.41 (m, 2H, ArCHC*H$_2$*), 2.37 (s, 3H, ArC*H$_3$*).**^{13}C NMR** (126 MHz, $CDCl_3$) δ 172.6, 152.3, 144.3, 133.0, 130.0, 128.4, 128.2, 127.6, 127.3, 126.1, 109.7, 104.4, 99.8, 56.3, 52.0, 43.7, 41.7, 38.2, 30.5, 11.9. **HRMS** (APCI/QTOF) m/z: [M + H]+ Calcd for C22H25ClNO3+ 386.1517; Found 386.1512.

N-(2-(4-(3-Chloro-1-phenylpropyl)phenoxy)-4-nitrophenyl)methanesulfonamide (15j)

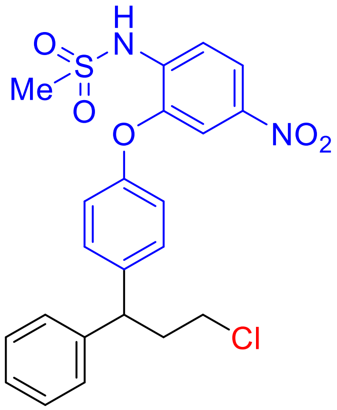

Following general procedure D, starting from **8a** (23.6 mg, 0.200 mmol, 1.00 equiv), Nimesulide (154 mg, 0.500 mmol, 2.50 equiv.) and $FeCl_3$ (16.2 mg, 0.100 mmol, 0.500 equiv.) isolated on reverse phase column chromatography Biotage (gradient 5% to 95% of CH_3CN – water 0.1% formic acid) to obtain product **15j** (56.4 mg, 0.122 mmol, 61%) as a pale-yellow oil. **TLC** (EtOAc/heptane 1/3) Rf = 0.16. **^{1}H NMR** (500 MHz, $CDCl_3$) δ 7.98 (dd, *J* = 9.0, 2.4 Hz, 1H, Ar*H*). 7.73 (d, *J* = 9.0 Hz, 1H, Ar*H*), 7.64 (d, *J* = 2.4 Hz, 1H, Ar*H*), 7.37 (s, 1H, N*H*) 7.36–7.29 (m, 4H, Ar*H*), 7.27–7.21 (m, 3H, Ar*H*), 7.01–6.92 (m, 2H, Ar*H*), 4.26 (t, *J* = 7.8 Hz, 1H, ArC*H*), 3.46 (td, *J* = 6.5, 2.1 Hz, 2H, C*H$_2$*Cl), 3.13 (s, 3H, SC*H$_3$*), 2.58–2.37 (m, 2H, ArCHCH$_2$). **^{13}C NMR** (126 MHz, $CDCl_3$) δ 152.9, 146.3, 143.8, 142.9, 141.6, 134.2, 130.1, 129.0, 128.0, 127.0, 119.8, 119.6, 117.5, 112.2, 47.3, 43.1, 40.7, 38.3. **HRMS** (nanochip-ESI/LTQ-Orbitrap) m/z: $[M + Na]^+$ Calcd for $C_{22}H_{21}ClN_2NaO_5S^+$ 483.0752; Found 483.0775.

(8R,9S,13S,14S)-2-(3-Chloro-1-phenylpropyl)-3-methoxy-13-methyl-6,7,8,9,11,12,13,14,15,16-decahydro-17H-cyclopenta[a]phenanthren-17-one (15k)

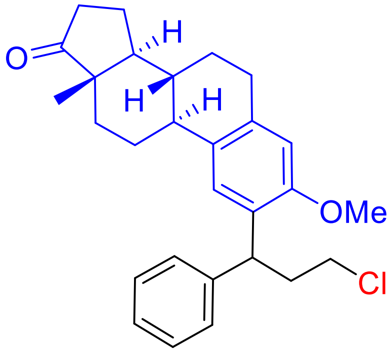

Following general procedure D, starting from **8a** (23.6 mg, 0.200 mmol, 1.00 equiv), estrone methyl ether (142 mg, 0.500 mmol, 2.50 equiv.) and $FeCl_3$ (16.2 mg, 0.100 mmol, 0.500 equiv.) isolated on reverse phase column chromatography Biotage two times (gradient 5% to 95% of CH_3CN – water 0.1% formic acid) to obtain product **15k** (31.2 mg, 0.0710 mmol, 36%) as a pale-yellow sticky oil, mixture of two diastereomer. dr 53:47 was determined by NMR signal at ArH-estrone, major 7.12 ppm, minor 7.10 ppm. **TLC** (EtOAc/heptane 1/10) Rf = 0.15.

^{1}H NMR major only (500 MHz, $CDCl_3$) δ 7.29 – 7.26 (m, 3H, Ar*H*), 7.26 – 7.24 (m, 1H, Ar*H*), 7.19 – 7.14 (m, 1H, Ar*H*), 7.12 (s, 1H, Ar*H*), 6.56 (s, 1H, Ar*H*), 4.53 (td, *J* = 7.9, 3.1 Hz, 1H, ArC*H*), 3.74 (s, 3H, OCH_3), 3.51 – 3.39 (m, 2H, CH_2Cl), 2.92 – 2.79 (m, 2H, estrone-C*H*), 2.55 – 2.41 (m, 3H, CH_2 and estrone-C*H*), 2.40 – 2.32 (m, 1H, CH_2), 2.28 – 2.19 (m, 1H, estrone-C*H*), 2.17 – 2.10 (m, 1H, estrone-C*H*), 2.06 – 1.92 (m, 3H, estrone-C*H*), 1.66 – 1.58 (m, 2H, estrone-C*H*), 1.55 – 1.33 (m, 5H, estrone-CH_2), 0.90 (s, 3H, $COCCH_3$).

^{13}C NMR for two diastereomer (126 MHz, $CDCl_3$) δ 221.14 (2 carbons) 155.3, 155.2, 143.8, 143.7, 135.7(2 carbons), 131.74, 131.67, 129.7, 129.7, 128.5(2 carbons), 128.19, 128.16, 126.3(2 carbons), 124.94, 124.86, 111.4 (2 carbons), 55.7(2 carbons), 50.5(2 carbons), 48.2(2 carbons), 44.3(2 carbons), 44.2, 43.6, 41.3, 41.2, 38.6, 38.5, 37.9, 37.8, 36.1, 36.0, 31.8, 31.7, 29.71, 29.68, 26.7(2 carbons), 26.2, 26.1, 21.7(2 carbons), 14.0 (2 carbons).

HRMS (ESI/QTOF) m/z: $[M + Na]^+$ Calcd for $C_{28}H_{33}ClNaO_2^+$ 459.2061; Found 459.2061.

N-(3-Chloro-1-phenylpropyl)-4-methoxybenzenesulfonamide (15l)

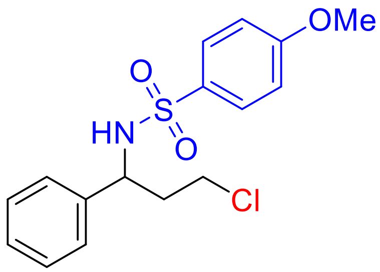

Following general procedure D, starting from **8a** (23.6 mg, 0.200 mmol, 1.00 equiv), 4-methoxybenzenesulfonamide (94.0 mg, 0.500 mmol, 2.50 equiv.) and $FeCl_3$ (32.0 mg, 0.200 mmol, 1.00 equiv.). The mixture was isolated on column chromatography on Biotage (gradient Heptane/EtOAc 100/0 to 60/40) to obtain **15l** (51.3 mg, 0.151 mmol, 75%) as a white solid. **TLC** (SiO_2, Et_2O/heptane 1/4) Rf = 0.36. **^{1}H NMR** (500 MHz, $CDCl_3$) δ 7.67–7.53 (m, 2H, Ar*H*), 7.24–7.14 (m, 3H, Ar*H*), 7.05–6.99 (m, 2H, Ar*H*), 6.86–6.73 (m, 2H, Ar*H*), 4.51 (q, *J* = 7.4 Hz, 1H, N*H*), 4.90 (d, *J* = 7.7 Hz, 1H, ArC*H*), 3.83 (s, 3H, OC*H₃*), 3.56 – 3.43 (m, 1H, C*H₂*Cl), 3.38–3.24 (m, 1H, C*H₂*Cl), 2.36–2.23 (m, 1H, ArCHC*H₂*), 2.15–2.04 (m, 1H, ArCHC*H₂*). **^{13}C NMR** (126 MHz, $CDCl_3$) δ 162.9, 139.6, 132.0, 129.4, 129.0, 128.1, 126.6, 114.1, 55.9, 55.7, 41.2, 40.1. **HRMS** (ESI/QTOF) m/z: $[M + Na]^+$ Calcd for $C_{16}H_{18}ClNNaO_3S^+$ 362.0588; Found 362.0589.

N-(3-Chloro-1-phenylpropyl)-4-(5-(p-tolyl)-3-(trifluoromethyl)-1H-pyrazol-1-yl)benzenesulfonamide (15m)

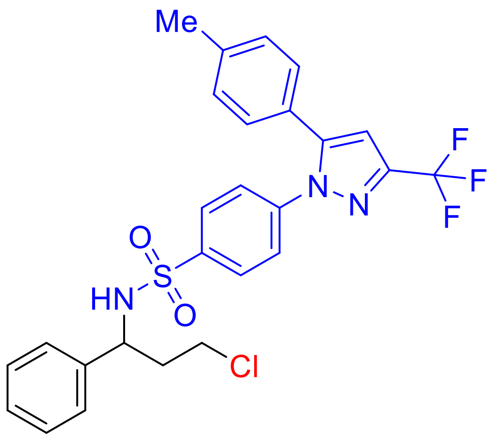

Following general procedure D, starting from **8a** (23.6 mg, 0.200 mmol, 1.00 equiv), Celecoxib (191 mg, 0.500 mmol, 2.50 equiv.) and $FeCl_3$ (32.0 mg, 0.200 mmol, 1.00 equiv.). The mixture was isolated on column chromatography on Biotage (gradient Heptane/EtOAc 100/0 to 60/40) to obtain **15m** (75.0 mg, 0.140 mmol, 70%) as a white sticky solid. **TLC** (SiO_2, Et_2O/heptane 1/4) Rf

= 0.28. **¹H NMR** (500 MHz, $CDCl_3$) δ 7.68–7.63 (m, 2H, Ar*H*). 7.34–7.28 (m, 2H, Ar*H*), 7.23 (dd, *J* = 5.0, 1.9 Hz, 3H, Ar*H*), 7.16 (s, 2H, Ar*H*), 7.03–7.08 (m, 4H, Ar*H*), 6.72 (s, 1H, Ar*H*), 5.15 (d, *J* = 7.7 Hz, 1H, N*H*), 4.58 (q, *J* = 7.4 Hz, 1H, ArC*H*), 3.55–3.38 (m, 1H, $ClCH_2$), 3.36–3.21 (m, 1H, $ClCH_2$), 2.38 (s, 3H, $ArCH_3$), 2.32–2.20 (m, 1H, $ArCHCH_2$), 2.17–2.05 (m, 1H, $ArCHCH_2$). **¹³C NMR** (126 MHz, $CDCl_3$) δ 145.3, 144.2 (d, *J* = 38.5 Hz), 142.5, 139.9 (d, *J* = 4.8 Hz), 139.2, 129.8, 129.1, 128.8, 128.5, 128.2, 126.5, 125.9, 125.3, 121.2 (d, *J* = 268.9 Hz), 106.5, 56.0, 41.1, 39.9, 21.5. **¹⁹F NMR** (470 MHz, $CDCl_3$) δ -62.5. **HRMS** (ESI/QTOF) m/z: $[M + H]^+$ Calcd for $C_{26}H_{24}ClF_3N_3O_2S^+$ 534.1224; Found 534.1230.

1-Bromo-4-(2-(3-chloro-1-phenylpropoxy)ethyl)benzene (15n)

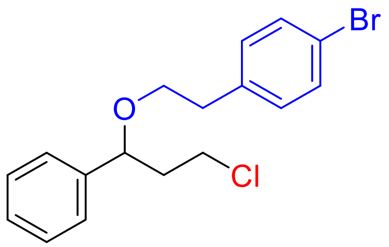

Following general procedure D, starting from **8a** (23.6 mg, 0.200 mmol, 1.0 equiv), 2-(4-bromophenyl)ethan-1-ol (101 mg, 0.500 mmol, 2.5 equiv.) and $FeCl_3$ (32.0 mg, 0.200 mmol, 1.00 equiv.). The mixture was isolated on column chromatography on Biotage (gradient Heptane/EtOAc 100/0 to 80/20) to obtain **15n** (52.3 mg, 0.148 mmol, 74 %) as a colorless oil. **TLC** (SiO_2, Et_2O/heptane 1/20) Rf = 0.41. **¹H NMR** (500 MHz, $CDCl_3$) δ 7.43–7.35 (m, 2H, Ar*H*), 7.34–7.3 (m, 2H, Ar*H*), 7.29–7.26 (m, 1H, Ar*H*), 7.24–7.18 (m, 2H, Ar*H*), 7.09–6.99 (m, 2H, Ar*H*), 4.46 (dd, *J* = 8.8, 4.6 Hz, 1H, ArC*H*), 3.71–3.60 (m, 1H, $ClCH_2$), 3.60–3.52 (m, 1H, OCH_2), 3.50–3.44 (m, 2H, $ClCH_2$ and OCH_2), 2.87–2.74 (m, 2H, CH_2), 2.23–2.12 (m, 1H, CH_2), 2.04–1.89 (m, 1H, CH_2). **¹³C NMR** (126 MHz, $CDCl_3$) δ 141.6, 138.3, 131.4, 130.8, 128.7, 128.0, 126.6, 120.1, 78.9, 69.4, 41.8, 41.2, 35.9. **HRMS** (APCI/QTOF) m/z: $[M + Na]^+$ Calcd for $C_{17}H_{18}BrClNaO^+$ 375.0122; Found 375.0106.

3-(3-Chloro-1-phenylpropoxy)propanenitrile (15o)

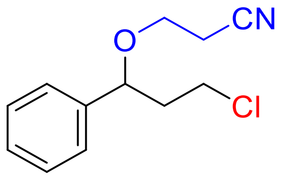

Following general procedure D, starting from **8a** (23.6 mg, 0.200 mmol, 1.00 equiv), 3-hydroxypropanenitrile (0.036 g, 0.500 mmol, 2.50 equiv.) and $FeCl_3$ (32.0 mg, 0.200 mmol, 1.0 equiv.). The mixture was isolated on column chromatography on Biotage (gradient Heptane/EtOAc 100/0 to 65/35) to obtain **15o** (29.1 mg, 0.130 mmol, 65 %) as a pale yellow oil. **TLC** (Et_2O/heptane 1/4) Rf = 0.15. **^{1}H NMR** (500 MHz, $CDCl_3$) δ 7.41–7.35 (m, 2H, Ar*H*). 7.35–7.28 (m, 3H, Ar*H*), 4.55 (dd, *J* = 8.8, 4.5 Hz, 1H, ArC*H*), 3.84–3.69 (m, 1H, ClC*H*$_2$), 3.63–3.4 (m, 3H, OC*H*$_2$ and ClC*H*$_2$), 2.65–2.49 (m, 2H, C*H*$_2$), 2.34–2.22 (m, 1H, C*H*$_2$), 2.10–1.93 (m, 1H, C*H*$_2$). **^{13}C NMR** (126 MHz, $CDCl_3$) δ 140.6, 128.9, 128.4, 126.6, 117.9, 79.4, 63.5, 41.6, 40.9, 19.1. **HRMS** (APCI/QTOF) m/z: $[M + Na]^+$ Calcd for $C_{12}H_{14}ClNNaO^+$ 246.0656; Found 246.0665.

(3-Chloro-1-phenylpropyl)(cyclohexyl)sulfane (15p)

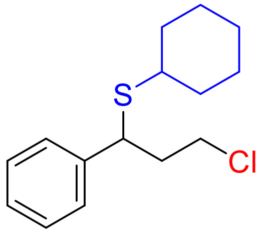

Following general procedure D, starting from **8a** (23.6 mg, 0.200 mmol, 1.00 equiv), cyclohexanethiol (58.1 mg, 0.500 mmol, 2.50 equiv.) and $FeCl_3$ (32.0 mg, 0.200 mmol, 1.00 equiv.). The mixture was isolated on column chromatography on Biotage (gradient Heptane/EtOAc 100/0 to 65/35) to obtain **15p** (35.4 mg, 0.132 mmol, 66%) as a pale yellow oil. **TLC** (Et_2O/heptane 1/20) Rf = 0.41. **^{1}H NMR** (500 MHz, $CDCl_3$) δ 7.37 – 7.28 (m, 4H, Ar*H*), 7.26 – 7.22 (m, 1H, Ar*H*), 4.11 (dd, *J* = 8.3, 6.7 Hz, 1H, ArC*H*), 3.64 – 3.52 (m, 1H, ClC*H*$_2$), 3.42 – 3.27 (m, 1H, ClC*H*$_2$), 2.46 – 2.39 (m, 1H, SC*H*), 2.33 – 2.25 (m, 1H, ArCHC*H*$_2$), 2.25 – 2.15 (m, 1H, ArCHC*H*$_2$), 2.02 – 1.91 (m, 1H, C*H*$_2$), 1.77 – 1.68 (m, 2H, C*H*$_2$), 1.68 – 1.61 (m, 1H, C*H*$_2$), 1.56 – 1.49 (m, 1H, C*H*$_2$), 1.38 – 1.29 (m, 1H, C*H*$_2$), 1.28 – 1.12 (m, 4H, C*H*$_2$). **^{13}C NMR** (126 MHz, $CDCl_3$) δ 142.4, 128.8, 127.8, 127.4, 45.0, 43.0, 42.8, 39.7, 33.9, 33.5, 26.1, 25.9, 25.9. **HRMS** (APCI/QTOF) m/z: $[M + H]^+$ Calcd for $C_{15}H_{22}ClS^+$ 269.1125; Found 269.1129.

4-(1-Phenylethyl)-1,1'-biphenyl (15q)

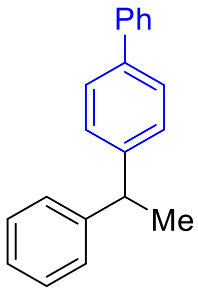

Following general procedure D, starting from **12b** (0.021 g, 0.200 mmol, 1.00 equiv), biphenyl (77.0 mg, 0.500 mmol, 2.50 equiv.) and $FeCl_3$ (16.0 mg, 0.100 mmol, 0.500 equiv.). The mixture was isolated on column chromatography on Biotage (Heptane) to obtain **15q** (31.3 mg, 0.120 mmol, 61 %) as a colorless oil. **TLC** (EtOAc/hexane 1/10) Rf = 0.64. **^{1}H NMR** (500 MHz, $CDCl_3$) δ 7.61 – 7.57 (m, 2H, Ar*H*), 7.56 – 7.51 (m, 2H, Ar*H*), 7.44 (t, *J* = 7.7 Hz, 2H, Ar*H*), 7.36 – 7.27 (m, 7H, Ar*H*), 7.25 – 7.20 (m, 1H, Ar*H*), 4.22 (q, *J* = 7.2 Hz, 1H, ArC*H*), 1.70 (d, *J* = 7.2 Hz, 3H, C*H₃*). **^{13}C NMR** (126 MHz, $CDCl_3$) δ 146.4, 145.6, 141.1, 139.1, 128.8, 128.6, 128.2, 127.8, 127.3, 127.2, 127.2, 126.3, 44.6, 22.0. NMR data matches reported value.[21]

5-(2-Chloro-1-phenylpropyl)benzo[d][1,3]dioxole (15r)

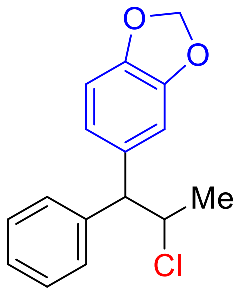

Following general procedure D, starting from **10f** (23.6 mg, 0.200 mmol, 1.00 equiv), benzo[*d*][1,3]dioxole (61.1 mg, 0.500 mmol, 2.50 equiv.) and $FeCl_3$ (16.0 mg, 0.100 mmol, 0.500 equiv.). The mixture was isolated on column chromatography on Biotage (gradient Heptane/EtOAc 100/0 to 80/20) to obtain **15r** (33.1 mg, 0.120 mmol, 60 %) as a pale-yellow oil, mixture of two diastereomer. dr 1.6:1 was determined by NMR signal CH_3 major 1.46 ppm, minor 1.49 ppm : **TLC** (EtOAc/hexane 1/20) Rf = 0.33.

[21] Mahajani, N. S.; Chisholm, J. D. Synthesis of 1,1′-Diarylethanes and Related Systems by Displacement of Trichloroacetimidates with Trimethylaluminum. *J. Org. Chem.* **2018**, *83*, 4131–4139.

All chemical shifts proton of two diastereomers are overlapped, only CH_3 signal are separated. **^{1}H NMR** (500 MHz, $CDCl_3$) δ 7.35–7.3 (m, 3H, Ar*H*). 7.24–7.21 (m, 2H, Ar*H*), 6.86–6.79 (m, 2H, Ar*H*), 6.77–6.73 (m, 1H, Ar*H*), 5.93–5.91 (m, 2H, OCH_2), 4.70–4.68 (m, 1H, ArC*H*), 3.98–3.96 (m, 1H, C*H*Cl), Major 1.46 (d, *J* = 6.4 Hz, 3H, CH_3), Minor 1.49 (d, *J* = 6.5 Hz, 3H, CH_3). **^{13}C NMR** (126 MHz, $CDCl_3$) for both diastereomers δ 148.0, 147.8, 146.6, 146.4, 142.3 (2 carbons), 136.24, 136.16, 129.0, 128.6, 128.1, 127.9, 127.1, 126.9, 121.5, 121.4, 108.60, 108.57, 108.30, 108.26, 101.2, 101.1, 60.80, 60.77, 59.9, 59.5, 24.6, 24.5. **HRMS** (APPI/LTQ-Orbitrap) m/z: $[M]^+$ Calcd for $C_{16}H_{15}ClO_2^+$ 274.0755; Found 274.0761.

6. Sequence chlorination/reductive coupling

Protocol for optimization

In a 4 mL Vial, the chlorinating reagent **6i** was added, then DCM (1.0 mL, 0.1 M), followed by addition of cyclopropyl benzene (12.5 μL, 0.100 mmol, 1.00 equiv). The vial was flushed with nitrogen for 10 seconds before sealing with a cap. The reaction mixture was stirred at room temperature under irradiation of a Kessil lamp (I_{max} = 440 nm) for 10 minutes. The reaction mixture obtained was concentrated under reduced pressure to remove the solvent, then DMA (2.0 mL, 0.05 M) was added to the crude residue and it was transferred to another 12*75 mm Borosilicate glass tube, which was previously charged with the nickel catalyst. The additive, Zn and the ligand were then added. The reaction was stirred at room temperature for 18 hours. The reaction was quenched by loading directly onto a short plug of silica, eluting with diethylether. The solution was then extracted with water and the combined organic layers were dried over Na_2SO_4, filtered, and concentrated in vacuo. The crude mixture was added CH_2Br_2 as internal standard. NMR yield was determined by integration the ArC*H* NMR signal of the products.

Table S1. Optimization of pyridines ligands for the reductive coupling

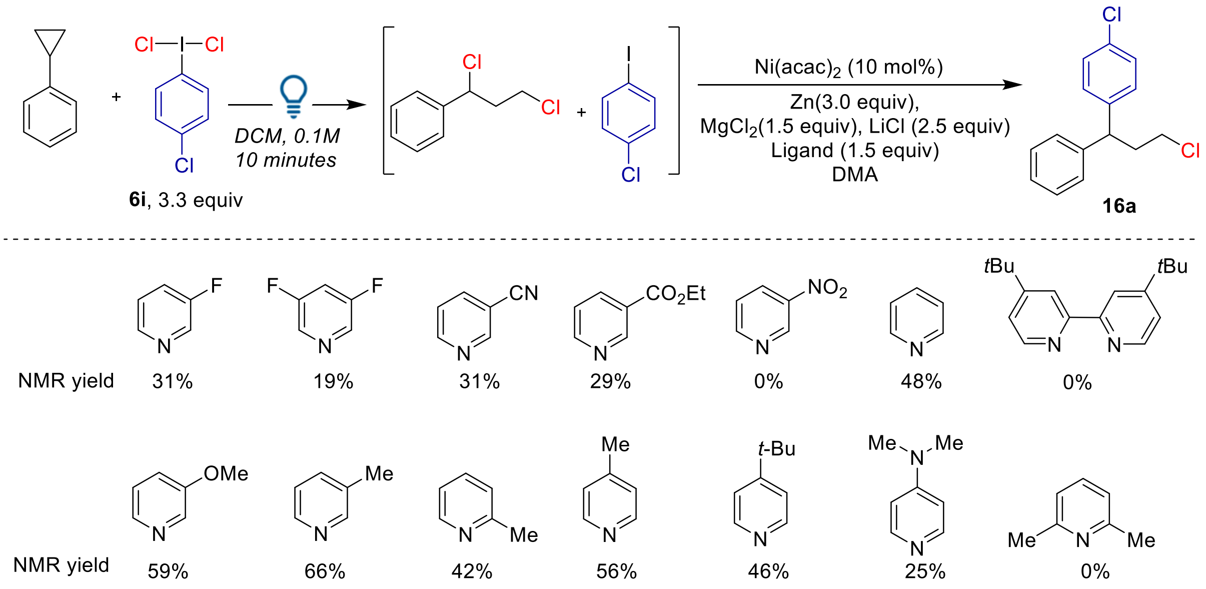

Table S2. Optimization of catalysts and additives for the reductive coupling.

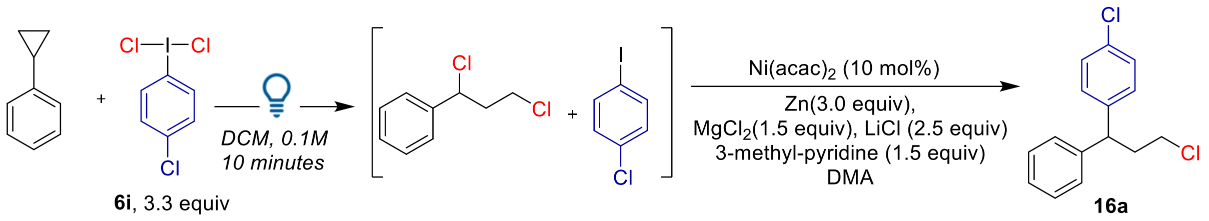

Entry	Variation from standard conditions	**16a**, NMR yield (%)
1	None	66
2	Ni(dtbbpy)Cl_2	21
3	$NiBr_2$(DME)	68
4	$NiCl_2$(DME)	61
5	$NiBr_2$(DME), 2.0 equiv. Zn	50
6	$NiBr_2$(DME) (20 mol%), 2.0 equiv. Zn	56
7	$NiBr_2$(DME) (20 mol%), 1.0 equiv. Zn	40
**8**	**$NiBr_2$(DME) (20 mol%), 2 equiv. Zn, 1.5 equiv. LiCl**	**69**
9	$NiBr_2$(DME) (20 mol%), 2 equiv. Zn, 1 equiv. LiCl	60

Table S3. Fine-tuning reaction conditions for the reductive coupling.

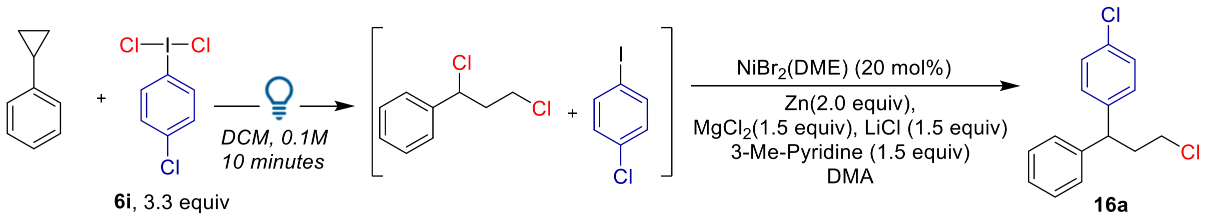

Entry	Variation from standard conditions	**16a**, NMR yield (%)
**1**	**None**	**69**
2	DMF instead of DMA	57
3	Dioxane instead of DMA	33
4	Propylene carbonate instead of DMA	0
5	Mn instead of Zn	0
6	TDAE instead of Zn	0
7	1.5 equiv **6i**	36
8	2 equiv **6i**	46
9	2.5 equiv **6i**	55

Substrate scope protocol

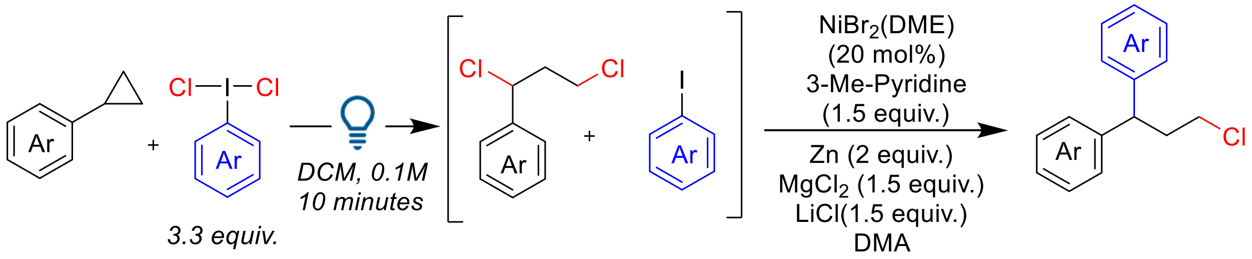

General procedure E, in a 4 mL Vial, The chlorinating reagent **6** (3.3 equiv.) was added followed by addition of the starting material (0.10 mmol, 1.0 equiv) if as solid, then DCM (1.0 mL, 0.1 M). The starting material as liquid was added after DCM addition. The vial was flushed with nitrogen for 10 seconds before sealed with a cap. The reaction mixture was stirred at room temperature under irradiation of Kessil lamp (l_{max} = 440 nm) for 10 minutes. The reaction mixture obtaining was concentrated under reduced pressure to remove solvent, then DMA (2.0 mL) was added to the crude residue to transfer to another 12*75 mm Borosilicate glass tube which was previously charged with $NiBr_2$(DME) (6.2 mg, 0.020 mmol, 20 mol%), $MgCl_2$ (14.3mg, 0.150 mmol, 1.50 equiv), lithium chloride (6.3 mg, 0.150 mmol, 1.50 equiv) and zinc (13.0mg, 0.200 mmol, 2.00 equiv). Next, 3-methylpyridine (14.0 mg, 0.150 mmol, 1.50 equiv) was added. The reaction is stirred at room temperature for 18 hours. The reaction was quenched by loading directly onto a short plug of silica, eluting with diethylether. The solution was then extracted with water and the combined organic layers were dried over Na_2SO_4, filtered, and concentrated in vacuo. The crude mixture was purified by flash silica gel column chromatography to afford 3,3 diarylchloropropane. The reaction was monitored by NMR with CH_2Br_2 as internal standard. NMR yield was determined by integration of ArC*H* NMR of products.

1-Chloro-4-(3-chloro-1-phenylpropyl)benzene (16a)

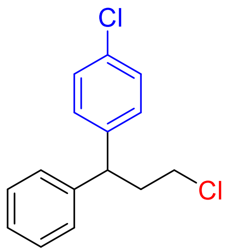

Following the general procedure **E**, starting with **6i** (102 mg, 0.330 mmol, 3.30 equiv) and cyclopropylbenzene (12.5 µL, 0.100 mmol, 1.00 equiv). The crude mixture was purified by flash silica gel column chromatography (0-5% Et_2O in pentane) to afford 1-chloro-4-(3-chloro-1-

phenylpropyl)benzene **(16a)** (16.2 mg, 0.0610 mmol, 61%) as a colourless oil. **TLC** (SiO_2, pentane) Rf = 0.43**.** **^{1}H NMR** (400 MHz, $CDCl_3$) δ 7.36 – 7.28 (m, 4H, Ar*H*), 7.26 – 7.18 (m, 5H, Ar*H*), 4.23 (t, *J* = 7.8 Hz, 1H, ArC*H*), 3.47 (t, *J* = 6.5 Hz, 2H, CH_2CH_2Cl), 2.49 (dtd, *J* = 7.8, 6.5, 4.0 Hz, 2H, CH_2CH_2Cl). **^{13}C NMR** (101 MHz, $CDCl_3$) δ ^{13}C NMR (101 MHz, $CDCl_3$) δ 143.0, 142.1, 132.3, 129.2, 128.8, 127.8, 126.8, 47.4, 47.2, 43.0, 38.0 (1 aromatic carbon is unresolved). **IR** (v_{max}, cm^{-1}) 3060 (w), 3028 (w), 2959 (w), 1598 (w), 1491 (s), 1452 (m), 1289 (w), 1092 (s), 1014 (m), 820 (s). **HRMS** (APPI/LTQ-Orbitrap) m/z: $[M]^+$ Calcd for $C_{15}H_{14}Cl_2^+$ 264.0467; Found 264.0471.

2-Chloro-4-(3-chloro-1-phenylpropyl)-1-methoxybenzene (16b)

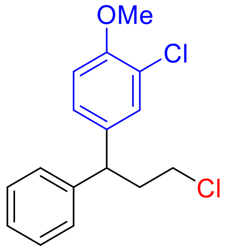

Following the general procedure **E**, starting with **6j** (113 mg, 0.330 mmol, 3.30 equiv) and cyclopropylbenzene (12.5 µL, 0.100 mmol, 1.00 equiv). The crude mixture was purified by flash silica gel column chromatography (0-30% Et_2O in pentane) to afford 2-chloro-4-(3-chloro-1-phenylpropyl)-1-methoxybenzene **(16b)** (14.9 mg, 0.0510 mmol, 51%) as a colourless oil. The reaction was monitored by NMR with CH_2Br_2 as internal standard. **TLC** (SiO_2, 90:10 pentane:ethyl acetate) Rf = 0.65**.** **^{1}H NMR** (400 MHz, $CDCl_3$) δ 7.34 – 7.28 (m, 2H, Ar*H*), 7.26 – 7.19 (m, 4H, Ar*H*), 7.11 (ddd, *J* = 8.5, 2.3, 0.6 Hz, 1H, Ar*H*), 6.86 (d, *J* = 8.5 Hz, 1H, Ar*H*), 4.16 (t, *J* = 7.8 Hz, 1H, ArC*H*), 3.87 (s, 3H, OCH_3), 3.45 (t, *J* = 6.5 Hz, 2H, CH_2CH_2Cl), 2.45 (dtd, *J* = 8.2, 6.5, 1.7 Hz, 2H, CH_2CH_2Cl). **^{13}C NMR** (101 MHz, $CDCl_3$) δ 153.7, 143.3, 137.0, 129.6, 128.9, 127.9, 127.2, 126.8, 122.6, 112.3, 56.3, 46.9, 43.2, 38.2. **IR** (v_{max}, cm^{-1}) 3028 (w), 2959 (w), 2839 (w), 1606 (w), 1500 (s), 1456 (m), 1283 (s), 1258 (s), 1065 (s), 1025 (m), 811 (m). **HRMS** (Sicrit plasma/LTQ-Orbitrap) m/z: $[M]^+$ Calcd for $C_{16}H_{16}Cl_2O^+$ 294.0573; Found 294.0573.

1-(3-Chloro-1-phenylpropyl)-4-(trifluoromethyl)benzene (16c)

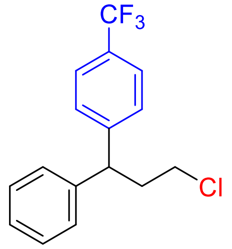

Following the general procedure **E**, starting with **6k** (112.8 mg, 0.330 mmol, 3.30 equiv) and cyclopropylbenzene (12.5 µL, 0.100 mmol, 1 equiv). The crude mixture was purified by flash silica gel column chromatography (0-5% Et_2O in pentane) to afford 1-(3-chloro-1-phenylpropyl)-4-(trifluoromethyl)benzene **(16c)** (14.5 mg, 0.0490 mmol, 49%) as a colourless oil. NMR yield was determined by integration of ArC*H* NMR of products. TLC (SiO_2, pentane) Rf = 0.37**.** ^{1}**H NMR** (400 MHz, $CDCl_3$) δ 7.58 – 7.52 (m, 2H, Ar*H*), 7.40 – 7.35 (m, 2H, Ar*H*), 7.34 – 7.30 (m, 2H, Ar*H*), 7.25 – 7.20 (m, 3H, Ar*H*), 4.31 (t, *J* = 7.8 Hz, 1H, ArC*H*), 3.45 (t, *J* = 6.4 Hz, 2H, CH_2CH_2Cl), 2.51 (dtd, *J* = 7.6, 6.4, 4.8 Hz, 2H, CH_2CH_2Cl). ^{13}**C NMR** (101 MHz, $CDCl_3$) δ 147.70, 142.42, 128.86, 128.22, 127.87, 126.96, 125.61 (q, *J* = 3.8 Hz), 47.61, 42.83, 37.80. ^{19}**F NMR** (376 MHz, $CDCl_3$) δ -62.45. **IR** ($ν_{max}$, cm^{-1}) 3064 (w), 3029 (w), 2962 (w), 1619 (w), 1496 (w), 1326 (s), 1166 (m), 1123 (s), 1069 (m), 831 (w). **HRMS** (Sicrit plasma/LTQ-Orbitrap) m/z: $[M + H_{-1}]^+$ Calcd for $C_{16}H_{13}ClF_3^+$ 297.0652; Found 297.0652.

1-Chloro-4-(3-chloro-1-phenylbutyl)benzene (16d)

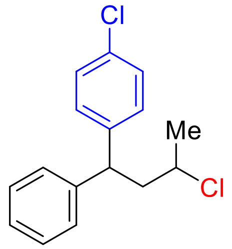

Following the general procedure **E**, starting with **6i** (102 mg, 0.330 mmol, 3.30 equiv) and (2-methylcyclopropyl)benzene (13.2 mg, 0.100 mmol, 1.00 equiv). The crude mixture was purified by flash silica gel column chromatography (0-5% Et_2O in pentane) to afford 1-chloro-4-(3-chloro-1-phenylbutyl)benzene **(16d)** (16.7 mg, 0.0601 mmol, 60%, dr 2:1) as a colourless oil. NMR yield was determined by integration of ArC*H* NMR of products. **TLC** (SiO_2, pentane) Rf = 0.43**. dr** 67:33 was approximately determined by NMR signal of $CH_2CHClCH_3$. ^{1}**H NMR**: ^{1}**H NMR** (400 MHz, $CDCl_3$) δ 7.33 – 7.26 (m, 3H, Ar*H*), 7.26 – 7.15 (m, 6H, Ar*H*), 4.30 – 4.24 (m, 1H, ArC*H*), 3.83 – 3.67 (m, 1H, $CH_2CHClCH_3$), 2.41 – 2.32 (m, 2H, $CH_2CHClCH_3$), 1.45-1.44 (m, 3H, $CH_2CHClCH_3$

– Signals for both diastereomer at the same chemical shift). **^{13}C NMR** (101 MHz, $CDCl_3$) for both diastereomer δ 143.9, 143.0, 142.9, 142.0, 132.5, 132.3, 129.6, 129.1, 129.0, 129.0, 128.8, 128.8, 128.2, 127.7, 127.0, 126.7, 56.6, 56.6, 47.8, 47.8, 46.1, 46.1, 25.9, 25.8. **IR** (v_{max}, cm^{-1}) 3028 (w), 2973 (w), 2929 (w), 1598 (w), 1490 (s), 1451 (m), 1092 (s), 1016 (m), 821 (m), 752 (s). **HRMS** (APPI/LTQ-Orbitrap) m/z: $[M]^+$ Calcd for $C_{16}H_{16}Cl_2^+$ 278.0624; Found 278.0626.

7. Sequence of chlorination/dielectrophilic substitution

4,4'-(1-Phenylpropane-1,3-diyldisulfonyl)bis(methylbenzene) (17a)

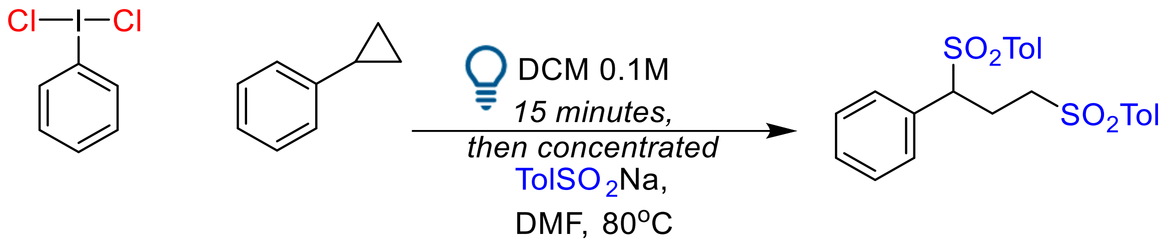

In a 4 mL Vial, **6a** (82 mg, 0.30 mmol, 1.5 equiv) was added. DCM (2.0 mL, 0.1 M) was added, followed by the addition of cyclopropane **8a** (23.6 mg, 0.200 mmol, 1.00 equiv). The vial was flushed with nitrogen for 10 seconds before sealed with a cap. The reaction mixture was stirred at room temperature under irradiation of Evolution chem 30 W LED (I_{max} = 450 nm) for 15 minutes. After irradiation, the reaction mixture was concentrated under reduced pressure to remove solvent, then to this vial is added DMF (2.0 mL, 0.10 M) and sodium p-toluenesulfinate (143 mg, 0.800 mmol, 4.00 equiv). The mixture was stirred at 80 °C for 16 h. The reaction was quenched by the addition of 1.5 mL 0.5 M aqueous LiCl and is washed 3x1.5 mL ethyl acetate. The organic extracts are combined, and the solvent is removed under reduced pressure. Purification by chromatography biotage on silica gel, eluting with 5-20% ethyl acetate in heptane, gave **17a** (49.1 mg, 0.115 mmol, 57 %) as a colorless oil. **Rf** = 0.48 (SiO_2, EtOAc/Heptane 1/1). **^{1}H NMR** (500 MHz, $CDCl_3$) δ 7.76 – 7.64 (m, 2H, Ar*H*), 7.41 – 7.33 (m, 4H, Ar*H*), 7.34 – 7.27 (m, 1H, Ar*H*), 7.26 – 7.20 (m, 2H, Ar*H*), 7.19 – 7.15 (m, 2H, Ar*H*), 7.06 – 6.96 (m, 2H, Ar*H*), 4.24 (dd, *J* = 10.8, 4.8 Hz, 1H, ArC*H*$_2$S), 3.06 – 2.92 (m, 2H, SC*H*$_2$), 2.81 – 2.65 (m, 1H, ArCHC*H*$_2$), 2.57 – 2.47 (m, 1H, ArCHC*H*$_2$), 2.47 (s, 3H, ArC*H*$_3$), 2.40 (s, 3H, ArC*H*$_3$). **^{13}C NMR** (126 MHz, $CDCl_3$) δ 145.3, 145.0, 135.5, 133.9, 131.2, 130.2, 129.9, 129.6, 129.4, 129.2, 128.9, 128.3, 69.0, 53.2, 22.2, 21.8, 21.8. **HRMS** (APCI/QTOF) m/z: $[M + Na]^+$ Calcd for $C_{23}H_{24}NaO_4S_2^+$ 451.1008; Found 451.1010.

(1,3-Diazidopropyl)benzene (17b)

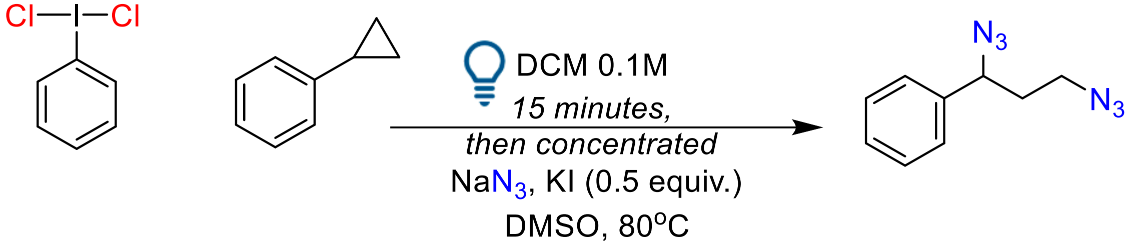

In a 4 mL Vial, dichloro(phenyl)-I3-iodane (**6a**) (82 mg, 0.30 mmol, 1.5 equiv) was added. DCM (2.0 mL, 0.1 M) was added, followed by the addition of cyclopropane **8a** (23.6 mg, 0.200 mmol, 1.00 equiv). The vial was flushed with nitrogen for 10 seconds before sealed with a cap. The reaction mixture was stirred at room temperature under irradiation of Evolution chem 30 W LED (l_{max} = 450 nm) for 15 minutes. After irradiation, the reaction mixture was concentrated under reduced pressure to remove solvent, then to this vial is added sodium azide (52.0 mg, 0.800 mmol, 4.00 equiv) and potassium iodide (16.6 mg, 0.100 mmol, 0.500 equiv) in 2 mL DMSO was stirred at 80 °C in 18 hour. The reaction mixture was cooled to room temperature, and 10 mL Et_2O was added for extraction. The resulting solution was washed by water and brine, dried over Na2SO4. Following concentration in vacuo, the crude residue was subjected to flash column chromatography on silica gel (heptane) to yield **17b** (25.6 mg, 0.127 mmol, 63%) as a colorless oil. **Rf** = 0.31 (SiO_2, heptane). **^{1}H NMR** (500 MHz, $CDCl_3$) δ 7.46 – 7.38 (m, 2H, Ar*H*), 7.38 – 7.34 (m, 1H, Ar*H*), 7.35 – 7.30 (m, 2H, Ar*H*), 4.62 (dd, *J* = 8.7, 5.9 Hz, 1H, ArC*H*), 3.48 – 3.36 (m, 1H, CH_2N_3), 3.37 – 3.27 (m, 1H, CH_2N_3), 2.11 – 2.01 (m, 1H, ArCHCH_2), 2.00 – 1.90 (m, 1H, ArCHCH_2). **^{13}C NMR** (126 MHz, $CDCl_3$) δ 138.8, 129.2, 128.8, 127.0, 63.4, 48.3, 35.6. NMR data matches reported value.[22]

8. Sequence for C-H amination

1-(1-Azidoethyl)-4-bromobenzene (18a)

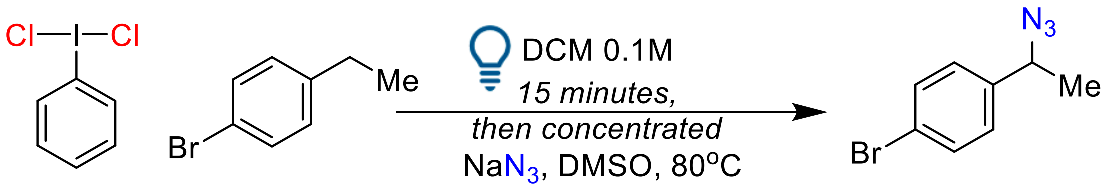

[22] Wang, M.-M.; Nguyen, T. V. T.; Waser, J. Diamine Synthesis via the Nitrogen-Directed Azidation of σ- and π-C–C Bonds. *J. Am. Chem. Soc.* **2021**, *143*, 11969–11975.

In a 4 mL Vial, dichloro(phenyl)-λ3-iodane (**6a**) (82 mg, 0.30 mmol, 1.5 equiv) was added. DCM (2.0 mL, 0.1 M) was added, followed by the addition of **12a** (37.0 mg, 0.200 mmol, 1.00 equiv). The vial was flushed with nitrogen for 10 seconds before sealed with a cap. The reaction mixture was stirred at room temperature under irradiation of Evolution chem 30 W LED (I_{max} = 450 nm) for 15 minutes. After irradiation, the reaction mixture was concentrated under reduced pressure to remove solvent, then to this vial is added sodium azide (20.0 mg, 0.300 mmol, 1.5 equiv) and 2.0 mL DMSO, the mixture was stirred at 80 °C in 18 hours. The reaction mixture was cooled to room temperature, and 10 mL Et_2O was added for extraction. The resulting solution was washed by water and brine, dried over Na_2SO_4. Following concentration in vacuo, the crude residue was subjected to flash column chromatography on silica gel (heptane) to yield **18a** (29.4 mg, 0.130 mmol, 65%) as a colorless liquid. **^{1}H NMR** (500 MHz, $CDCl_3$) δ 7.52 – 7.49 (m, 2H, Ar*H*), 7.24 – 7.19 (m, 2H, Ar*H*), 4.59 (q, *J* = 6.8 Hz, 1H, ArC*H*), 1.51 (d, *J* = 6.8 Hz, 3H, C*H₃*). **^{13}C NMR** (126 MHz, $CDCl_3$) δ 140.1, 132.1, 128.2, 122.1, 60.6, 21.7. NMR Data matches reported value.[23]

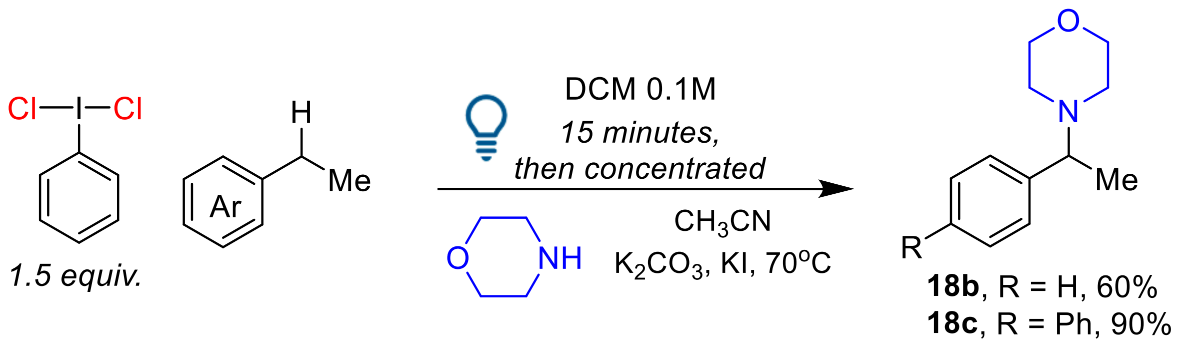

In a 4 mL Vial, **6a** (82 mg, 0.30 mmol, 1.5 equiv) was added. DCM (2.0 mL, 0.1 M) was added, followed by the addition of the starting material (0.20 mmol, 1.0 equiv). The vial was flushed with nitrogen for 10 seconds before sealed with a cap. The reaction mixture was stirred at room temperature under irradiation of Evolution chem 30 W LED (I_{max} = 450 nm) for 15 minutes. After irradiation, the reaction mixture was concentrated under reduced pressure to remove solvent, then to this vial was added morpholine (34.8 mg, 0.400 mmol, 2.00 equiv.), K_2CO_3 (31.3 mg, 0.500 mmol, 2.5 equiv) and potassium iodide (16.6 mg, 0.100 mmol, 0.5 equiv) in 2.0 mL CH_3CN, the mixture was stirred at 70 °C in 18 hour. The residue was filtered through silica plug and purified by flash column chromatography [DCM/MeOH 10:1] to afford **18b, c**.

N-(1-Phenylethyl)morpholine (18b)

[23] Yang, X.; Du, F.; Li, J.; Zhang, C. Late-Stage dehydroxyazidation of alcohols promoted by trifunctional hypervalent Azido-Iodine(III) reagents. *Chem. Eur. J.* **2022**, *28*, e202200272.

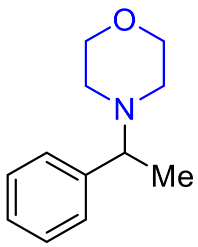

Starting from ethyl benzene (21.2 mg, 0.200 mmol, 1.00 equiv.). The residue was filtered through silica plug and purified by flash column chromatography (DCM/MeOH 10:1) to afford **18b** (22.9 mg, 0.120 mmol, 60%) as a colorless oil. TLC: R_f = 0.52 (DCM/MeOH 10:1). **^{1}H NMR** (400 MHz, $CDCl_3$) δ 7.29 – 7.23 (m, 4H, Ar*H*), 7.20 – 7.15 (m, 1H, Ar*H*), 3.68 – 3.56 (m, 4H, OC*H*$_2$), 3.29 – 3.17 (m, 1H, ArC*H*), 2.48 – 2.38 (m, 2H, NC*H*$_2$), 2.37 – 2.25 (m, 2H, NC*H*$_2$), 1.29 (d, *J* = 6.7 Hz, 3H, C*H*$_3$). **^{13}C NMR** (101 MHz, $CDCl_3$) δ 144.1, 128.4, 127.8, 127.1, 67.4, 65.6, 51.5, 20.0. NMR data matches reported value.[24]

4-(1-([1,1'-Biphenyl]-4-yl)ethyl)morpholine (18c)

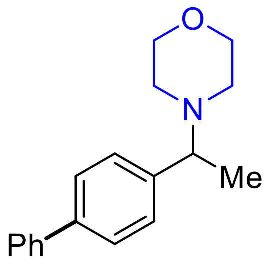

Starting from ethyl biphenyl (36.4 mg, 0.200 mmol, 1.00 equiv.). The residue was filtered through silica plug and purified by flash column chromatography (DCM/MeOH 10:1) to afford **18c** (48.1 mg, 0.180 mmol, 90%) as an pale-yellow oil. **TLC**: R_f = 0.52 (DCM/MeOH 10:1). **^{1}H NMR** (400 MHz, $CDCl_3$) δ 7.66 – 7.53 (m, 4H, Ar*H*), 7.47 – 7.32 (m, 5H, Ar*H*), 3.85 – 3.67 (m, 4H, OC*H*$_2$), 3.42 – 3.33 (m, 1H, ArC*H*N), 2.59 – 2.48 (m, 2H, NC*H*$_2$), 2.48 – 2.35 (m, 2H, NC*H*$_2$), 1.40 (d, *J* = 6.7 Hz, 3H, , C*H*$_3$). **^{13}C NMR** 101 MHz, $CDCl_3$) δ 143.1, 141.1, 140.1, 128.9, 128.2, 127.3, 127.2, 67.4, 65.2, 51.5, 19.9. 1 carbon is unresolved. NMR data matches reported value.[24]

[24] Murali, A.; Puppala, M.; Varghese, B.; Baskaran, S. A Lewis acid mediated Schmidt reaction of benzylic azide: synthesis of sterically crowded aromatic tertiary amines. *Eur. J. Org. Chem.* **2011**, 5297–5302.

9. Preliminary mechanistic study

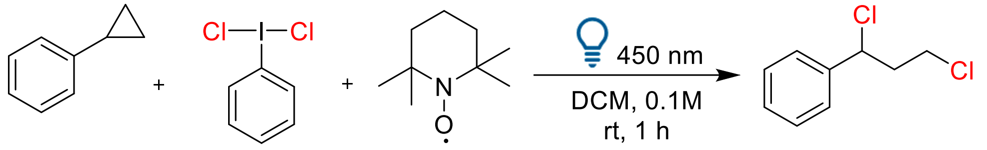

1,5 equiv. 1 equiv. not observed

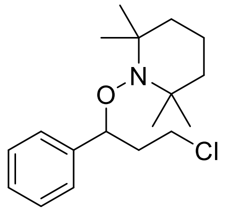

Cal: 310.1932. QTOF - ESI(+) : Found : 310.1933

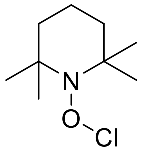

Cal: 192.1150. QTOF - ESI(+) : Found : 192.1132

In a 4 mL Vial, **6a** (82 mg, 0.30 mmol, 1.5 equiv), TEMPO (31.3 mg, 0.200 mmol, 1.00 equiv.), and DCM (2.0 mL, 0.1 M) were added, followed by the addition of cyclopropane **8a** (23.6 mg, 0.200 mmol, 1.00 equiv). The vial was flushed with nitrogen for 10 seconds before sealed with a cap. The reaction mixture was stirred at room temperature under irradiation of a Kessil lamp (I_{max} = 440 nm) for 15 minutes. The reaction was monitored by TLC, and no product was observed. The mixture was analysed by HRMS to identify the intermediates.

Proposed mechanism

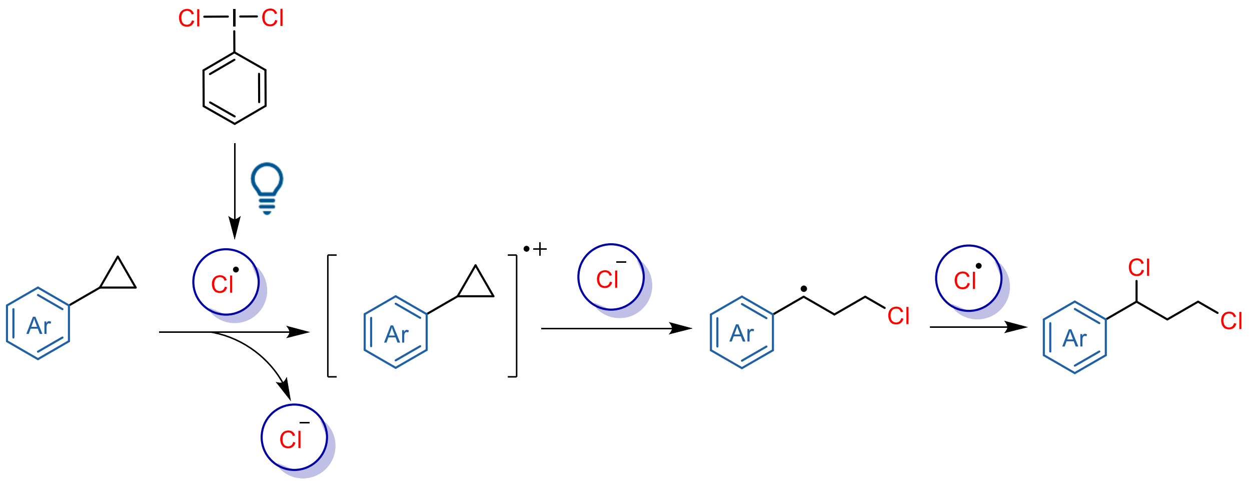

10. NMR spectrum.

1-Cyclopropyl-4-methoxybenzene (8b) ^{1}H NMR (400 MHz, $CDCl_3$)

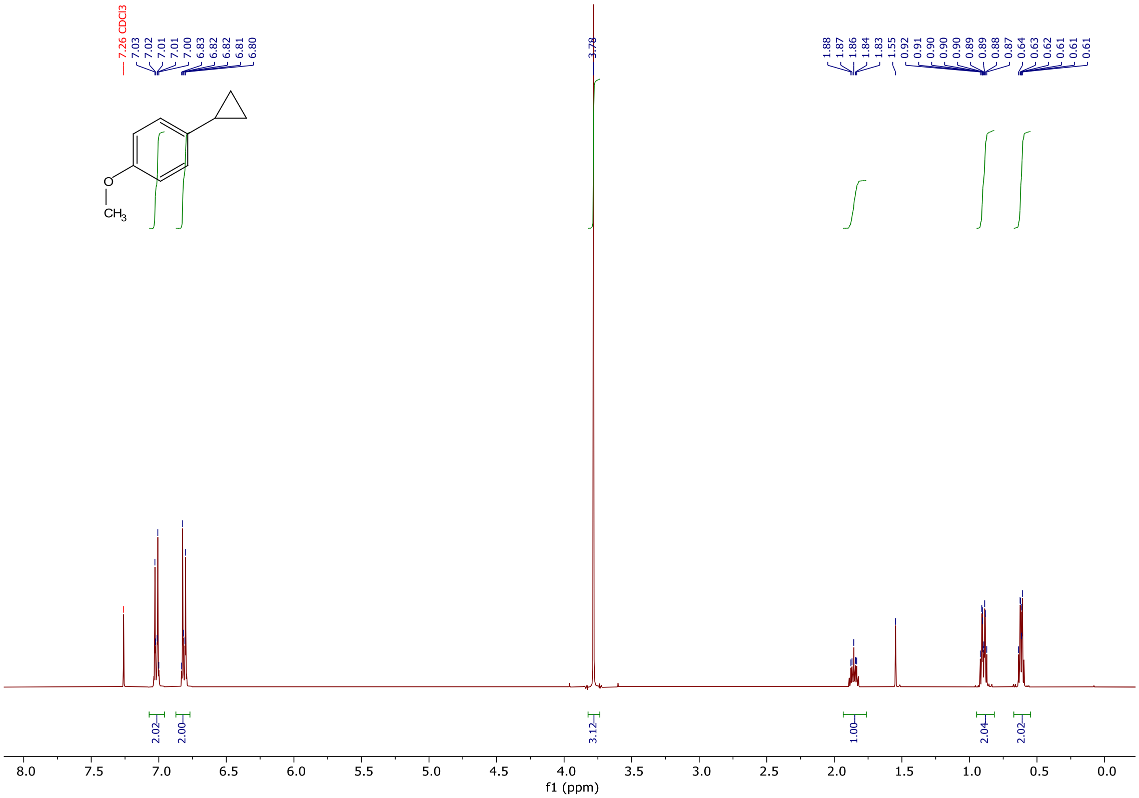

^{13}C NMR (101 MHz, $CDCl_3$)

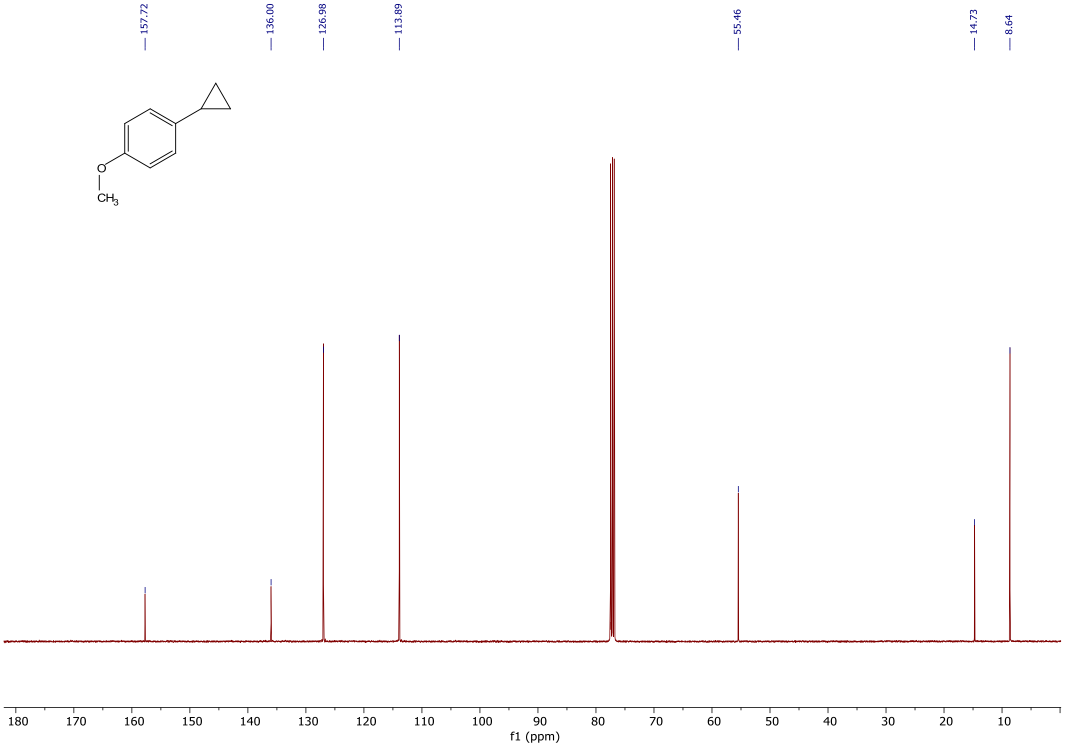

4-Cyclopropylphenyl acetate (8c) ^{1}**H NMR** (400 MHz, $CDCl_3$)

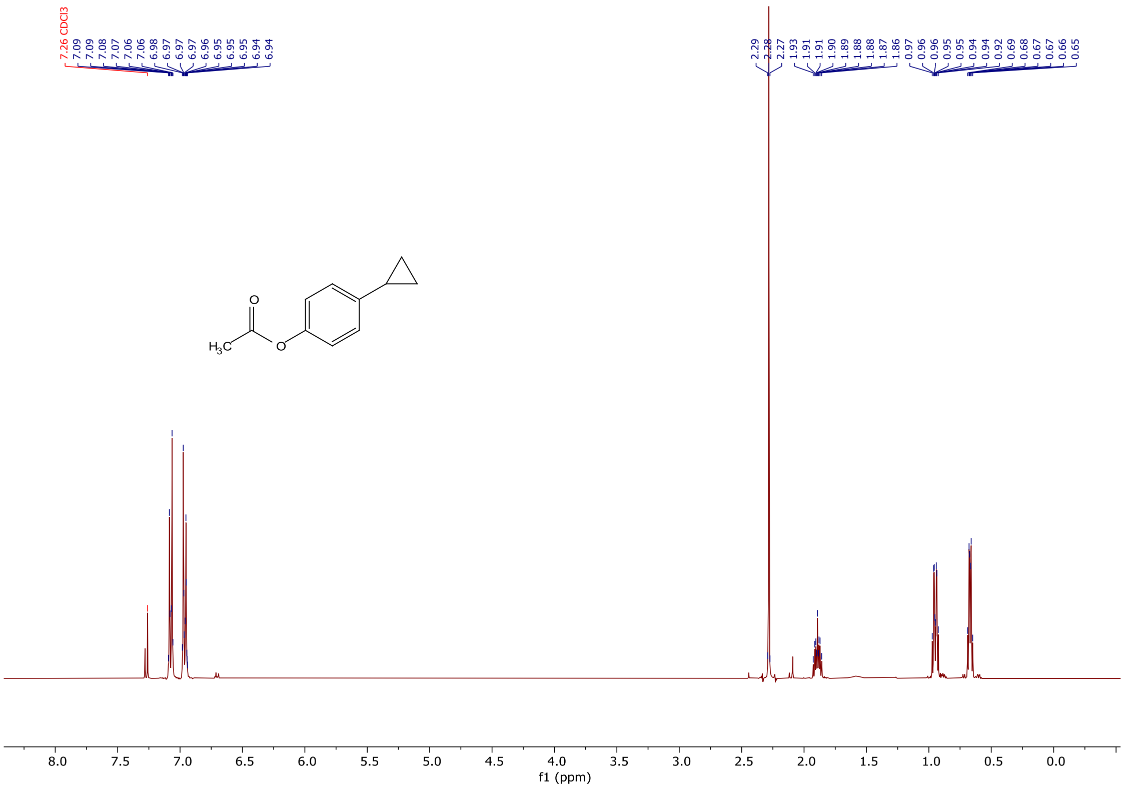

^{13}**C NMR** (101 MHz, $CDCl_3$)

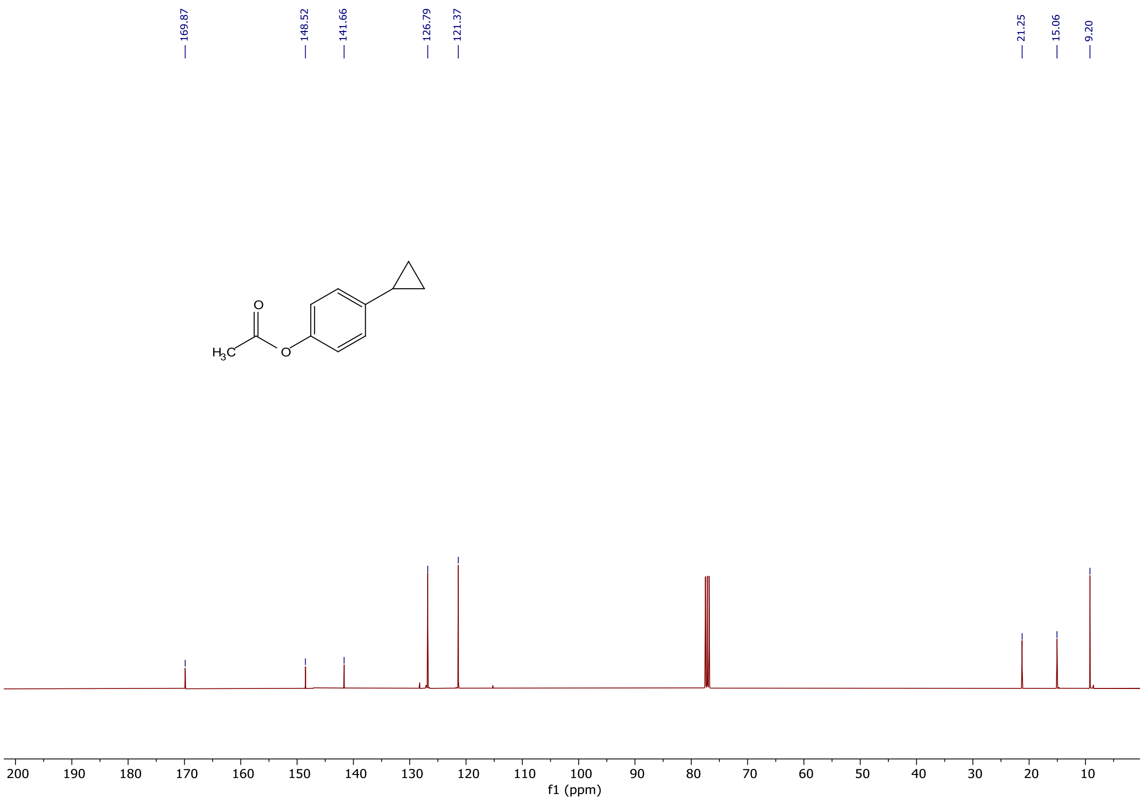

5-Cyclopropyl-2-methoxypyridine (8l) ^{1}H NMR (400 MHz, $CDCl_3$)

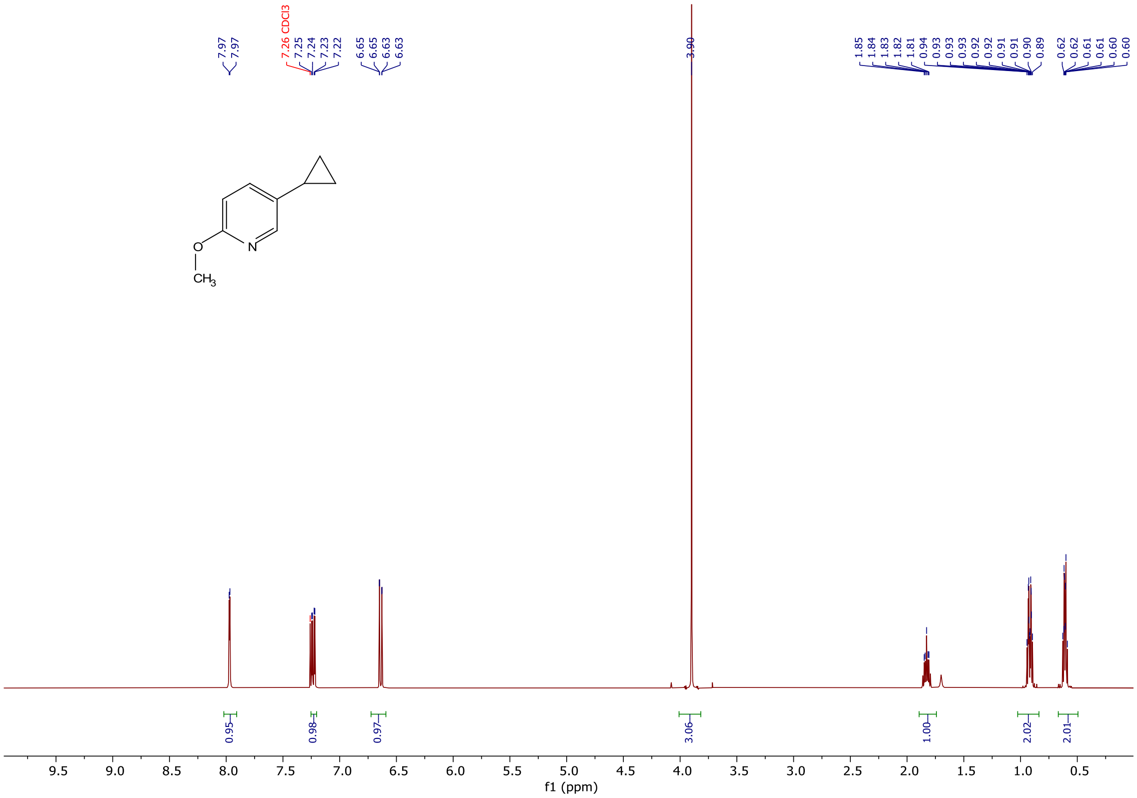

^{13}C NMR (101 MHz, $CDCl_3$)

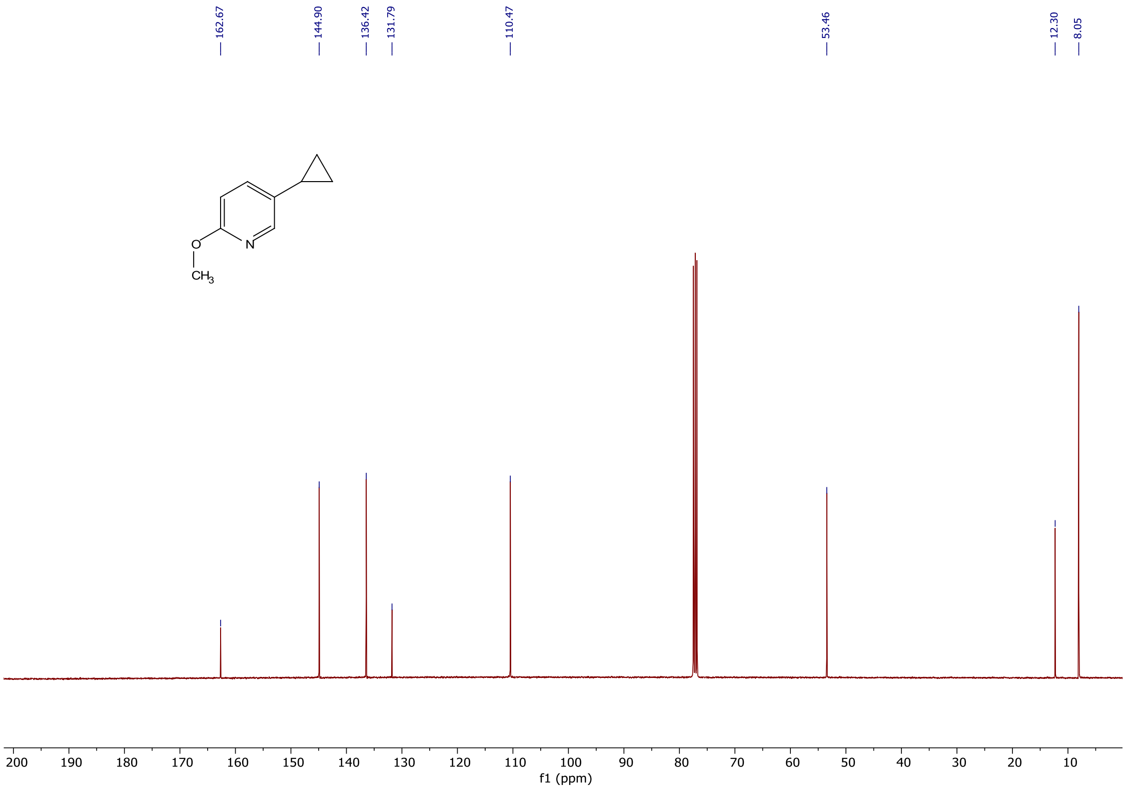

(1,3-Dichloropropyl)benzene (9a) **^{1}H NMR** (400 MHz, $CDCl_3$)

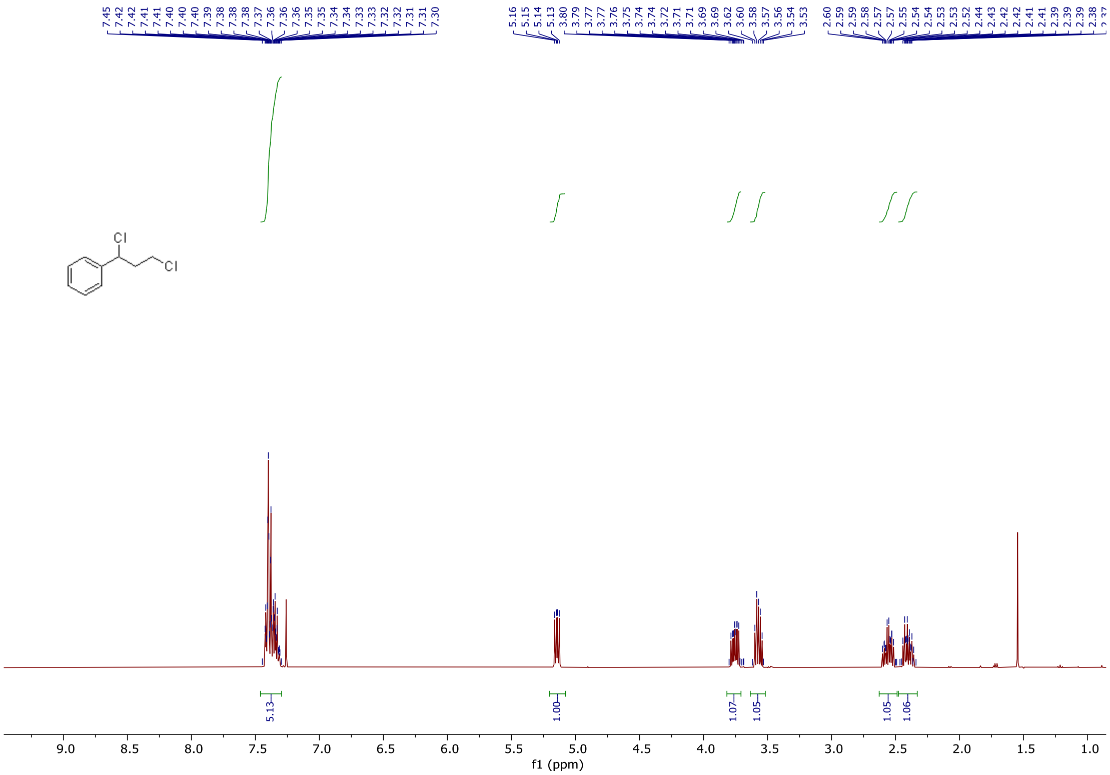

^{13}C NMR (101 MHz, $CDCl_3$)

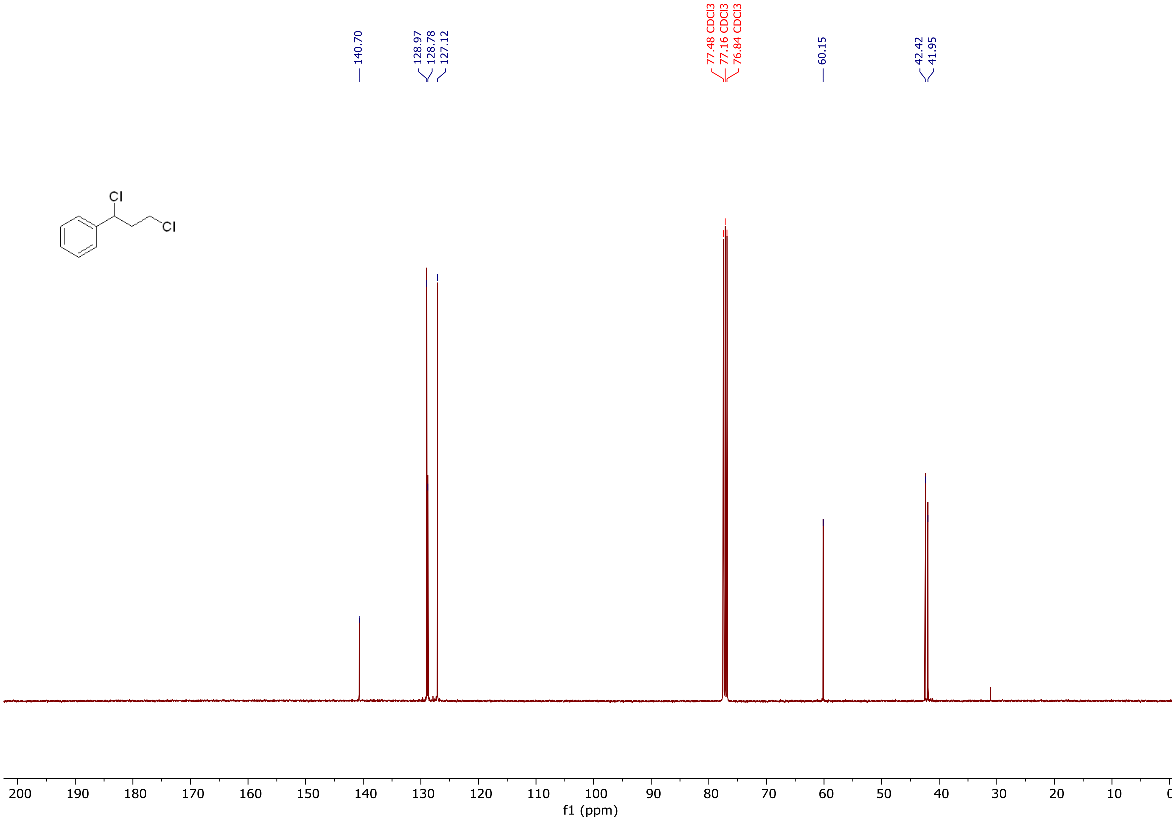

3-Chloro-1-(4-methoxyphenyl)propan-1-ol (9b) ^{1}H NMR (400 MHz, $CDCl_3$)

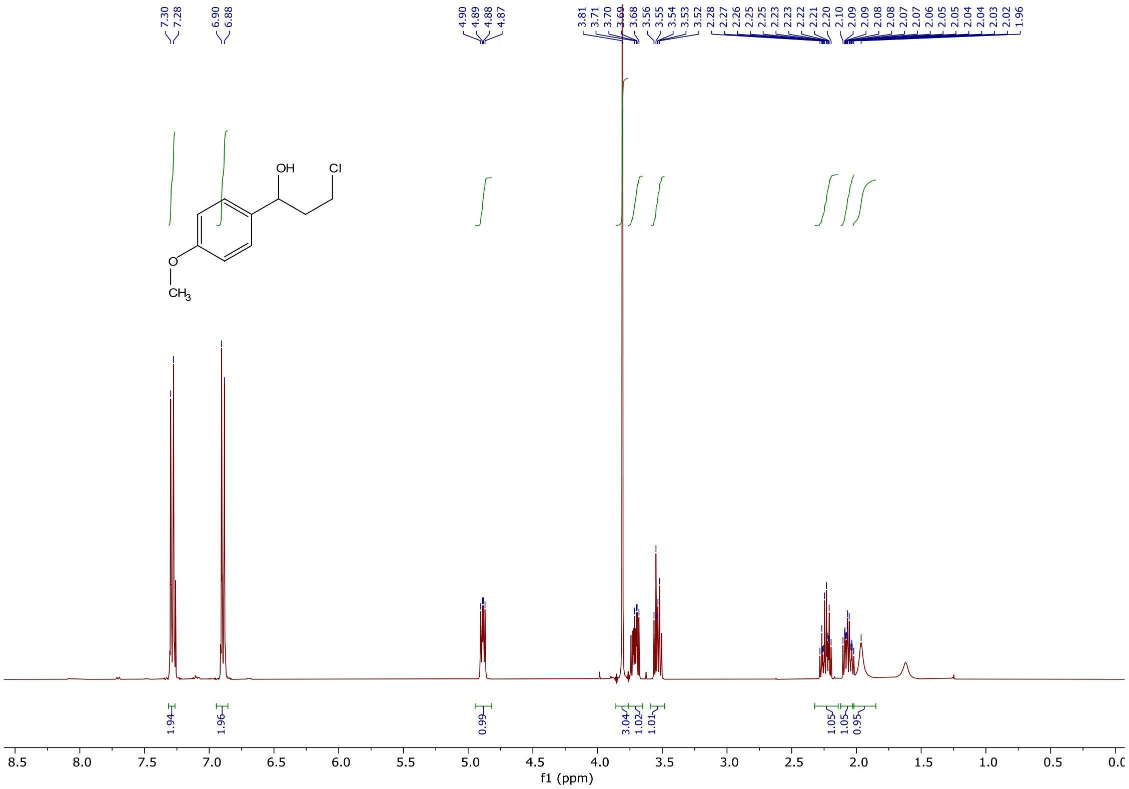

^{13}C NMR (101 MHz, $CDCl_3$)

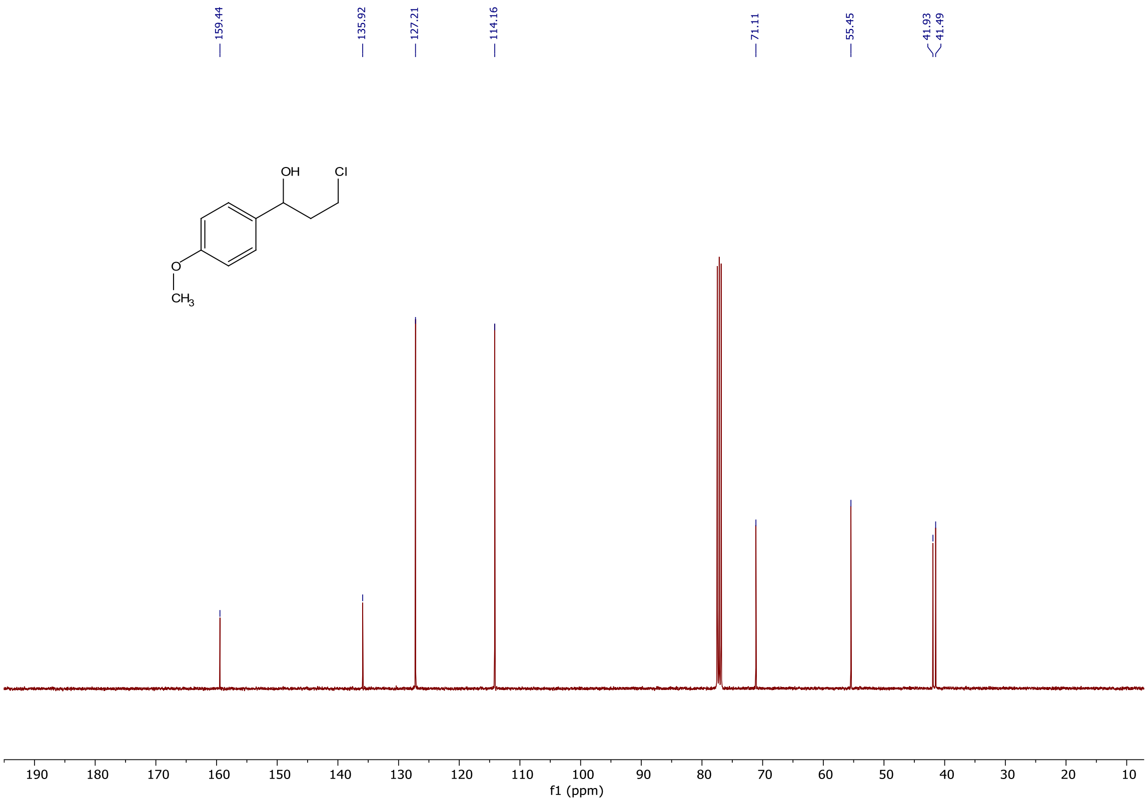

4-(1,3-Dichloropropyl)phenyl acetate (9c). ^{1}H NMR (400 MHz, $CDCl_3$)

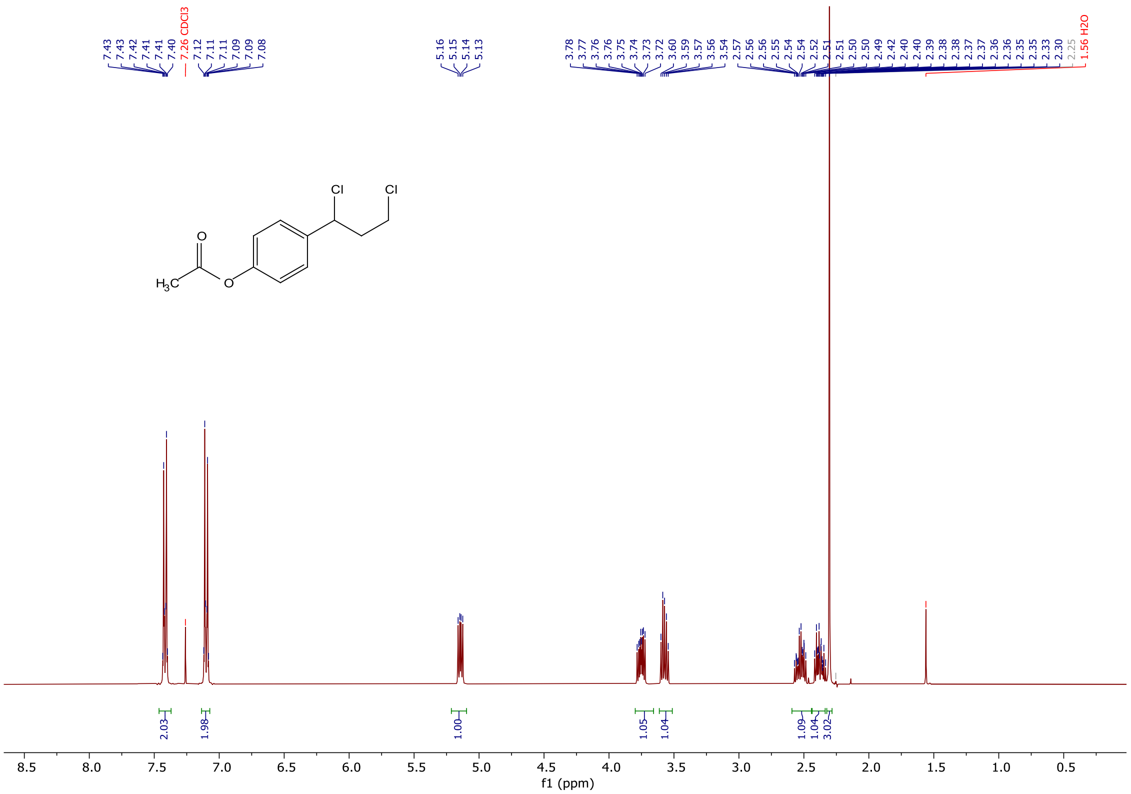

^{13}C NMR (101 MHz, $CDCl_3$)

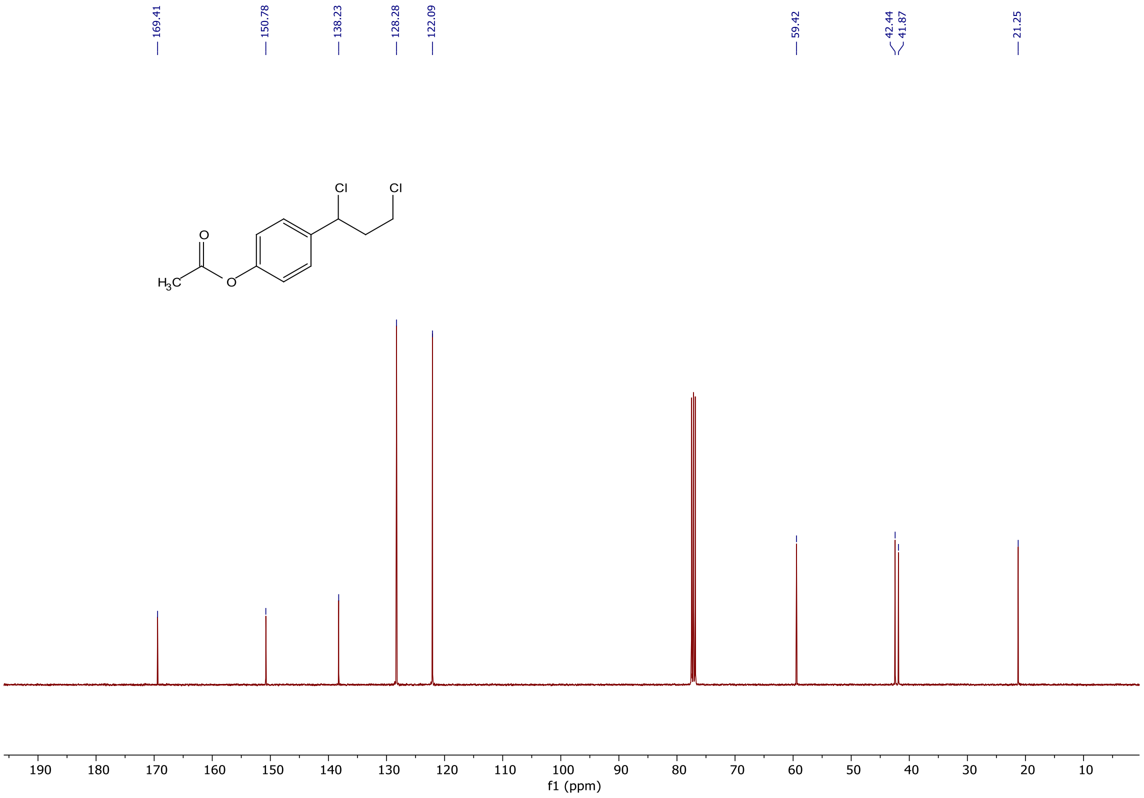

1-Bromo-4-(1,3-dichloropropyl)benzene (9d) ^{1}**H NMR** (500 MHz, $CDCl_3$)

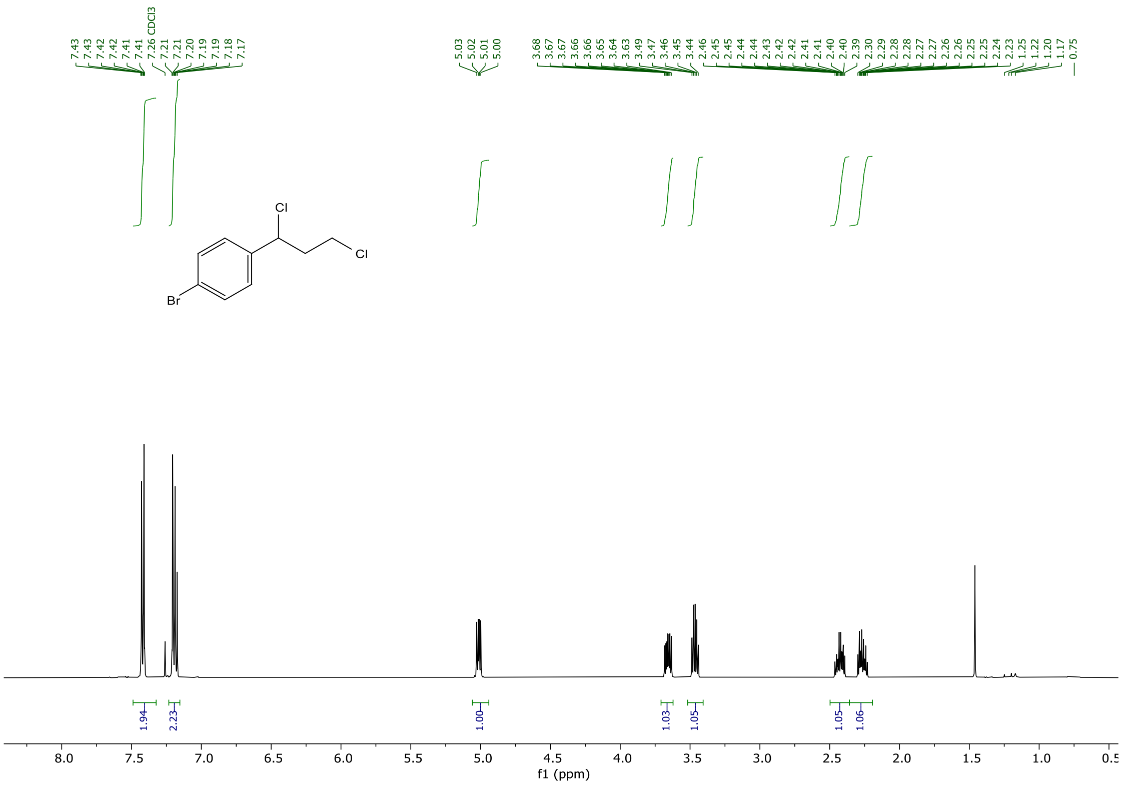

^{13}**C NMR** (126 MHz, $CDCl_3$)

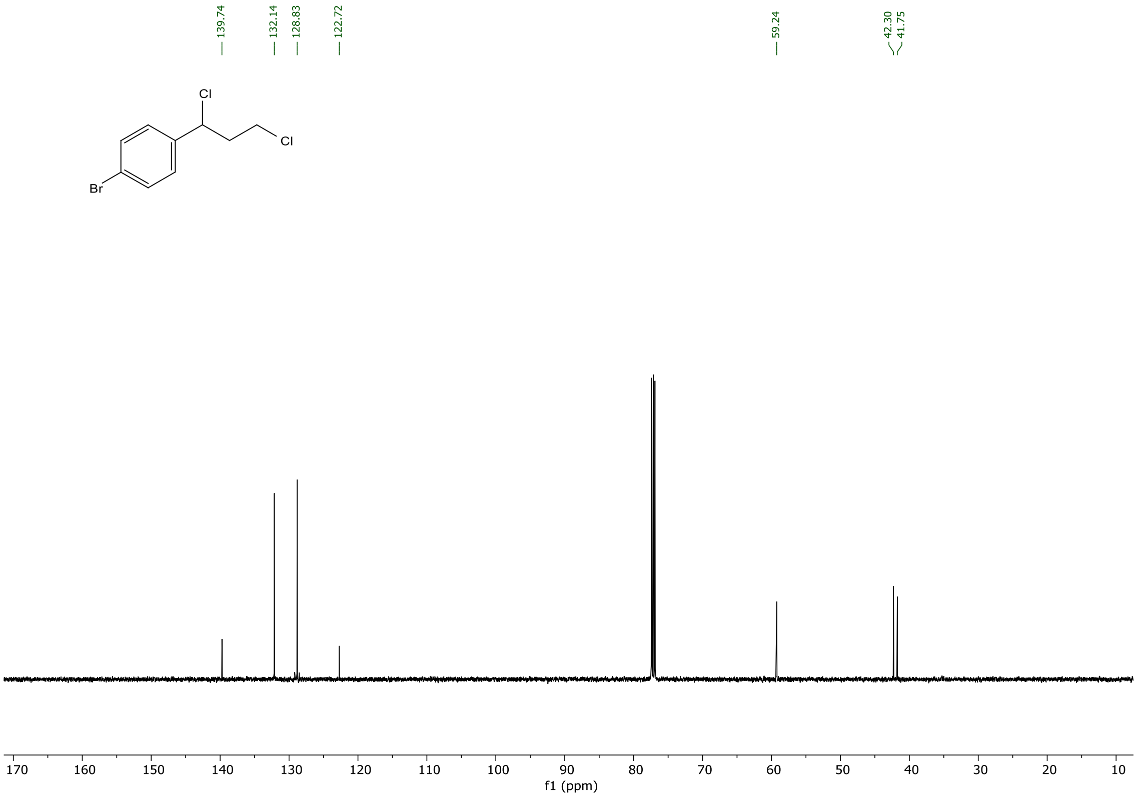

4-(1,3-Dichloropropyl)benzonitrile (9e). **^{1}H NMR** (500 MHz, $CDCl_3$)

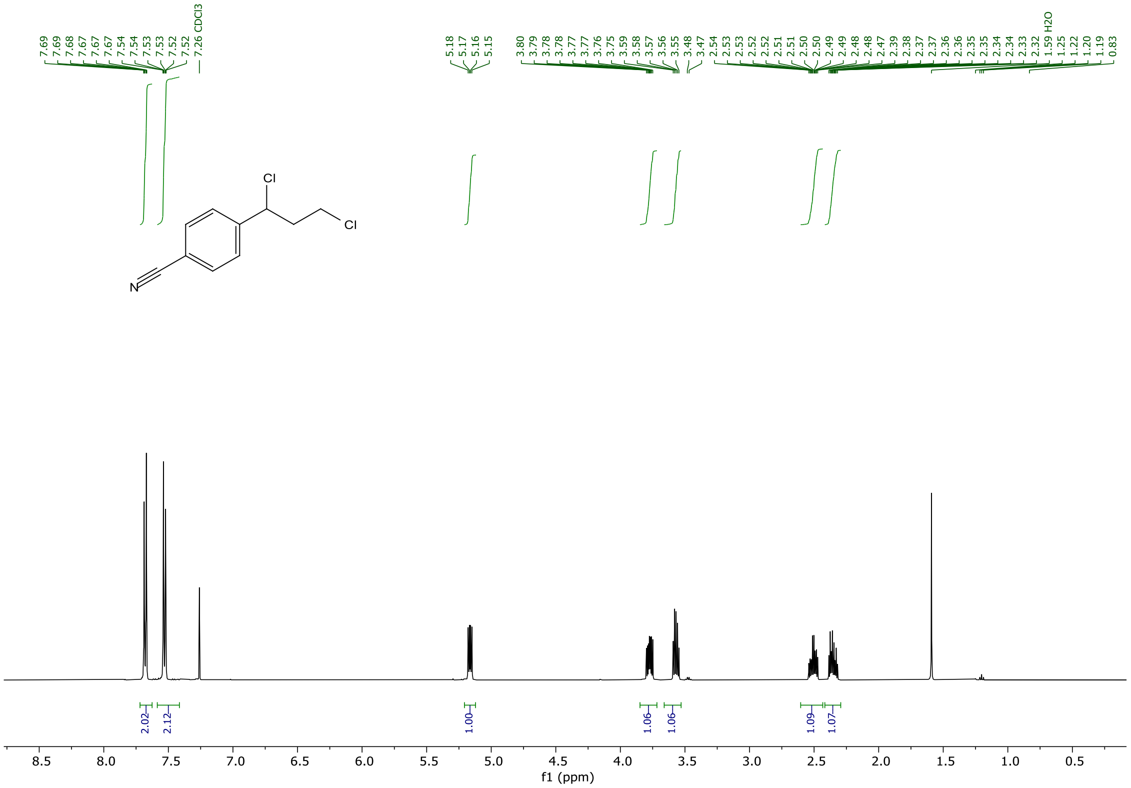

^{13}C NMR (126 MHz, $CDCl_3$)

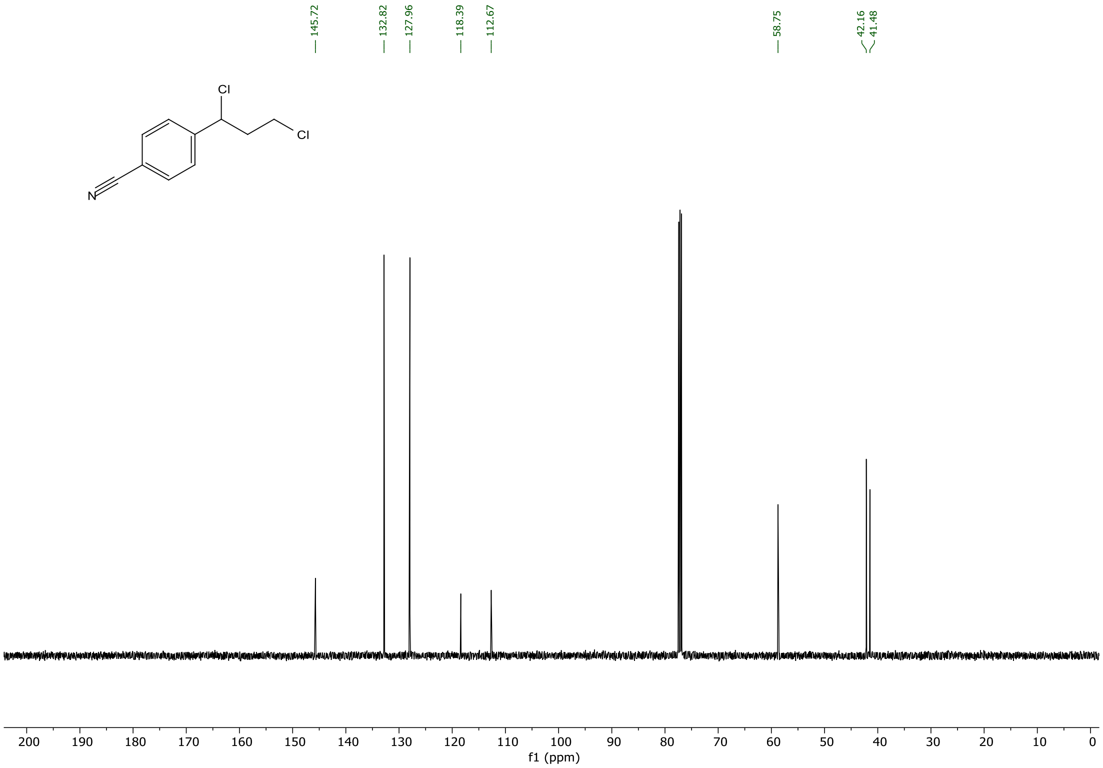

1-(4-(1,3-Dichloropropyl)phenyl)ethan-1-one (9f). **^{1}H NMR** (500 MHz, $CDCl_3$)

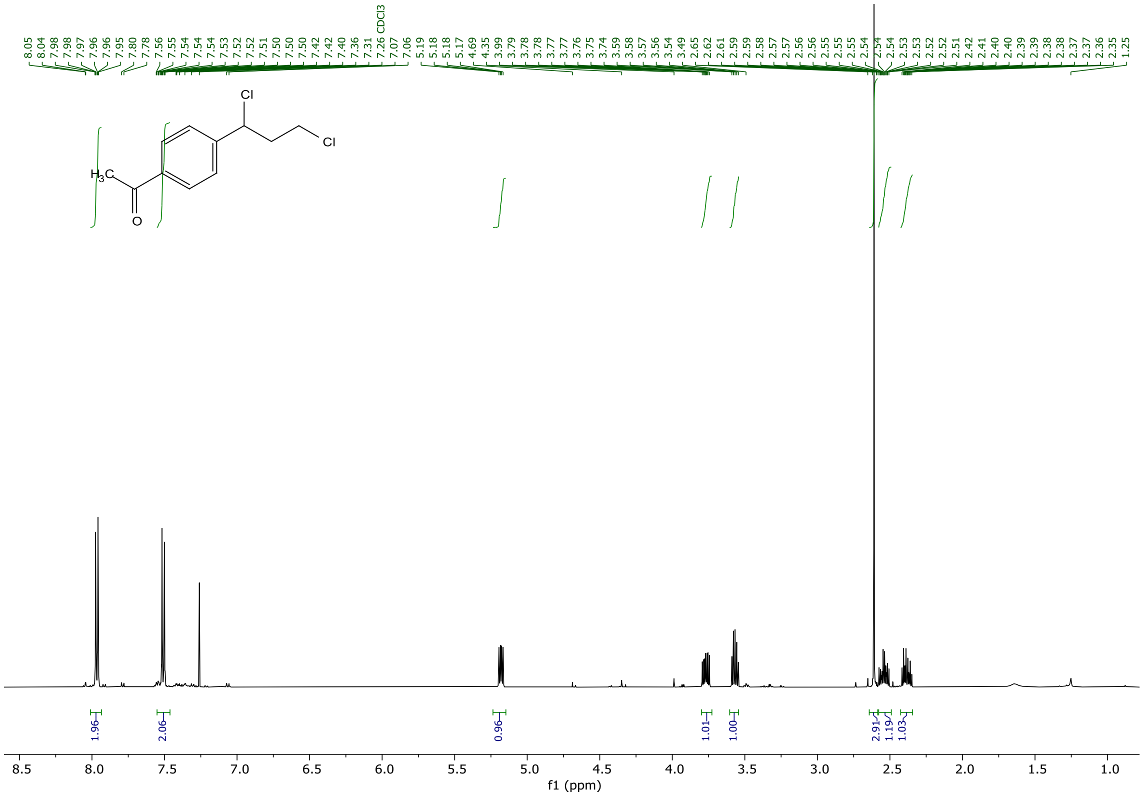

^{13}C NMR (126 MHz, $CDCl_3$)

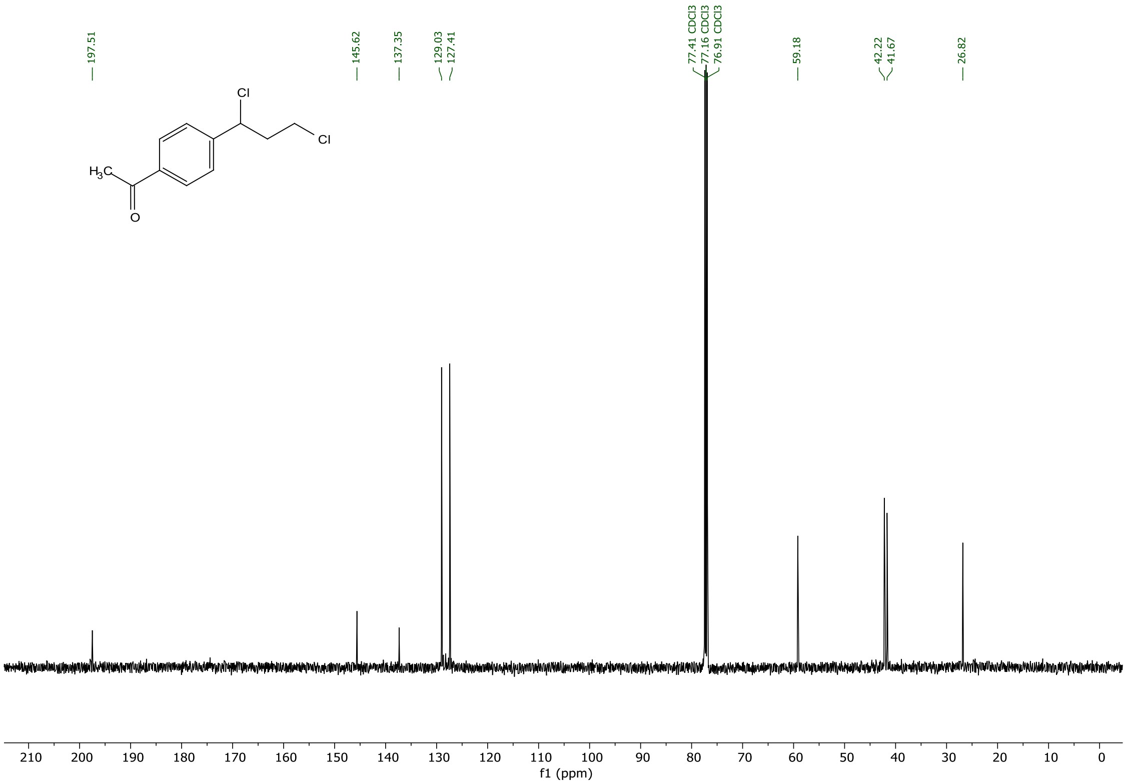

2-(4-(1,3-Dichloropropyl)phenyl)-4,4,5,5-tetramethyl-1,3,2-dioxaborolane (9g)

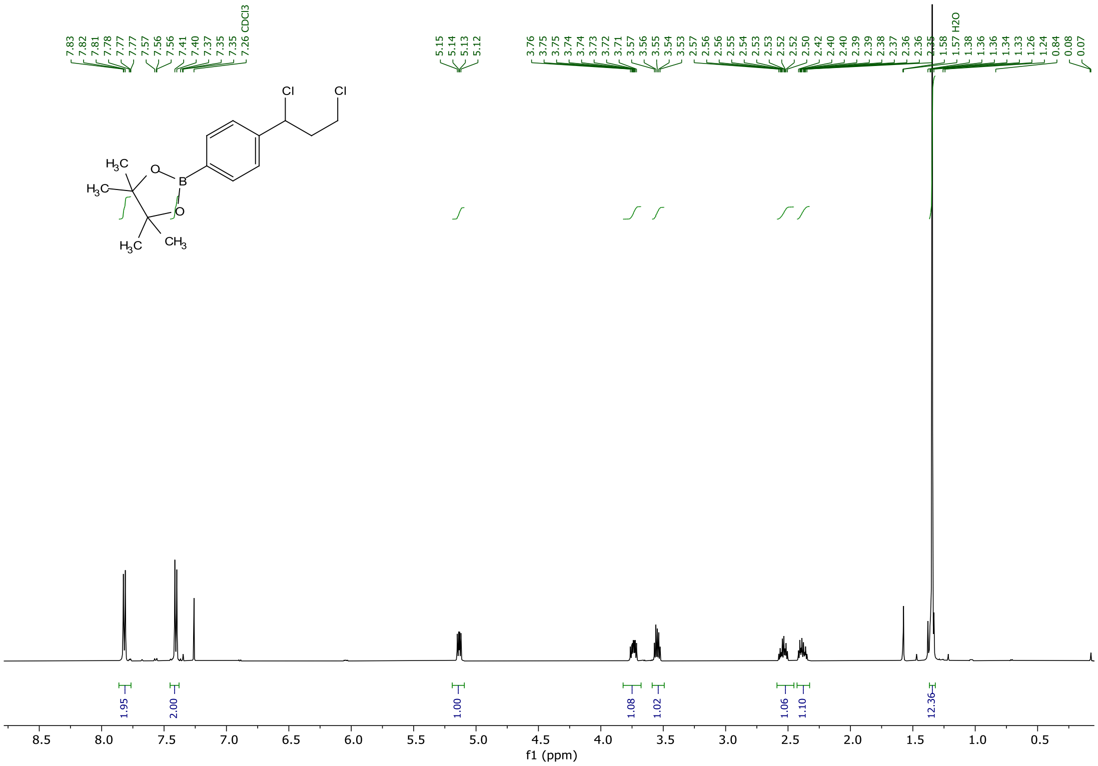

^{13}C NMR (126 MHz, $CDCl_3$)

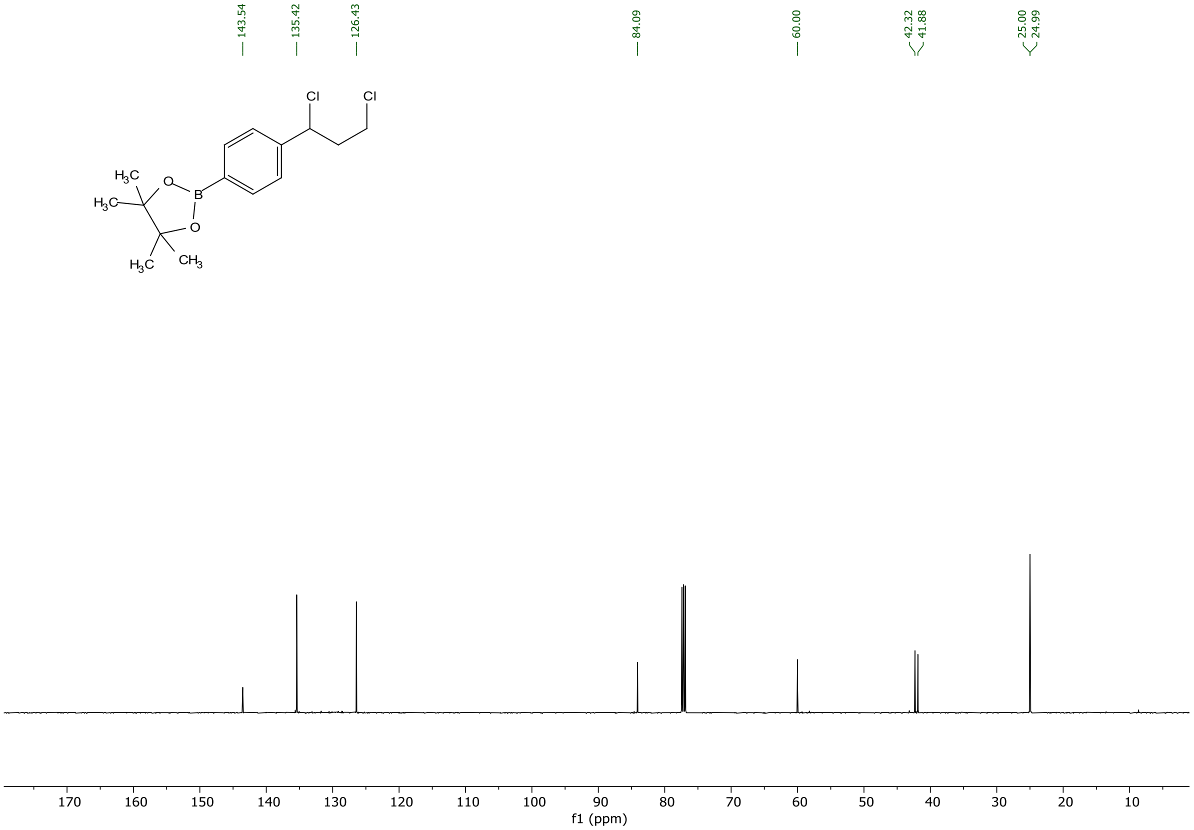

2-(1,3-Dichloropropyl)naphthalene (9h) ^{1}H NMR (500 MHz, $CDCl_3$)

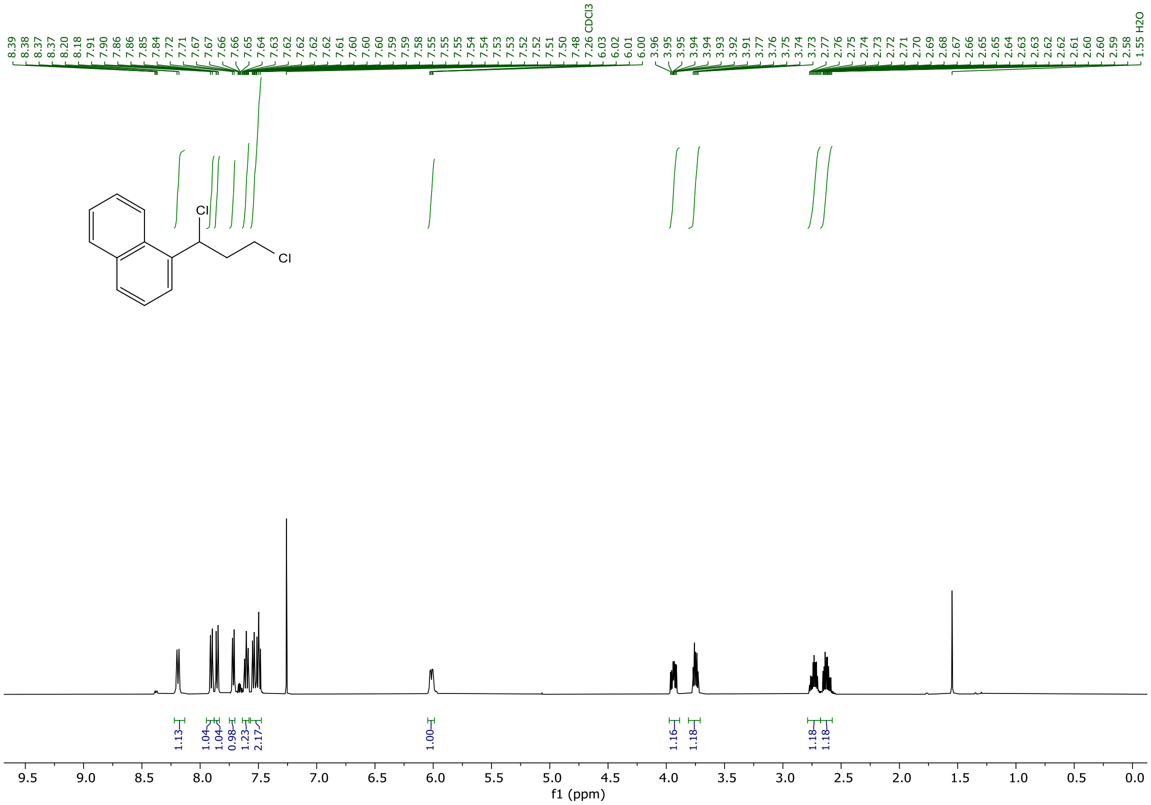

^{13}C NMR (126 MHz, $CDCl_3$)

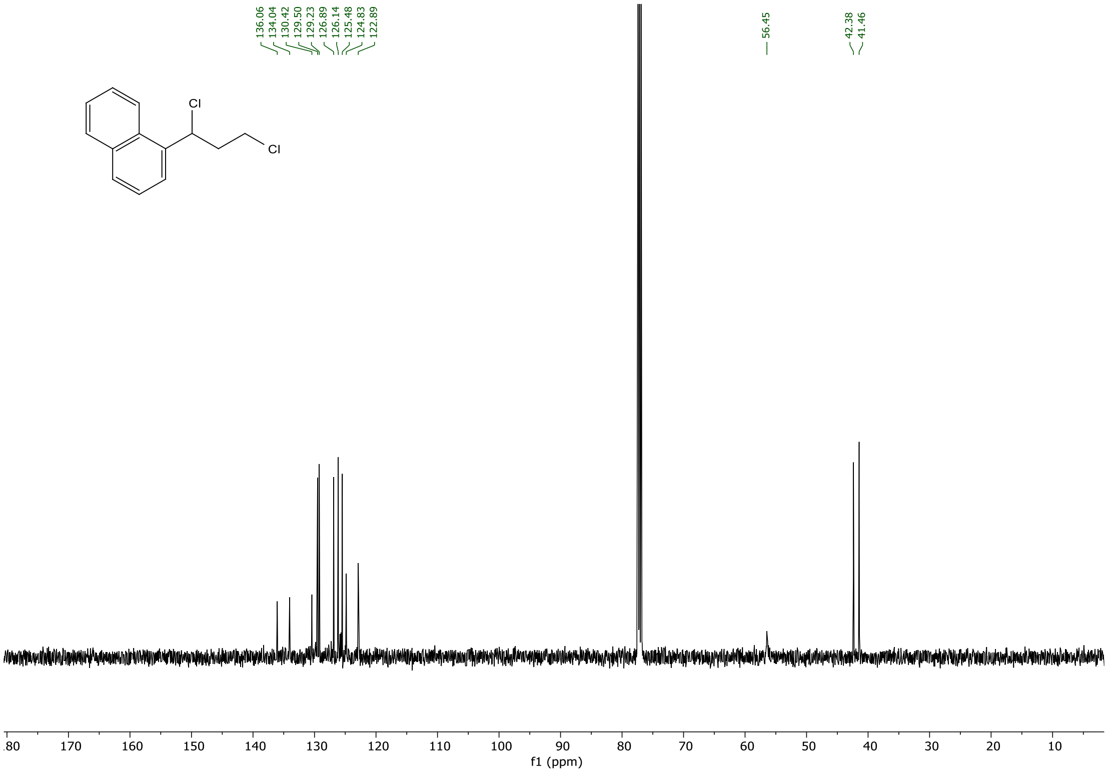

1,3-Dichloro-1,3-diphenylpropane (9i) ^{1}H NMR (500 MHz, $CDCl_3$). Mixture of 2 diastereomers

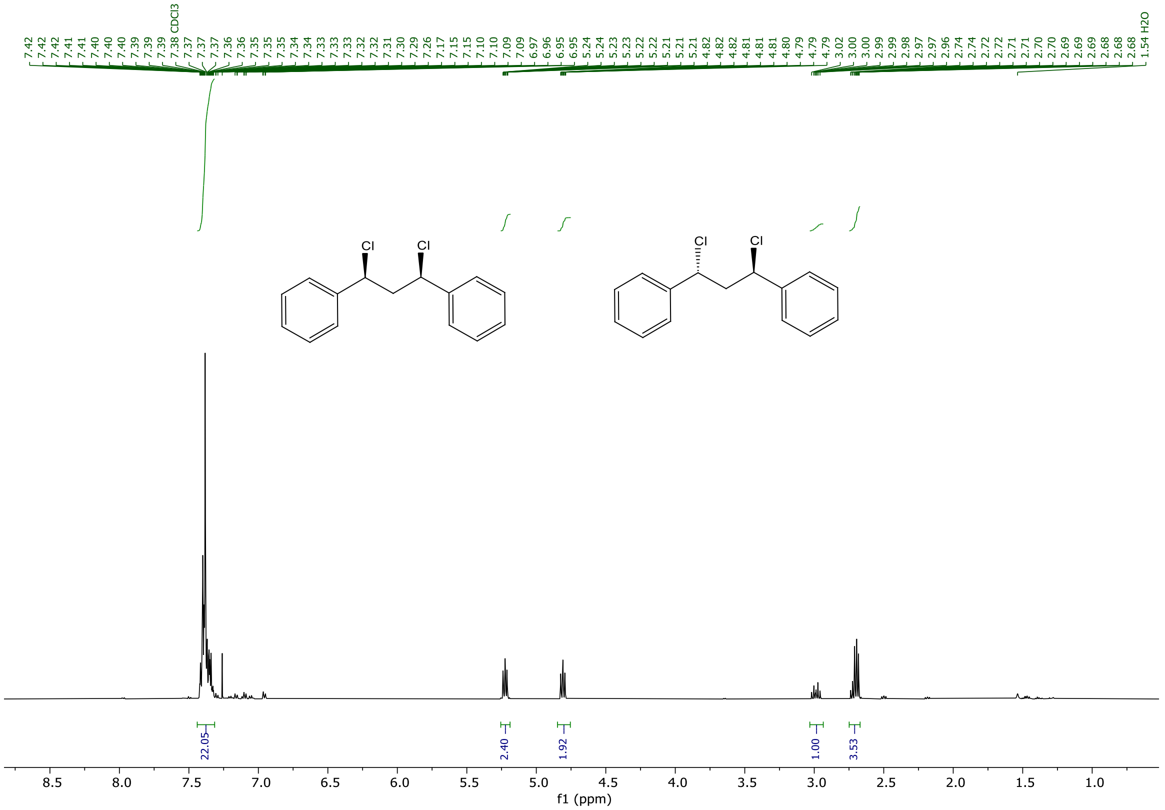

^{13}C NMR (126 MHz, $CDCl_3$)

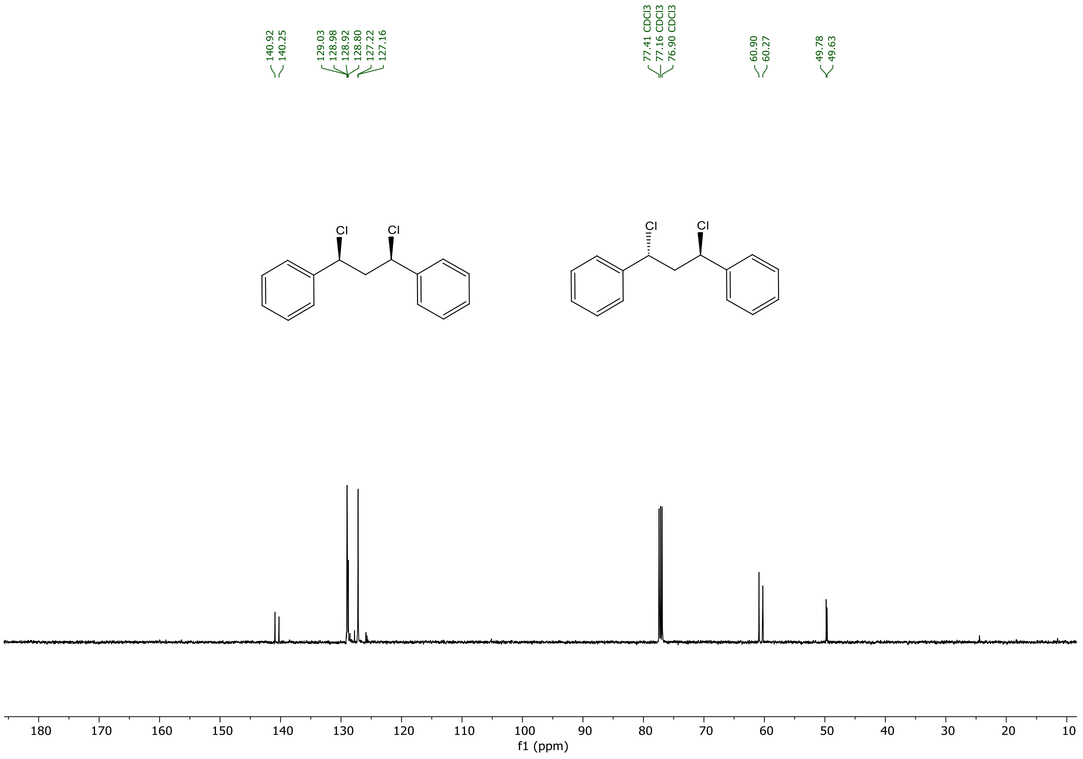

Dimethyl 2-chloro-2-(2-chloro-2-phenylethyl)malonate (9j) ^{1}H NMR (500 MHz, $CDCl_3$)

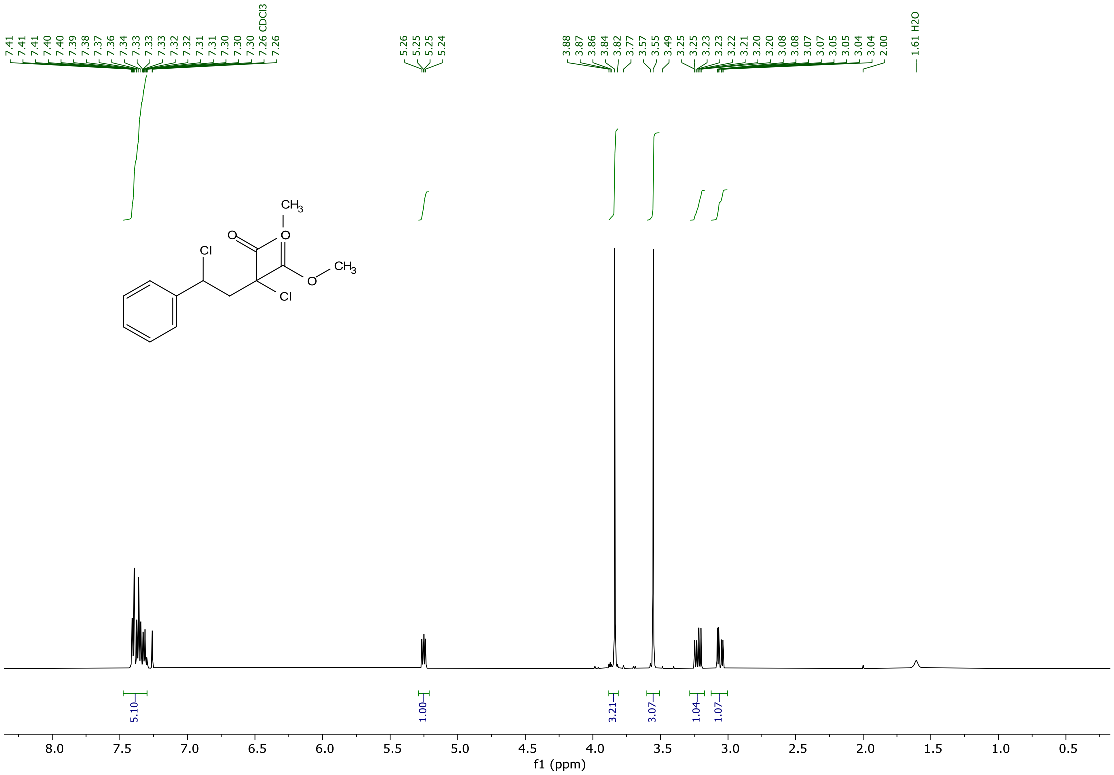

^{13}C NMR (126 MHz, $CDCl_3$)

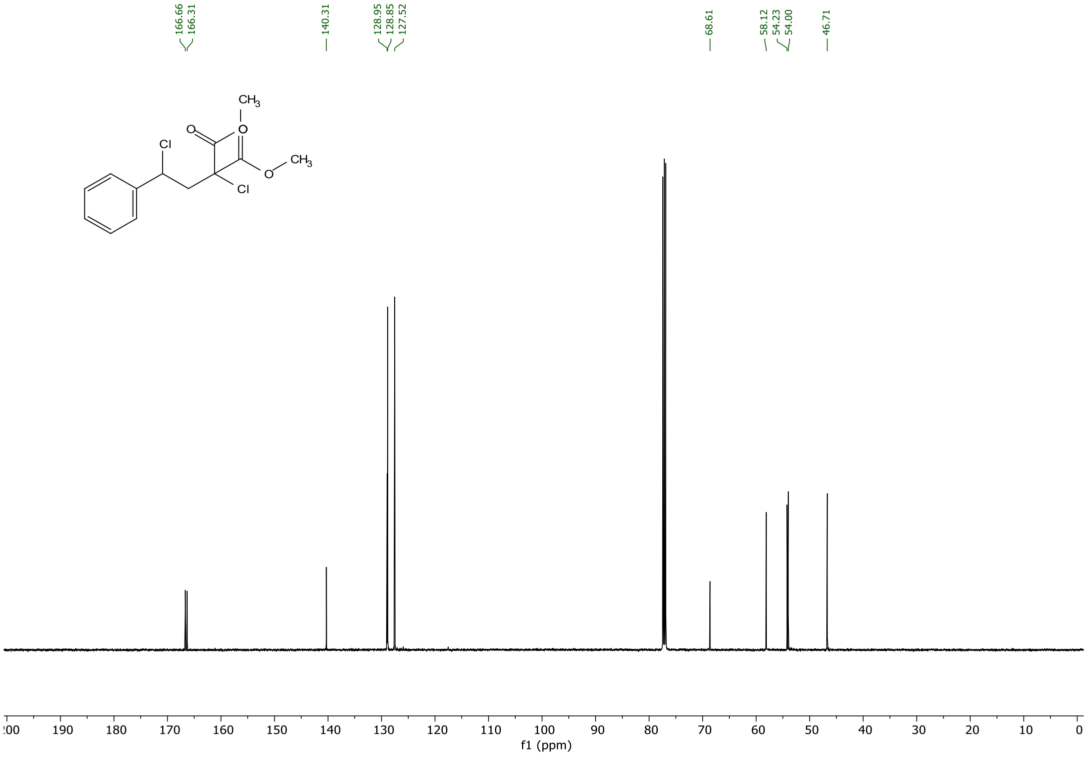

4-(1,3-Dichloropropyl)-2-methylbenzoic acid (9k) **^{1}H NMR** (500 MHz, CD_2Cl_2)

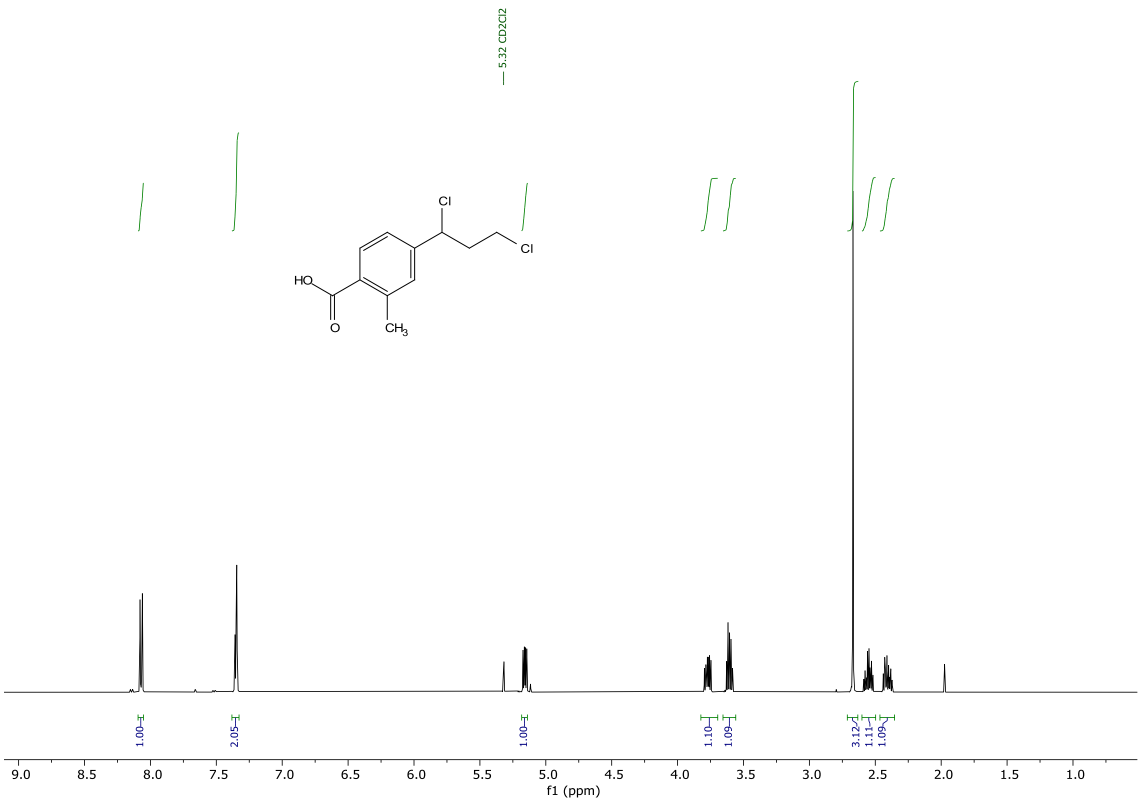

^{13}C NMR (126 MHz, CD_2Cl_2)

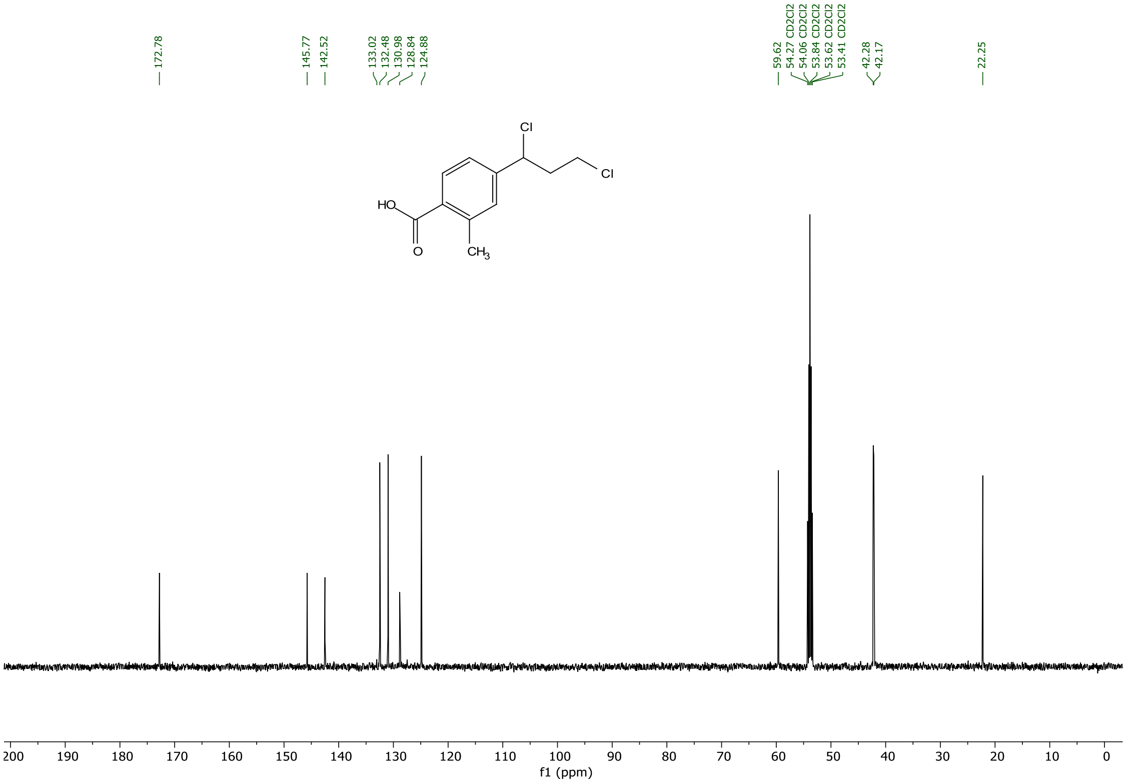

5-(1,3-Dichloropropyl)-2-methoxypyridine (9l) ^{1}H NMR (400 MHz, $CDCl_3$)

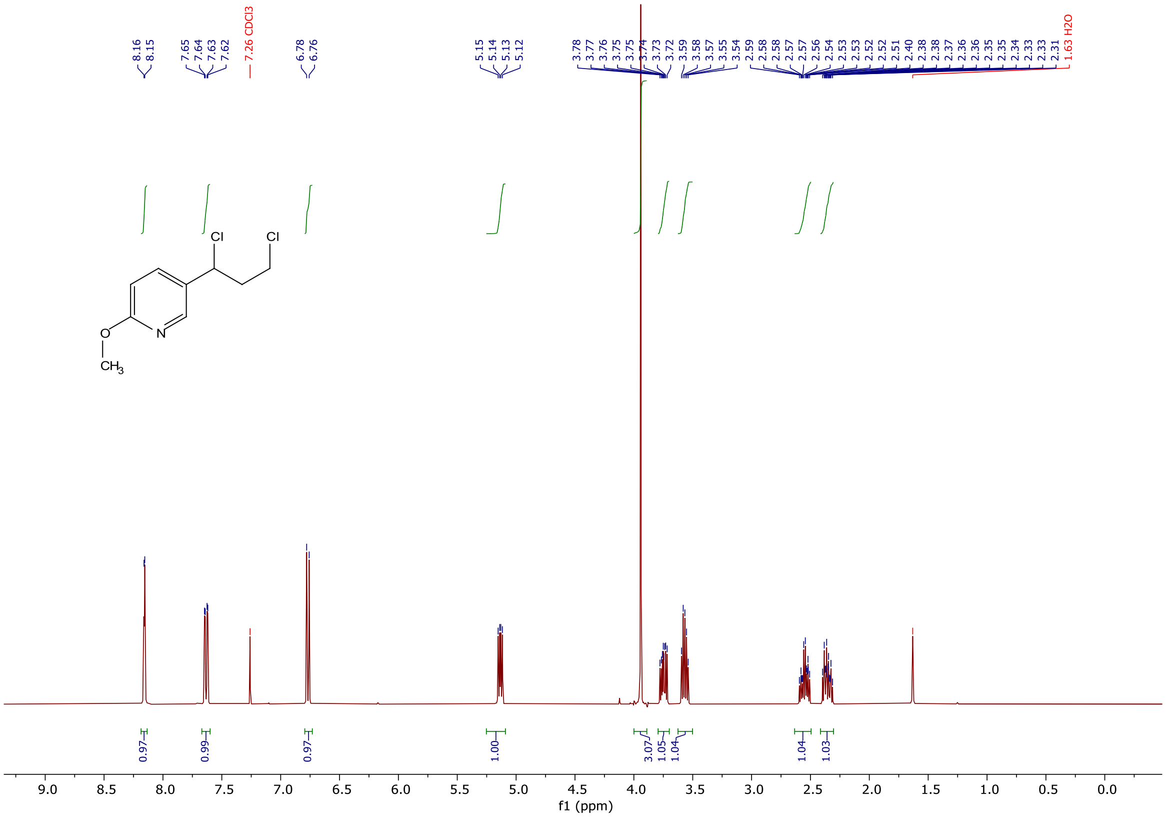

^{13}C NMR (101 MHz, $CDCl_3$)

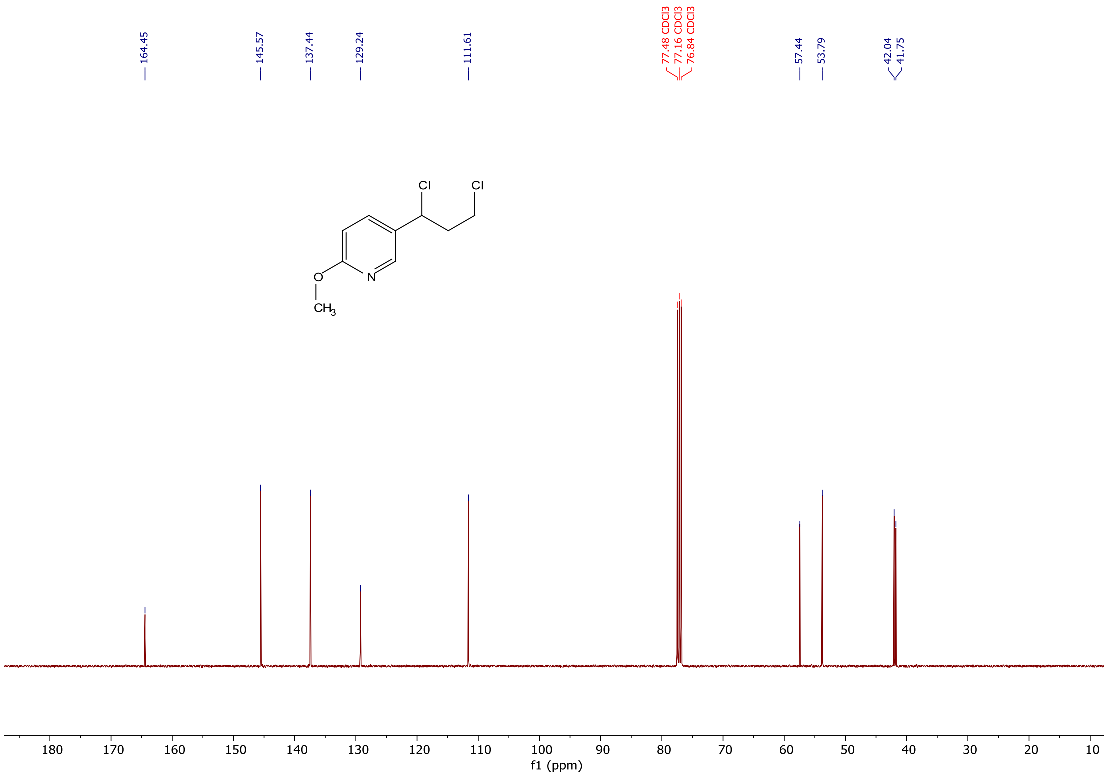

2-((5-Bromo-4-(4-(1,3-dichloropropyl)naphthalen-1-yl)-4H-1,2,4-triazol-3-yl)thio)acetic acid (9m). ^{1}H NMR (500 MHz, $CDCl_3$)

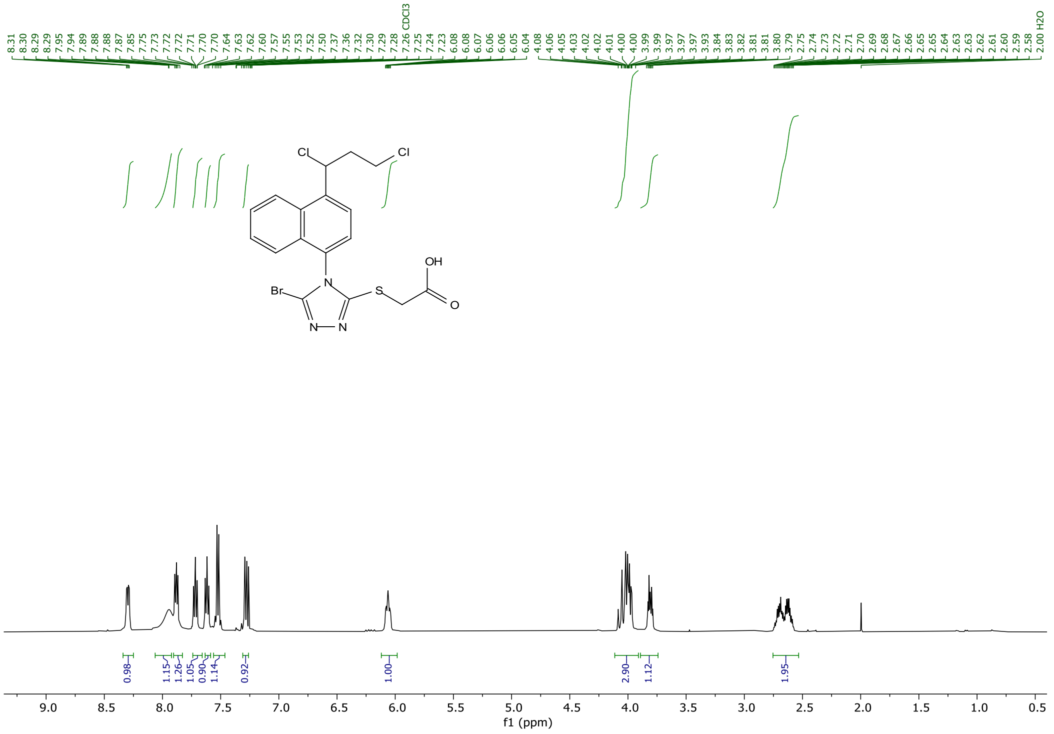

^{13}C NMR (126 MHz, $CDCl_3$)

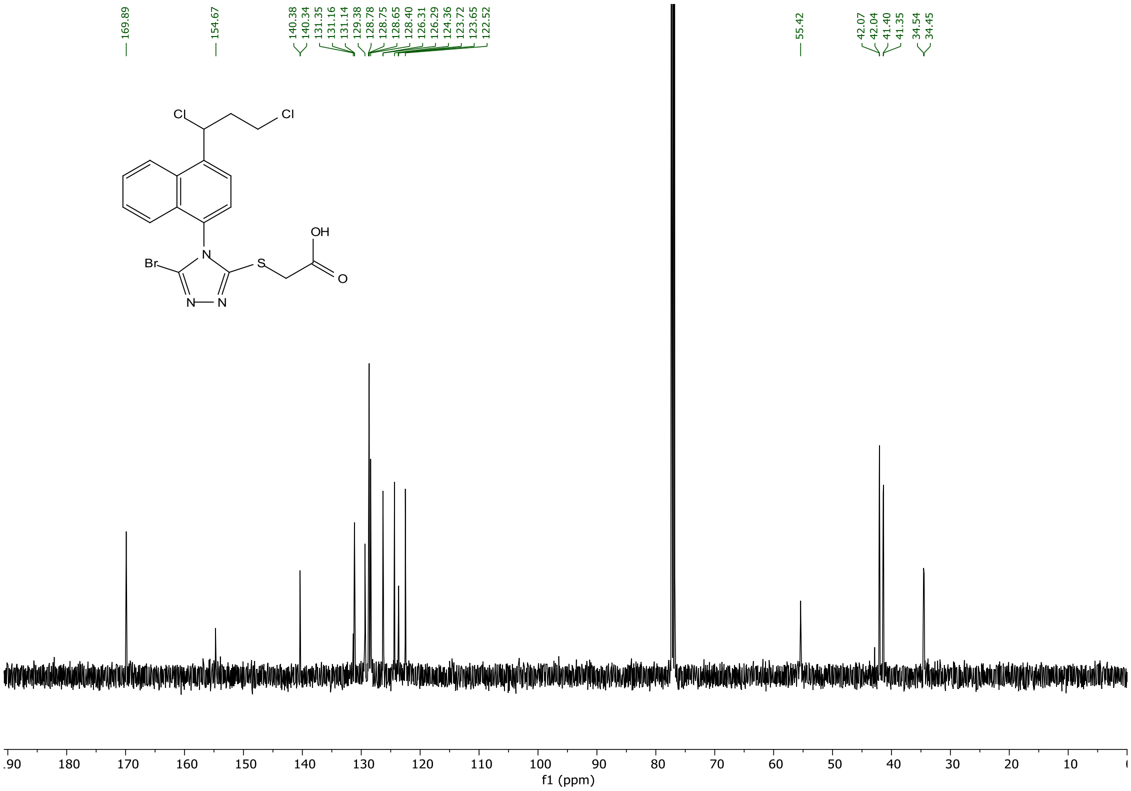

1-(2-Chloro-4-(1,3-dichloropropyl)phenyl)-5-methyl-1H-1,2,3-triazole-4-carboxylic acid (9n)

^{1}**H NMR** (500 MHz, d6-Acetone)

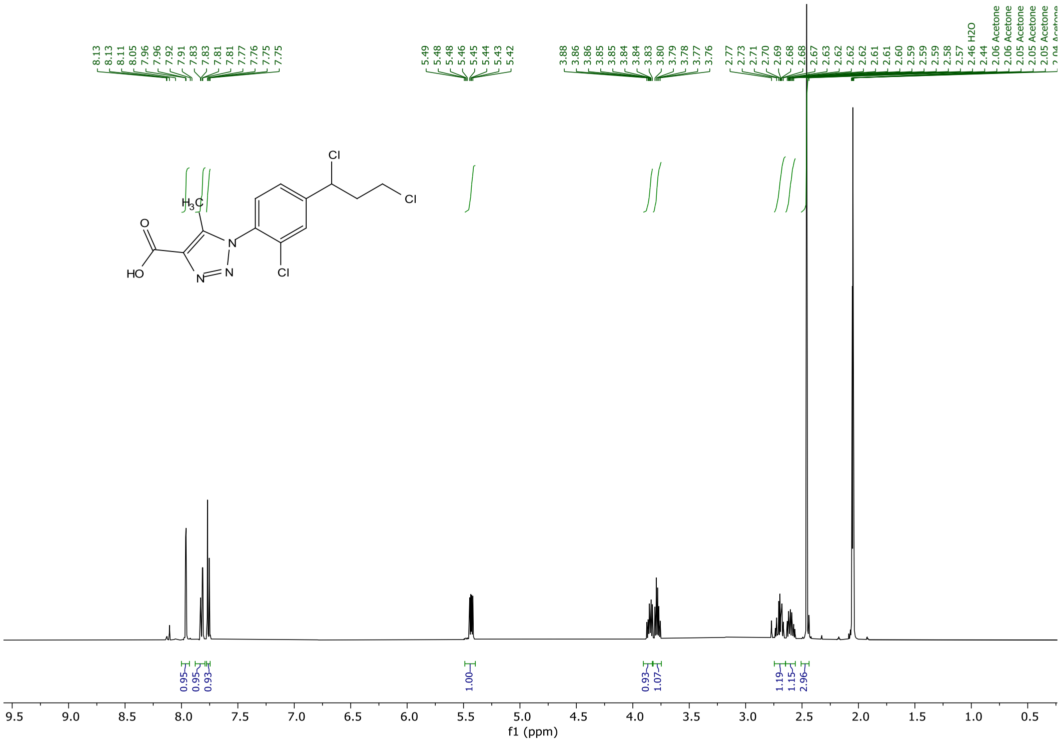

^{13}**C NMR** (126 MHz, d6-Acetone)

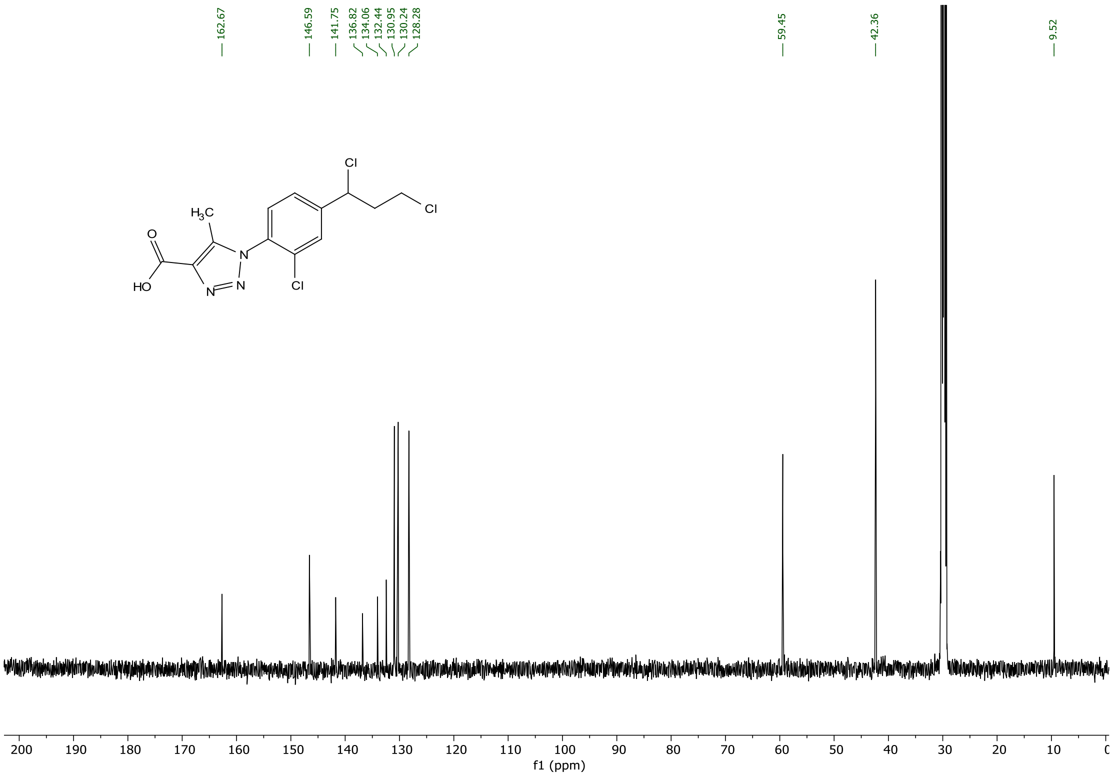

2-(1,2-Dichloroethyl)naphthalene (11a) **^{1}H NMR** (500 MHz, $CDCl_3$)

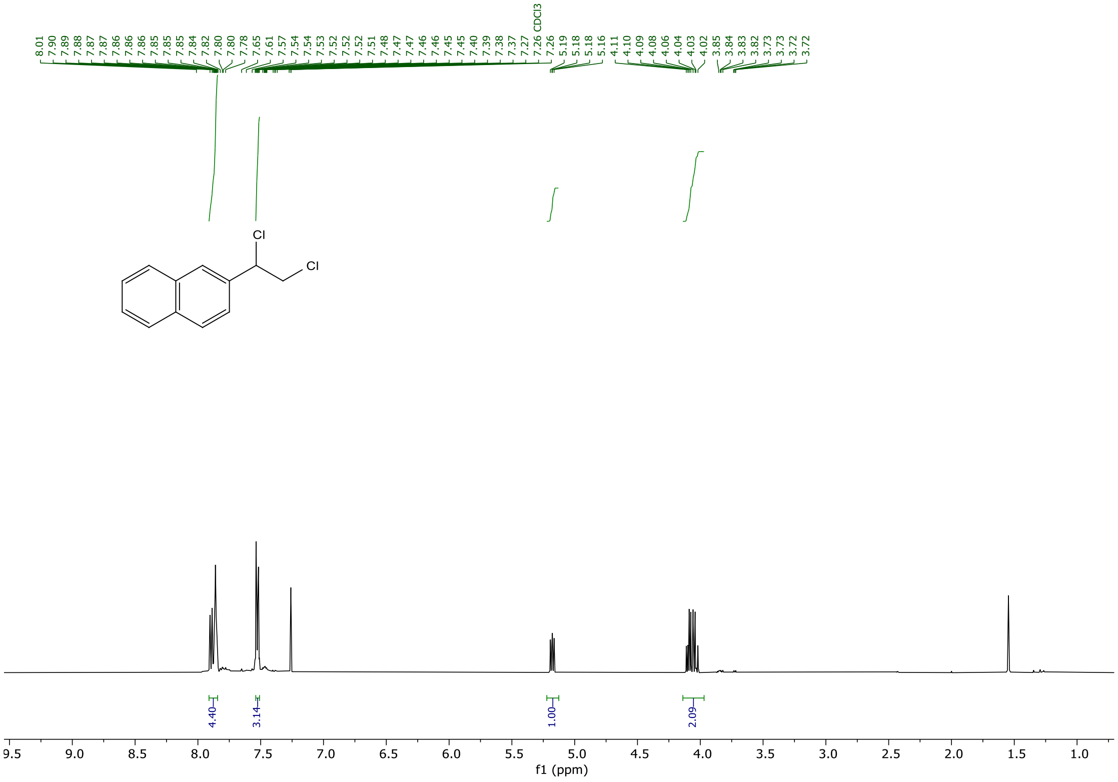

^{13}CNMR (126 MHz, $CDCl_3$)

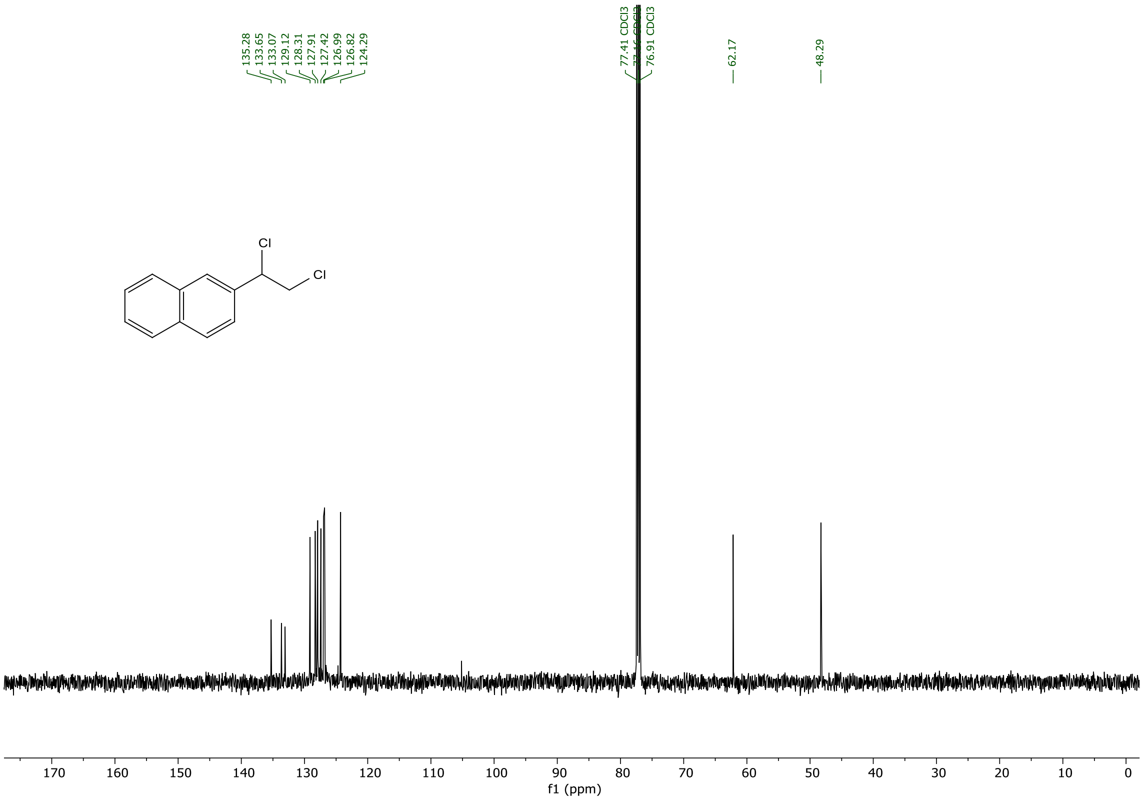

1-(1,2-Dichloroethyl)-4-methoxybenzene (11b) ^{1}H NMR (500 MHz, $CDCl_3$)

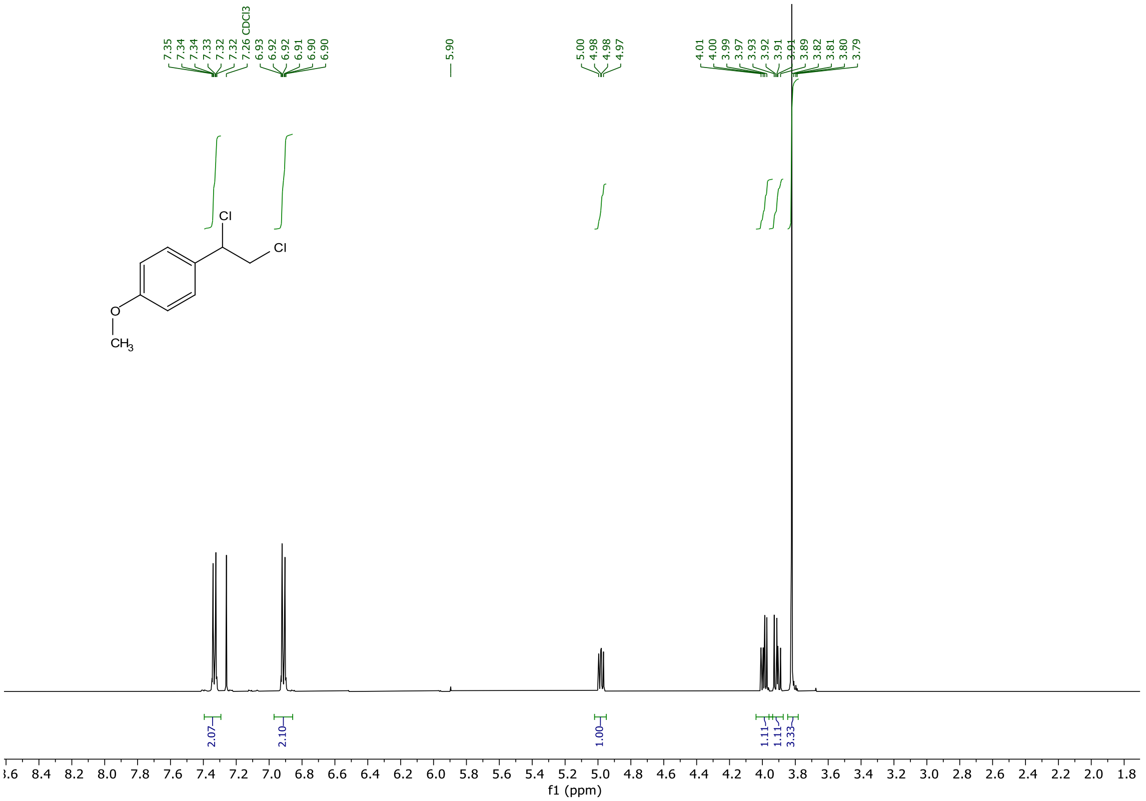

^{13}C NMR (126 MHz, $CDCl_3$)

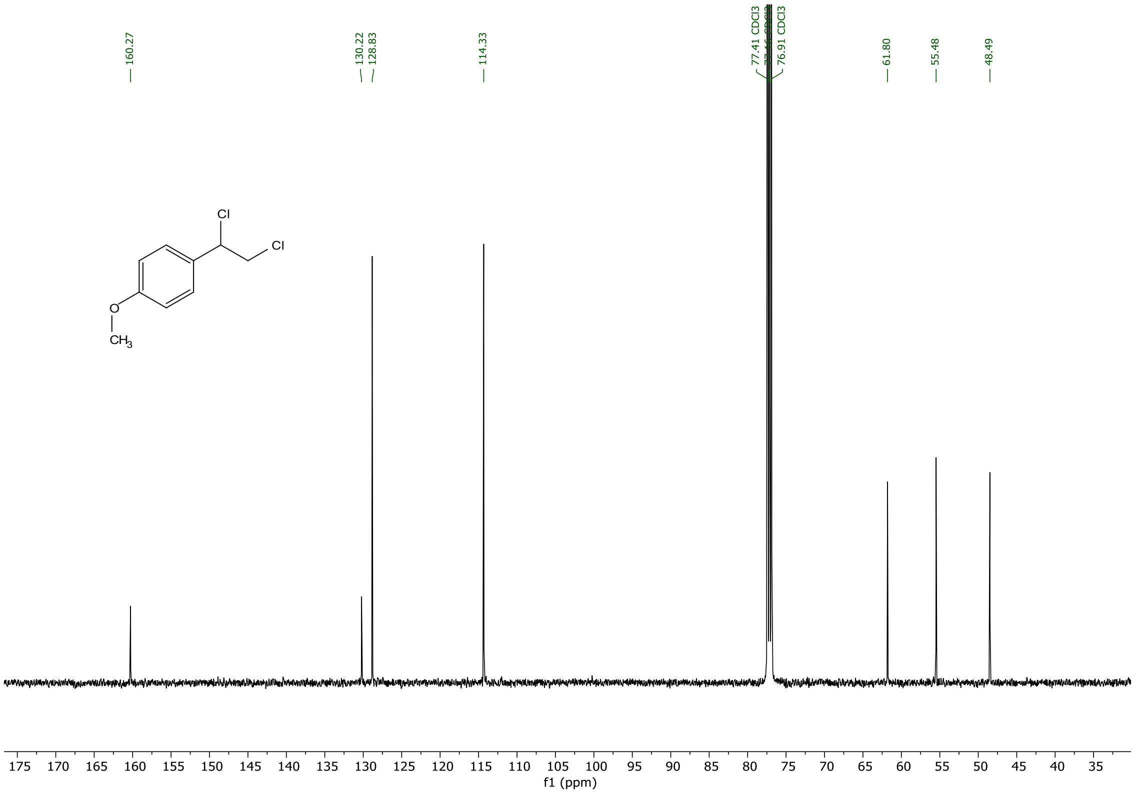

1-(1,2-Dichloroethyl)-4-(trifluoromethyl)benzene (11c) ^{1}H NMR (500 MHz, $CDCl_3$)

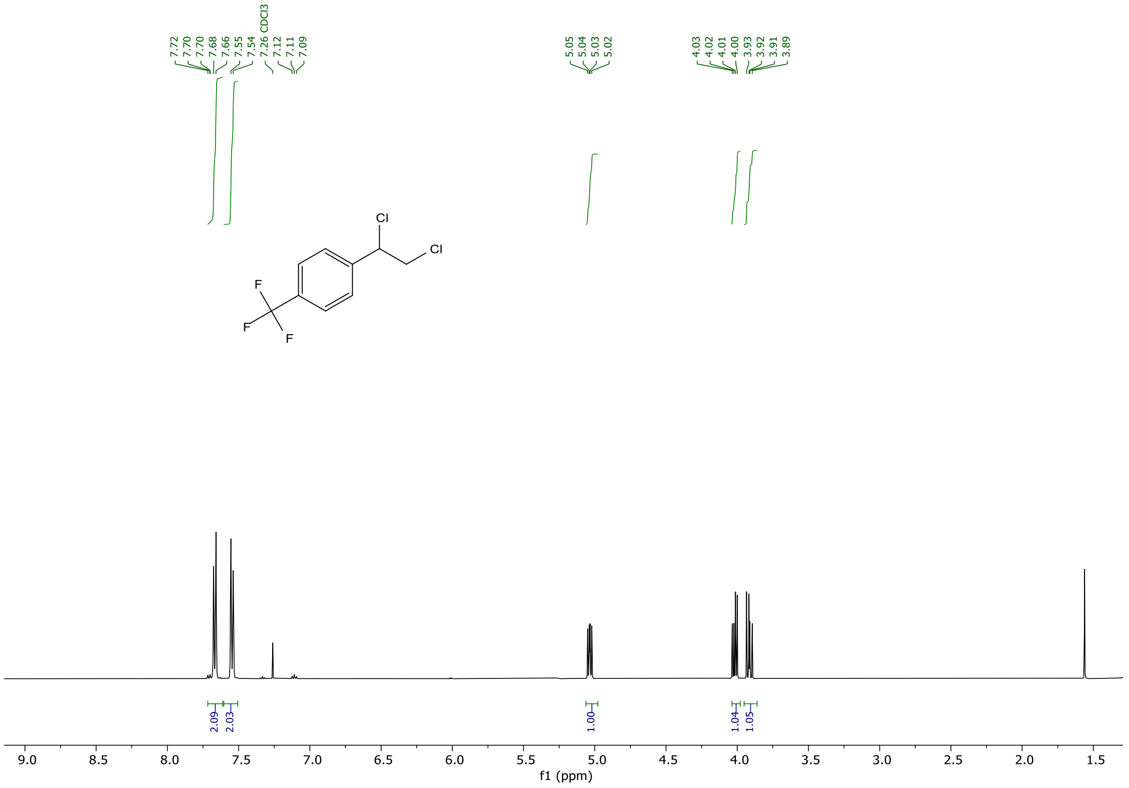

^{13}C NMR (126 MHz, $CDCl_3$)

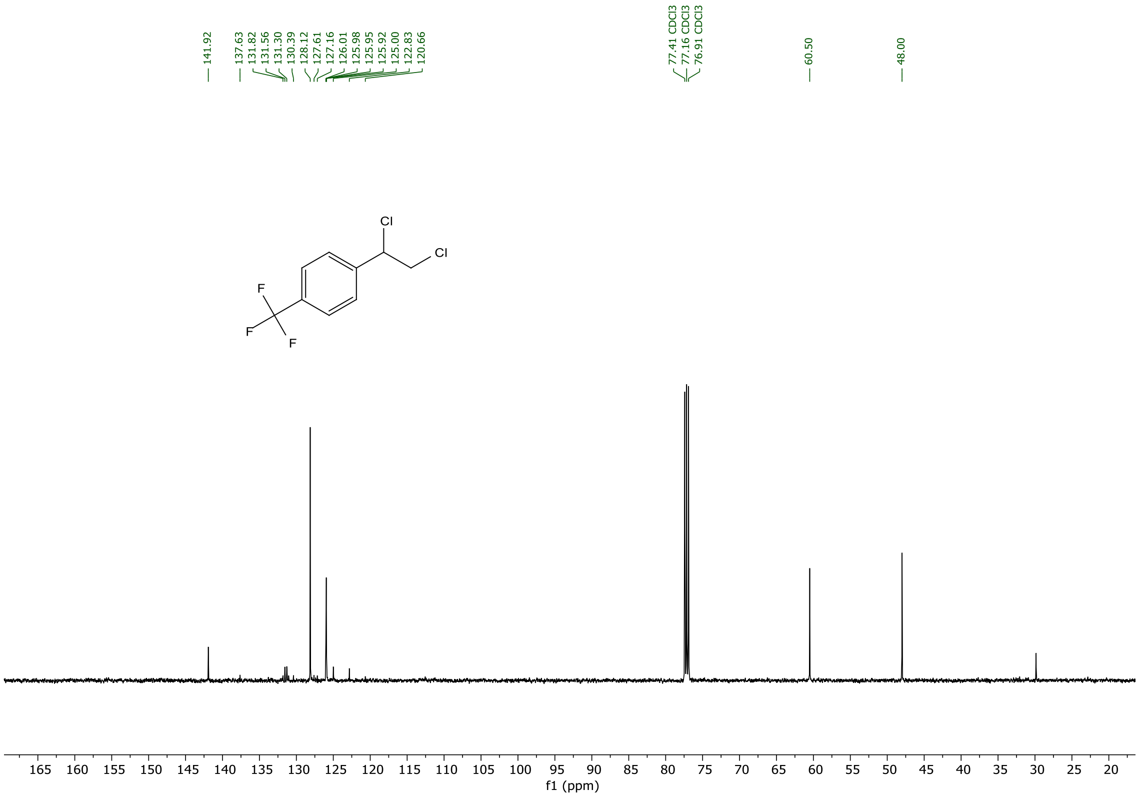

^{19}F NMR (470 MHz, $CDCl_3$)

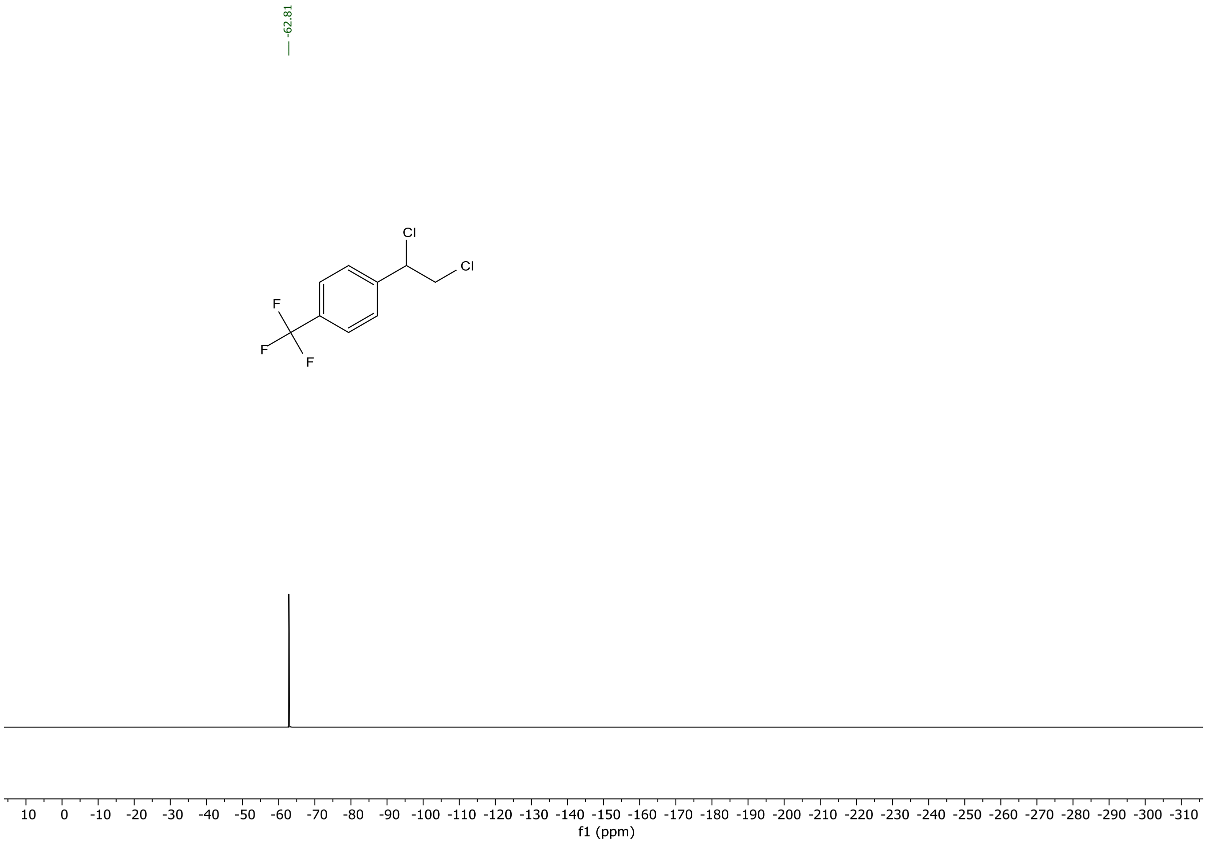

Methyl 4-(1,2-dichloroethyl)benzoate (11d) ^{1}H NMR (500 MHz, $CDCl_3$)

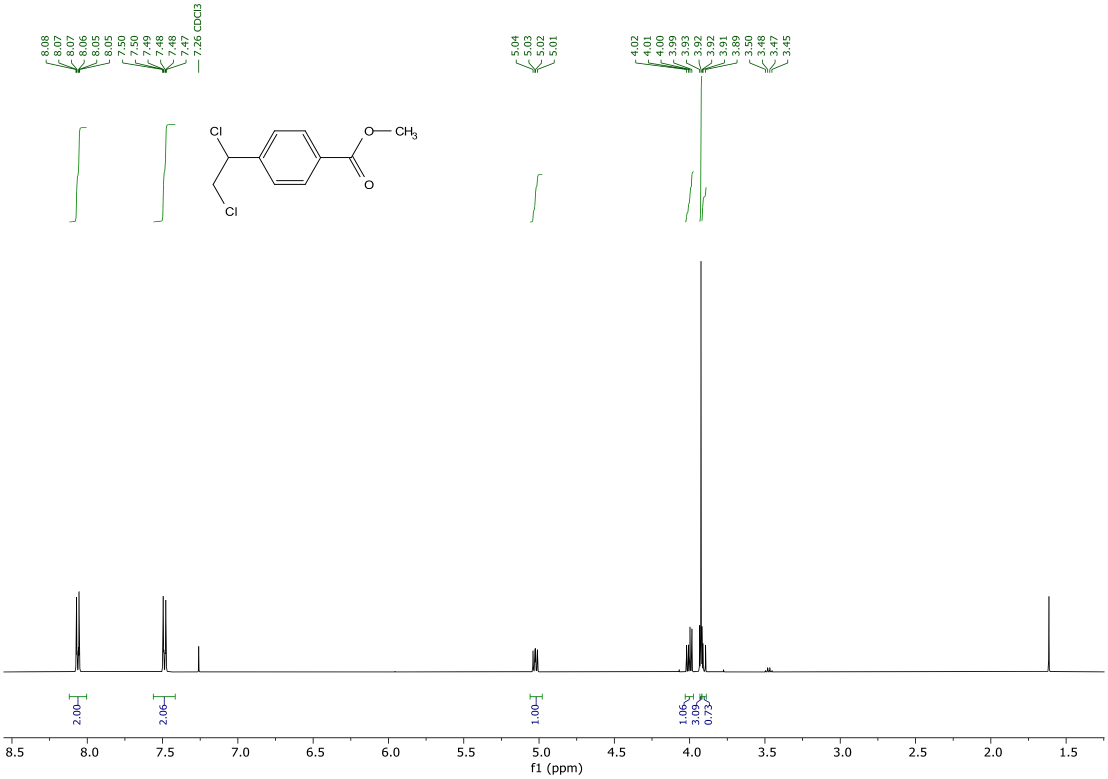

^{13}C NMR (126 MHz, $CDCl_3$)

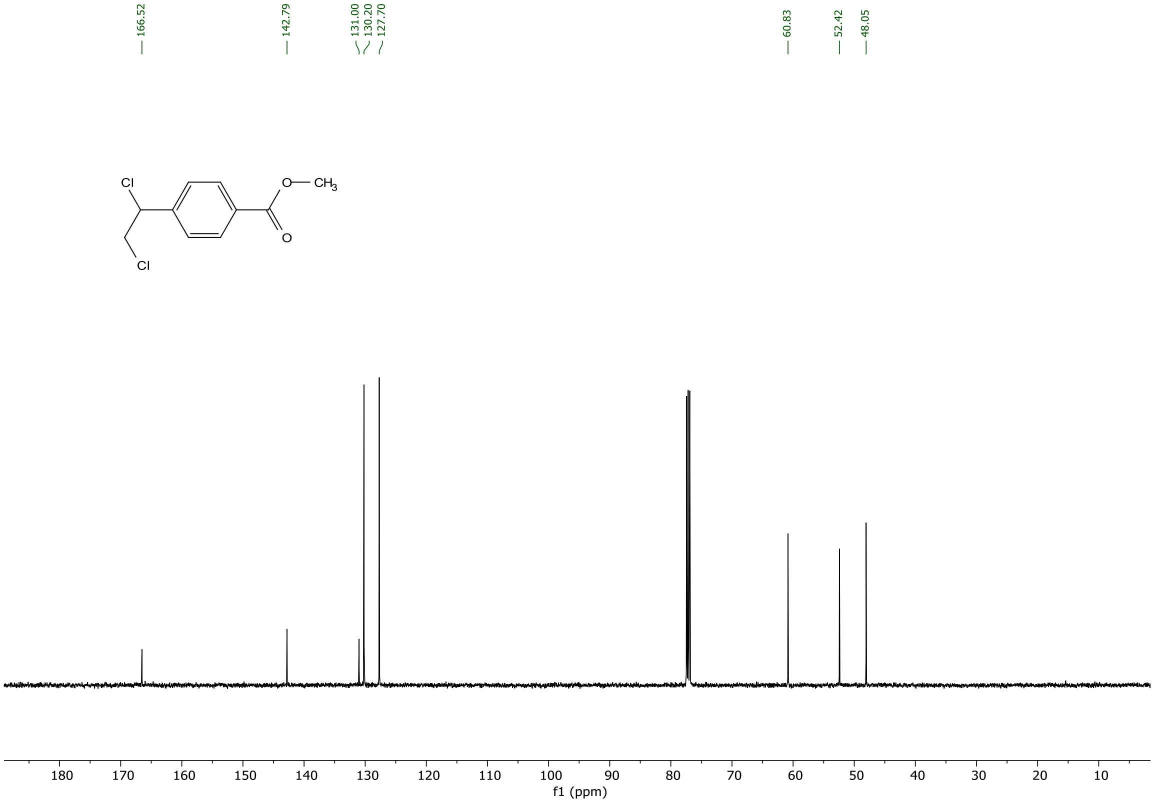

(1,2-Dichloropropan-2-yl)benzene (11e) ^{1}H NMR (500 MHz, $CDCl_3$)

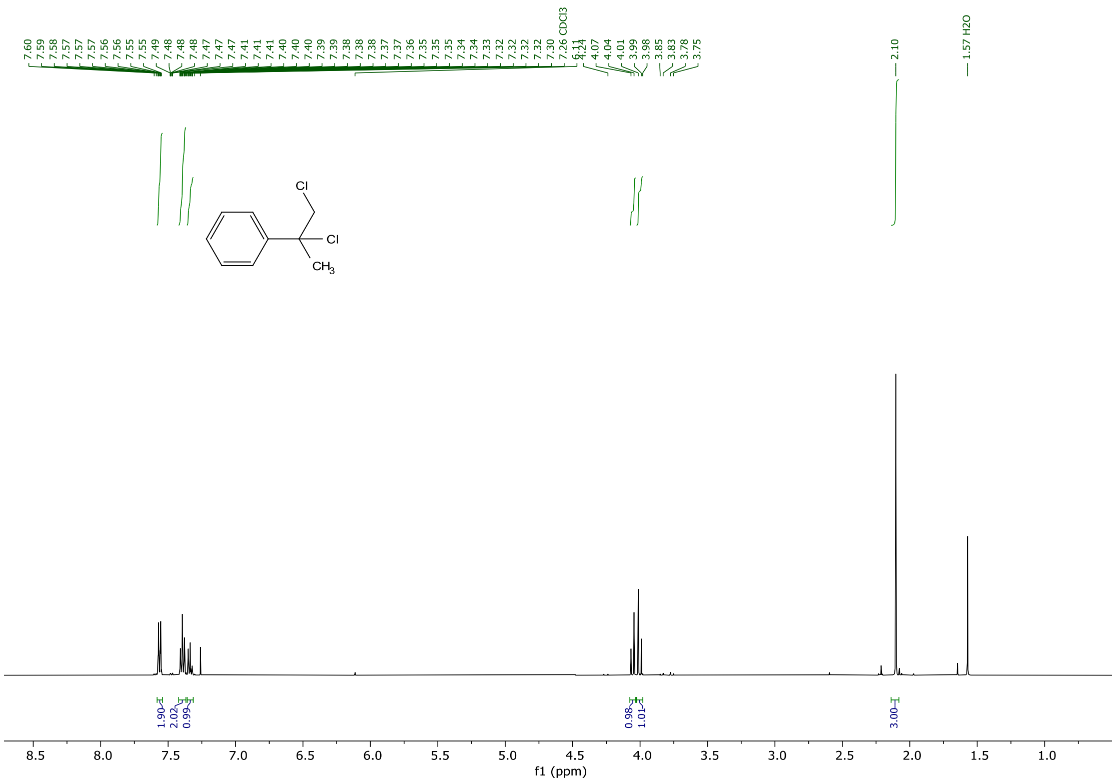

^{13}C NMR (126 MHz, $CDCl_3$)

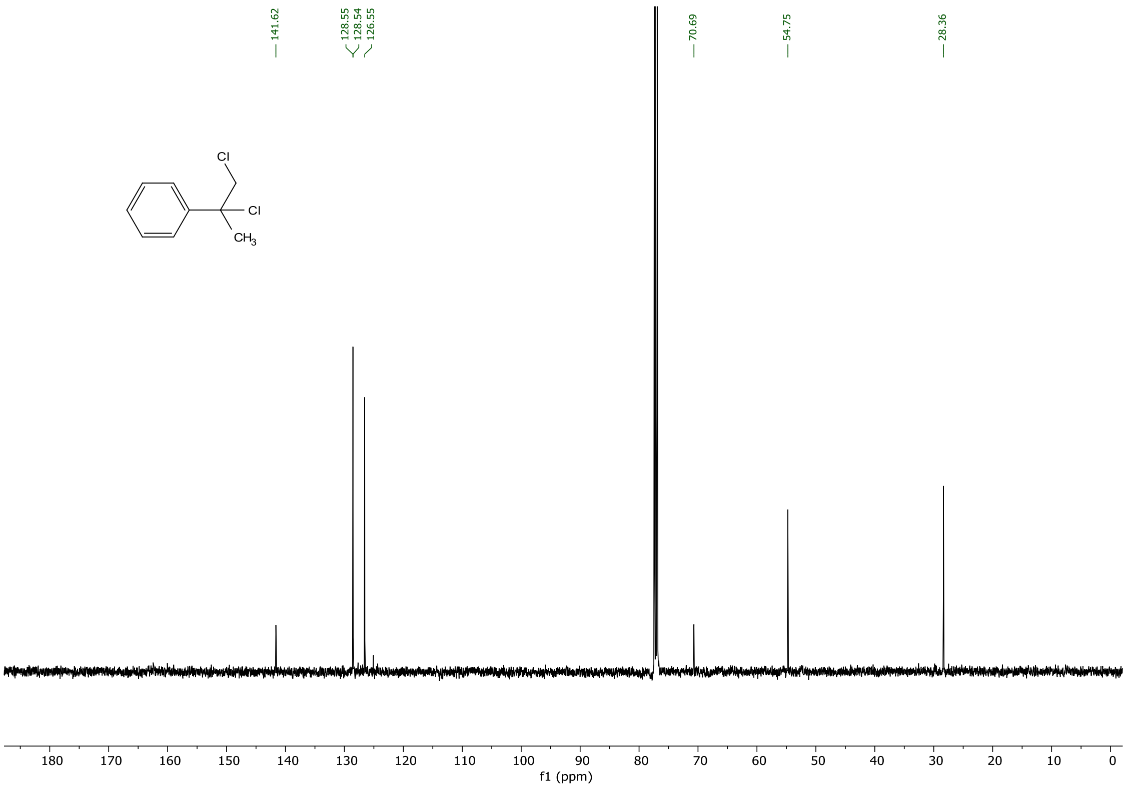

(1,2-Dichloropropyl)benzene (11f) ^{1}H NMR (500 MHz, $CDCl_3$). Mixture of two diastereomers

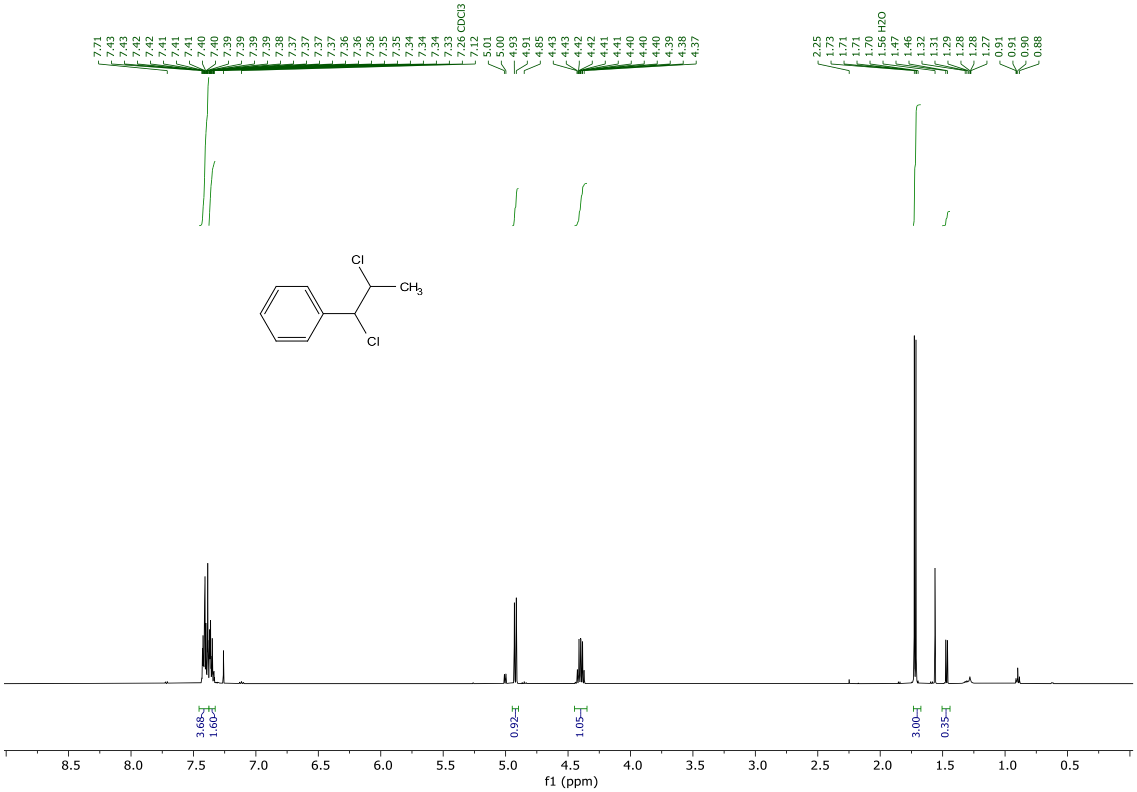

^{13}C NMR (126 MHz, $CDCl_3$)

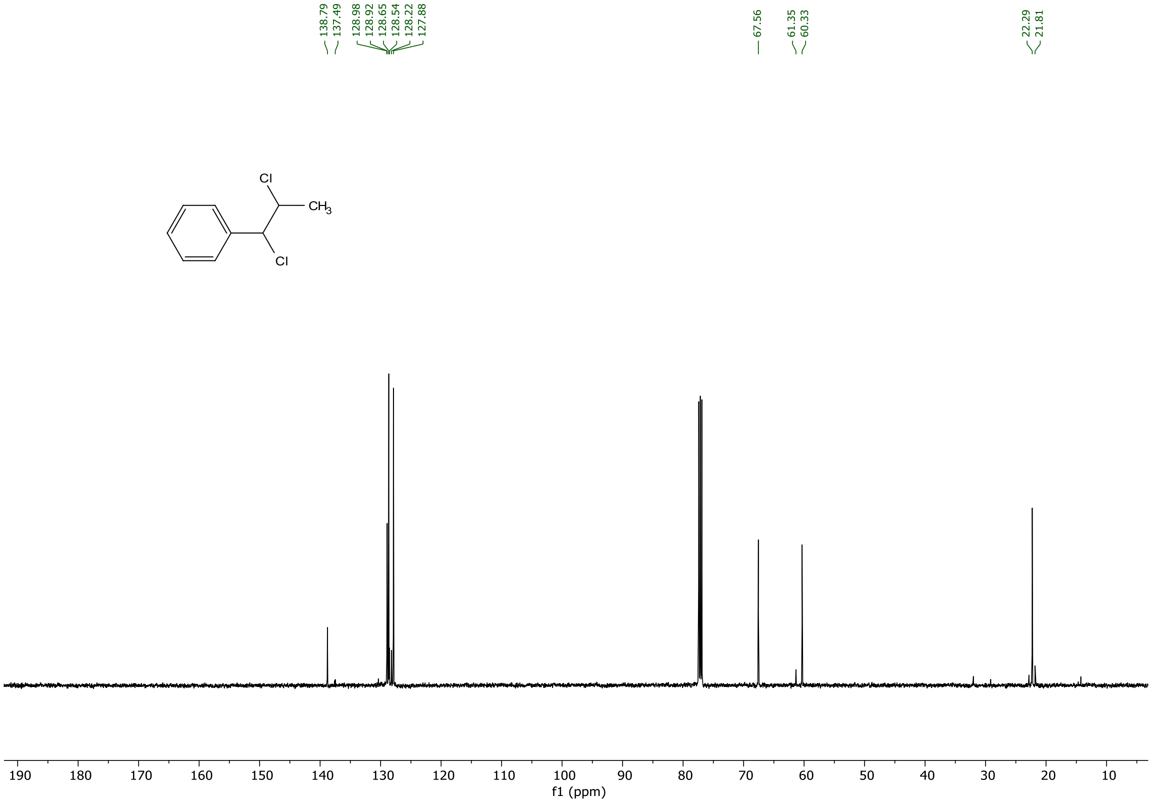

Methyl-2,3-dichloro-3-phenylpropanoate (11g) Major: ^{1}H NMR (500 MHz, $CDCl_3$)

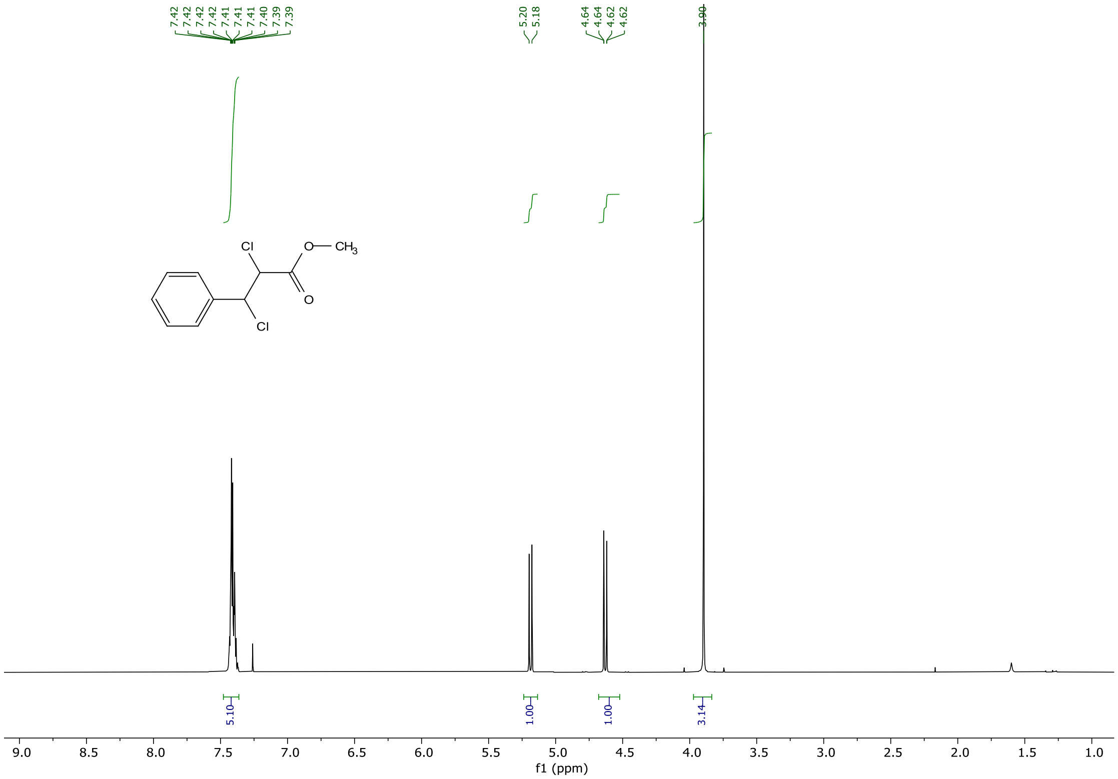

^{13}C NMR (126 MHz, $CDCl_3$)

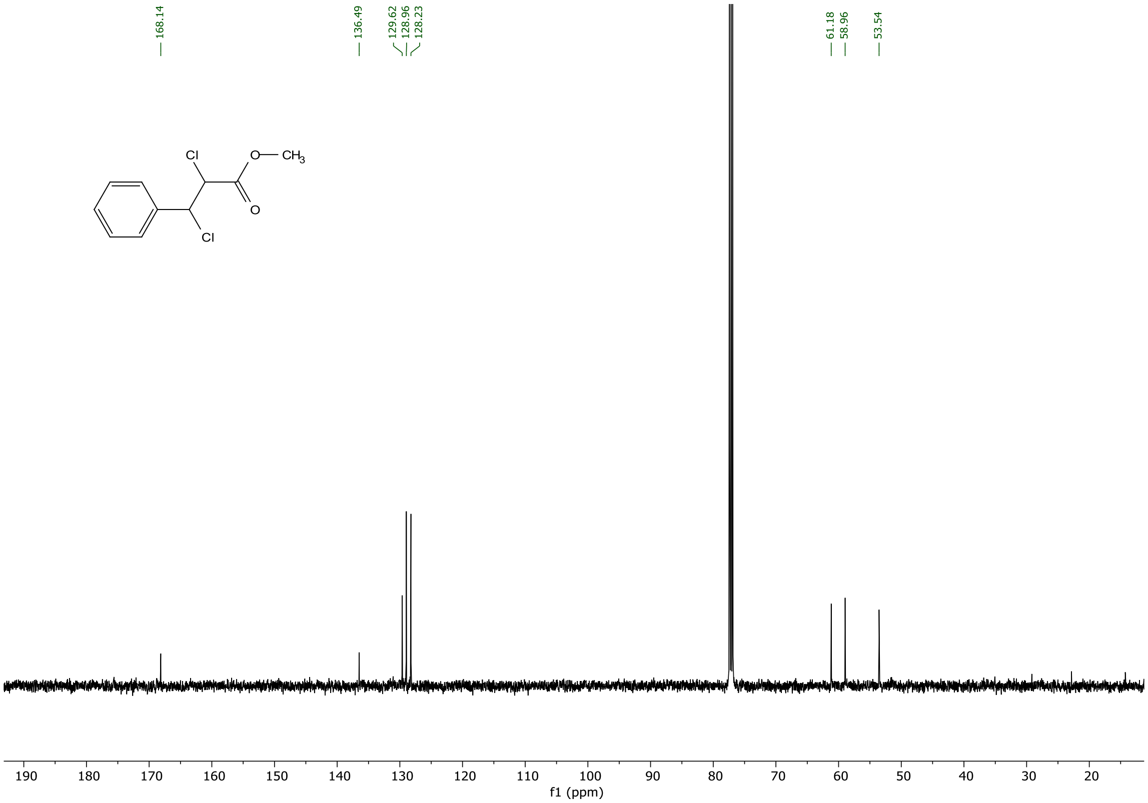

Minor: ^{1}**H NMR** (500 MHz, $CDCl_3$)

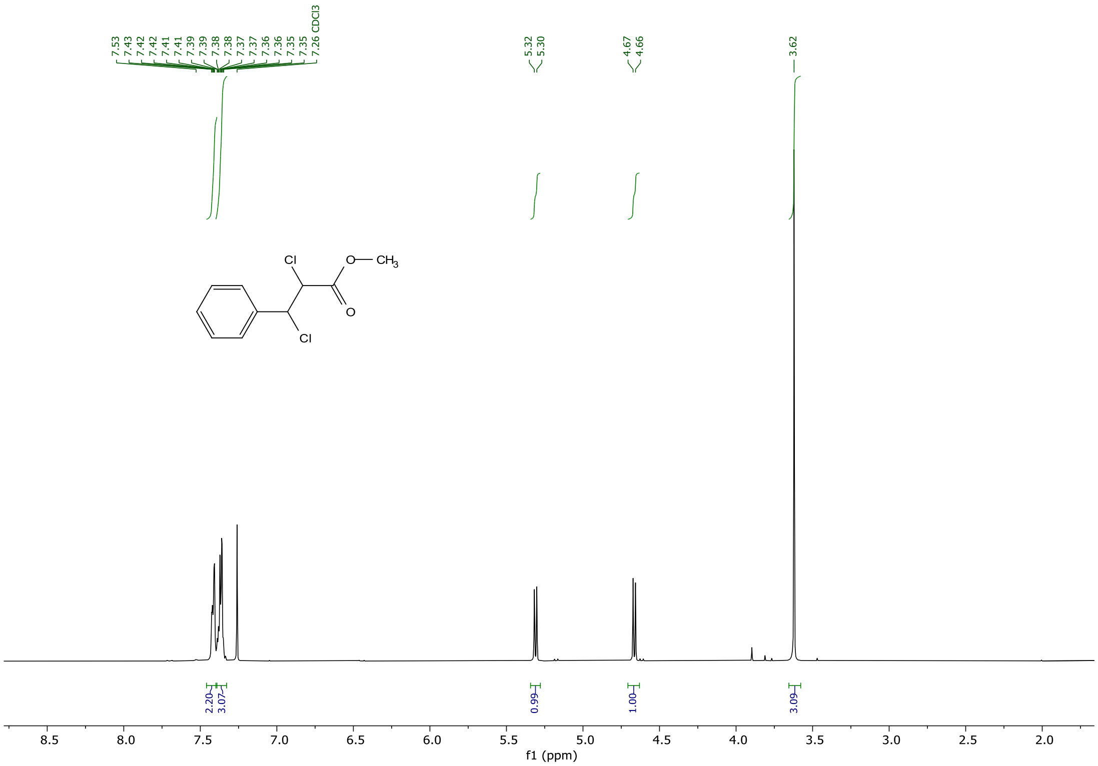

^{13}**C NMR** (126 MHz, $CDCl_3$)

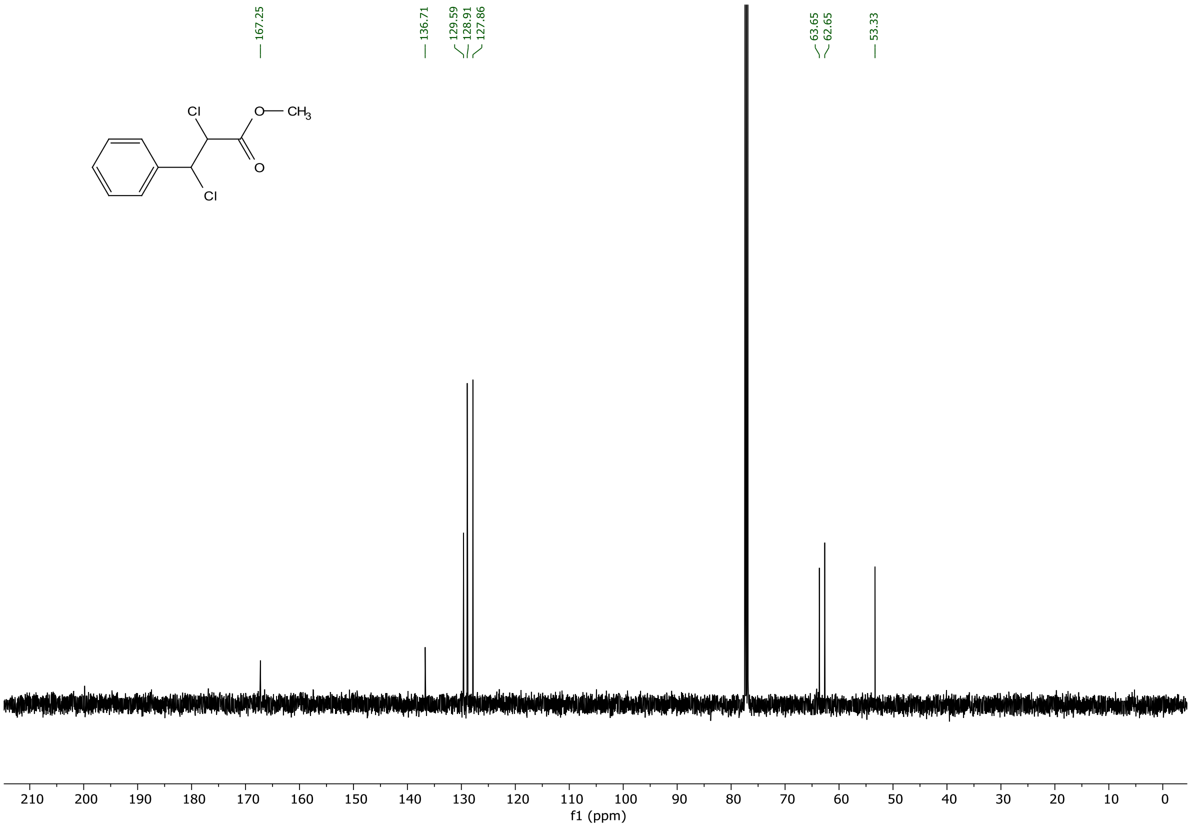

(2,3-Dichloropropyl)benzene (11h1) and (1,3-dichloropropan-2-yl)benzene (11h2)

^{1}H NMR (500 MHz, $CDCl_3$,)

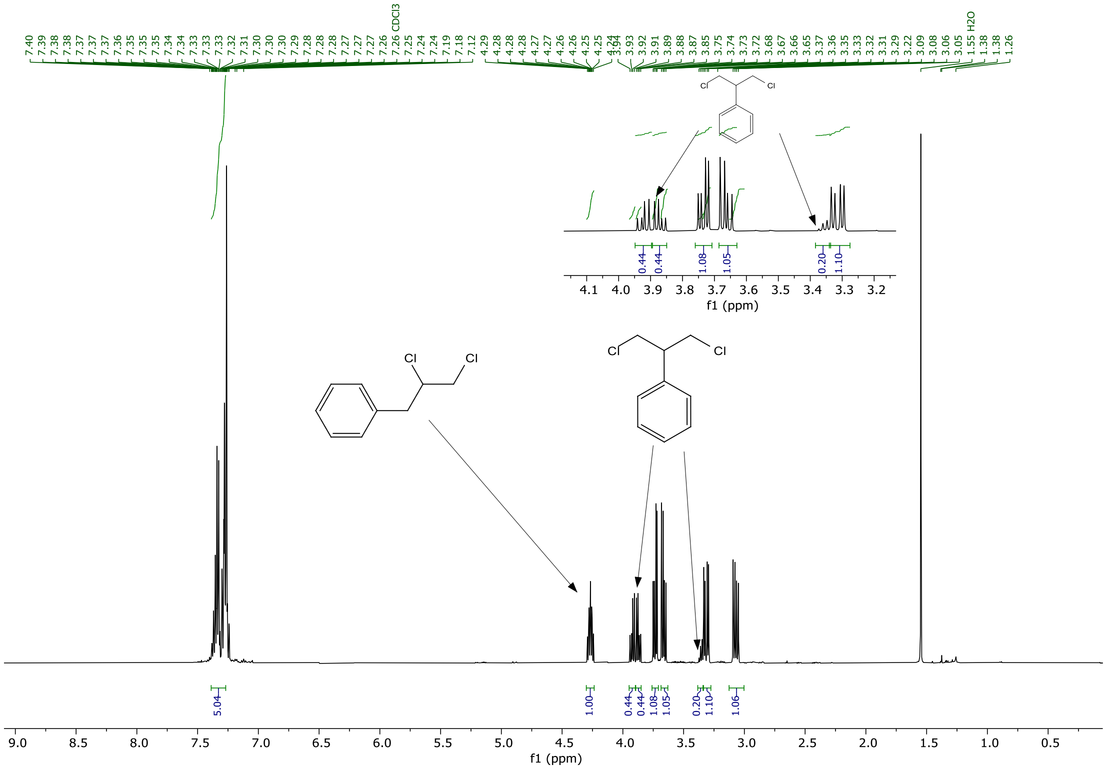

^{13}C NMR (126 MHz, $CDCl_3$)

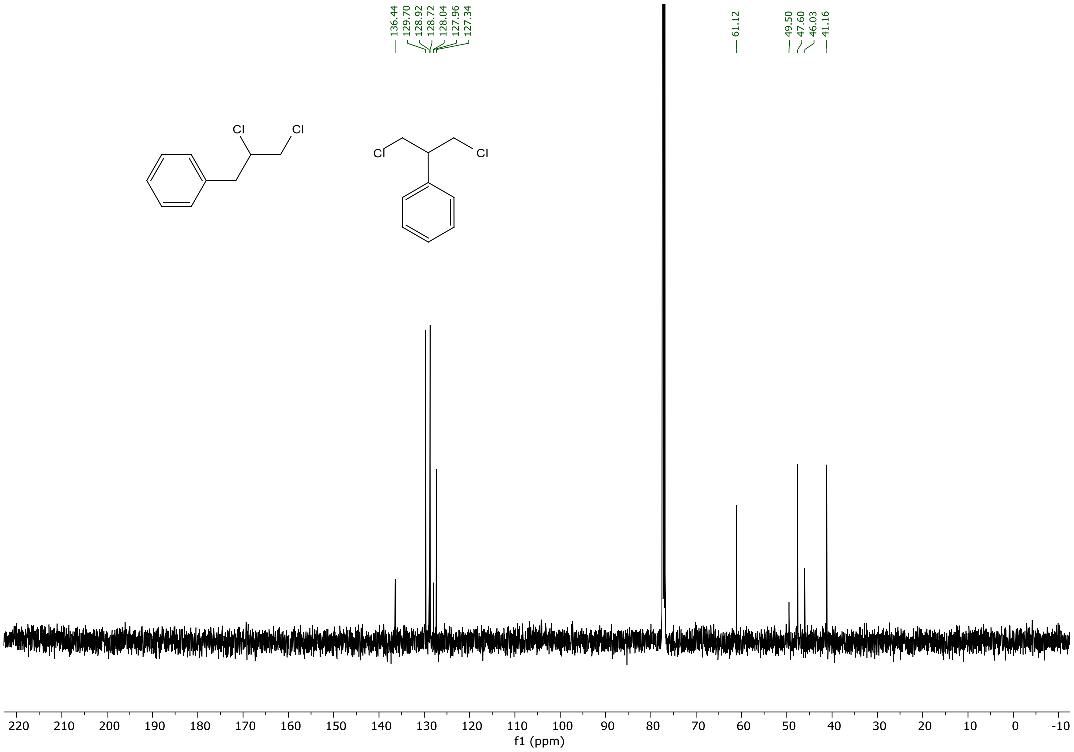

(3,4-Dichlorobutyl)benzene (11i). ^{1}H NMR (500 MHz, $CDCl_3$)

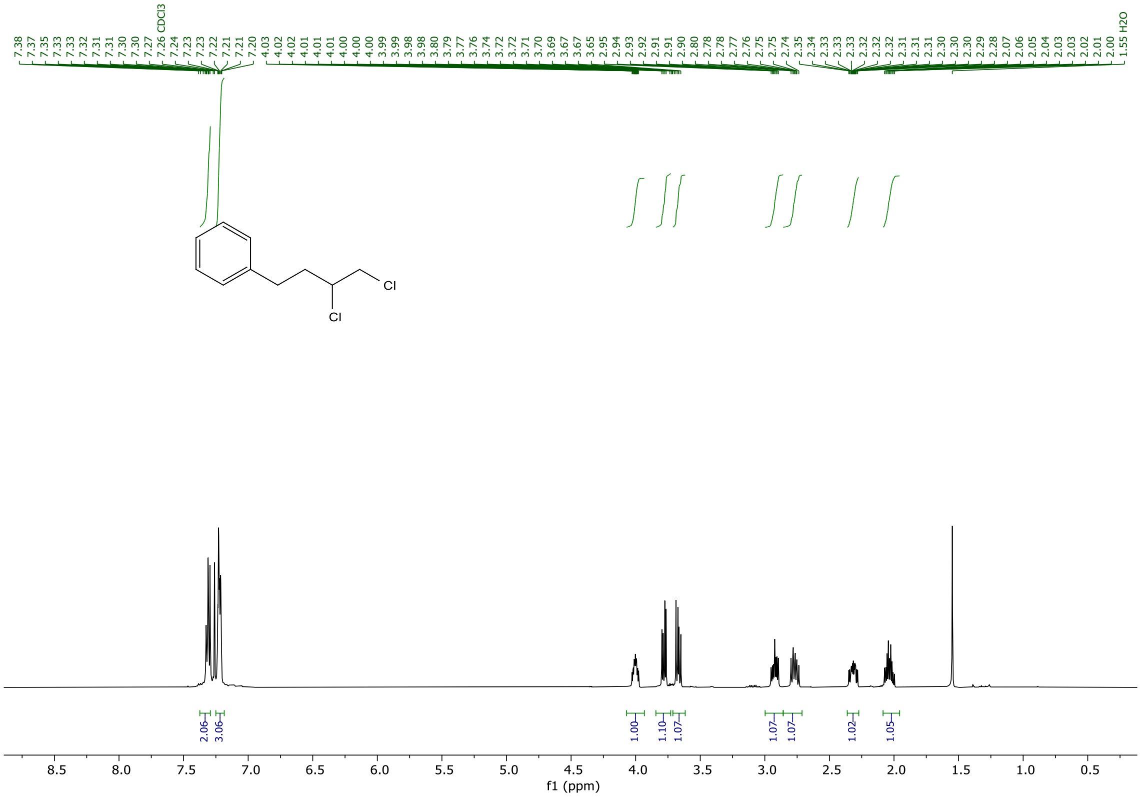

^{13}C NMR (126 MHz, $CDCl_3$)

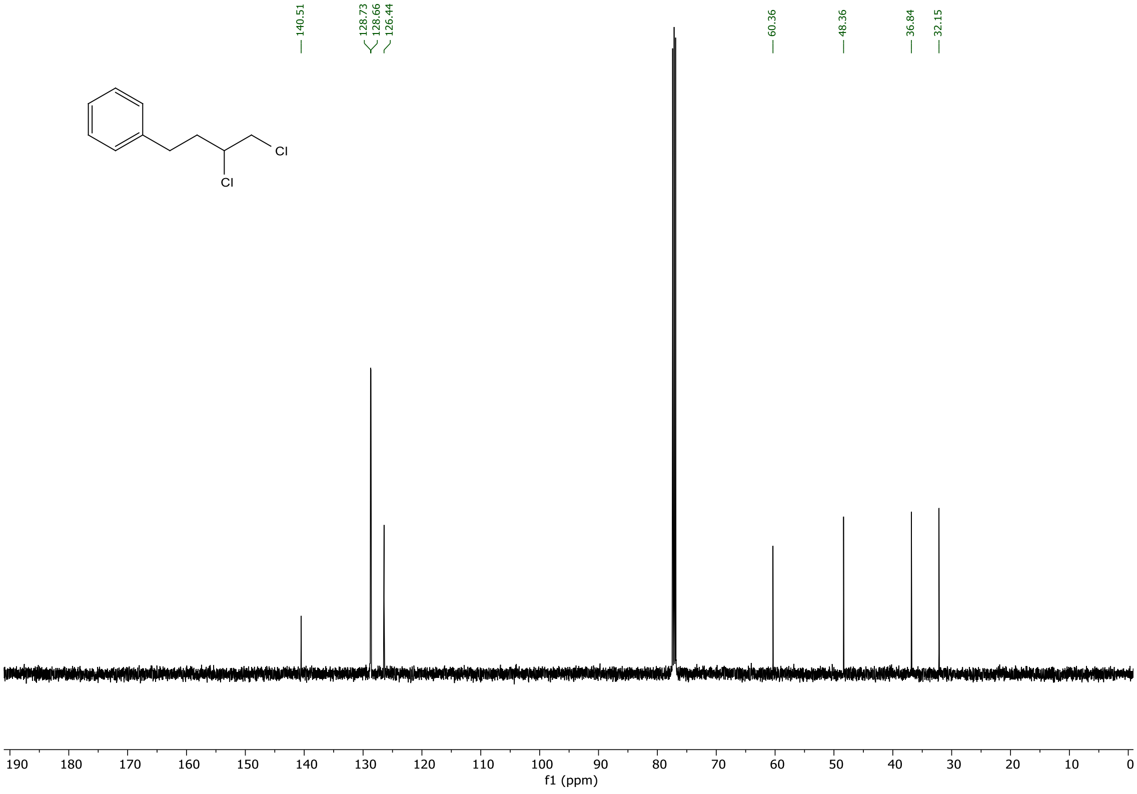

1-(2-(2,3-Dichloropropoxy)phenoxy)-3-(isopropylamino)propan-2-ol (11j).

^{1}H NMR (500 MHz, $CDCl_3$)

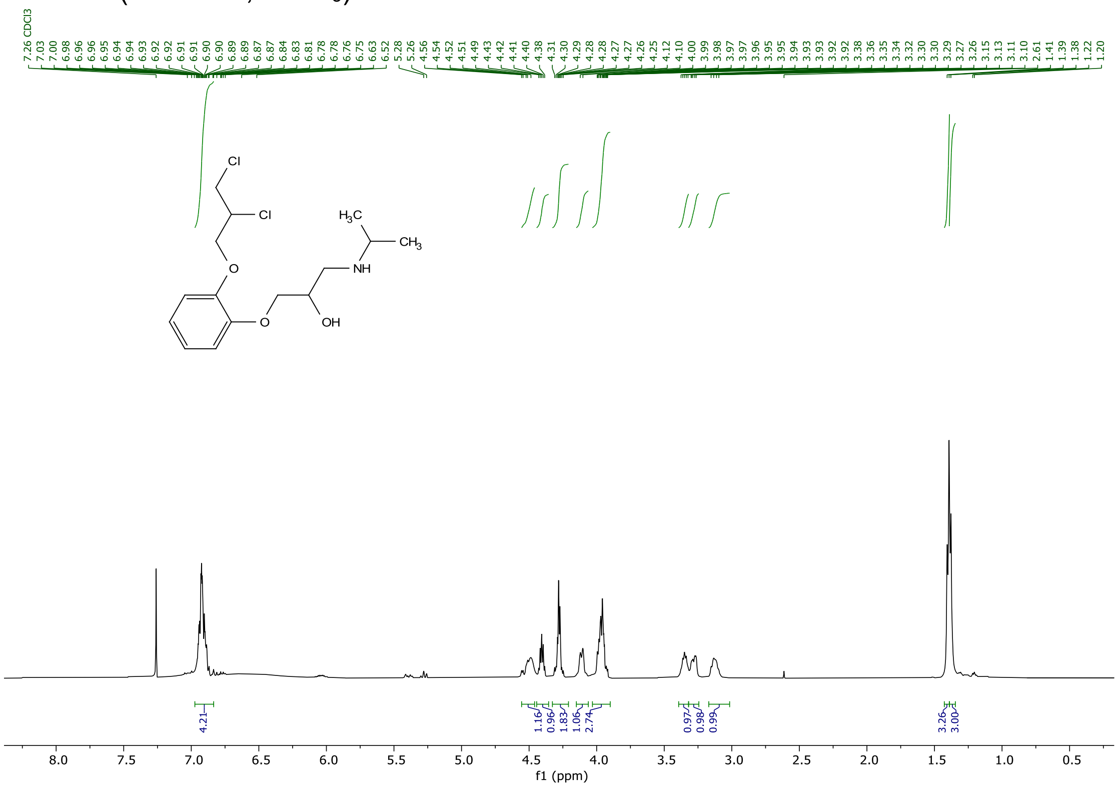

^{13}C NMR (126 MHz, $CDCl_3$)

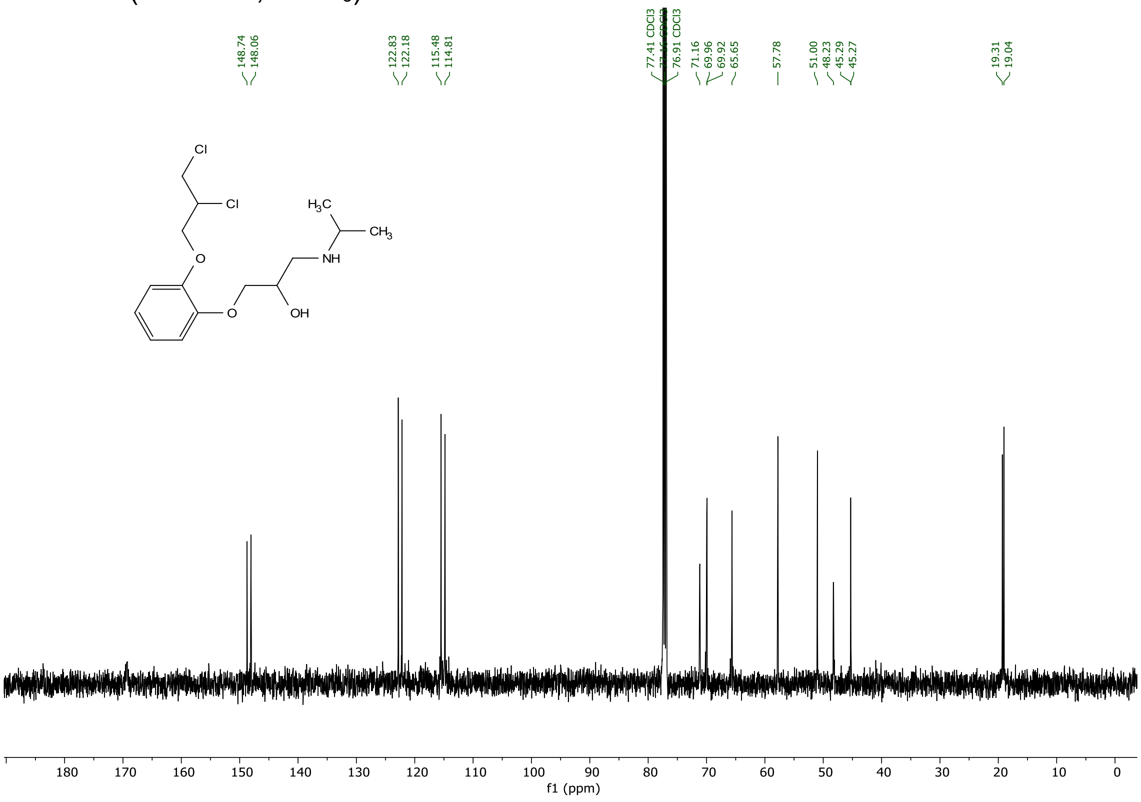

1-Bromo-4-(1-chloroethyl)benzene (13a) ^{1}H NMR (500 MHz, $CDCl_3$)

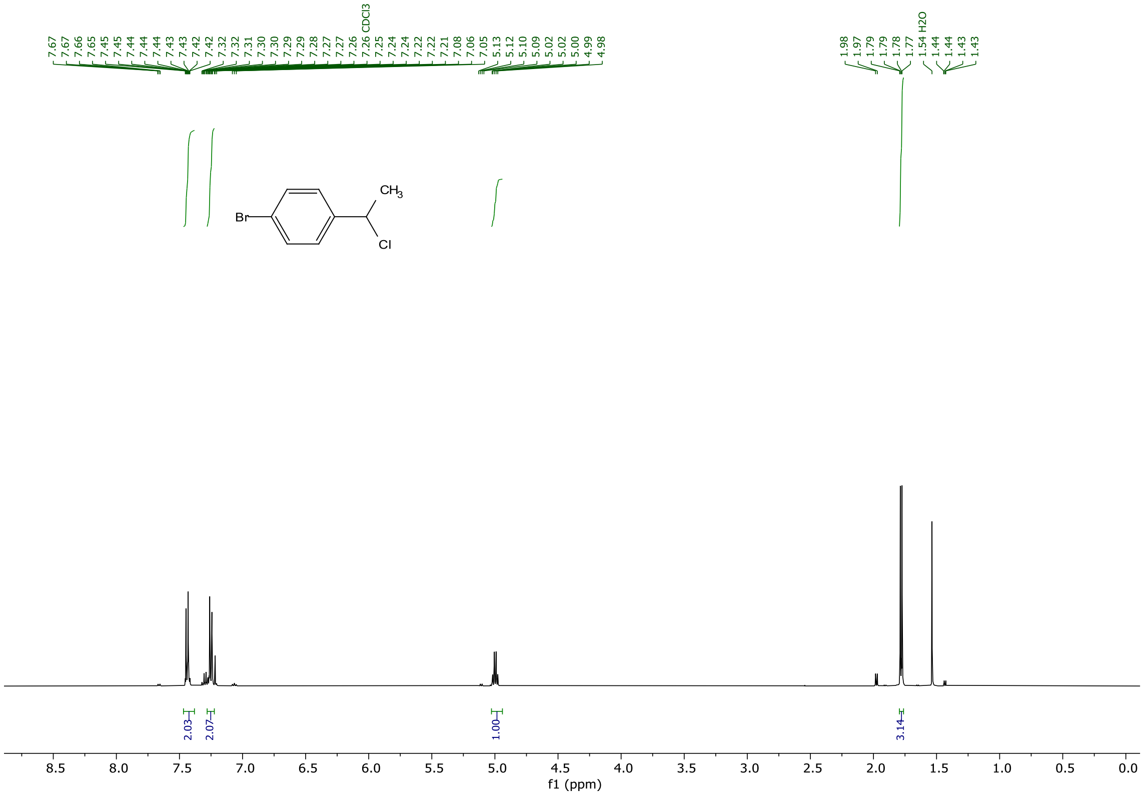

^{13}C NMR (126 MHz, $CDCl_3$)

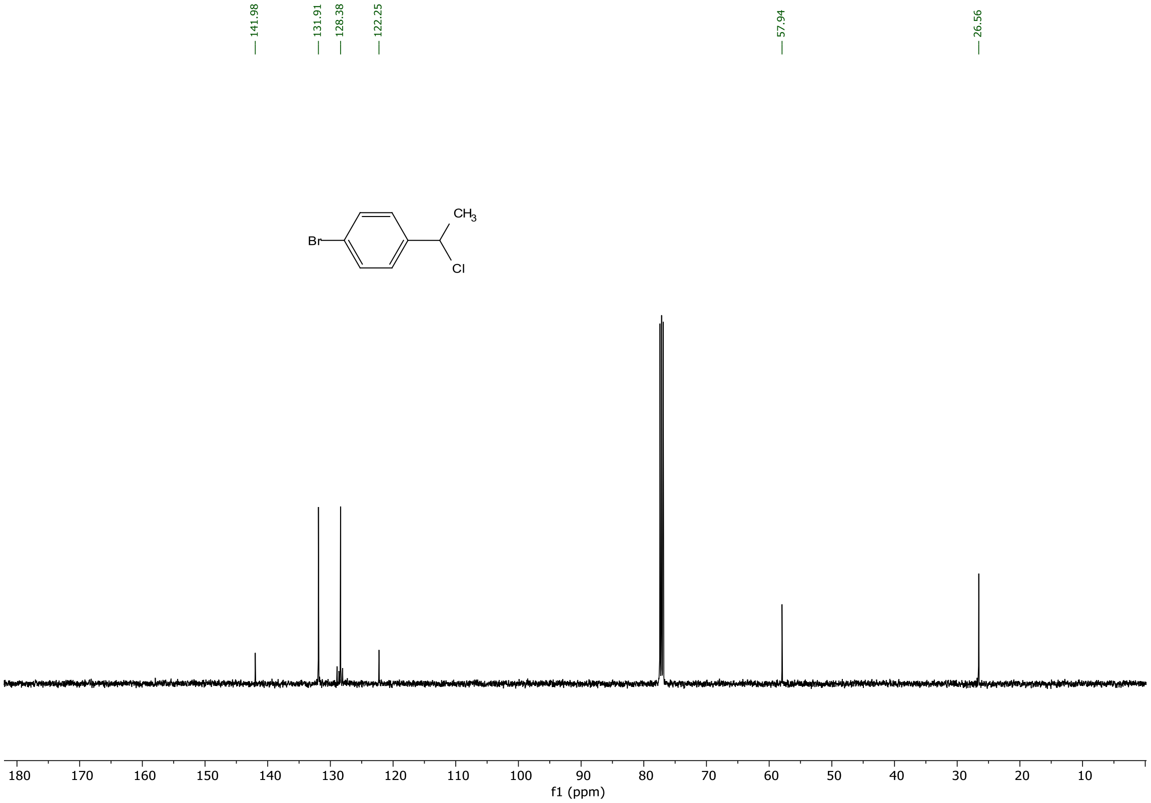

(1-Chloroethyl)benzene (13b) ^{1}H NMR (500 MHz, $CDCl_3$)

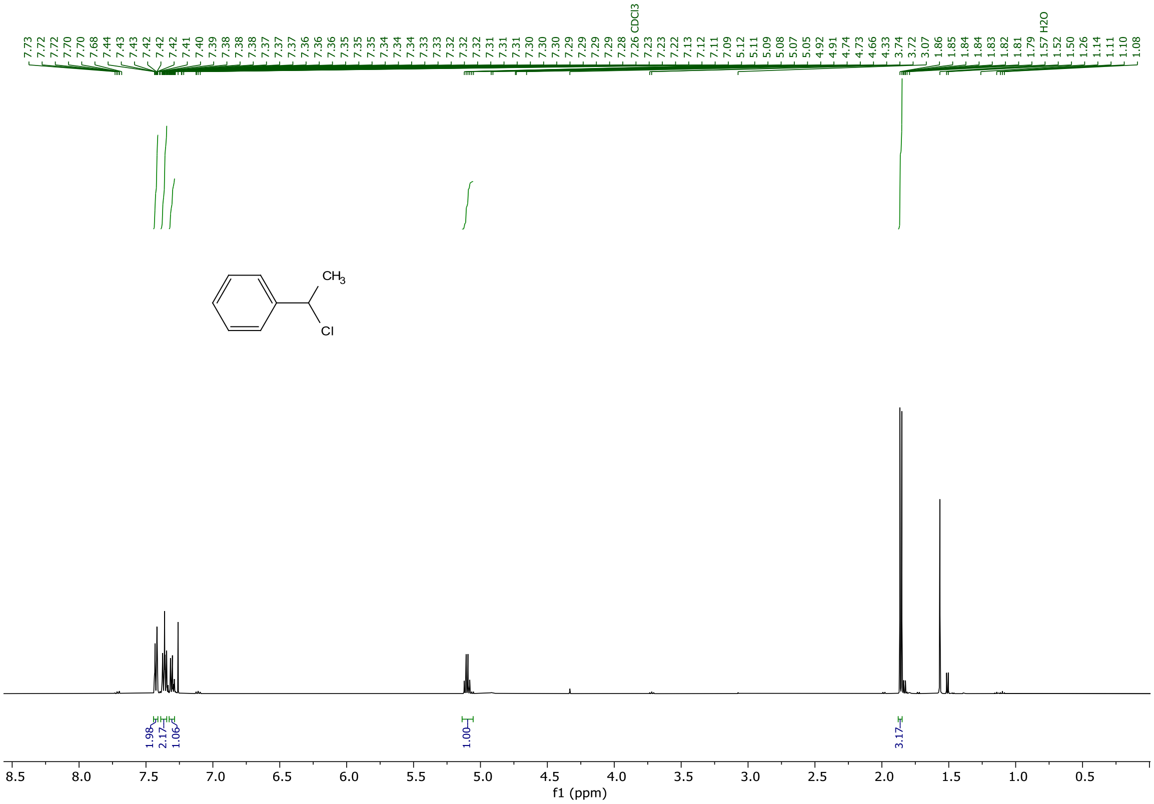

^{13}C NMR (126 MHz, $CDCl_3$)

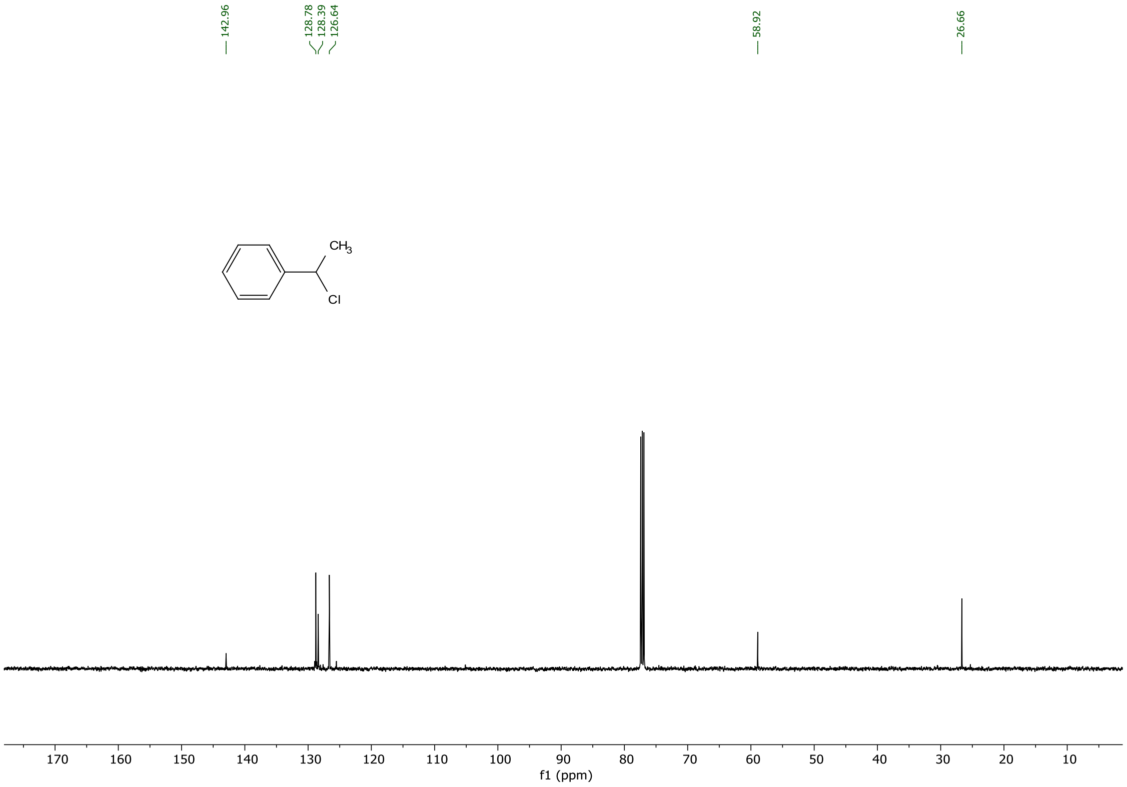

1-(4-Bromophenyl)cyclobutan-1-ol (13c) ^{1}**H NMR** (500 MHz, $CDCl_3$)

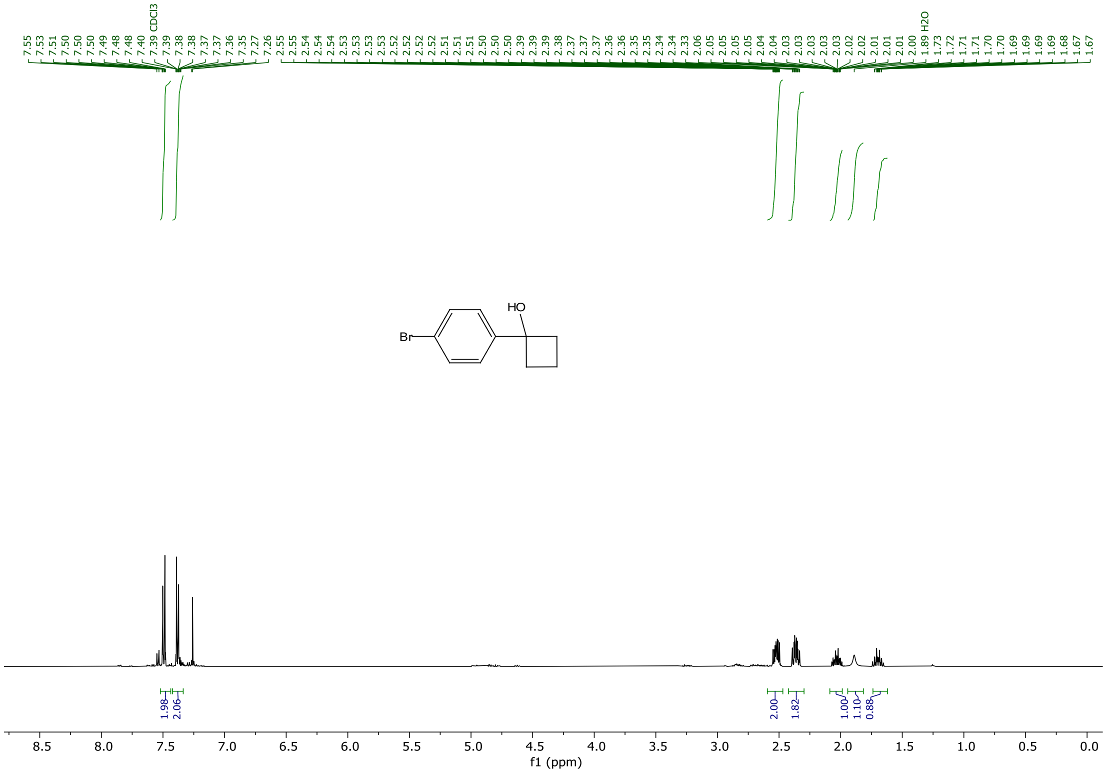

^{13}**C NMR** (126 MHz, $CDCl_3$)

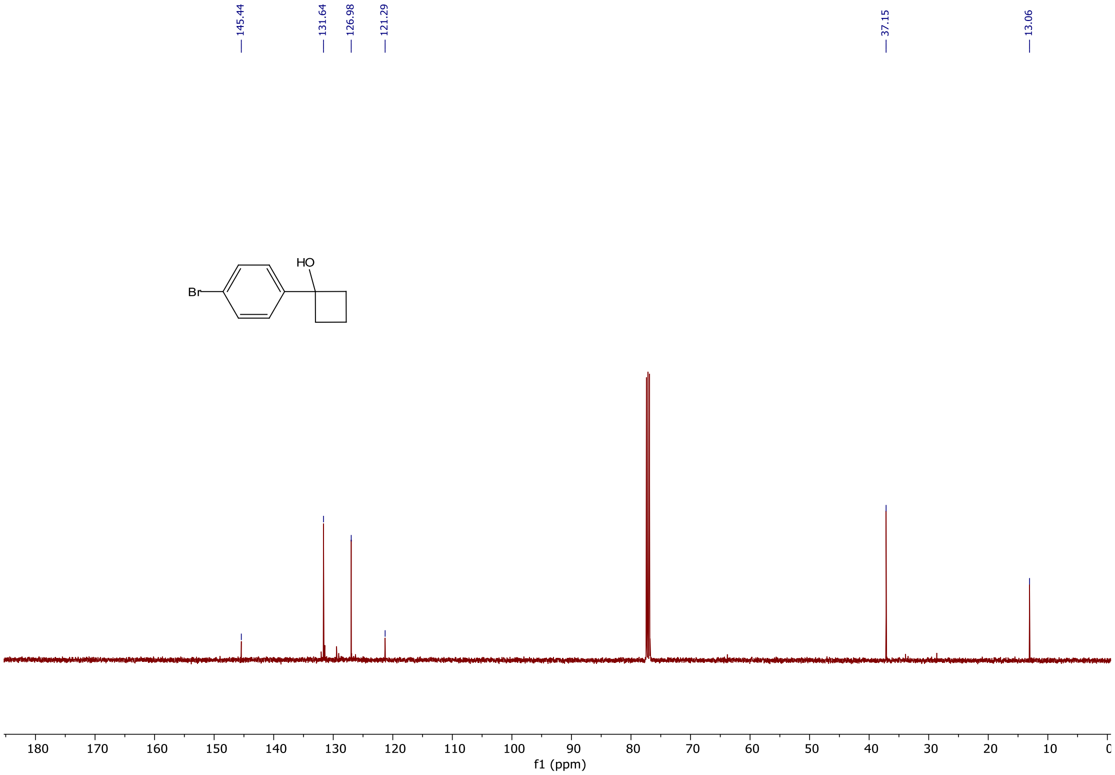

2-Chloro-1-phenylbutan-1-one (13d) ^{1}H NMR (500 MHz, $CDCl_3$)

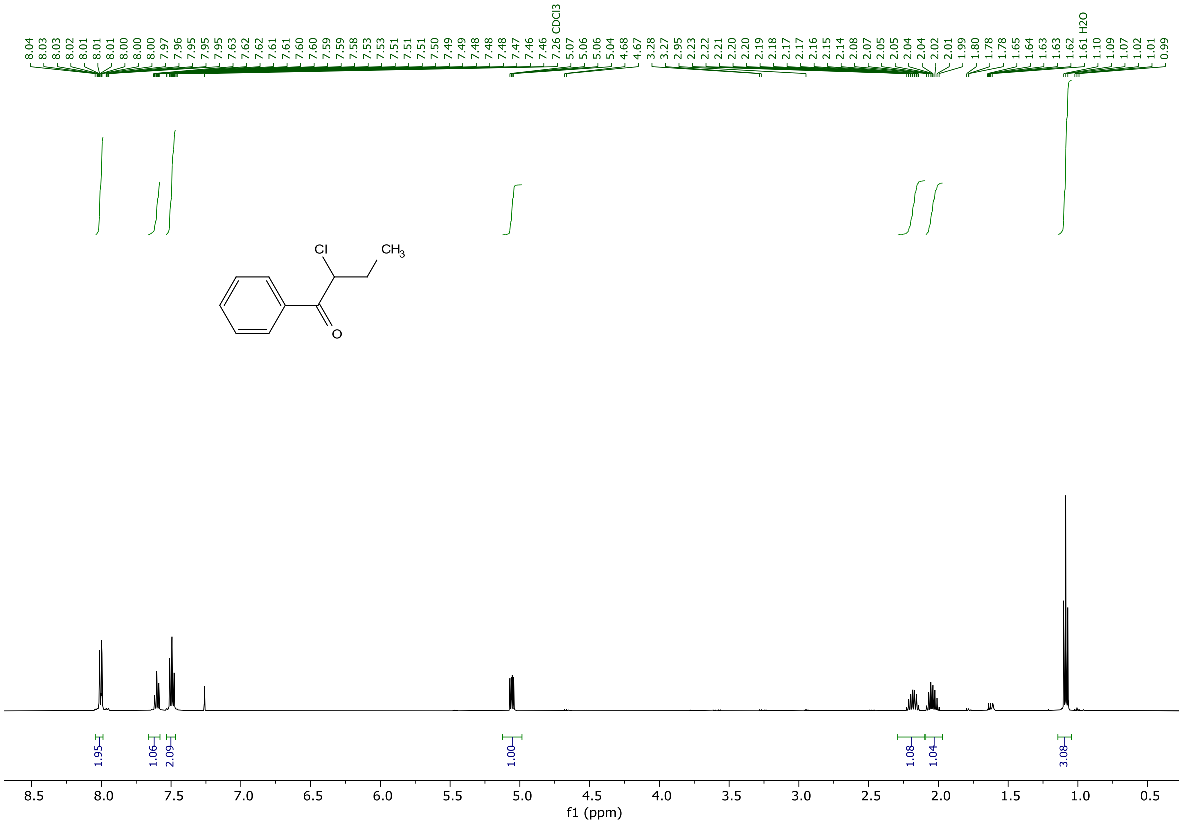

^{13}C NMR (126 MHz, $CDCl_3$)

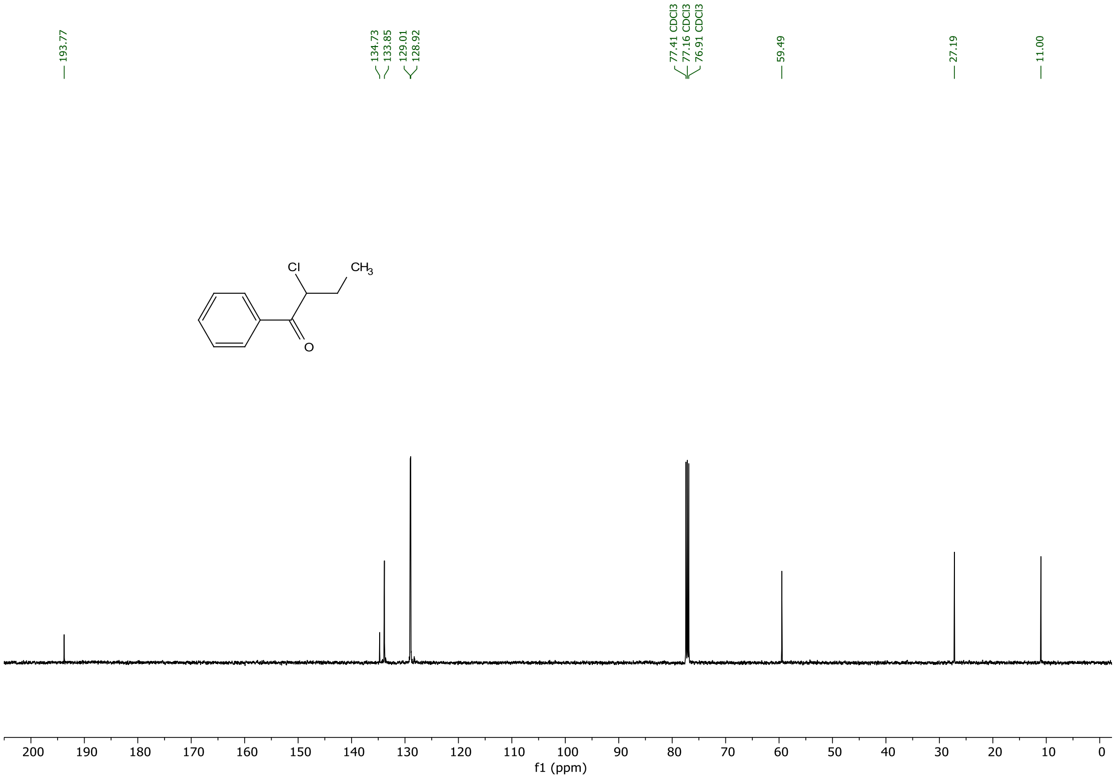

2-Chloro-2,3-dihydro-1H-inden-1-one (13e) ^{1}H NMR (500 MHz, $CDCl_3$)

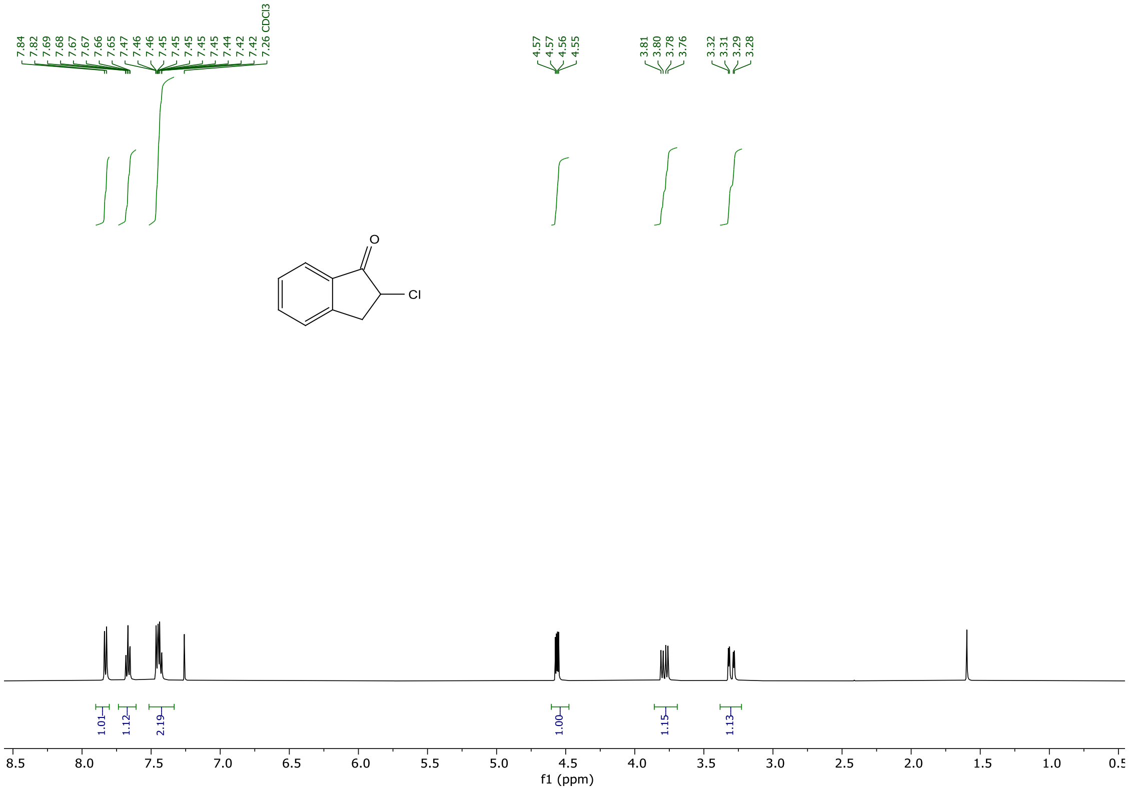

^{13}C NMR (126 MHz, $CDCl_3$)

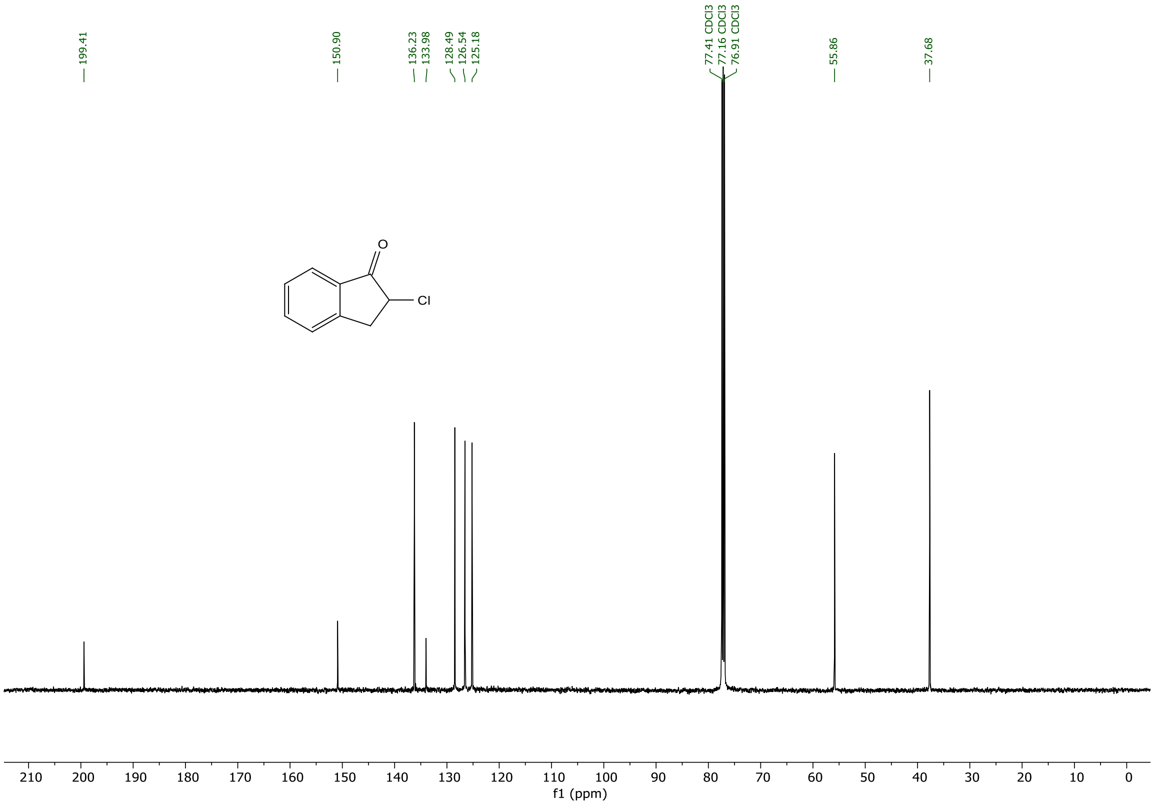

1-(6-(*Tert*-butyl)-3-chloro-1,1-dimethyl-2,3-dihydro-1H-inden-4-yl)ethan-1-one (13f)

^{1}H NMR (500 MHz, $CDCl_3$)

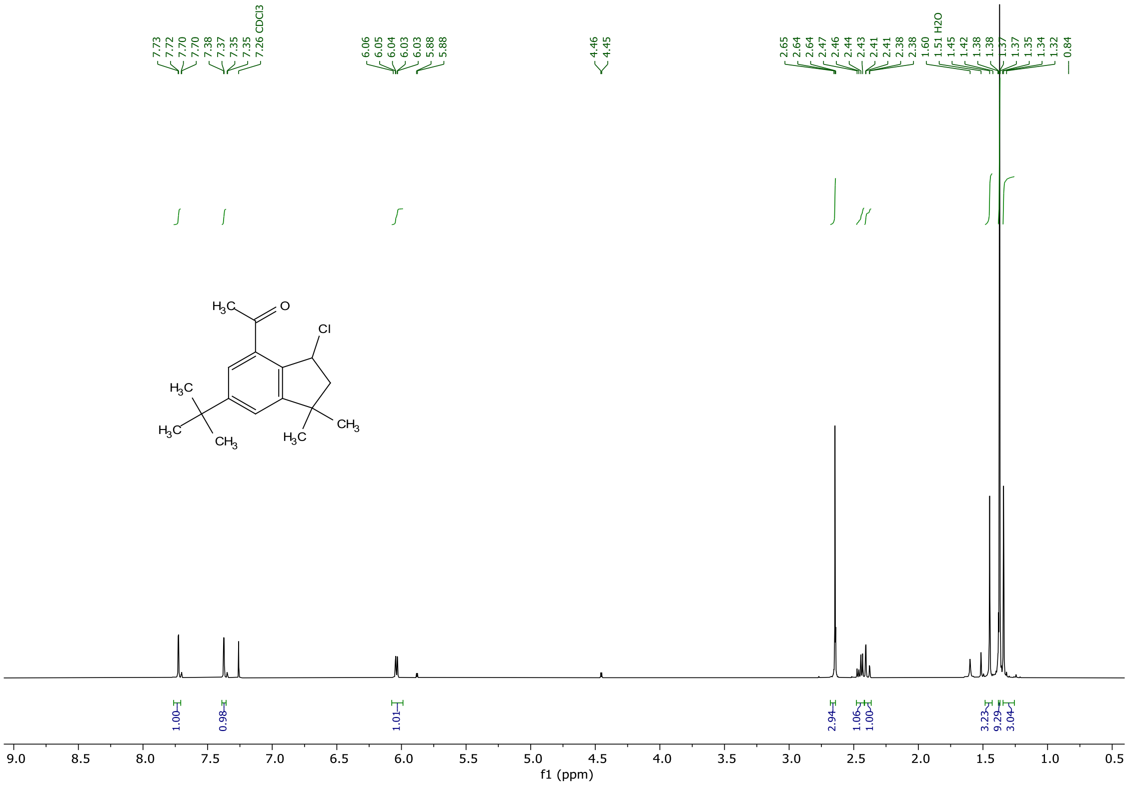

^{13}C NMR (126 MHz, $CDCl_3$)

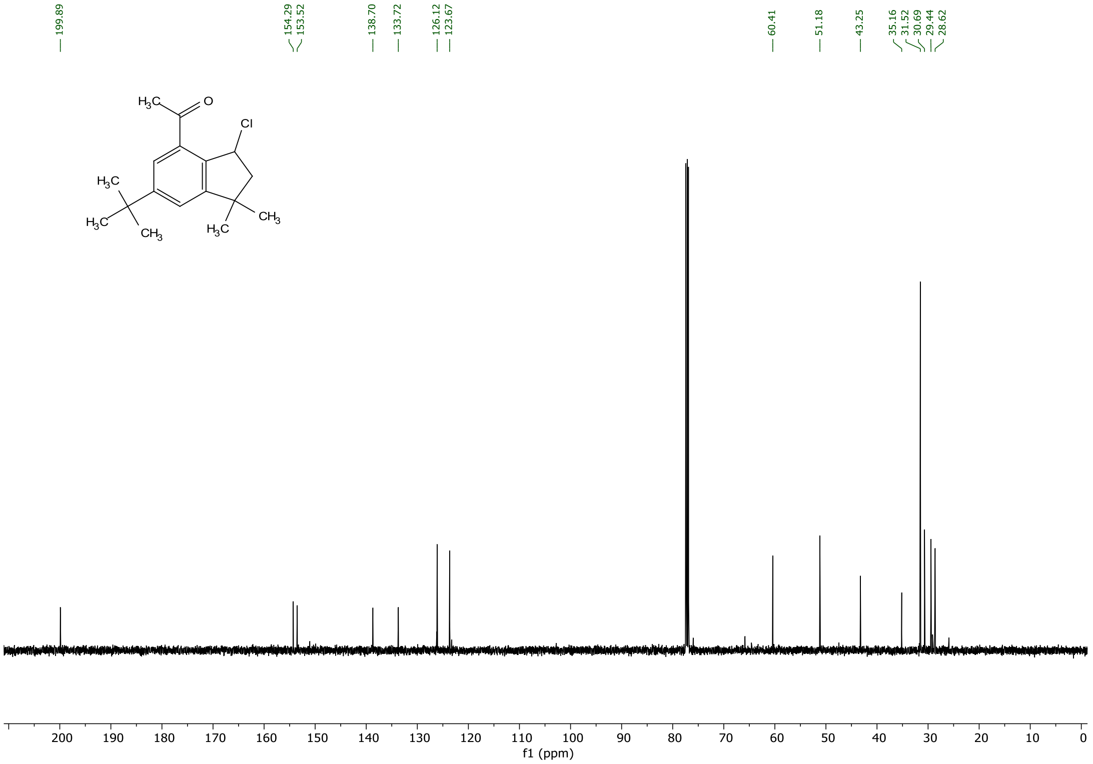

(2-(1-Chloroethyl)benzofuran-3-yl)(3,5-dibromo-4-hydroxyphenyl)methanone (13g)

^{1}H NMR (500 MHz, $CDCl_3$)

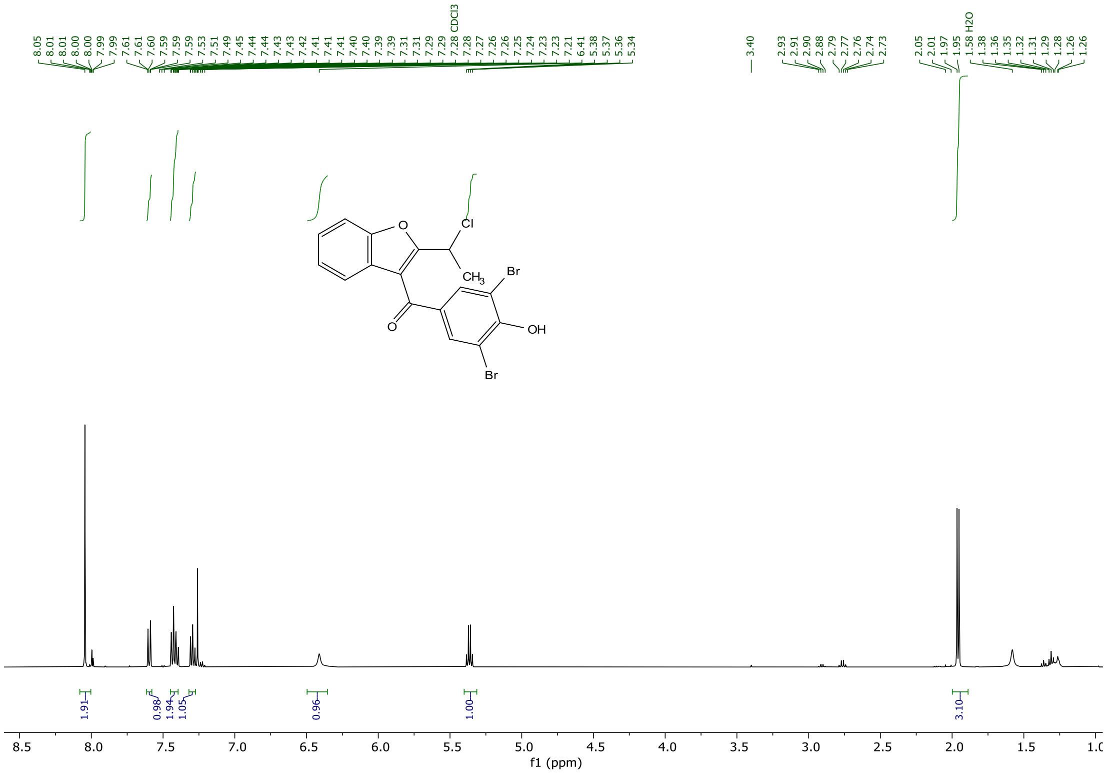

^{13}C NMR (126 MHz, $CDCl_3$)

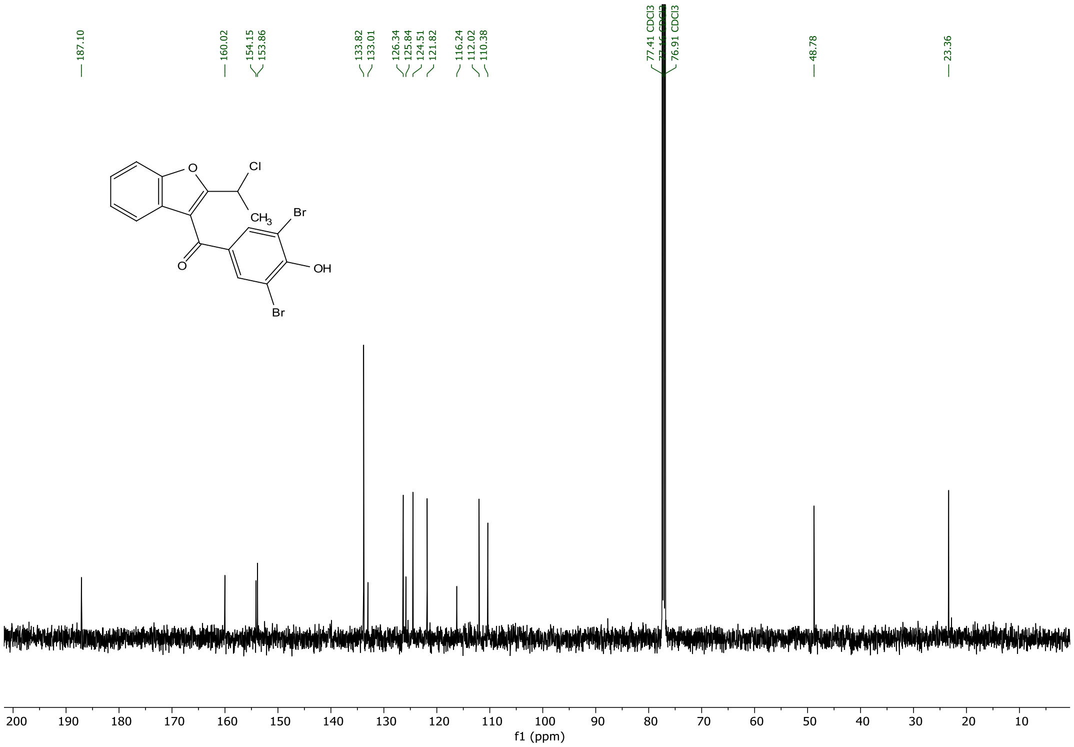

2-Chloro-3,4-dihydronaphthalen-1(2H)-one (13h) ^{1}H NMR (500 MHz, $CDCl_3$)

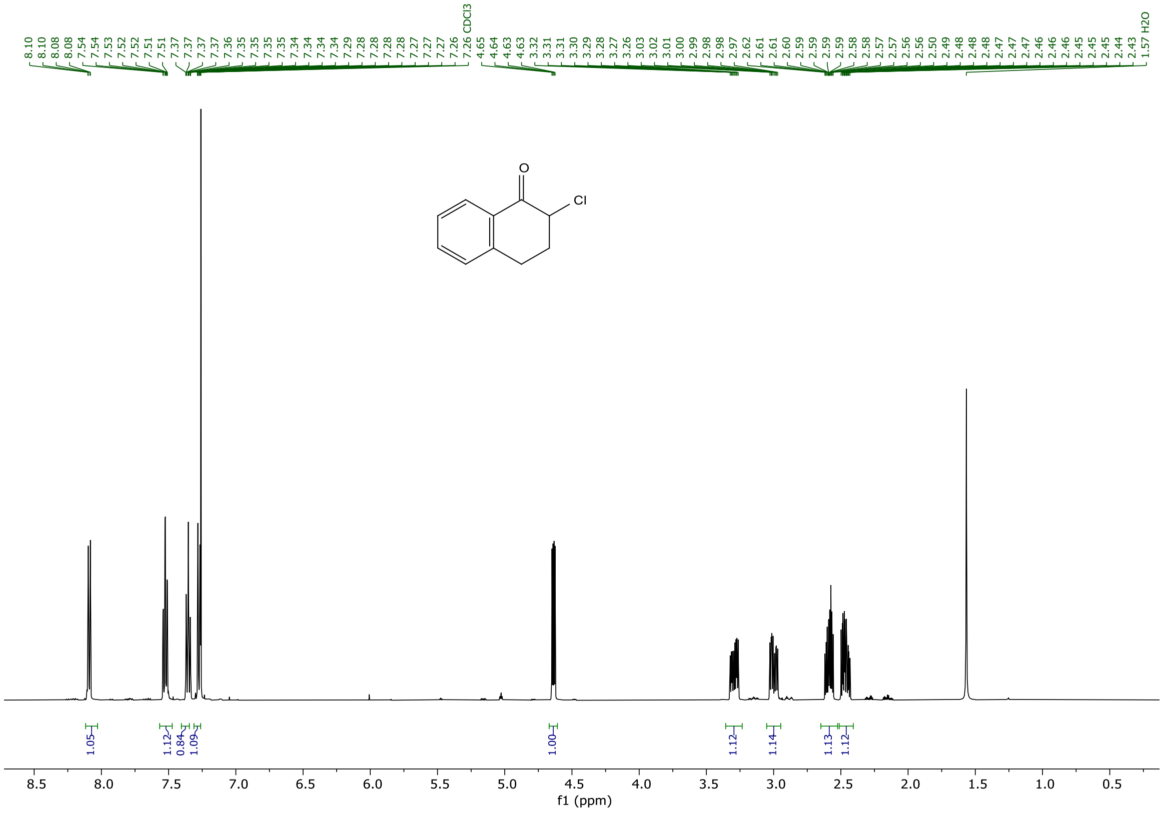

^{13}C NMR (126 MHz, $CDCl_3$)

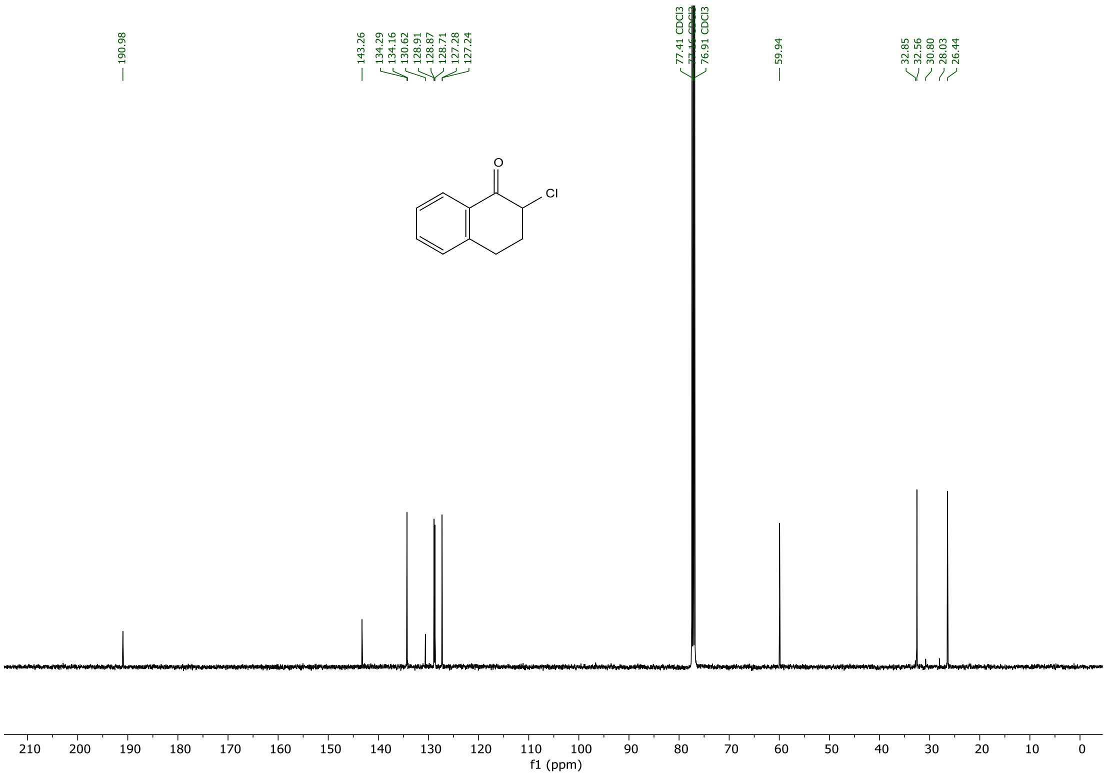

Methyl 2-(4-(1-chloro-2-methylpropyl)phenyl)propanoate (13i) ^{1}H NMR (500 MHz, $CDCl_3$)

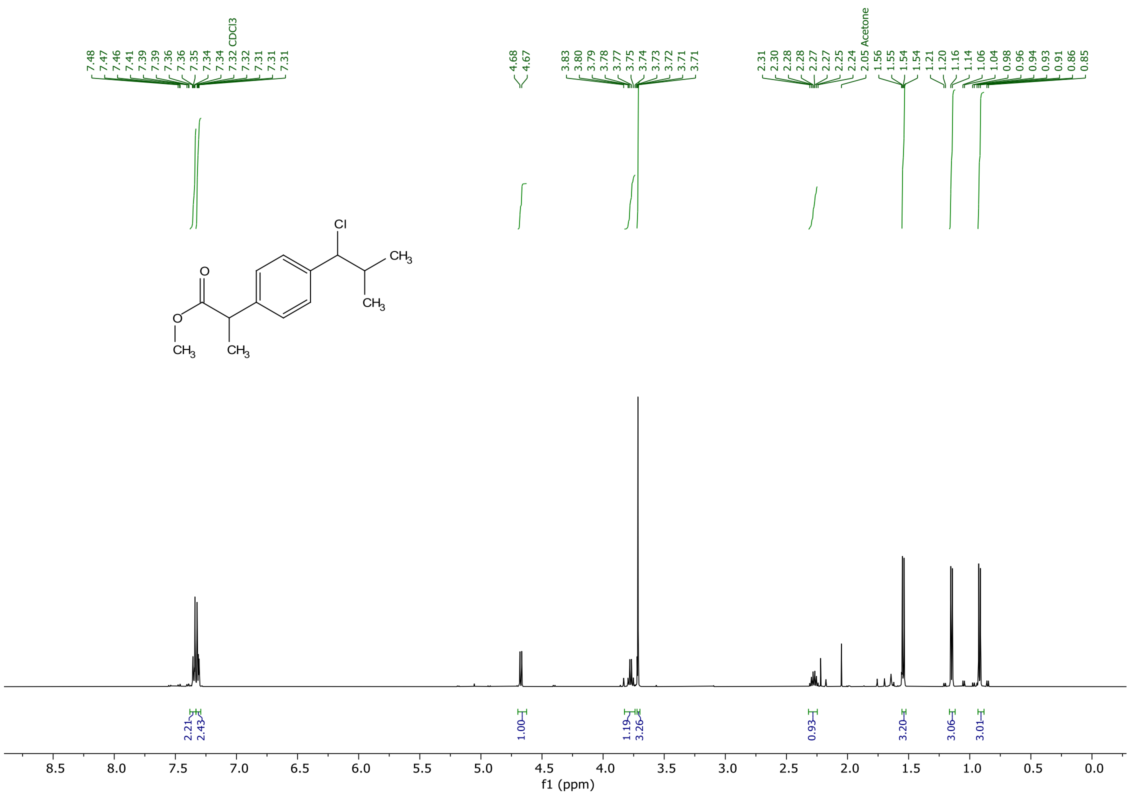

^{13}C NMR (126 MHz, $CDCl_3$)

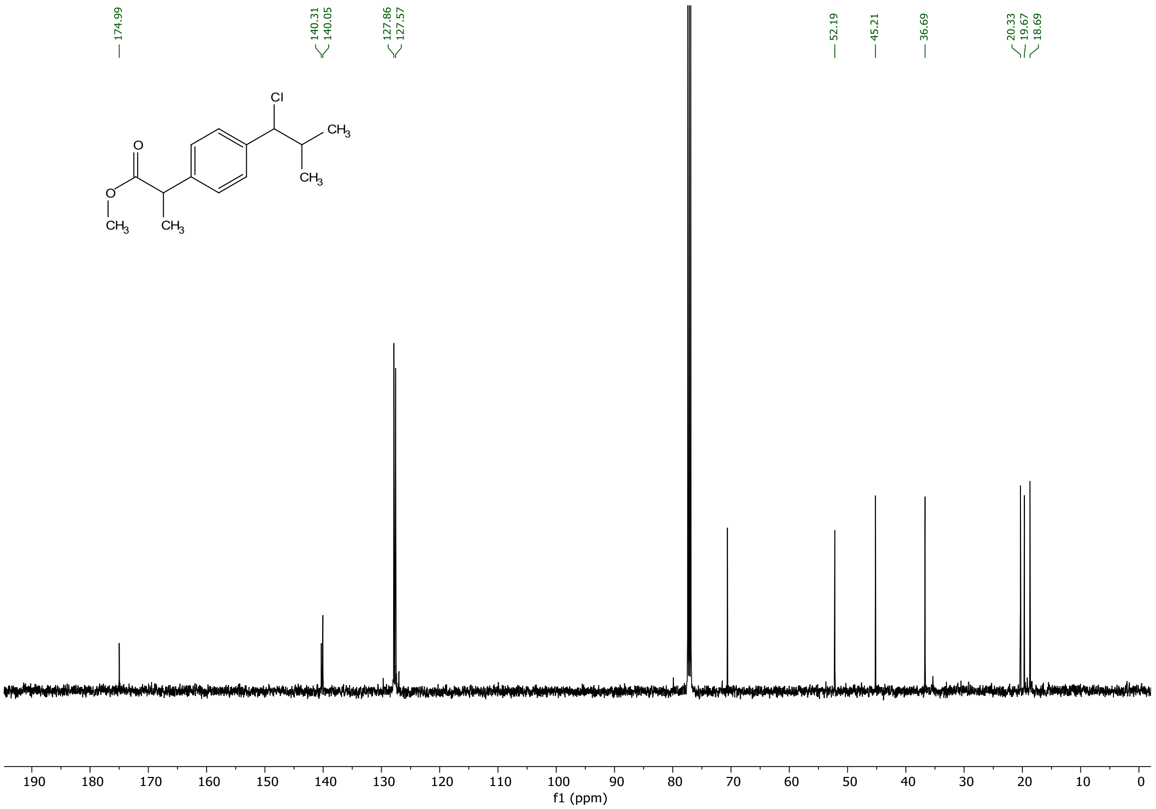

4-(3-Chloro-1-phenylpropyl)-1,1'-biphenyl (15a). ^{1}H NMR (500 MHz, $CDCl_3$)

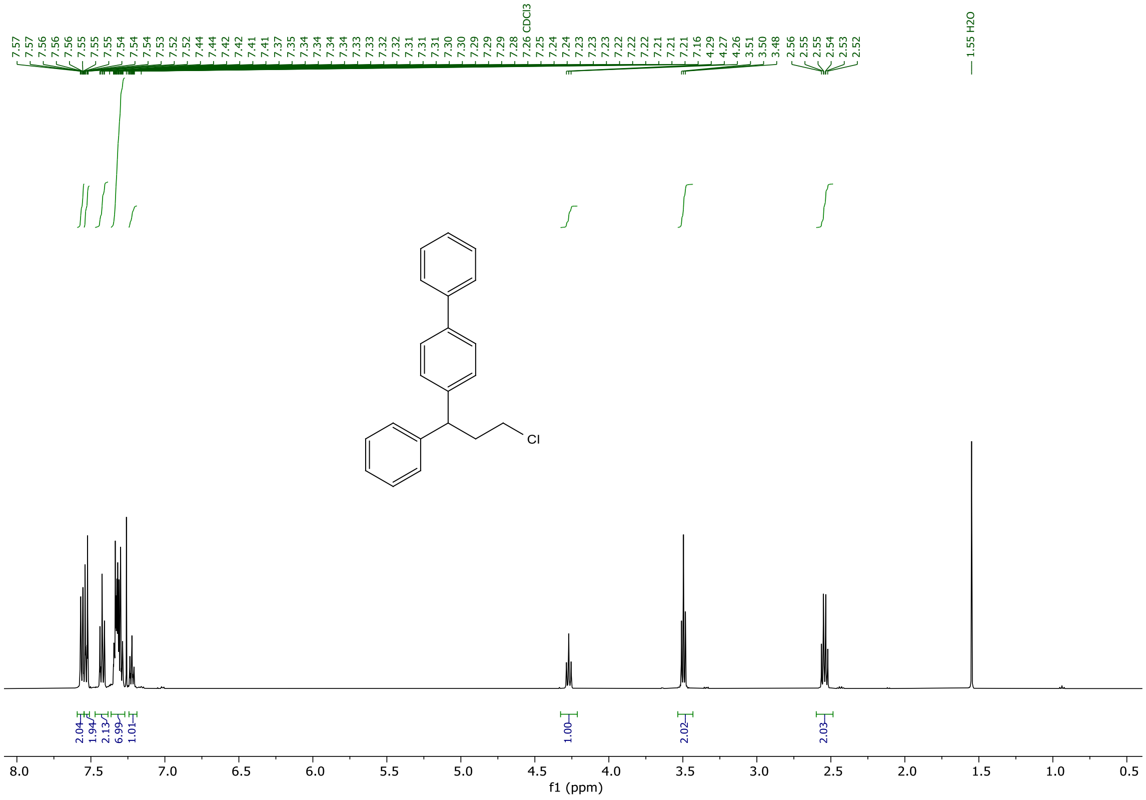

^{13}C NMR (126 MHz, $CDCl_3$)

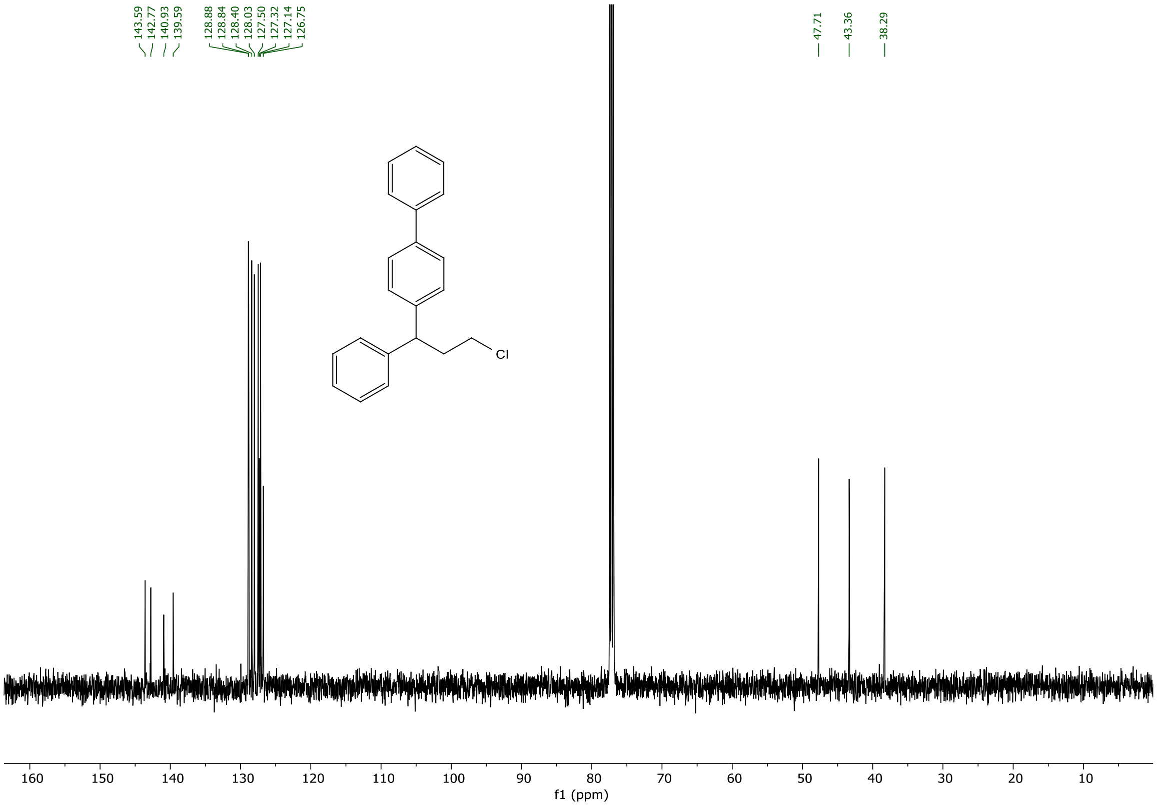

4-(1-(4-Bromophenyl)-3-chloropropyl)-1,1'-biphenyl (15b). ^{1}**H NMR** (500 MHz, CD_2Cl_2)

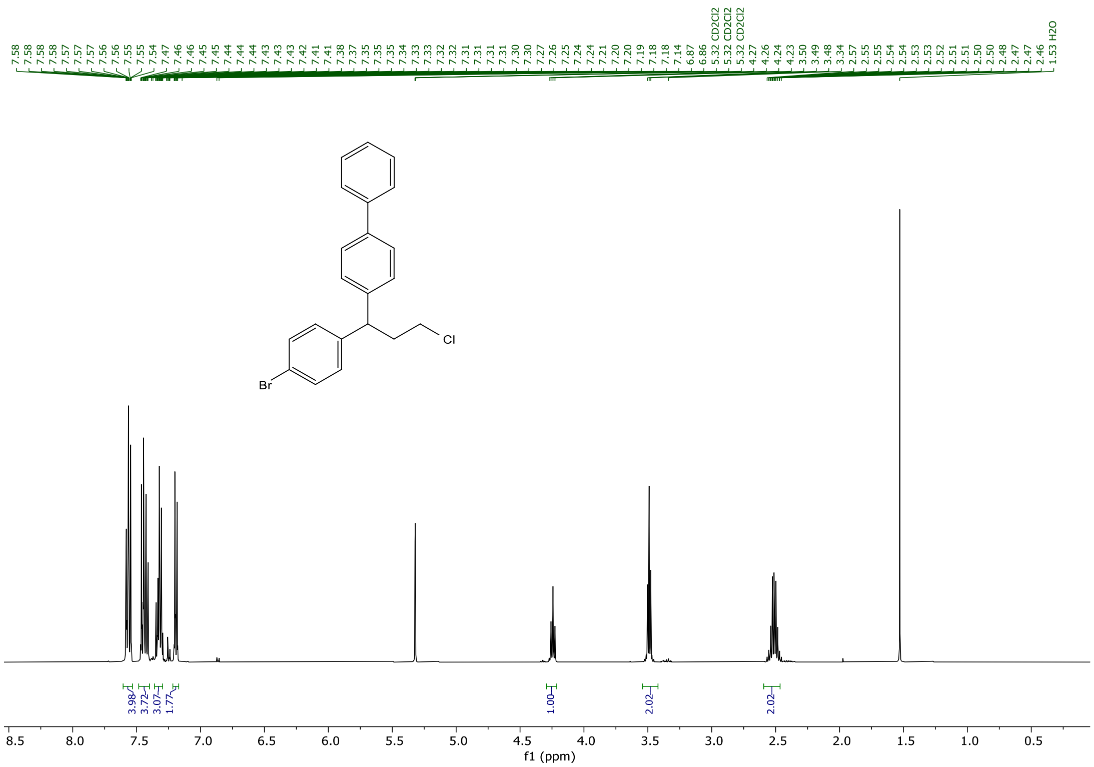

^{13}**C NMR** (126 MHz, CD_2Cl_2)

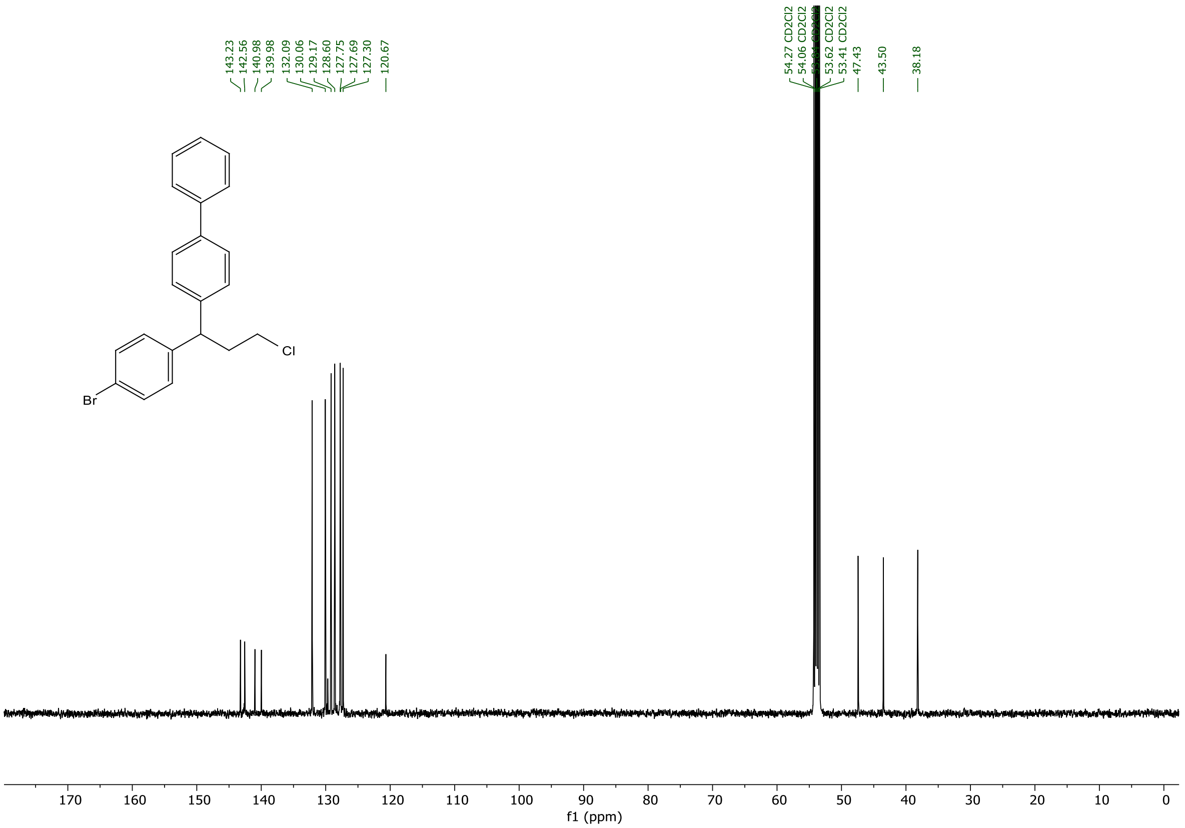

2-(4-(1-([1,1'-Biphenyl]-4-yl)-3-chloropropyl)phenyl)-4,4,5,5-tetramethyl-1,3,2-dioxaborolane (15c). ^{1}H NMR (500 MHz, $CDCl_3$)

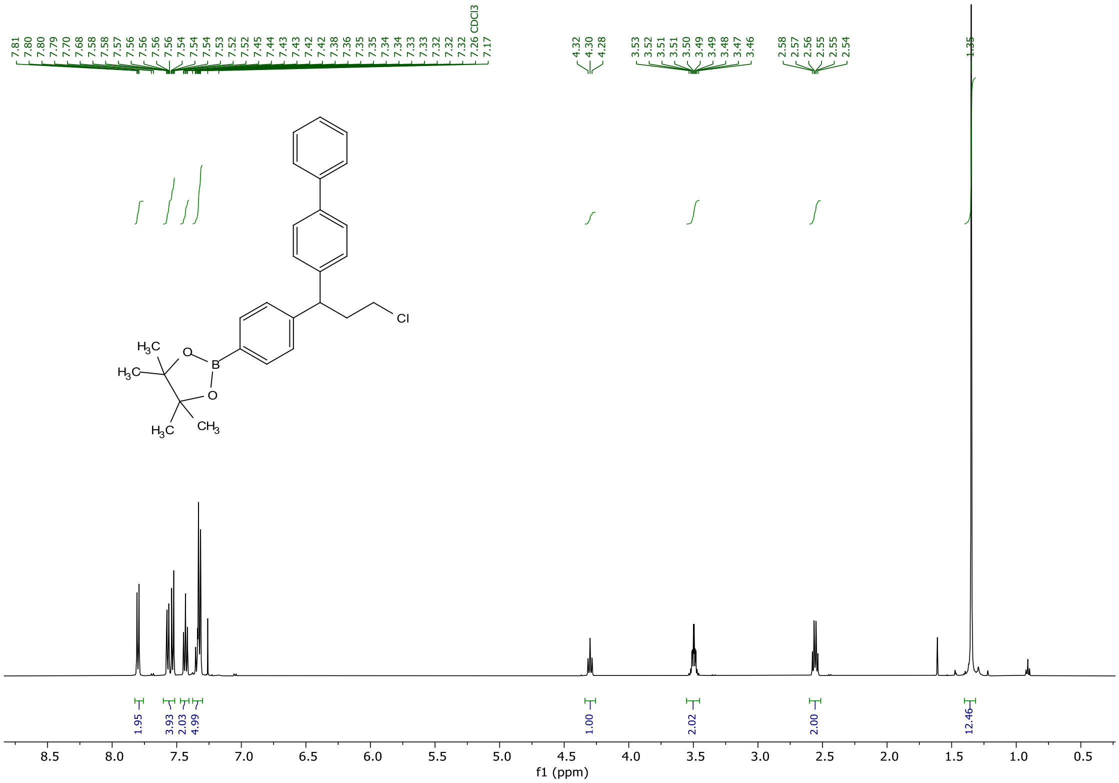

^{13}C NMR (126 MHz, $CDCl_3$)

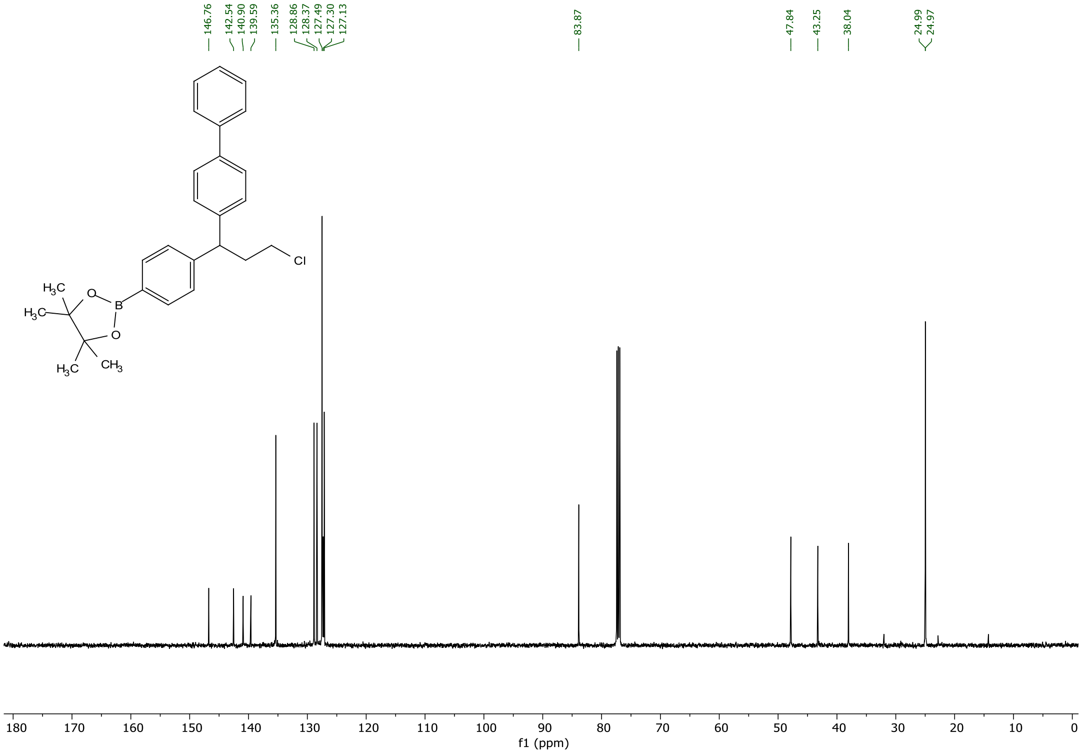

1-(4-(1-([1,1'-Biphenyl]-4-yl)-3-chloropropyl)phenyl)ethan-1-one (15d).

^{1}H NMR (500 MHz, $CDCl_3$)

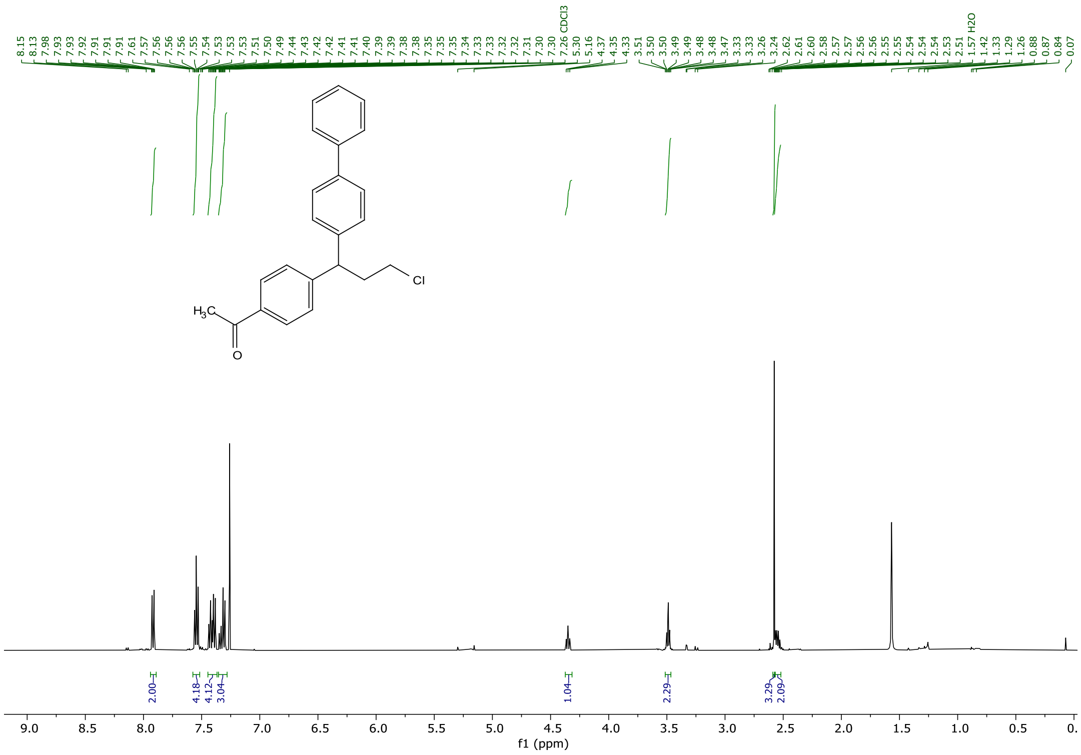

^{13}C NMR (126 MHz, $CDCl_3$)

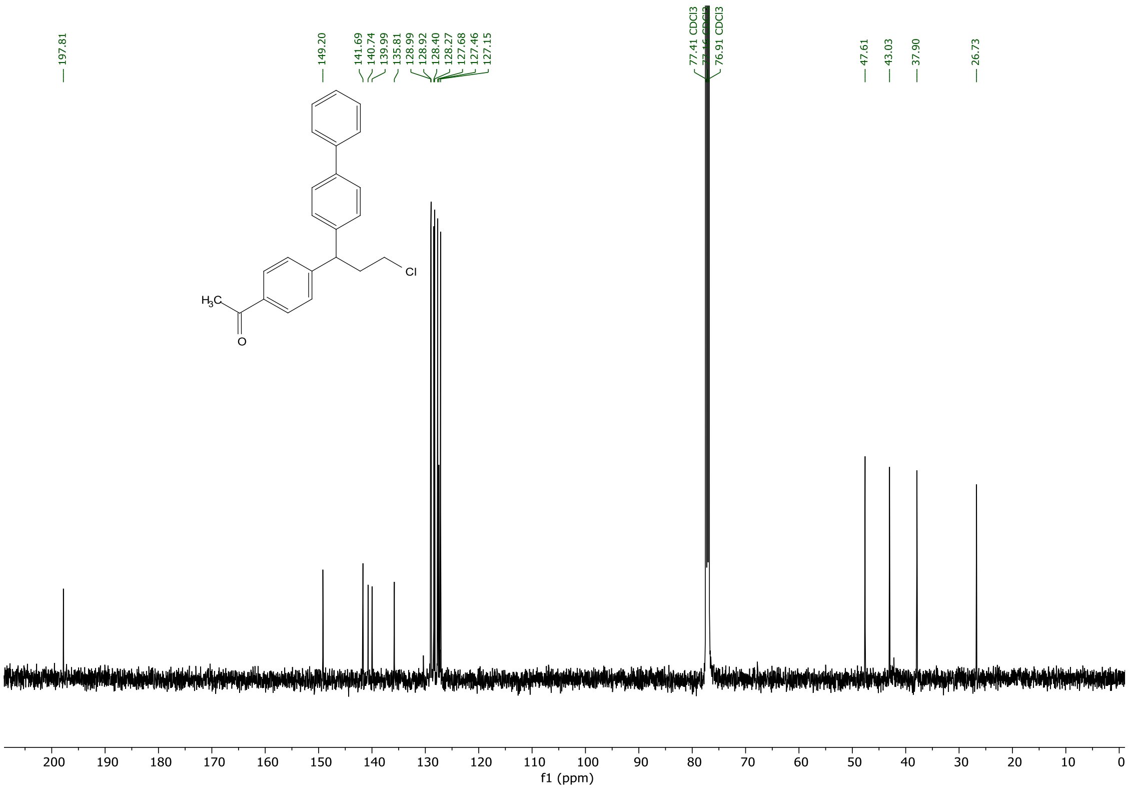

2-(1-([1,1'-Biphenyl]-4-yl)-3-chloropropyl)naphthalene (15e). ^{1}**H NMR** (500 MHz, CD_2Cl_2)

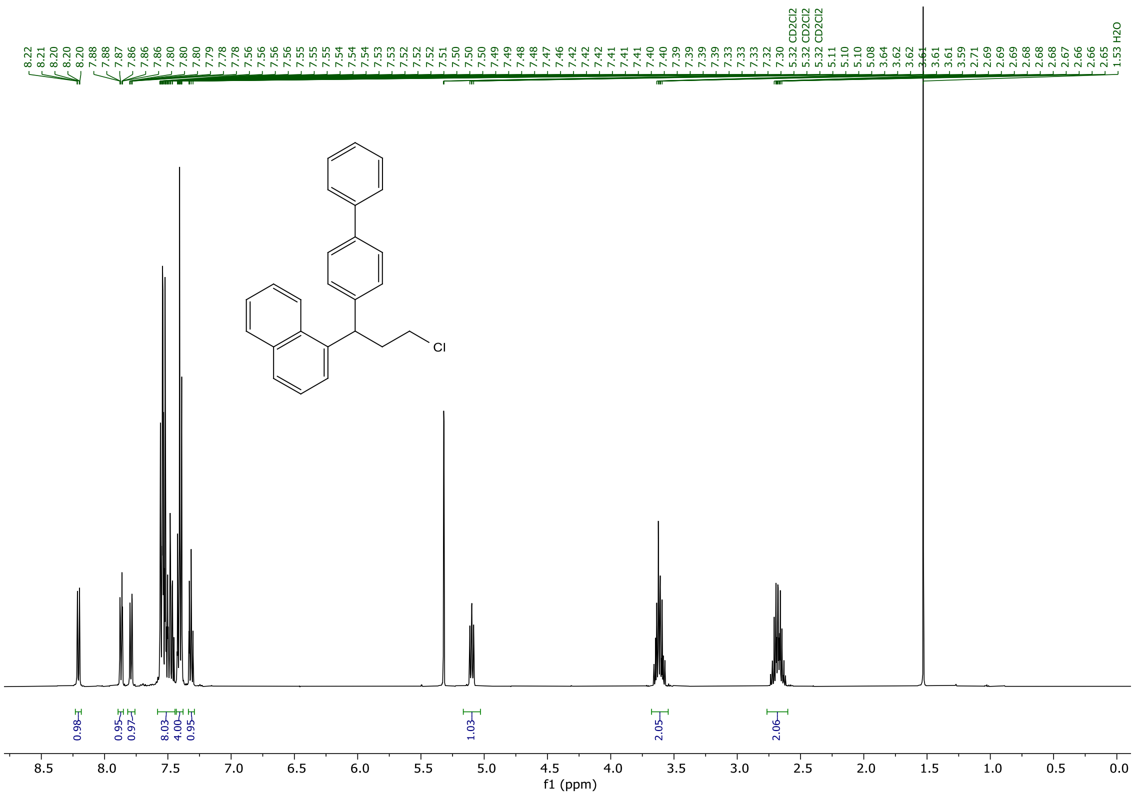

^{13}**C NMR** (126 MHz, CD_2Cl_2)

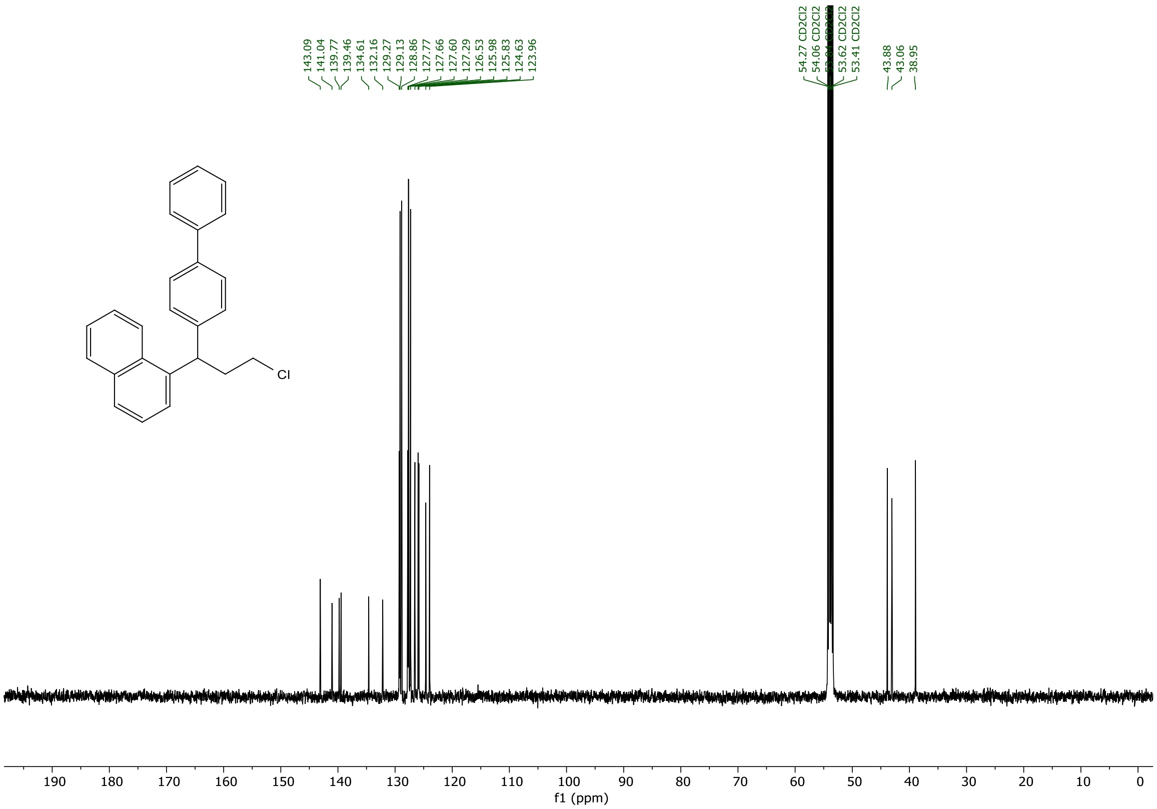

4-(3-Chloro-1-phenylpropyl)phenol (15f1). ^{1}H NMR (500 MHz, $CDCl_3$)

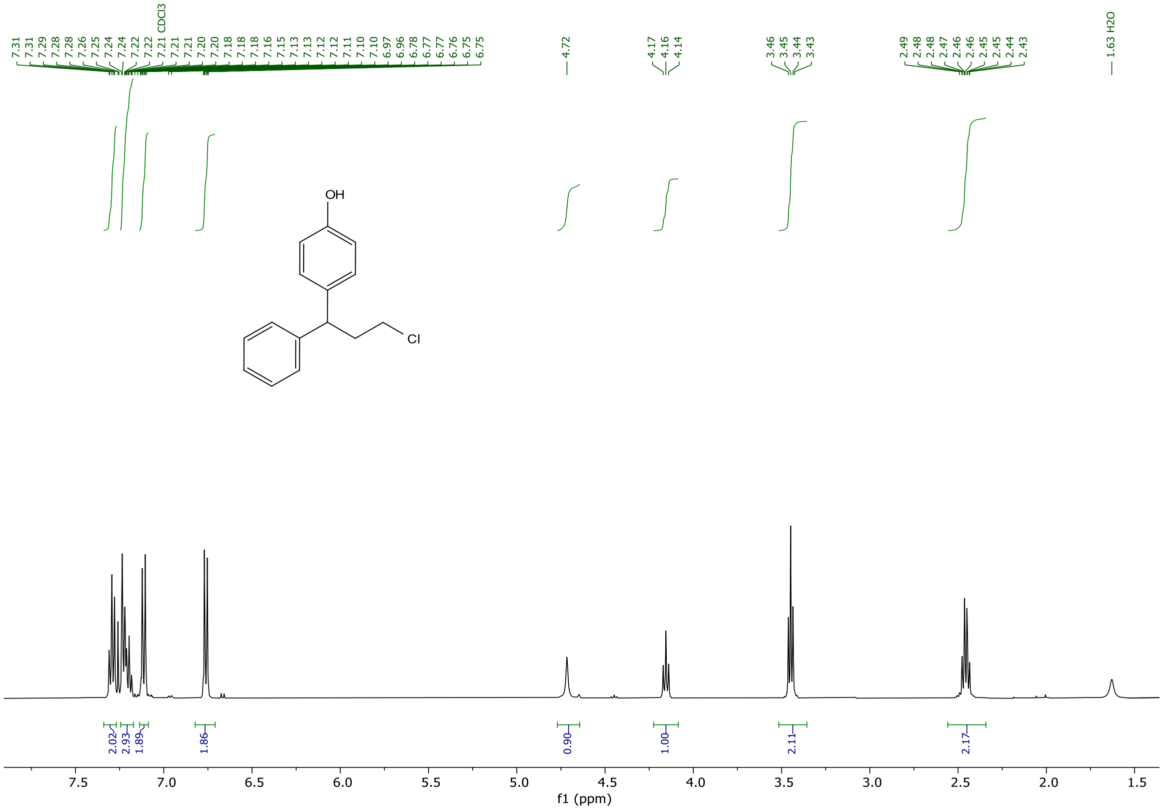

^{13}C NMR (126 MHz, $CDCl_3$)

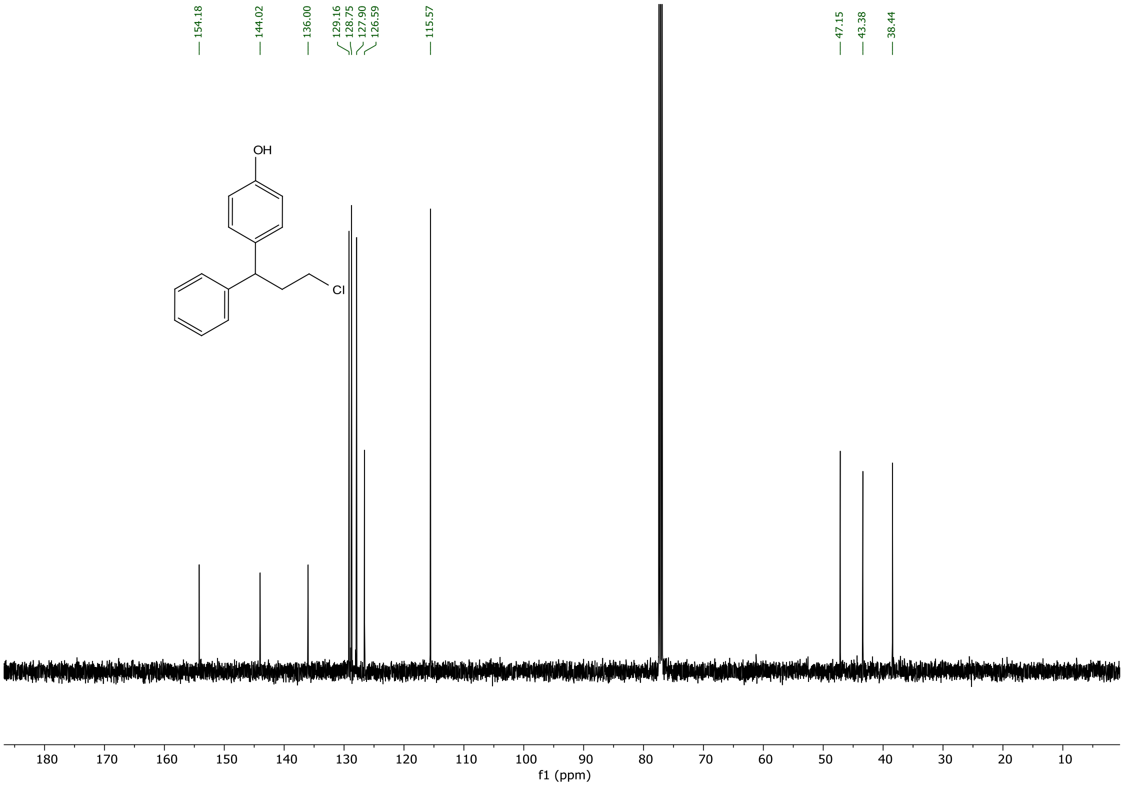

2-(3-Chloro-1-phenylpropyl)phenol (15f2) . **^{1}H NMR (**500 MHz, $CDCl_3$)

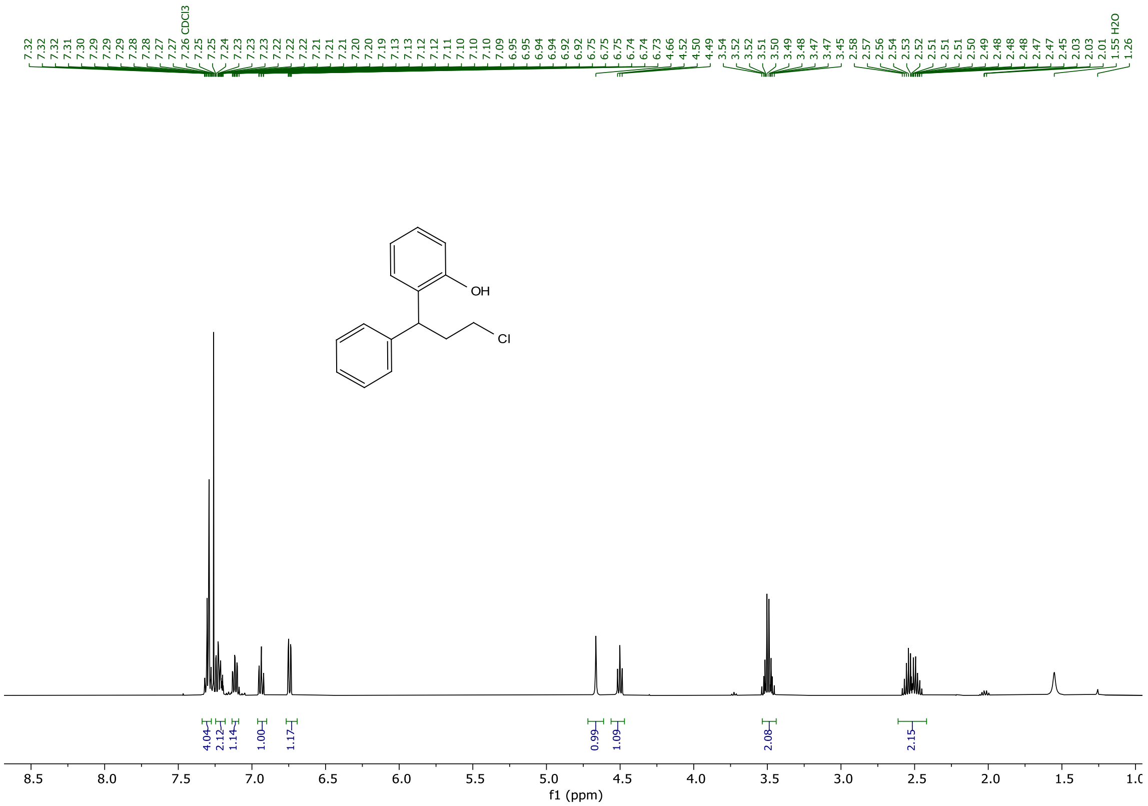

^{13}C NMR (126 MHz, $CDCl_3$)

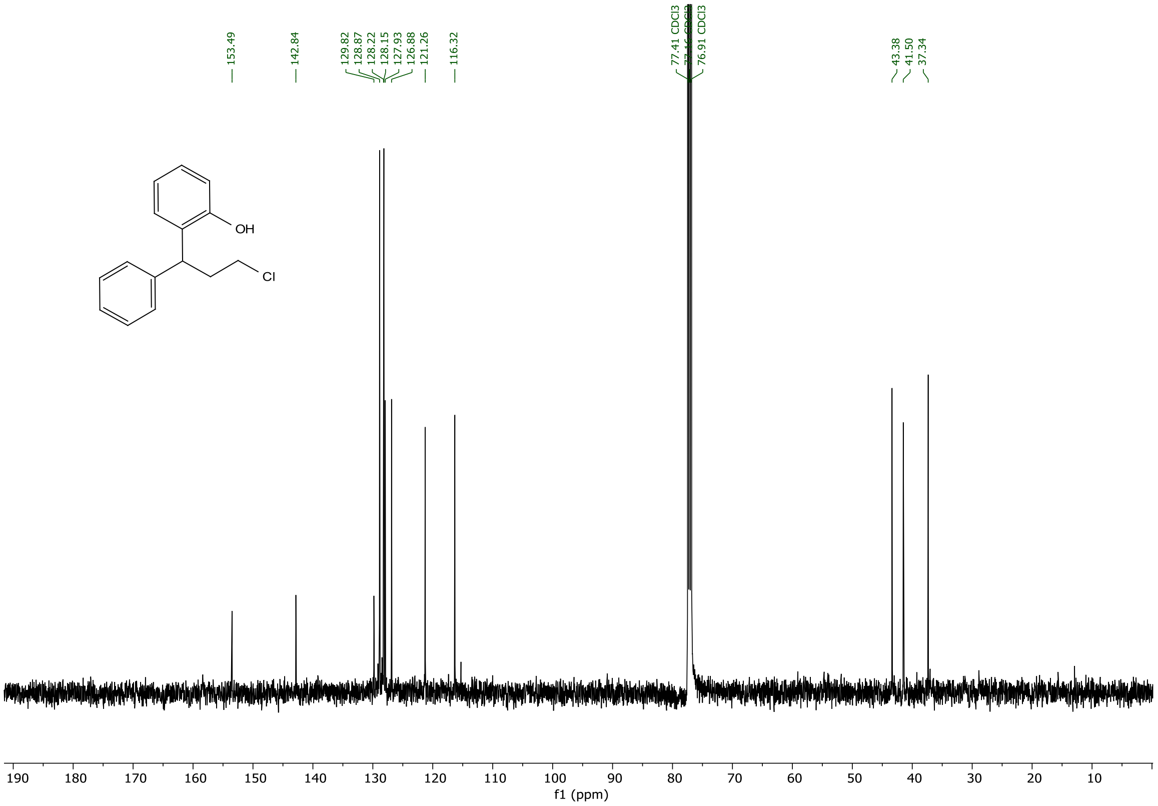

5-(3-Chloro-1-phenylpropyl)benzo[*d*][1,3]dioxole (15g). ^{1}H NMR (500 MHz, $CDCl_3$)

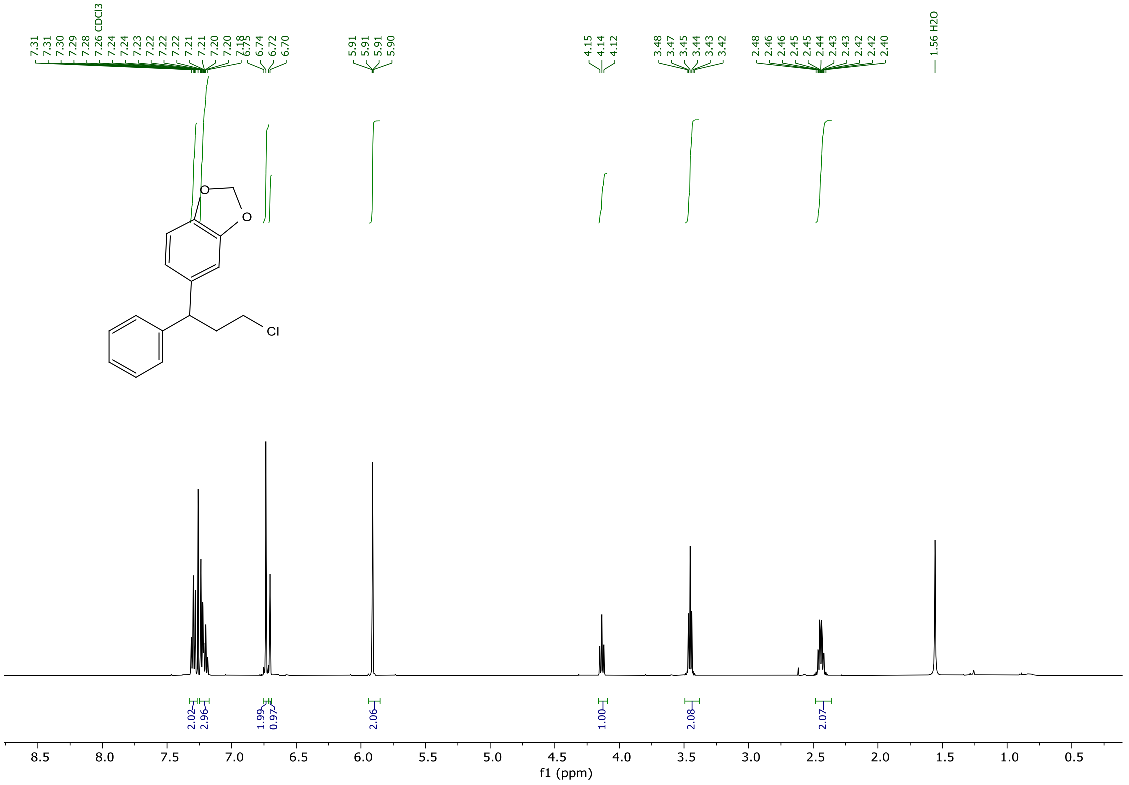

^{13}C NMR (126 MHz, $CDCl_3$)

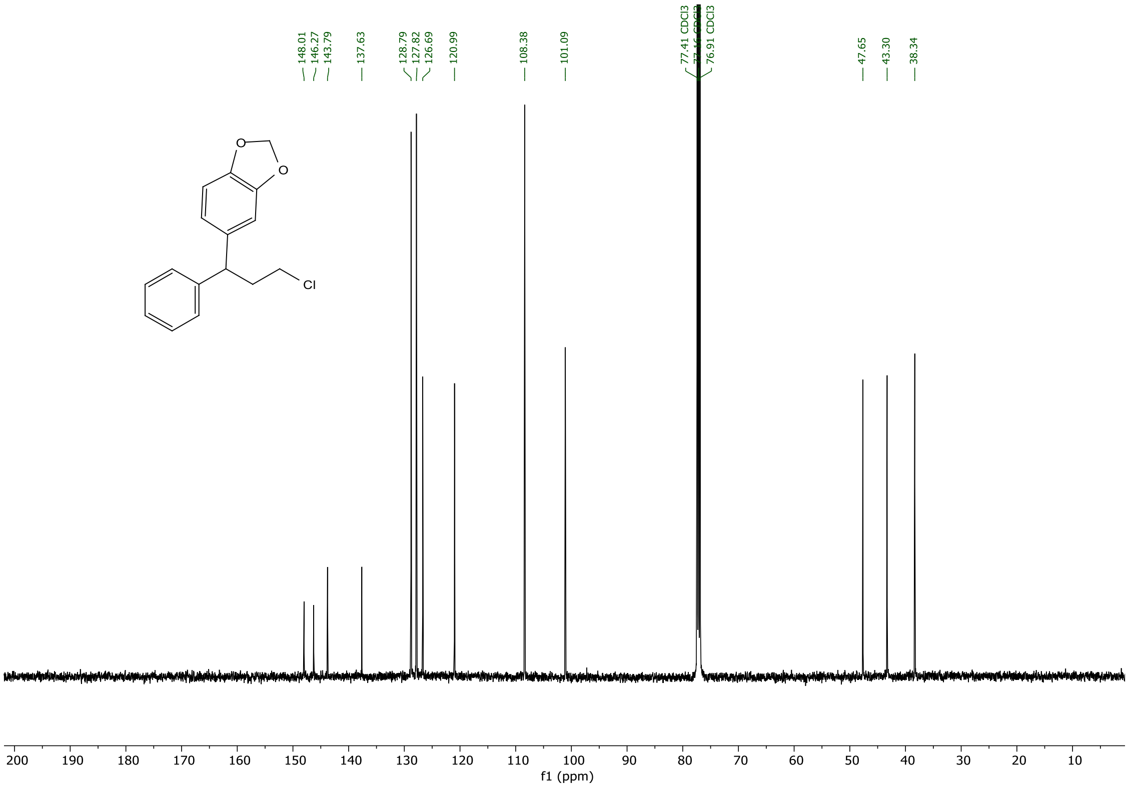

3-(3-Chloro-1-phenylpropyl)benzo[b]thiophene (15h) ^{1}H NMR (500 MHz, $CDCl_3$)

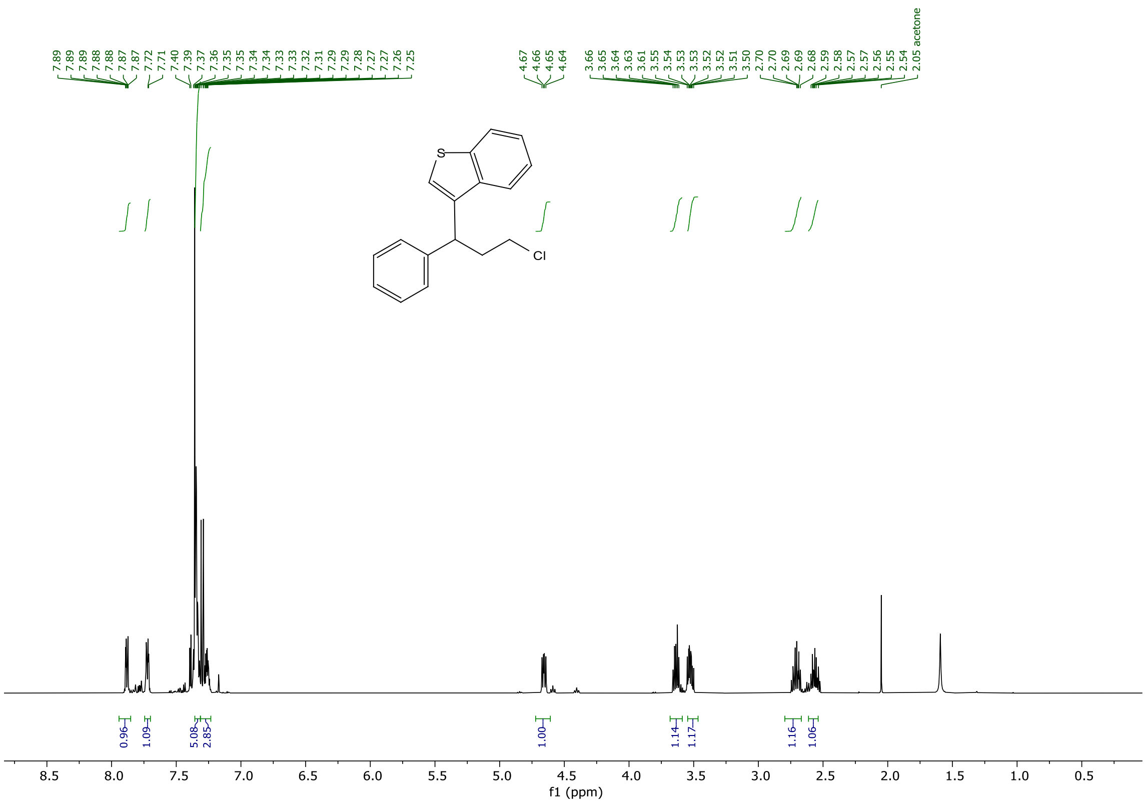

^{13}C NMR (126 MHz, $CDCl_3$)

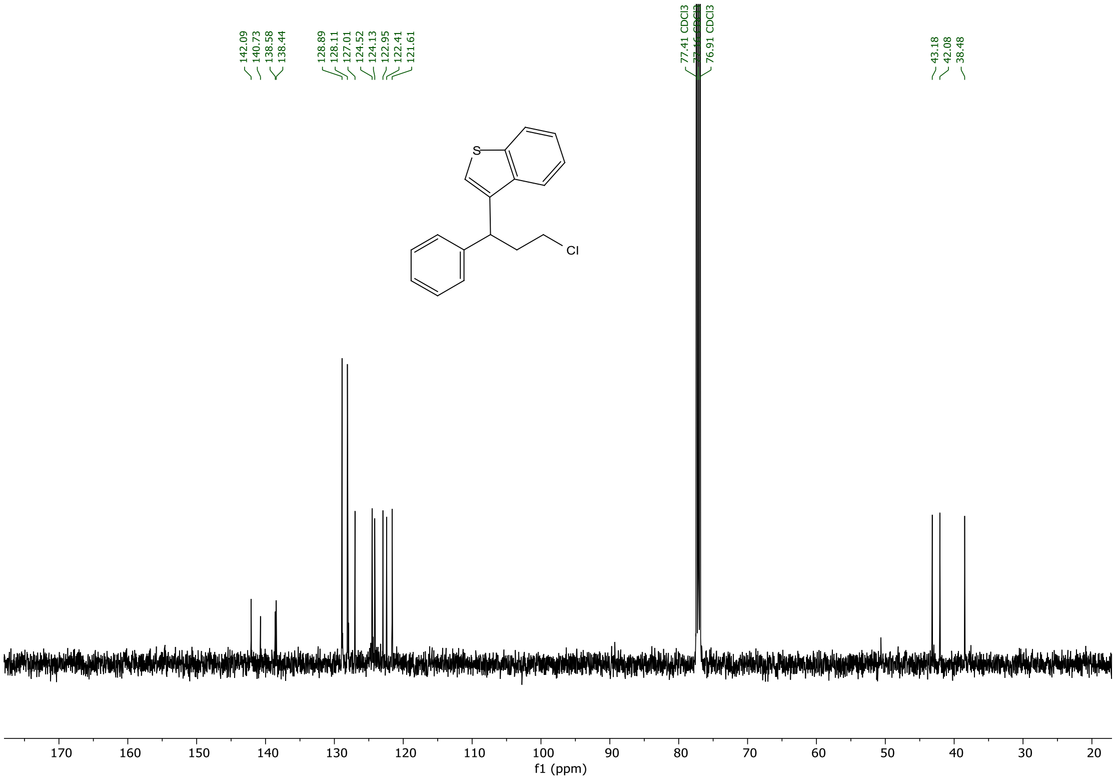

Methyl 2-(6-(3-chloro-1-phenylpropyl)-5-methoxy-2-methyl-1H-indol-3-yl)acetate (15i).

^{1}H NMR (500 MHz, $CDCl_3$)

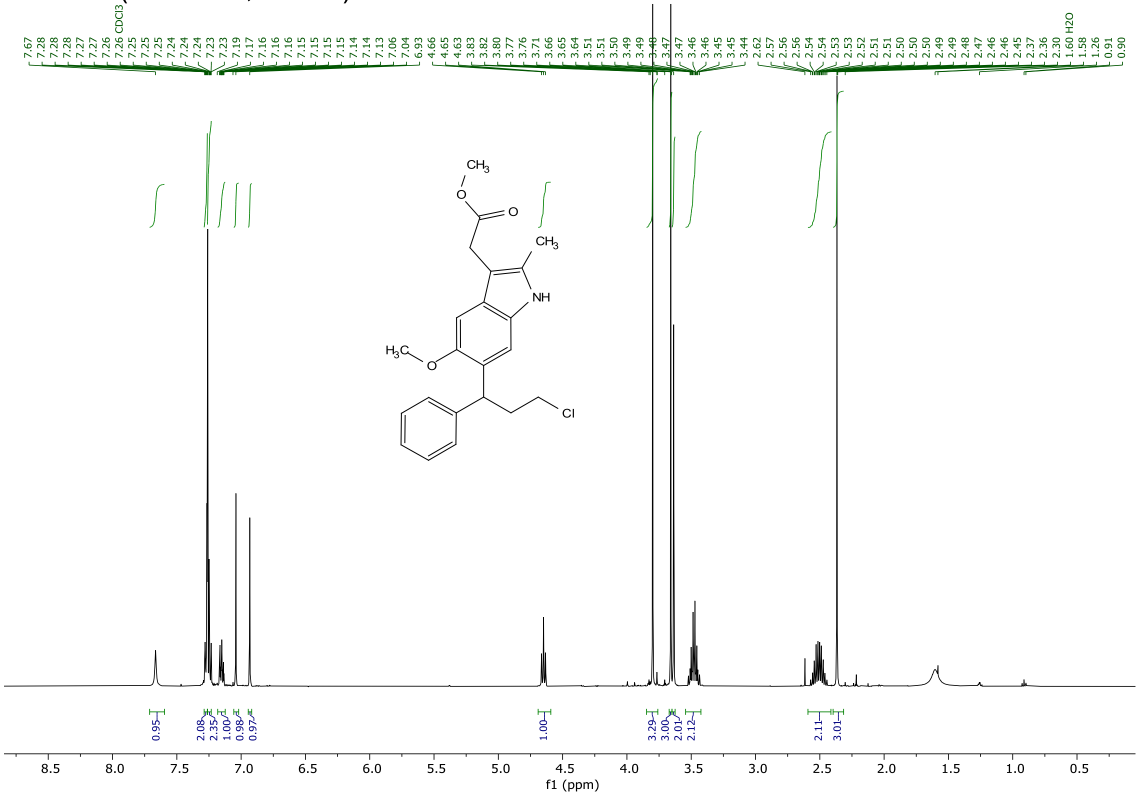

^{13}C NMR (126 MHz, $CDCl_3$)

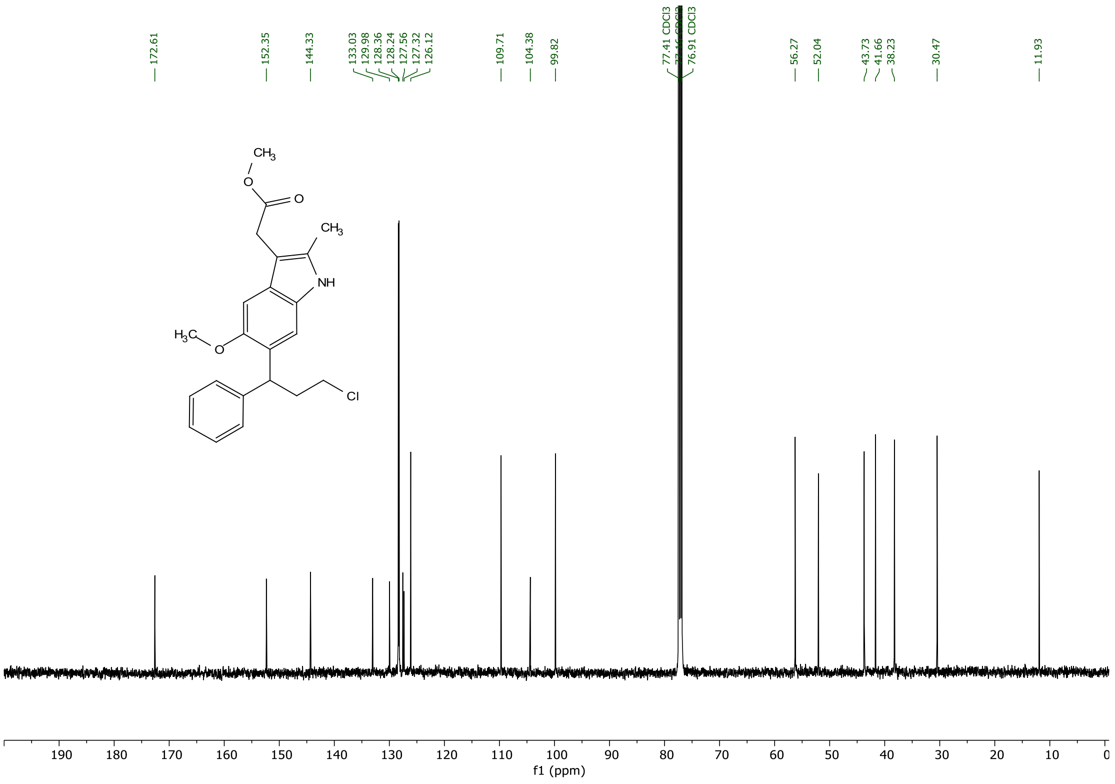

N-(2-(4-(3-Chloro-1-phenylpropyl)phenoxy)-4-nitrophenyl)methanesulfonamide (15j)

^{1}H NMR (500 MHz, $CDCl_3$)

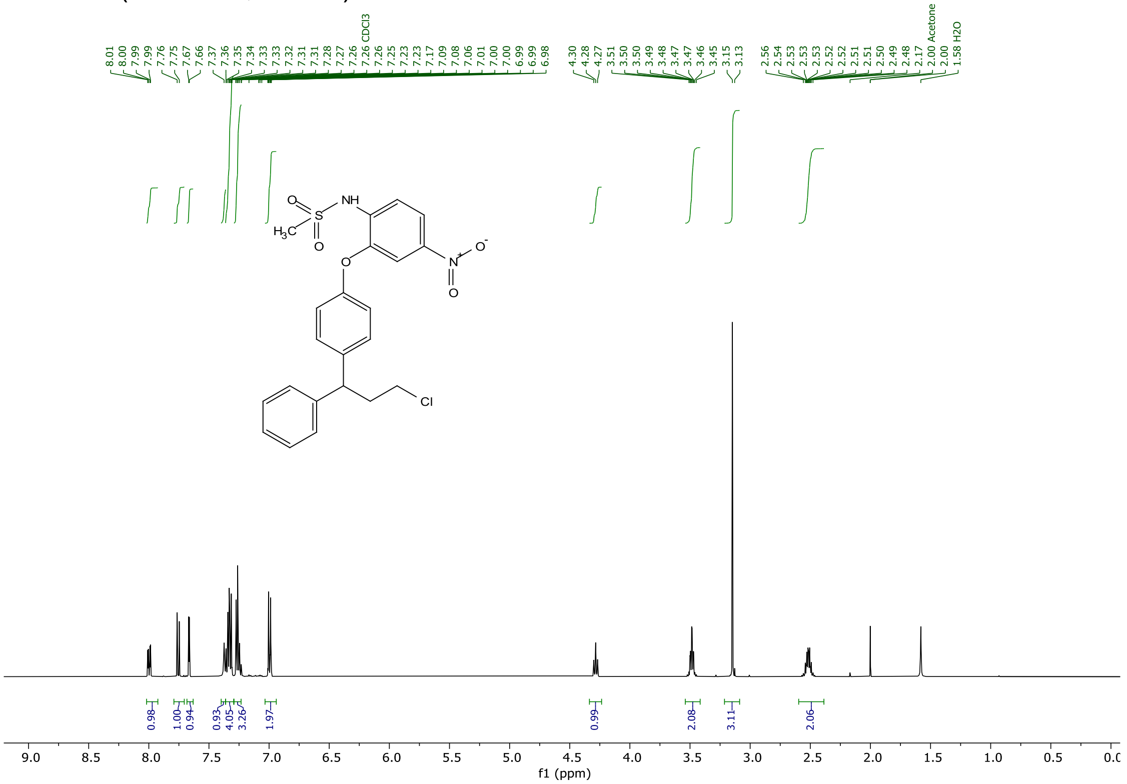

^{13}C NMR (126 MHz, $CDCl_3$)

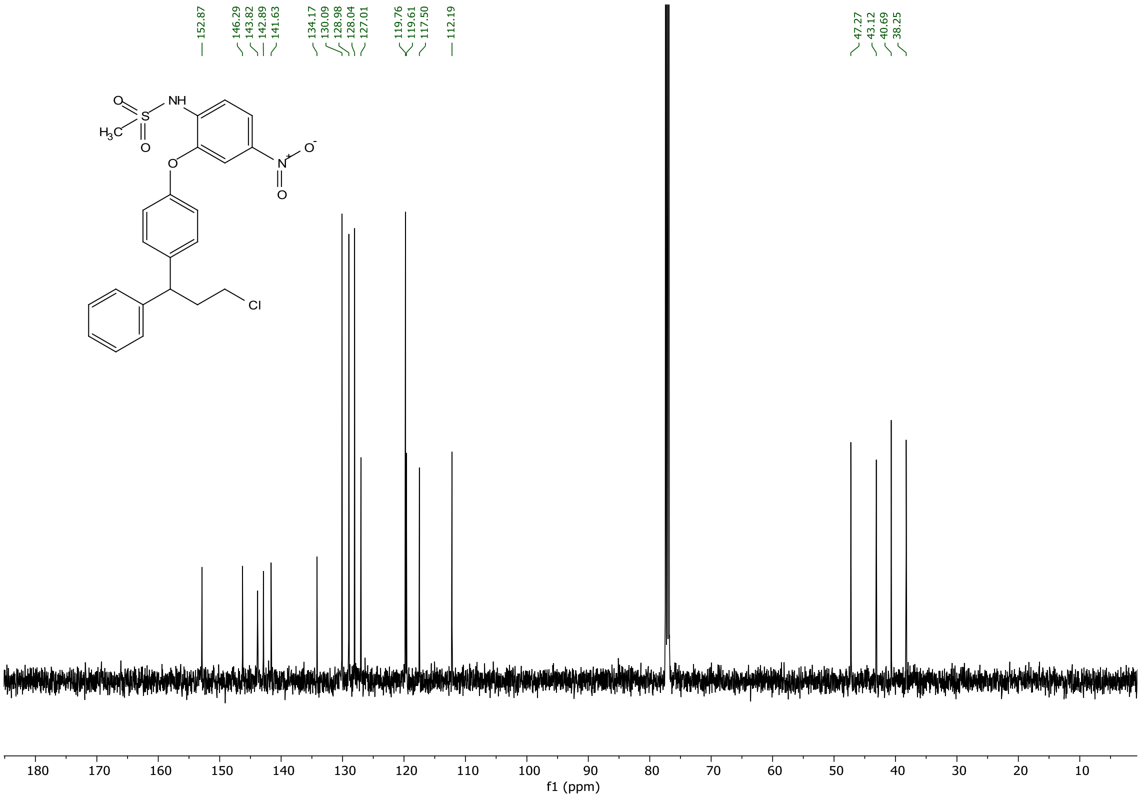

(8R,9S,13S,14S)-2-(3-Chloro-1-phenylpropyl)-3-methoxy-13-methyl-6,7,8,9,11,12,13,14,15,16-decahydro-17H-cyclopenta[a]phenanthren-17-one (15k)

^{1}H NMR (500 MHz, $CDCl_3$) mixture of two diastereomers

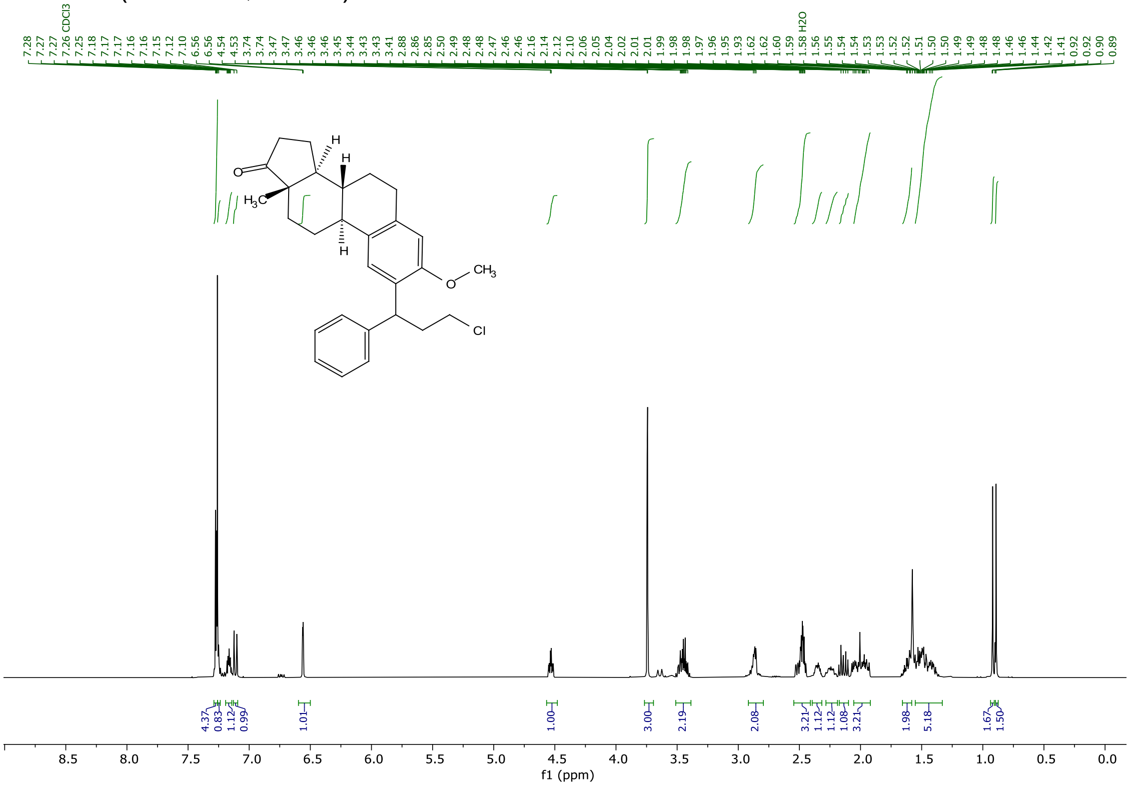

^{13}C NMR for two diastereomer (126 MHz, $CDCl_3$)

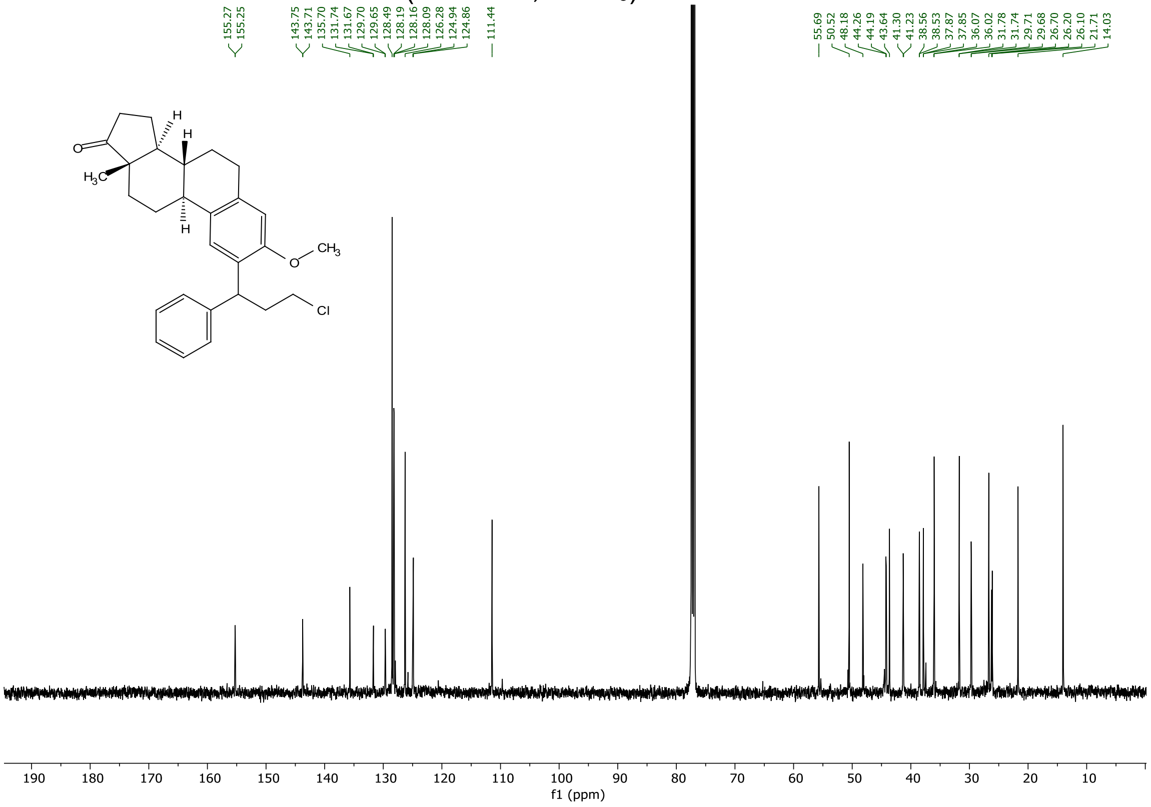

N-(3-chloro-1-phenylpropyl)-4-methoxybenzenesulfonamide (15l). ^{1}H NMR (500 MHz, $CDCl_3$)

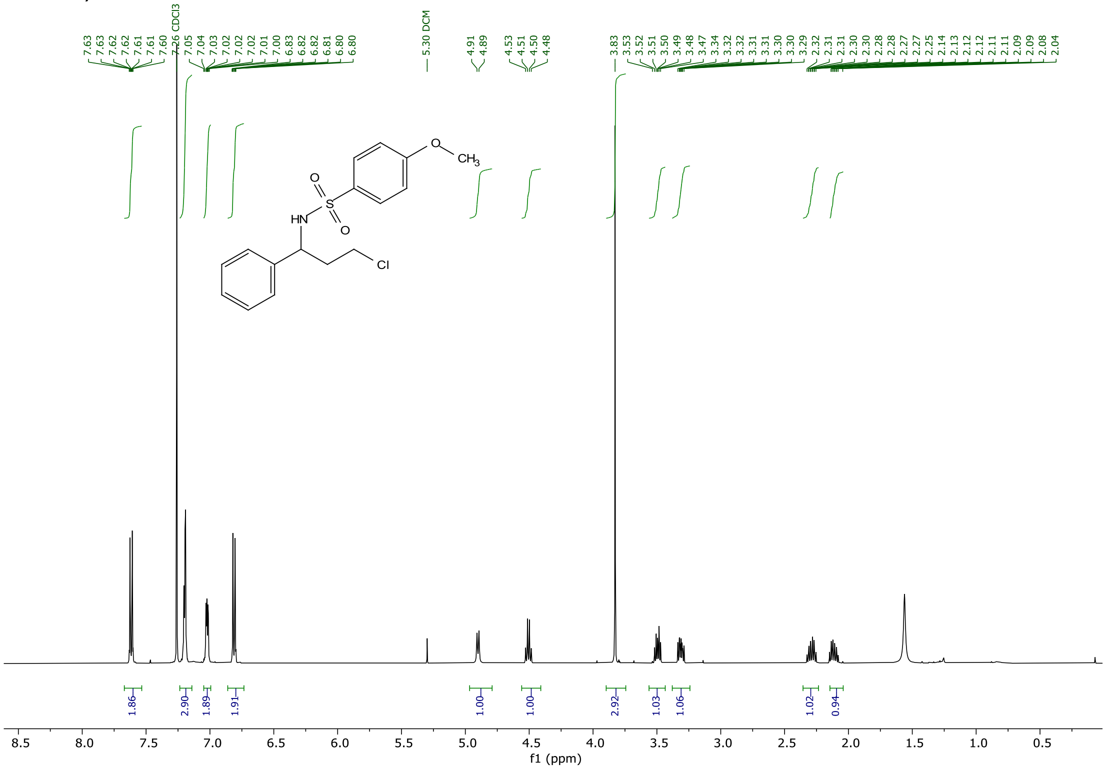

^{13}C NMR (126 MHz, $CDCl_3$)

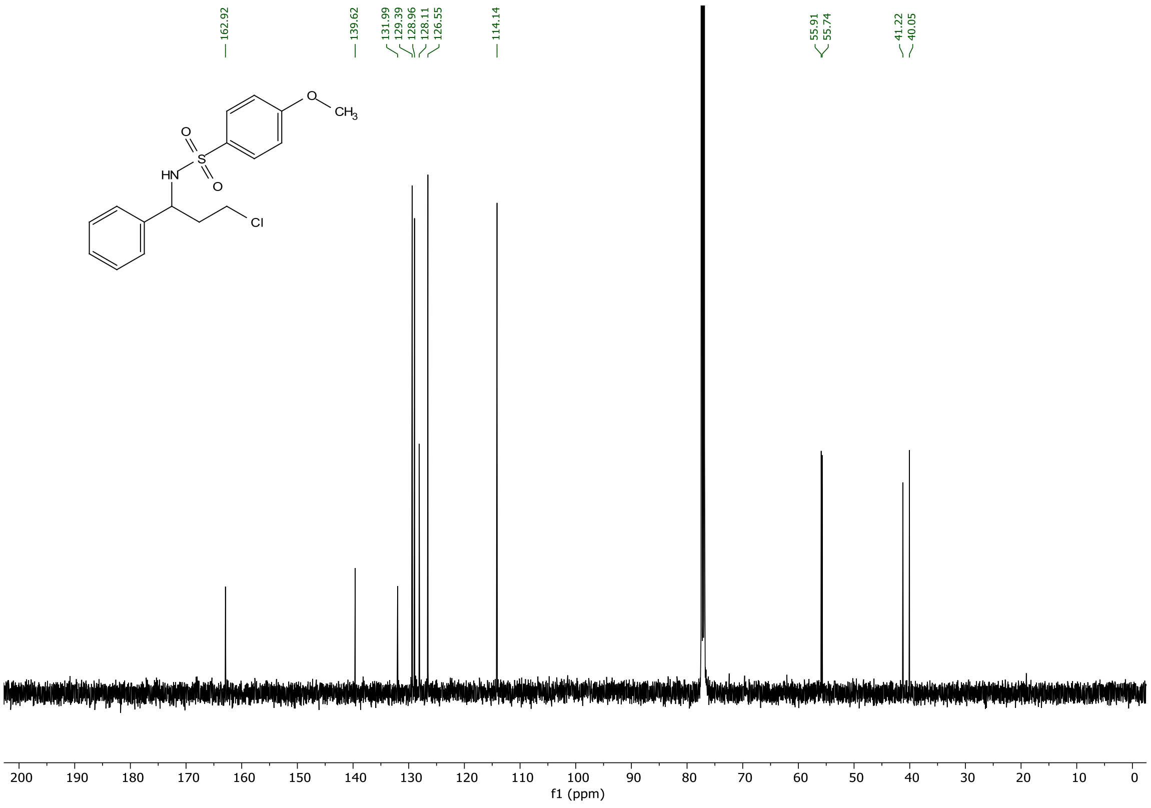

N-(3-Chloro-1-phenylpropyl)-4-(5-(p-tolyl)-3-(trifluoromethyl)-1H-pyrazol-1-yl)benzenesulfonamide (15m) ^{1}**H NMR** (500 MHz, $CDCl_3$)

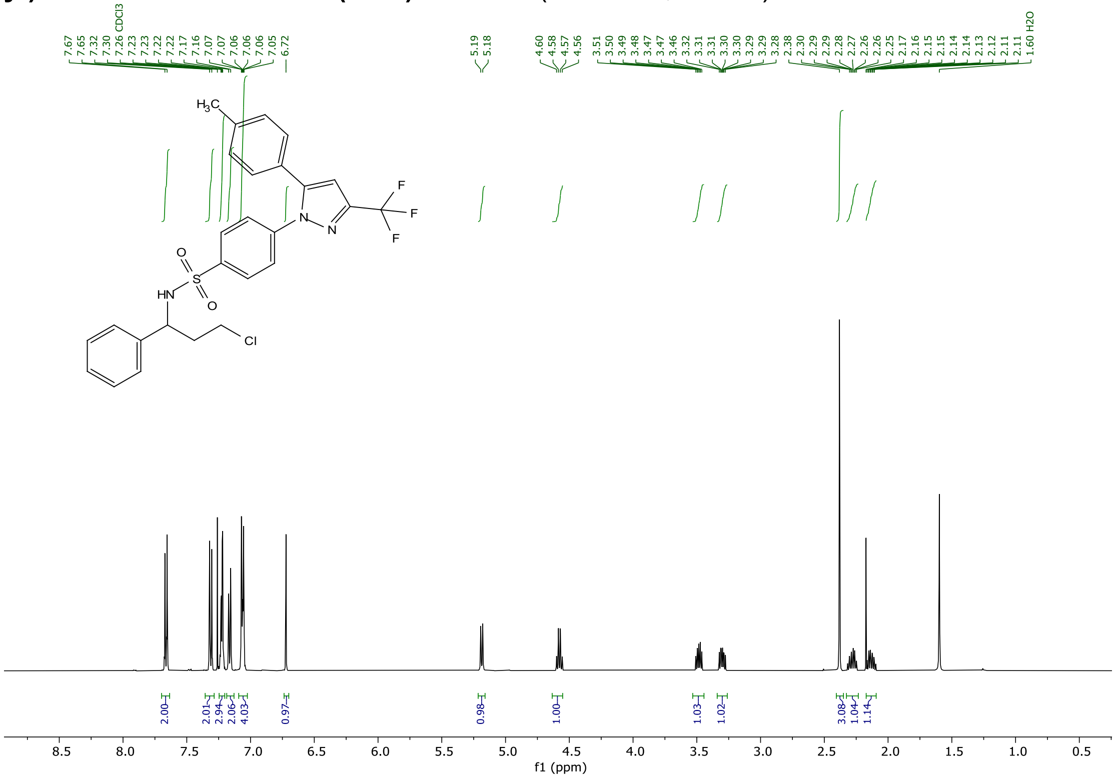

^{13}**C NMR** (126 MHz, $CDCl_3$)

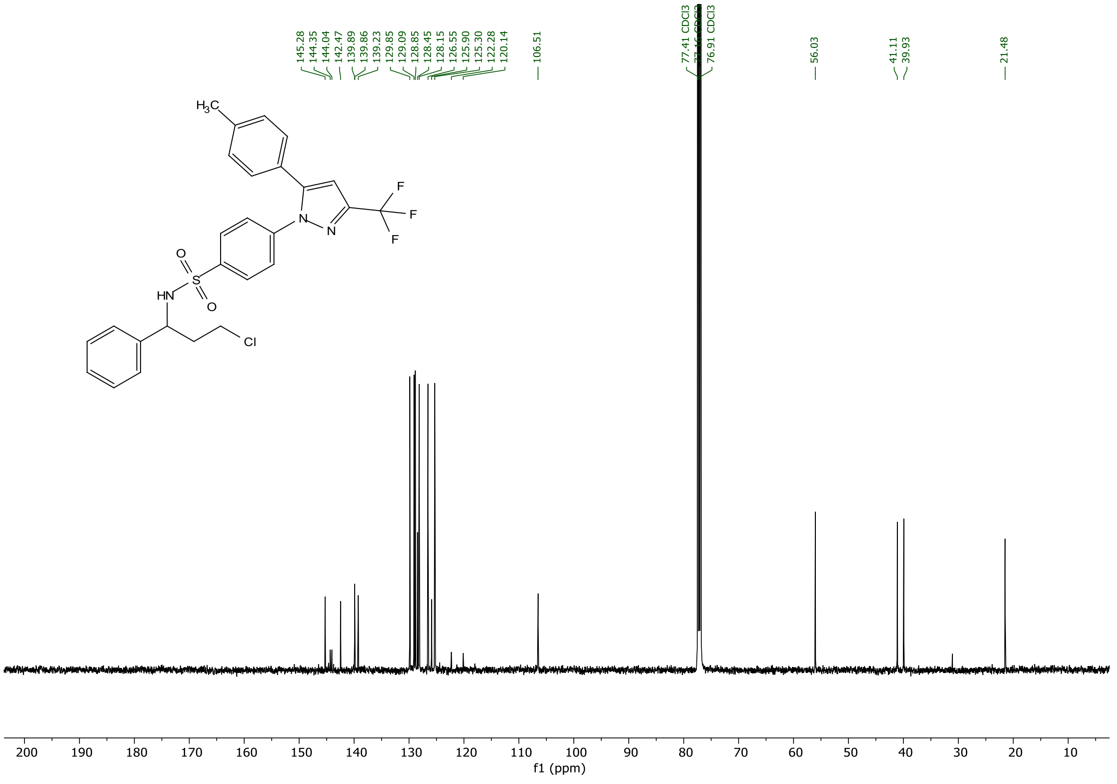

^{19}F NMR (470 MHz, $CDCl_3$)

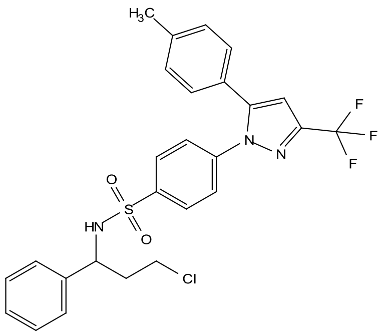

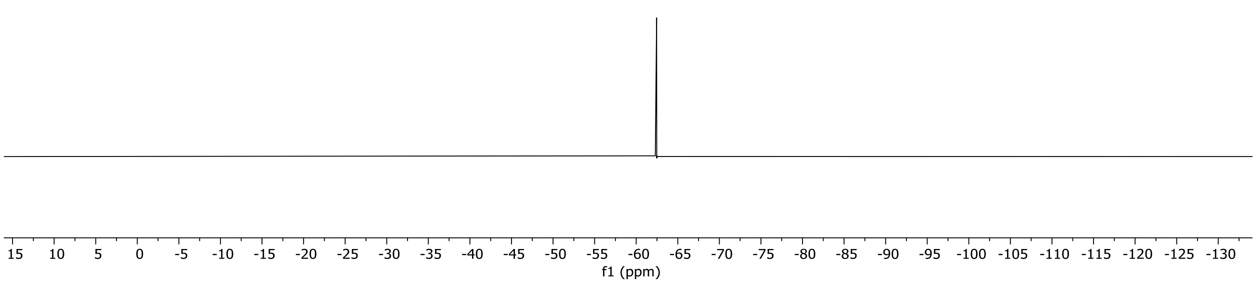

1-Bromo-4-(2-(3-chloro-1-phenylpropoxy)ethyl)benzene (15n). ^{1}H NMR (500 MHz, $CDCl_3$)

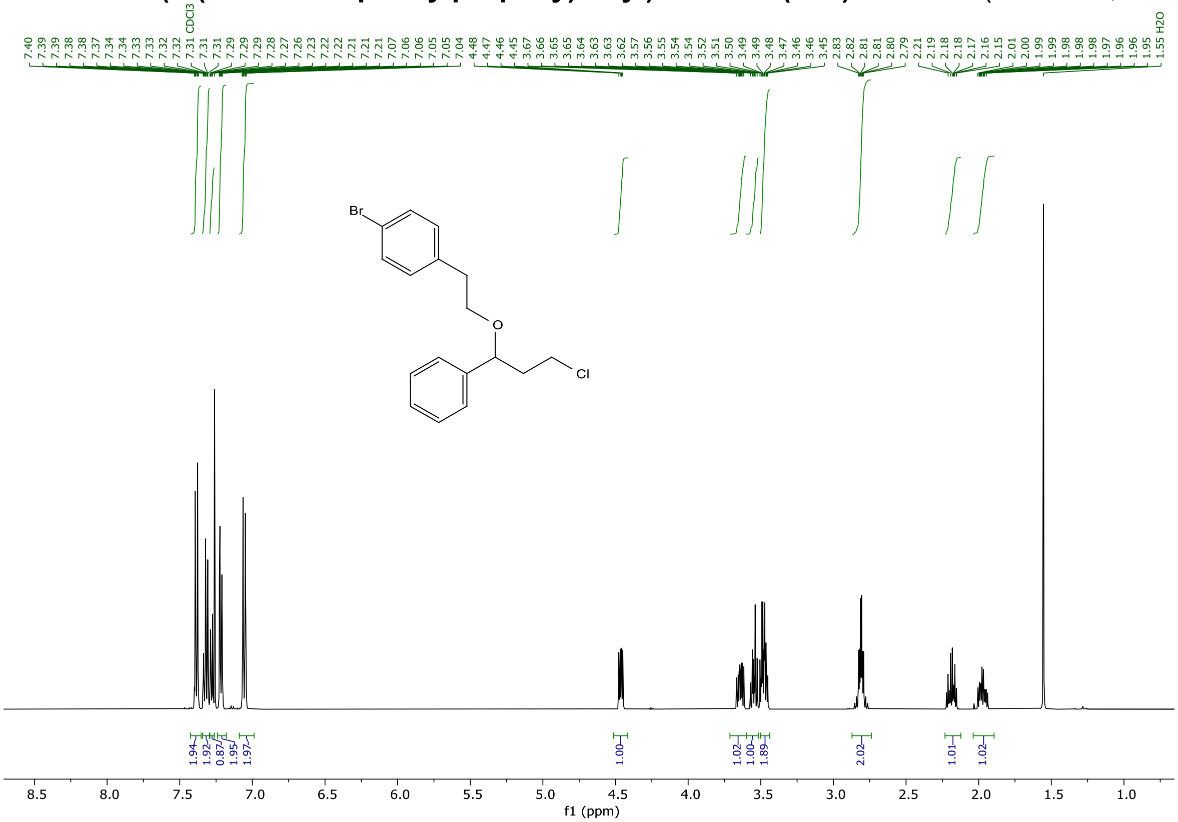

^{13}C NMR (126 MHz, $CDCl_3$)

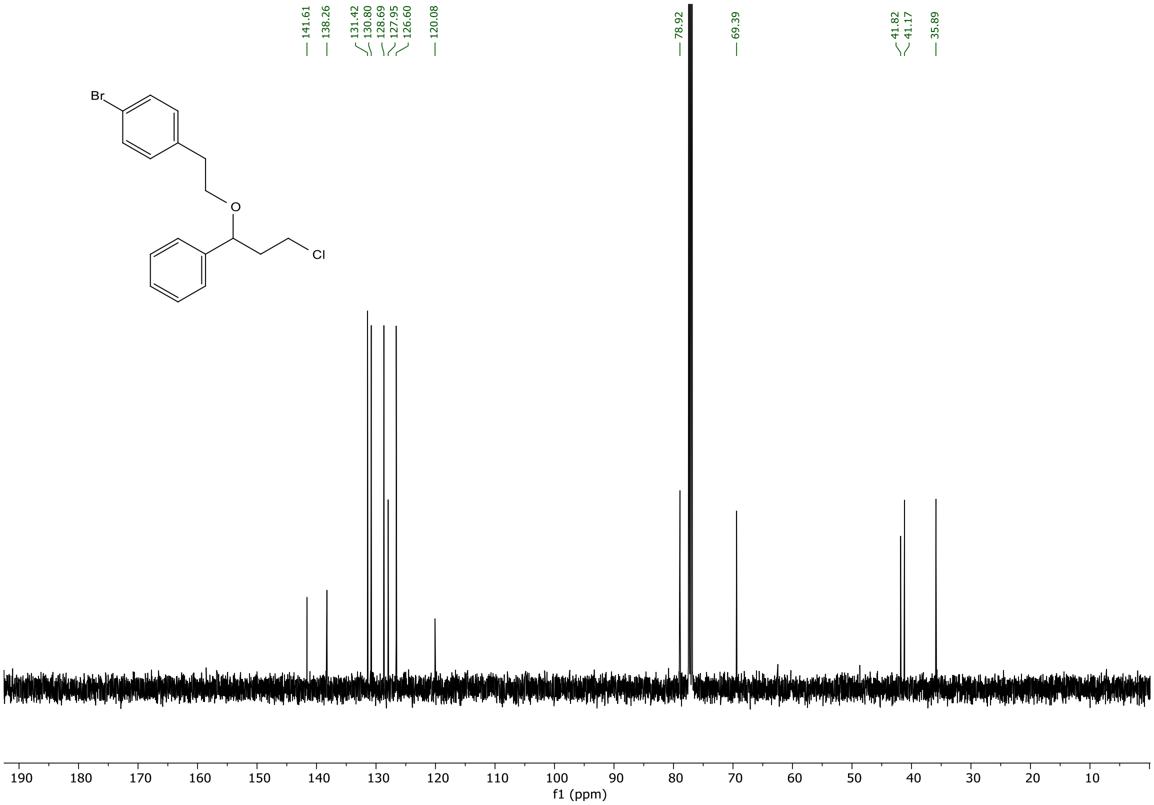

3-(3-Chloro-1-phenylpropoxy)propanenitrile (15o). ^{1}**H NMR** (500 MHz, $CDCl_3$)

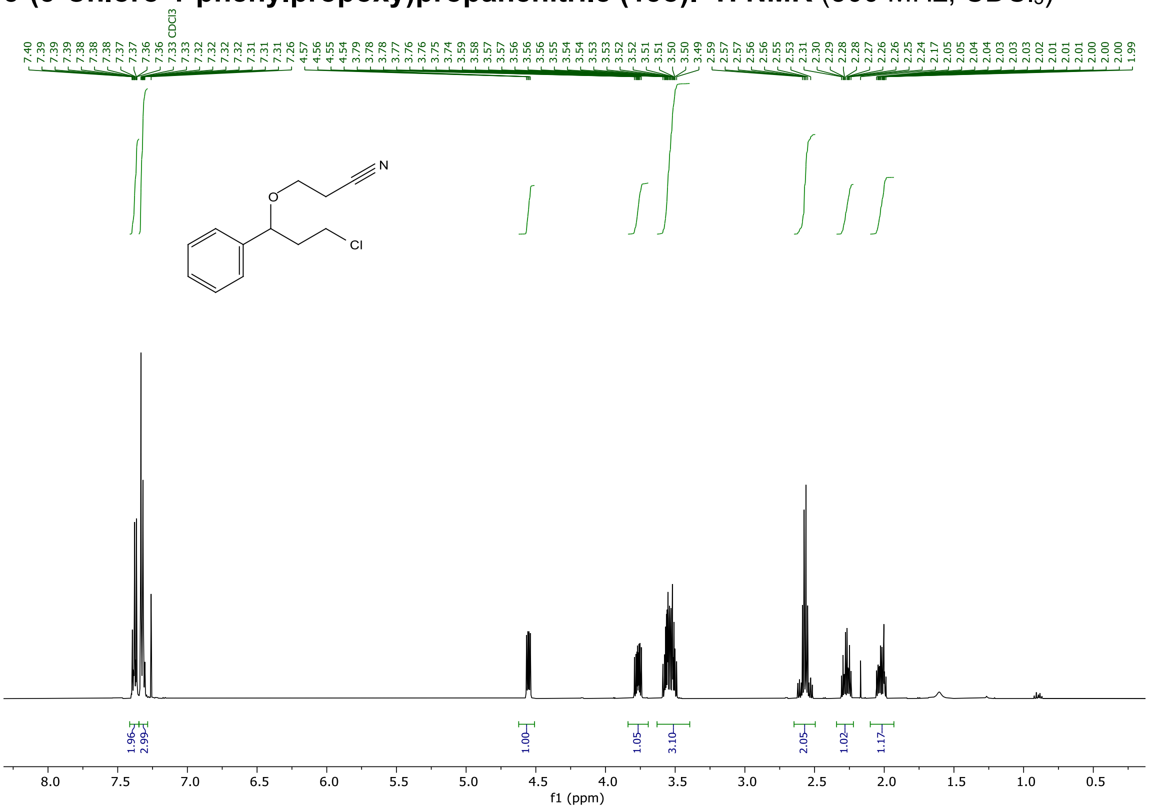

^{13}**C NMR** (126 MHz, $CDCl_3$)

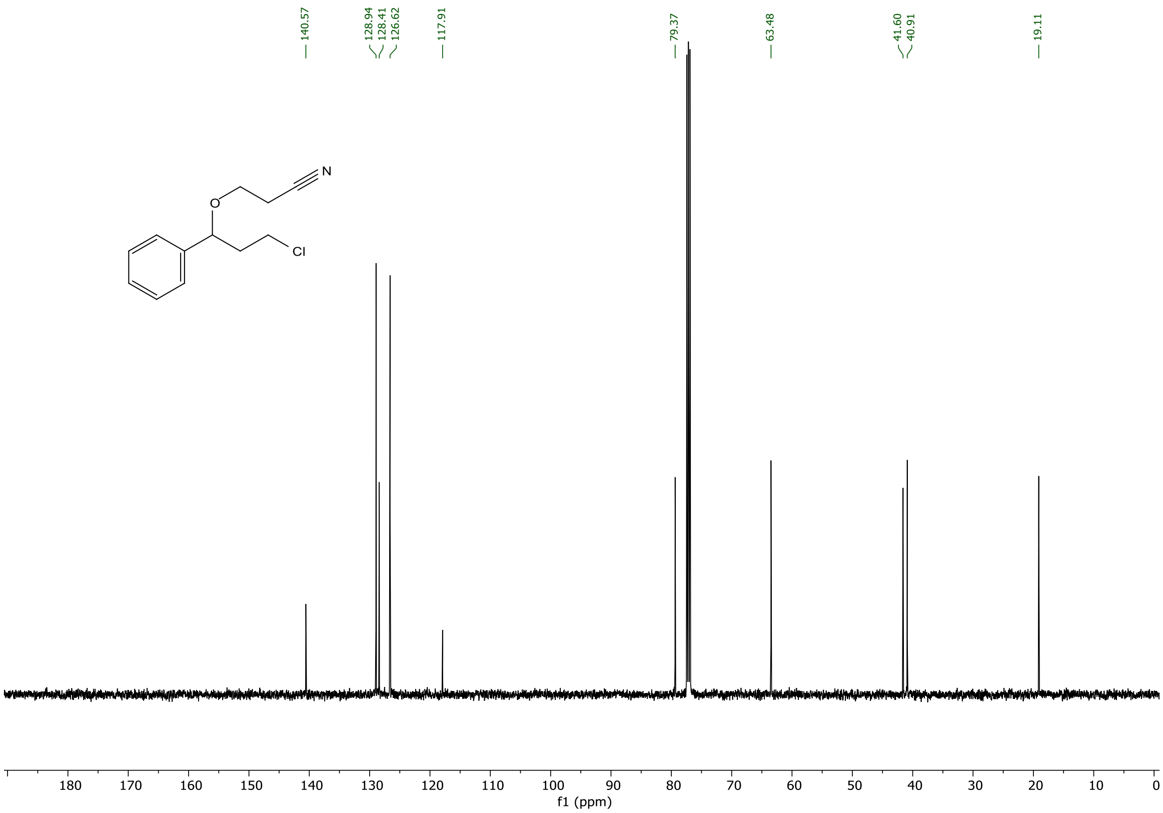

(3-Chloro-1-phenylpropyl)(cyclohexyl)sulfane (15p) **^{1}H NMR** (500 MHz, $CDCl_3$)

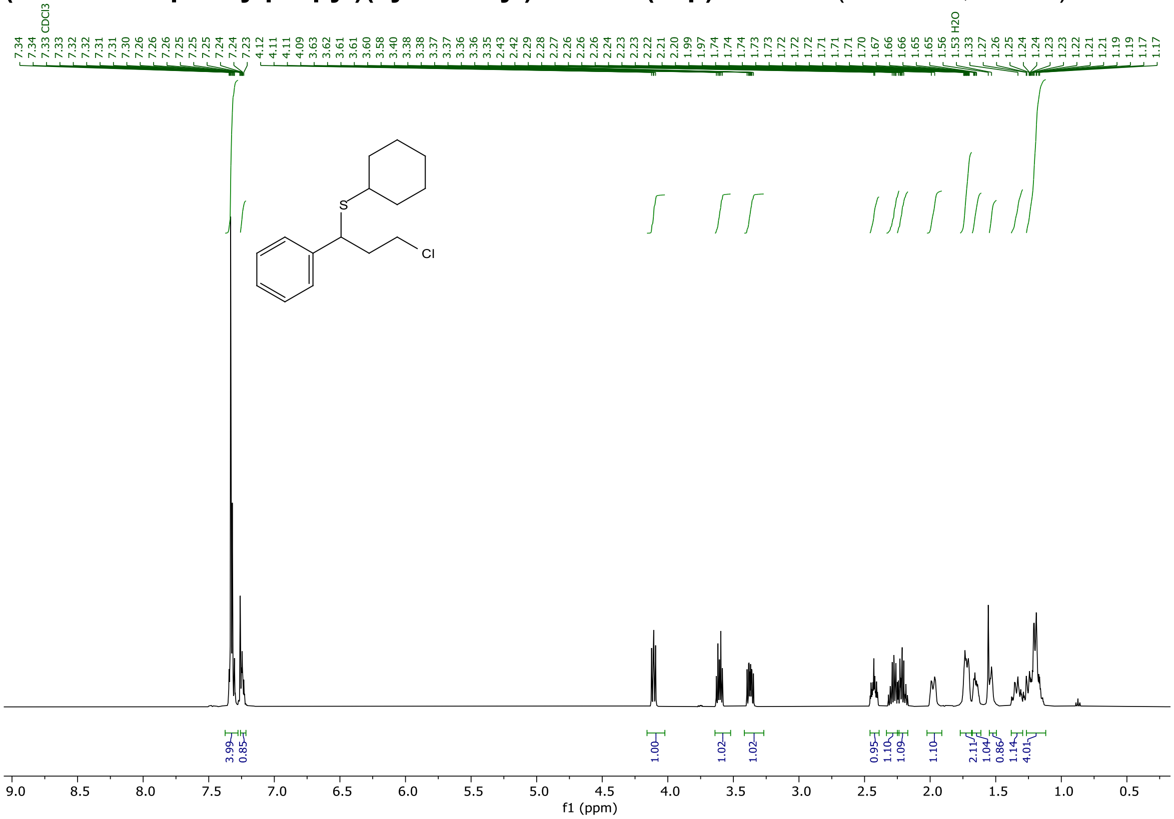

^{13}C NMR (126 MHz, $CDCl_3$)

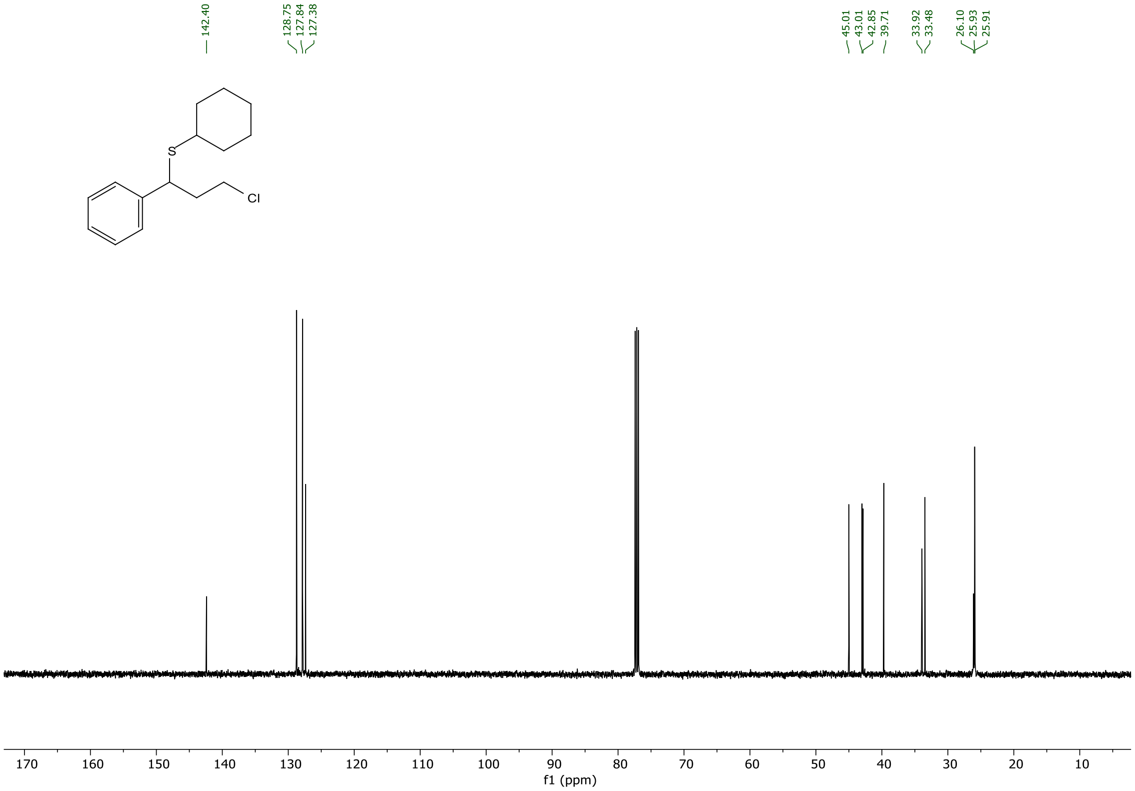

4-(1-Phenylethyl)-1,1'-biphenyl (15q). ^{1}H NMR (500 MHz, $CDCl_3$)

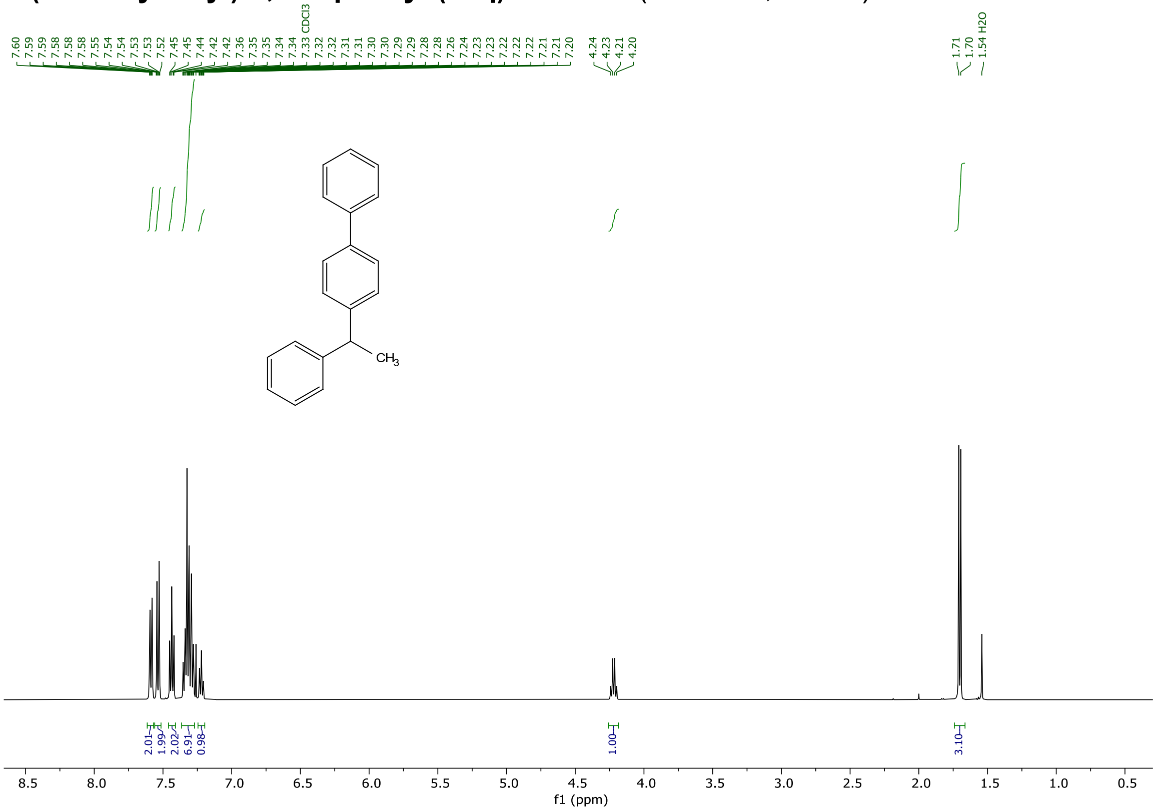

^{13}C NMR (126 MHz, $CDCl_3$)

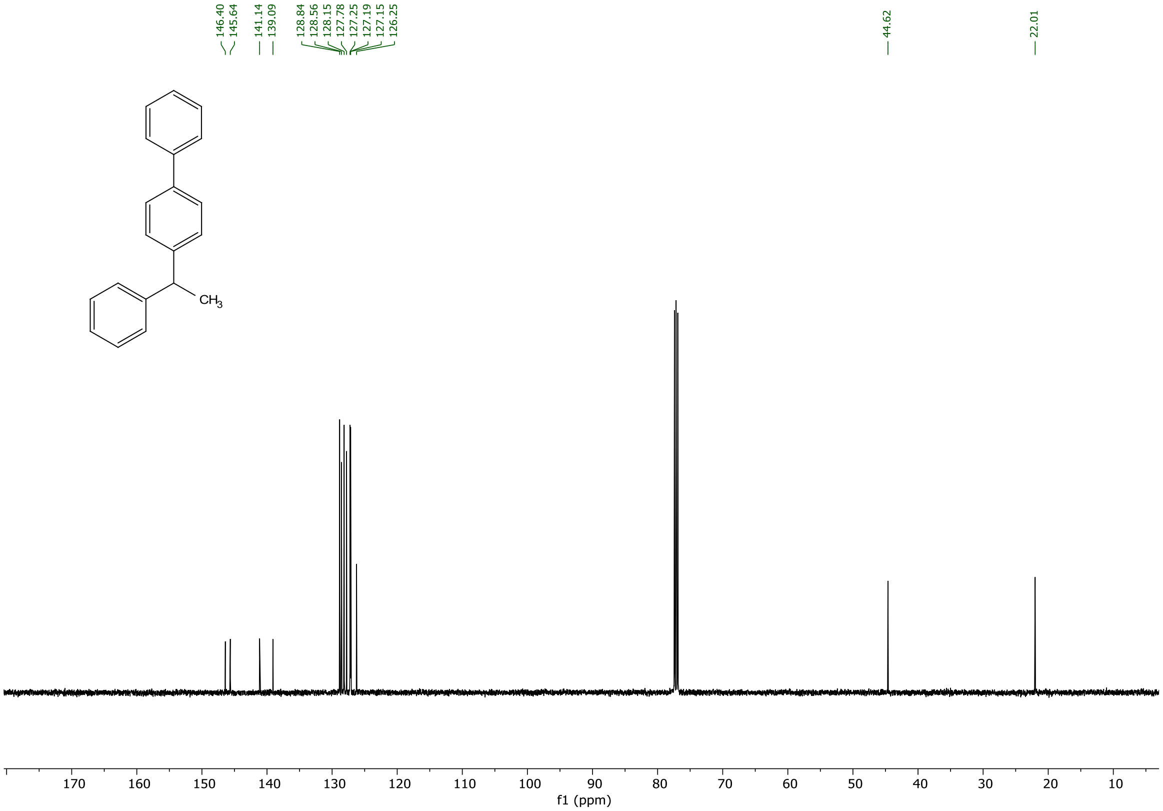

5-(2-Chloro-1-phenylpropyl)benzo[d][1,3]dioxole (15r) ^{1}**H NMR** (500 MHz, $CDCl_3$), mixture of two diastereomers

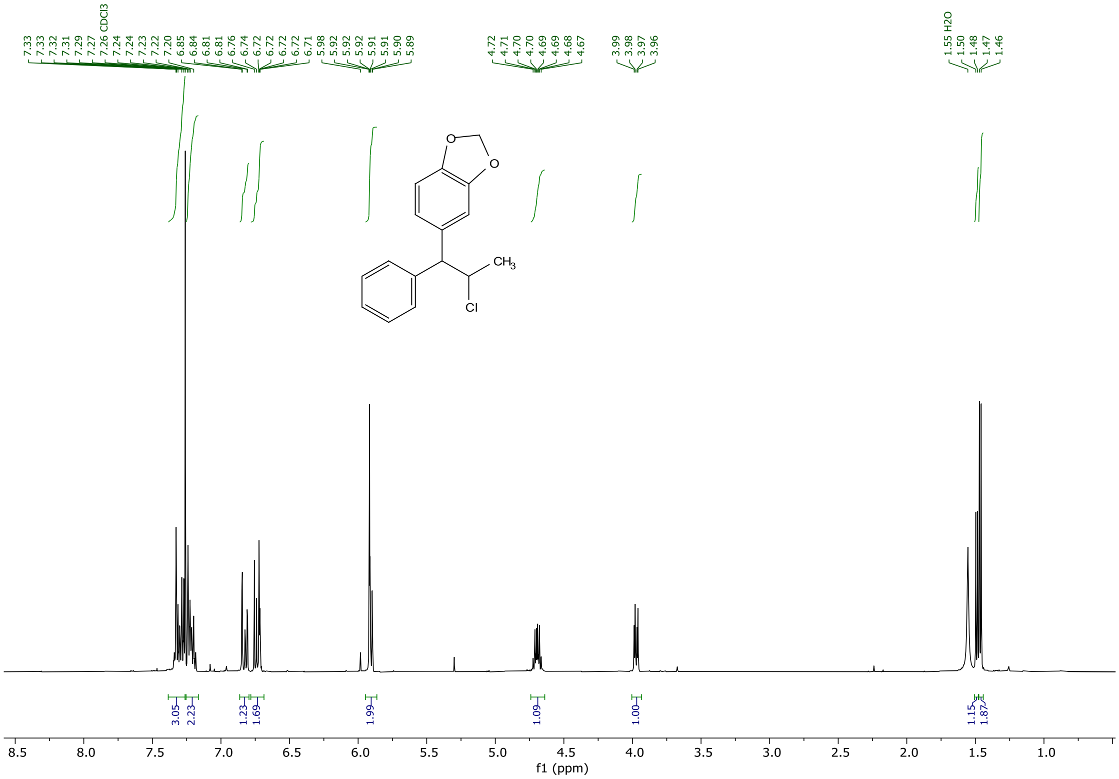

^{13}**C NMR** (126 MHz, $CDCl_3$)

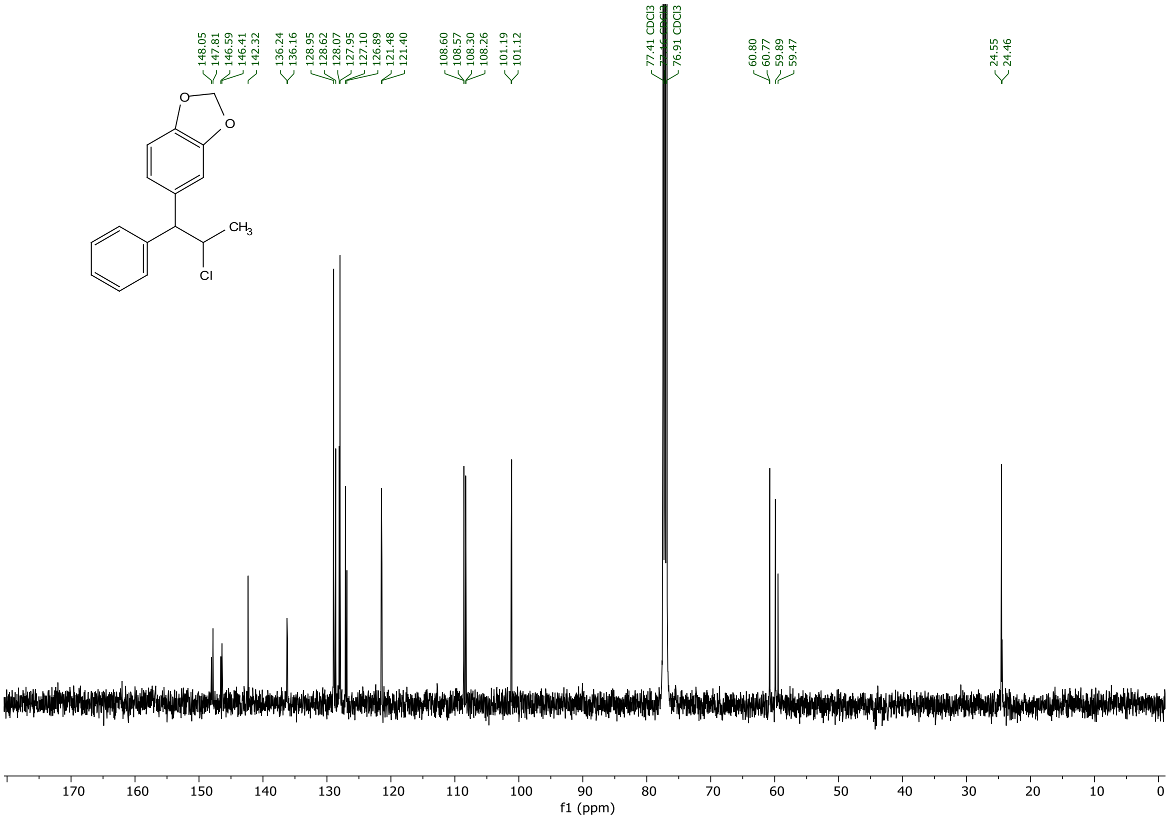

(1-Chloro-4-(3-chloro-1-phenylpropyl)benzene, (16a) ^{1}**H NMR** (400 MHz, $CDCl_3$)

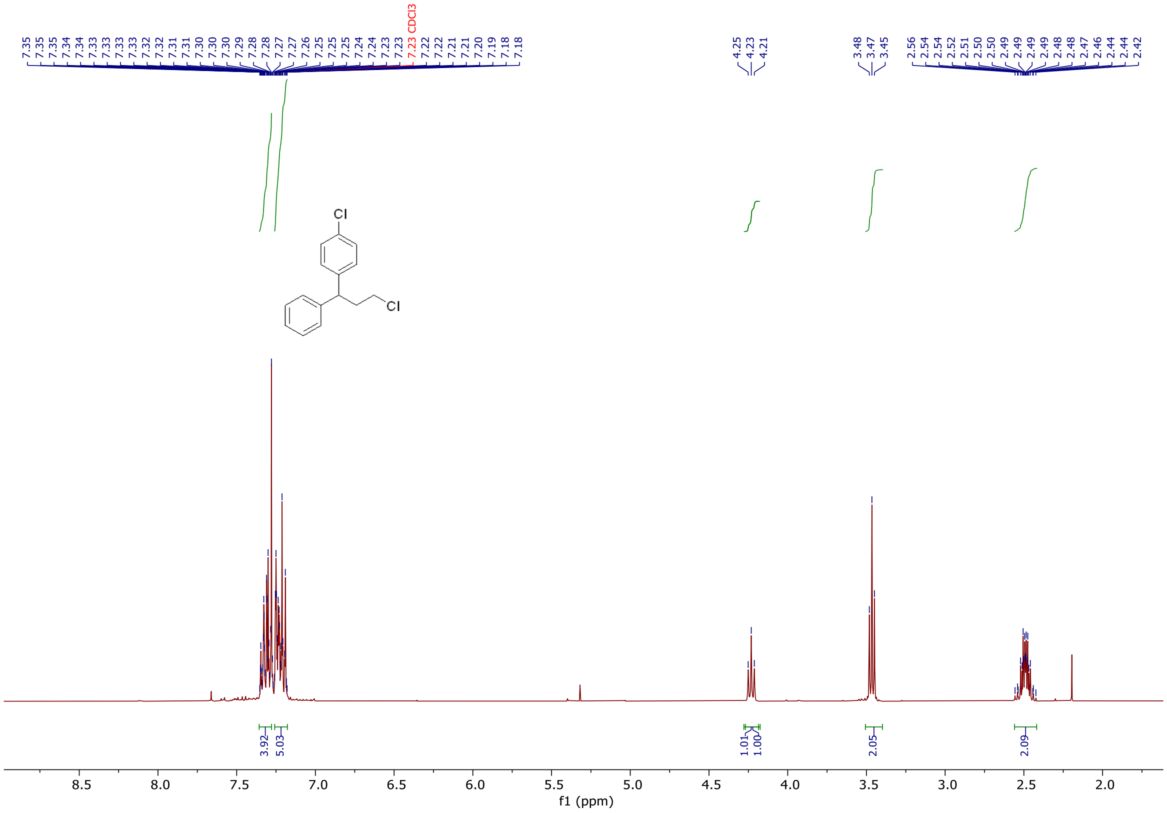

^{13}**C NMR** (101 MHz, $CDCl_3$)

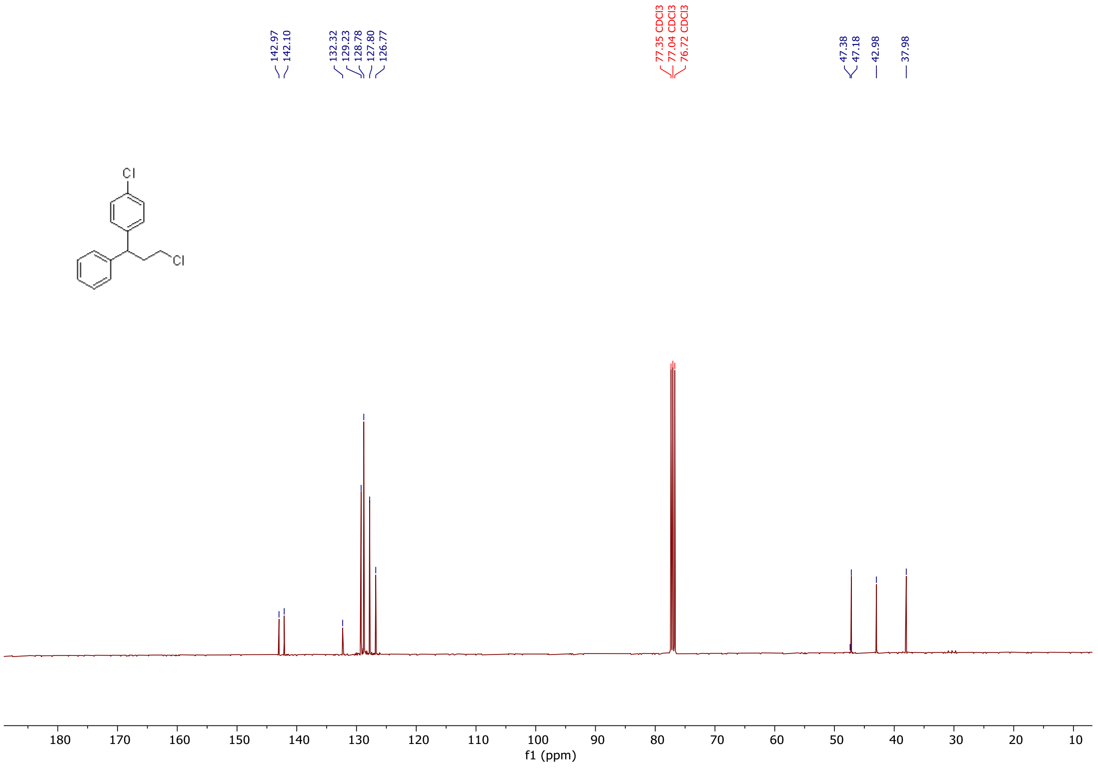

(2-Chloro-4-(3-chloro-1-phenylpropyl)-1-methoxybenzene (16b) ^{1}H NMR (400 MHz, $CDCl_3$)

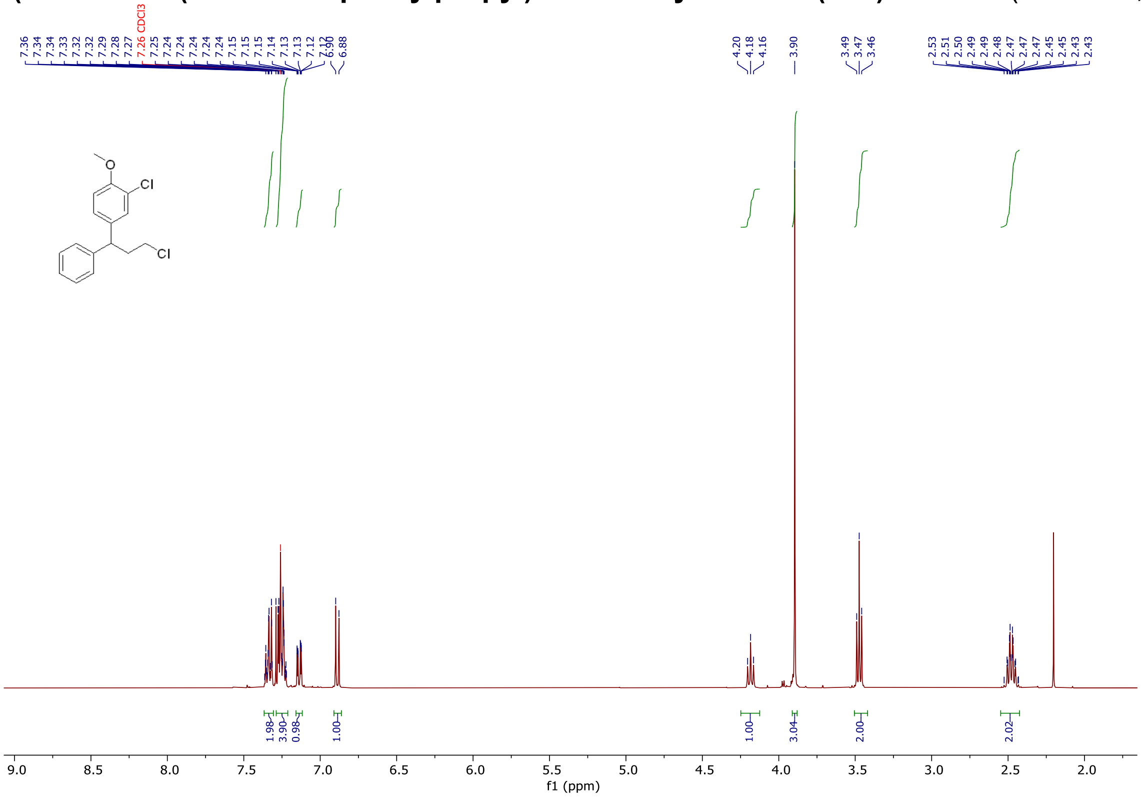

^{13}C NMR (101 MHz, $CDCl_3$)

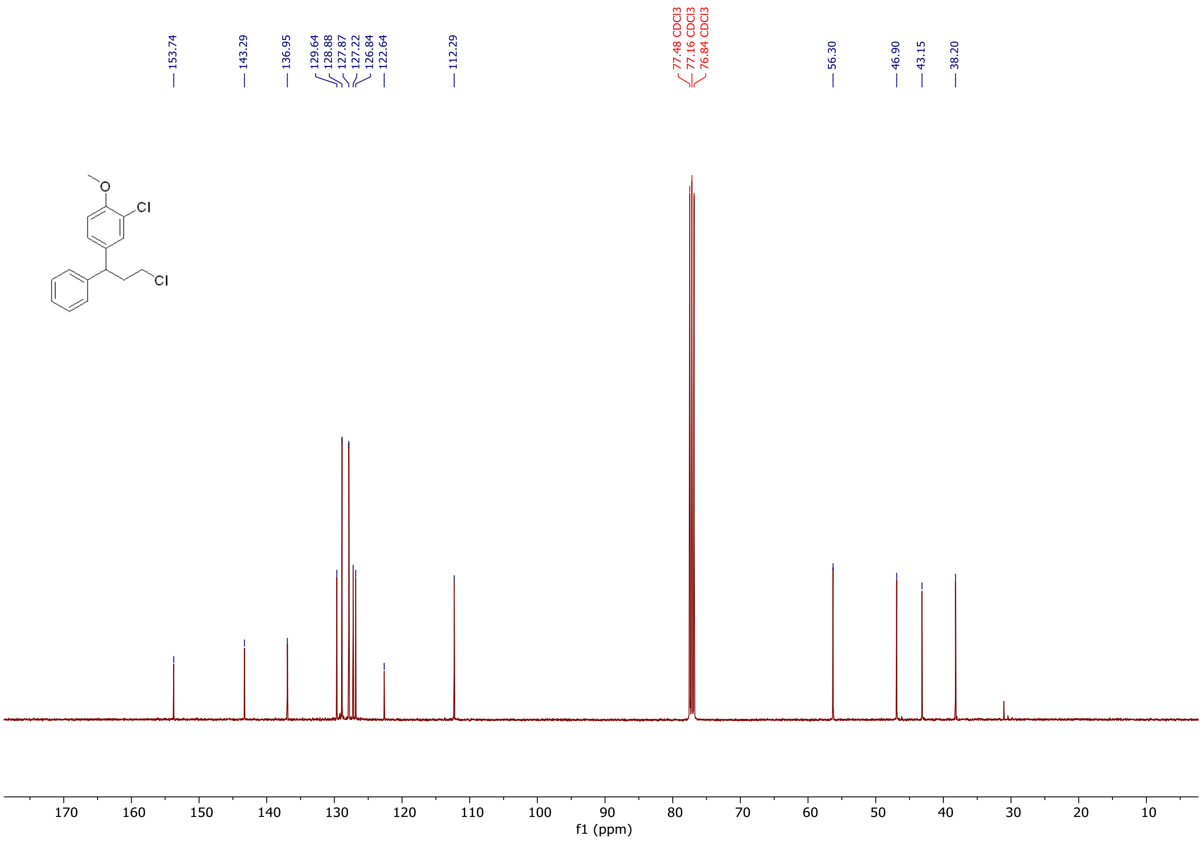

(1-(3-Chloro-1-phenylpropyl)-4-(trifluoromethyl)benzene, (16c) **^{1}H NMR** (400 MHz, $CDCl_3$)

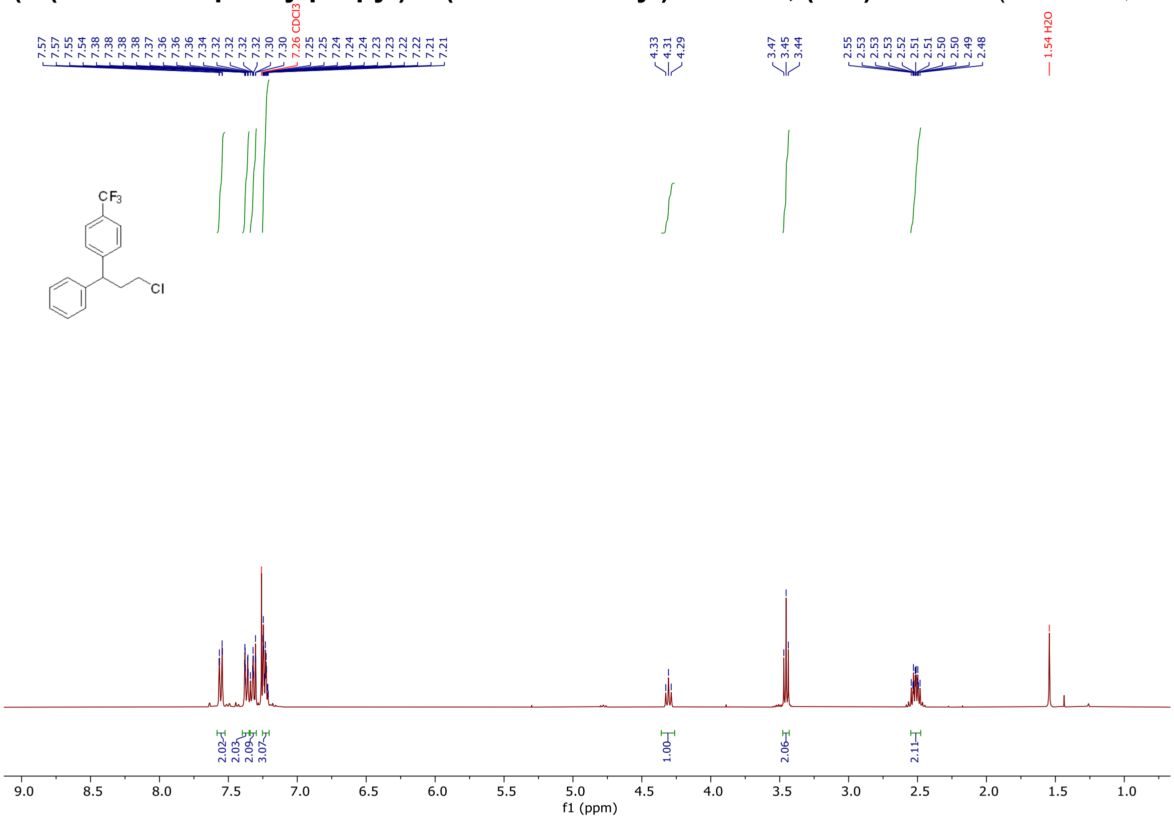

^{13}C NMR (101 MHz, $CDCl_3$)

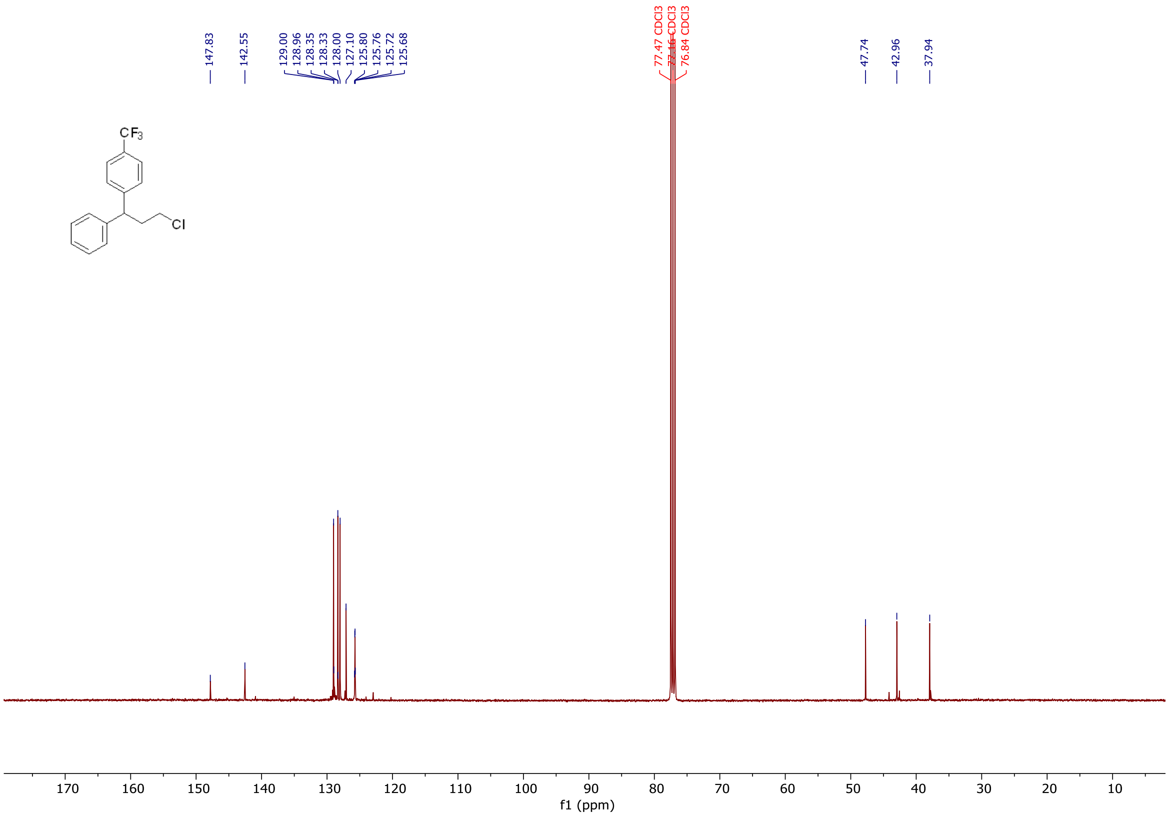

^{19}F NMR (376 MHz, $CDCl_3$)

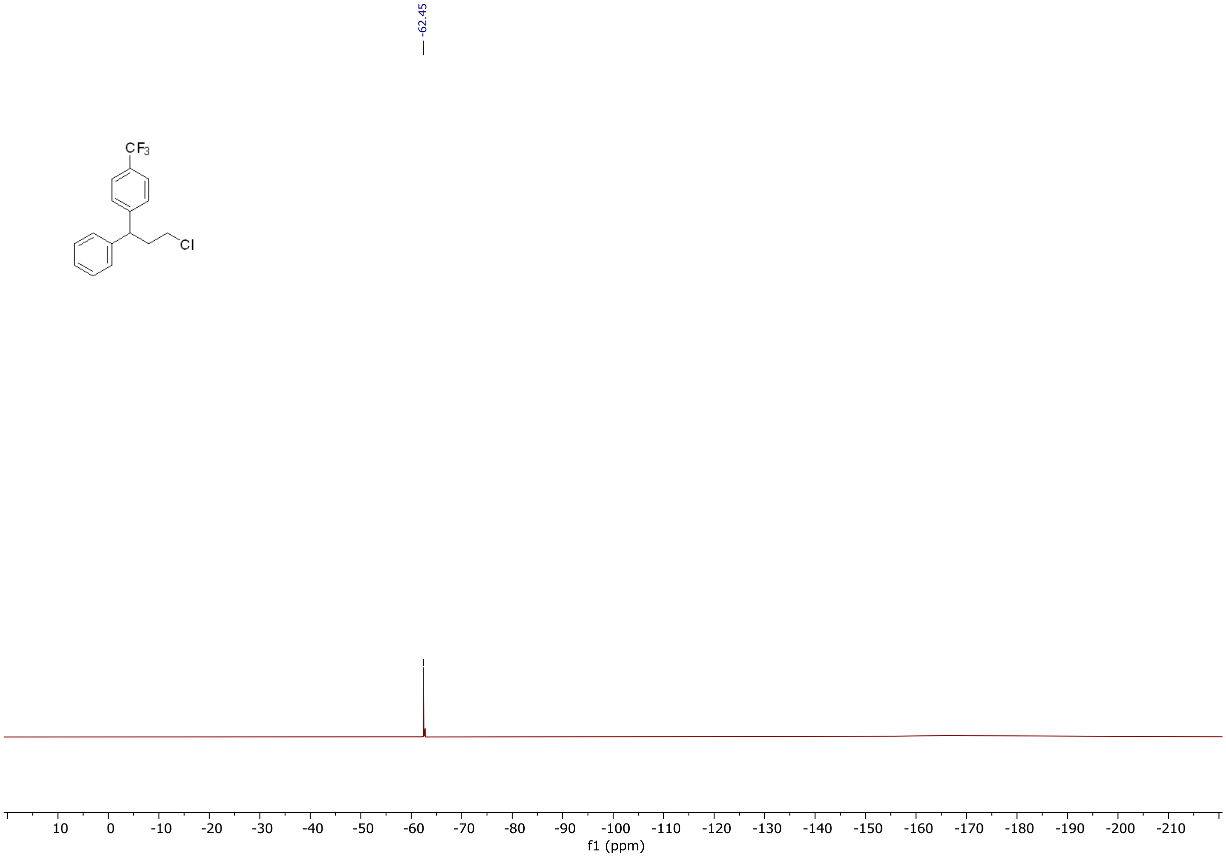

(1-Chloro-4-(3-chloro-1-phenylbutyl)benzene, (16d) ^{1}**H NMR** (400 MHz, $CDCl_3$), mixture of two diastereomers

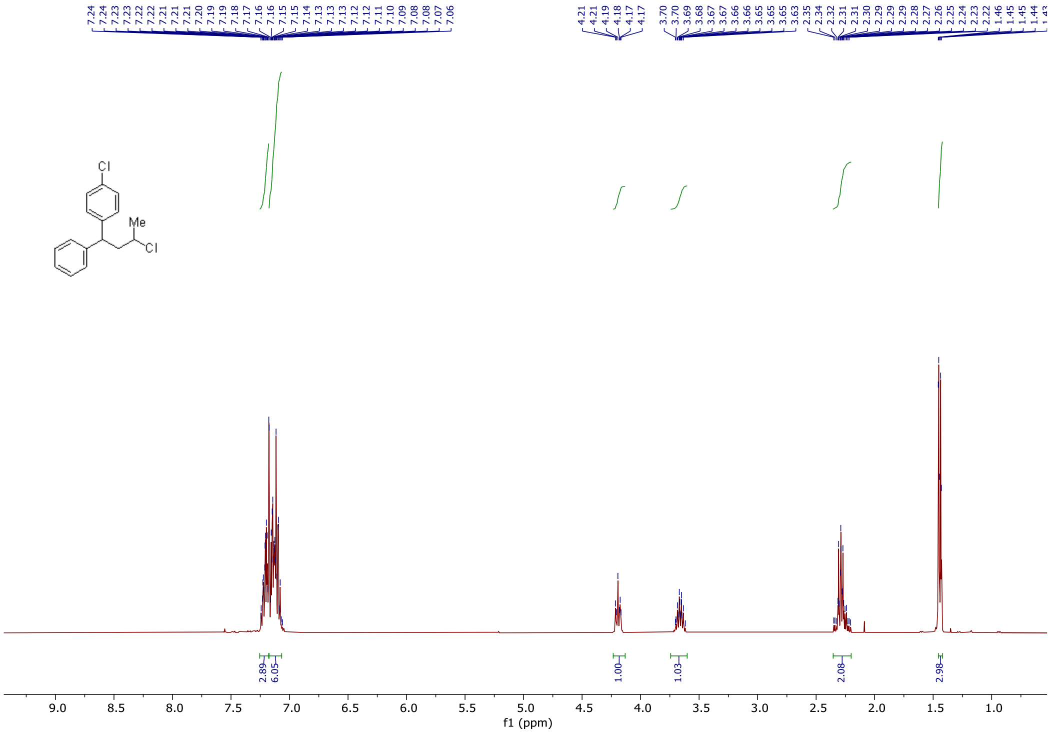

^{13}**C NMR** (101 MHz, $CDCl_3$)

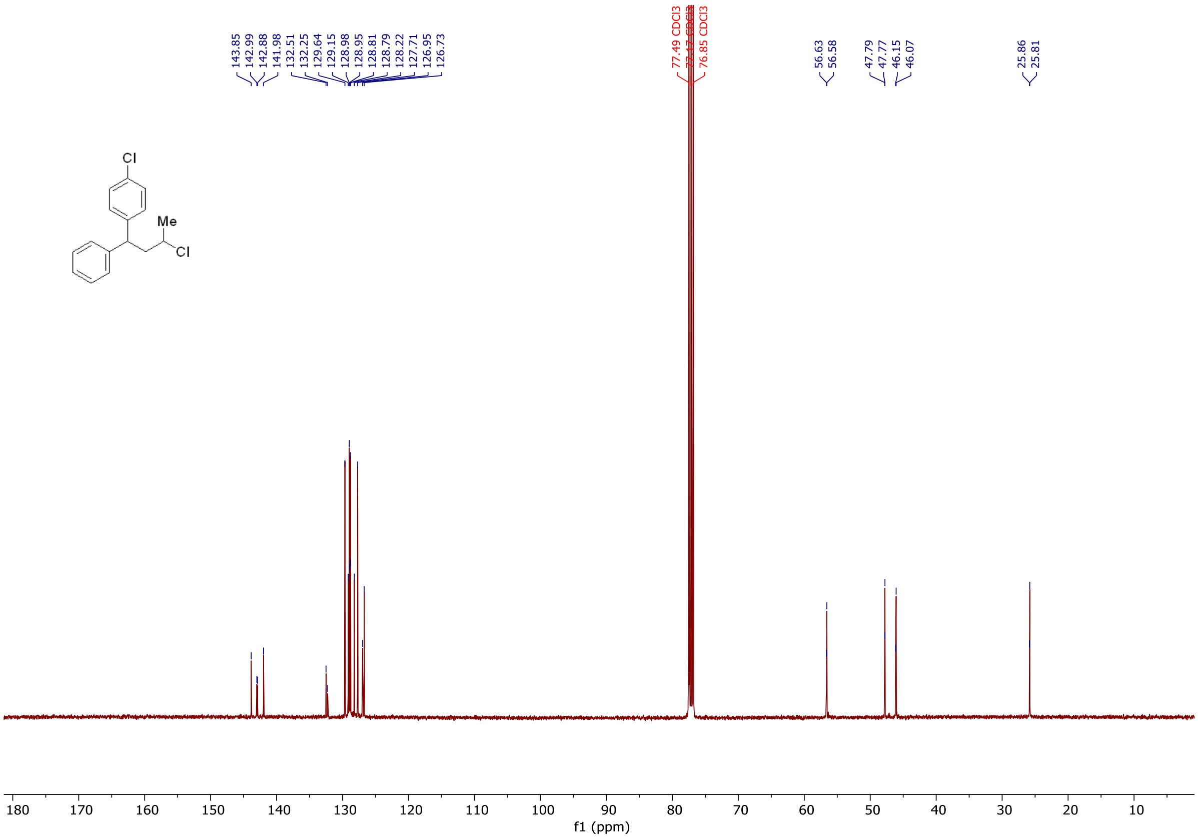

4,4'-(1-Phenylpropane-1,3-diyldisulfonyl)bis(methylbenzene) (17a). ^{1}H NMR (500 MHz, $CDCl_3$)

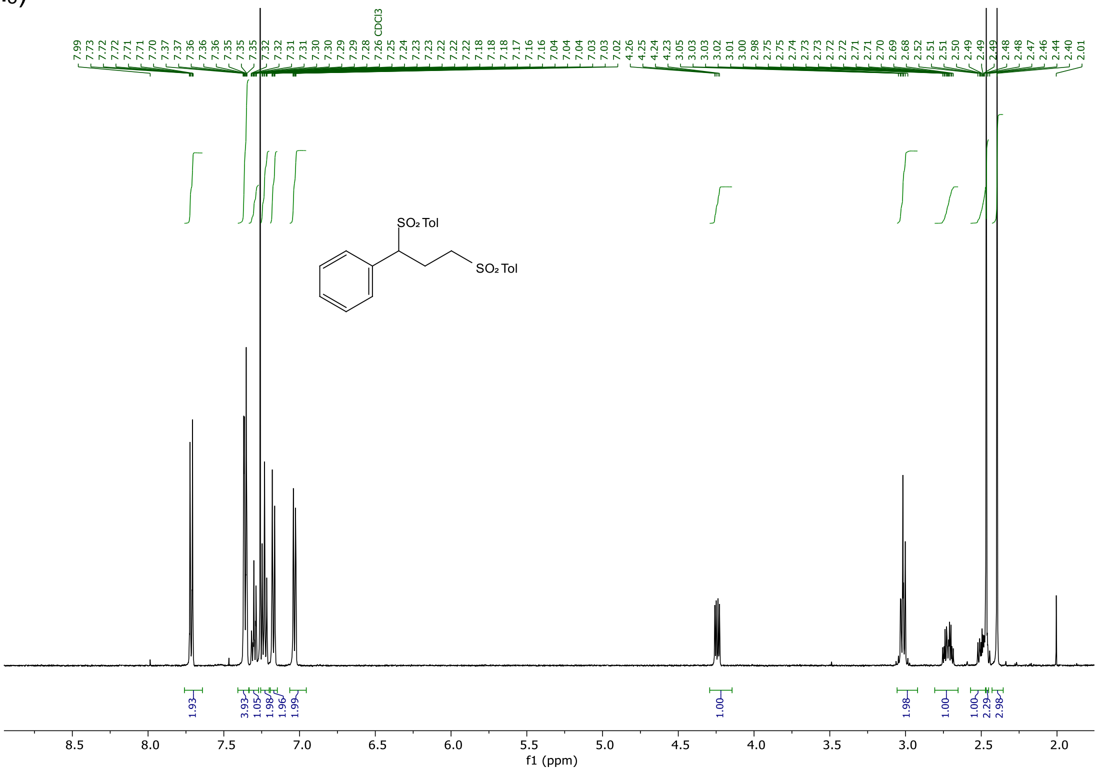

^{13}C NMR (126 MHz, $CDCl_3$)

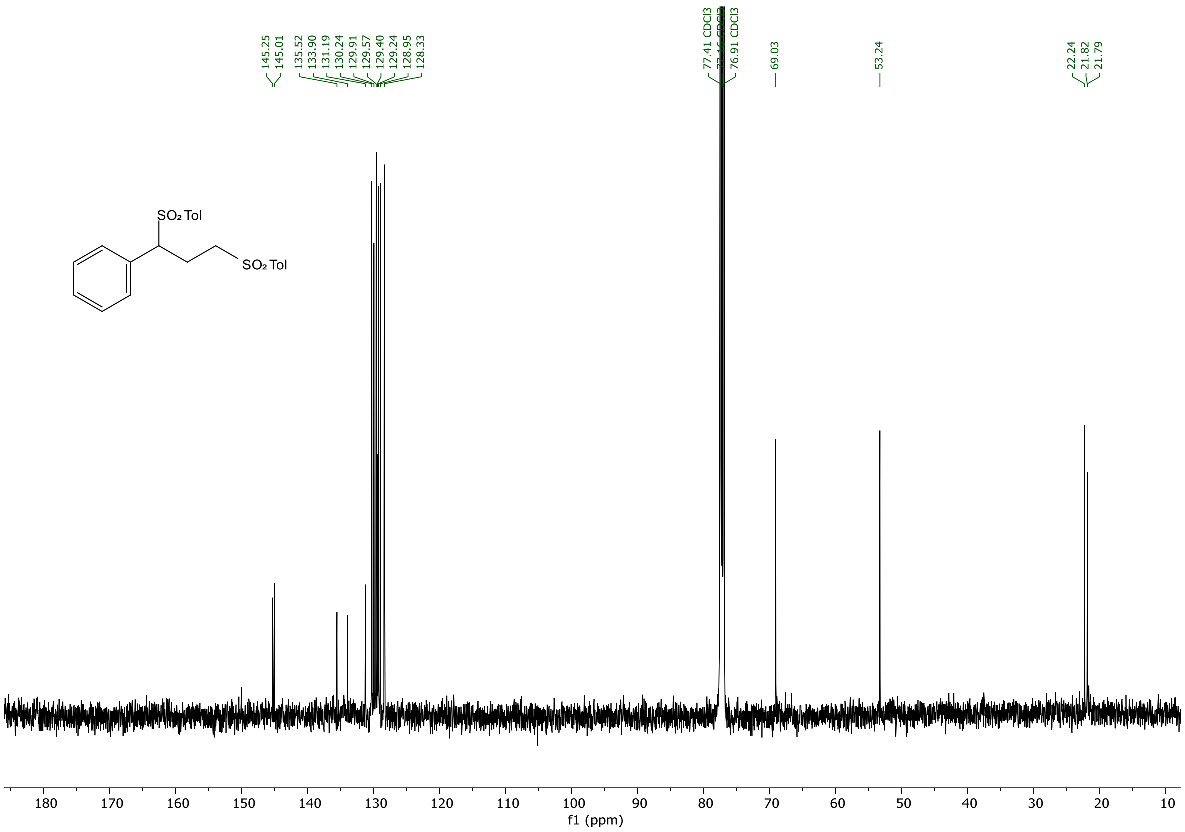

(1,3-Diazidopropyl)benzene (17b) ^{1}**H NMR** (500 MHz, $CDCl_3$)

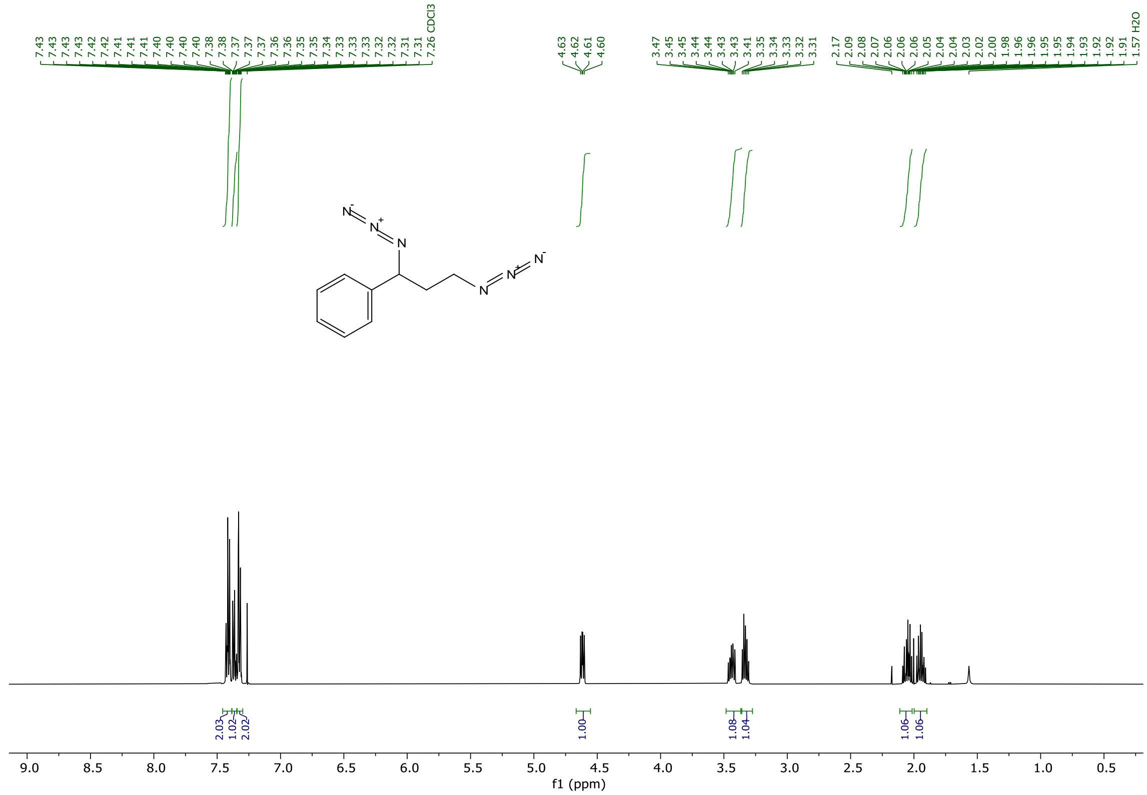

^{3}**C NMR** (126 MHz, $CDCl_3$)

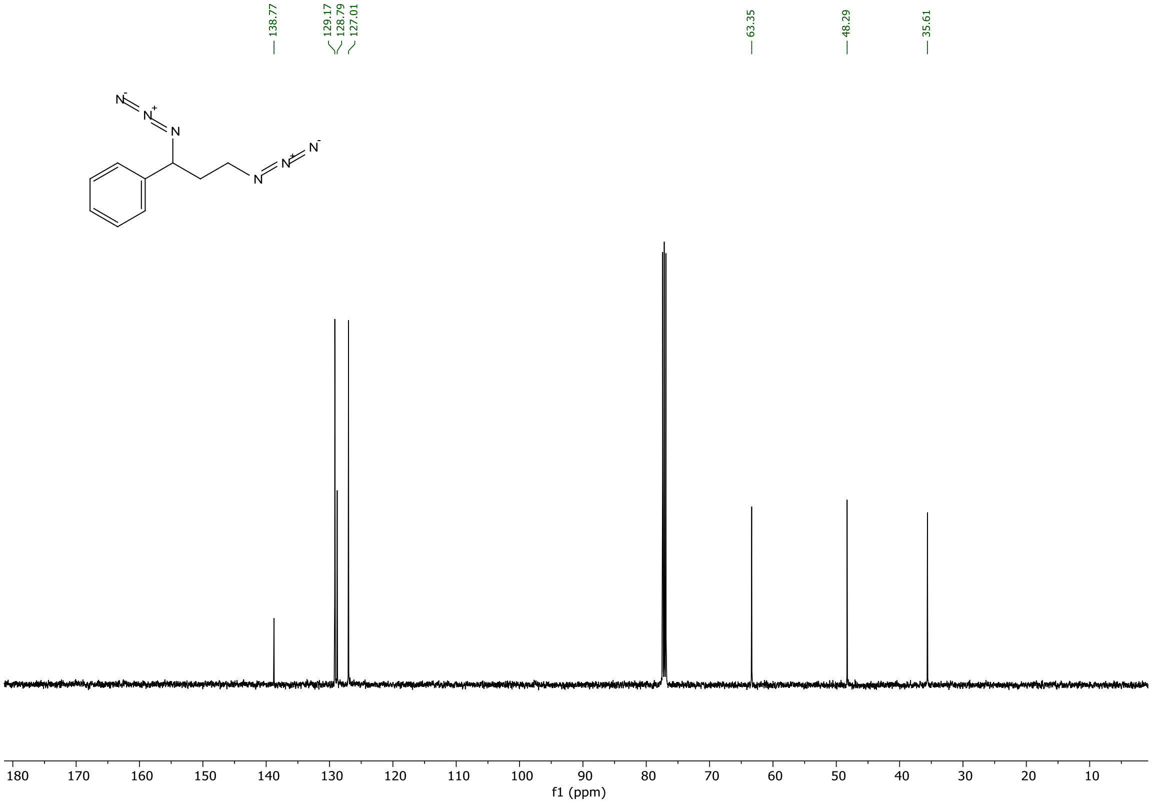

1-(1-Azidoethyl)-4-bromobenzene (18a). ^{1}H NMR (500 MHz, $CDCl_3$)

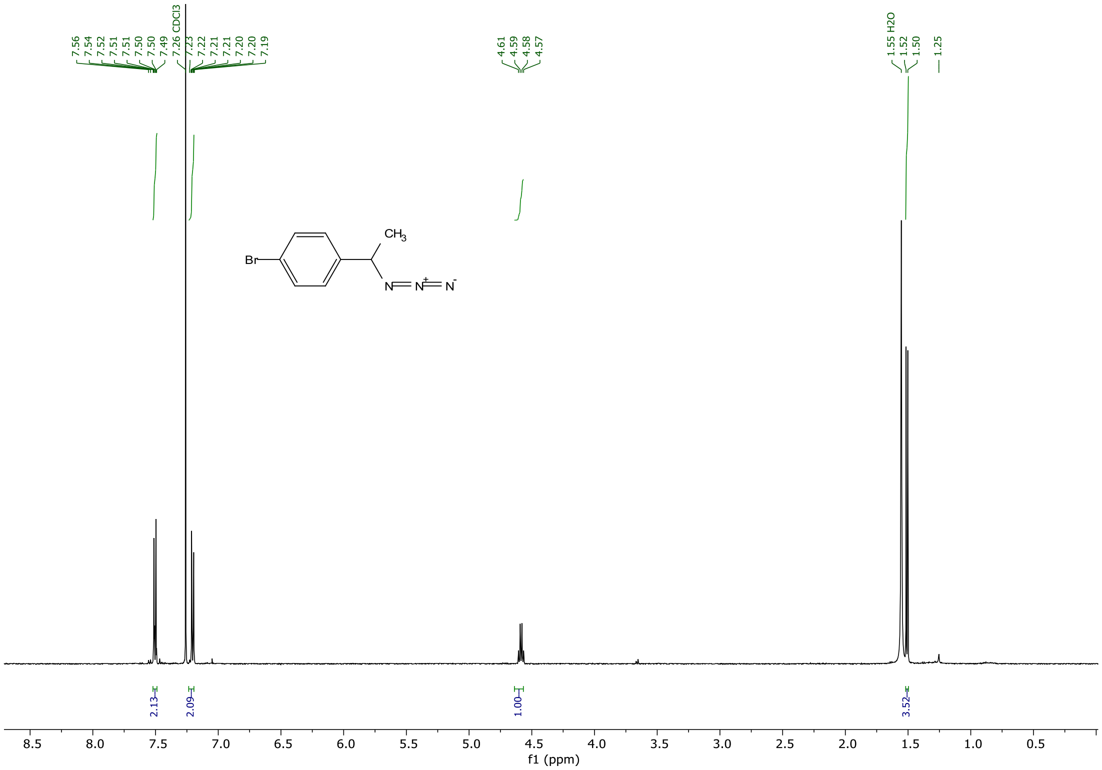

^{13}C NMR (126 MHz, $CDCl_3$)

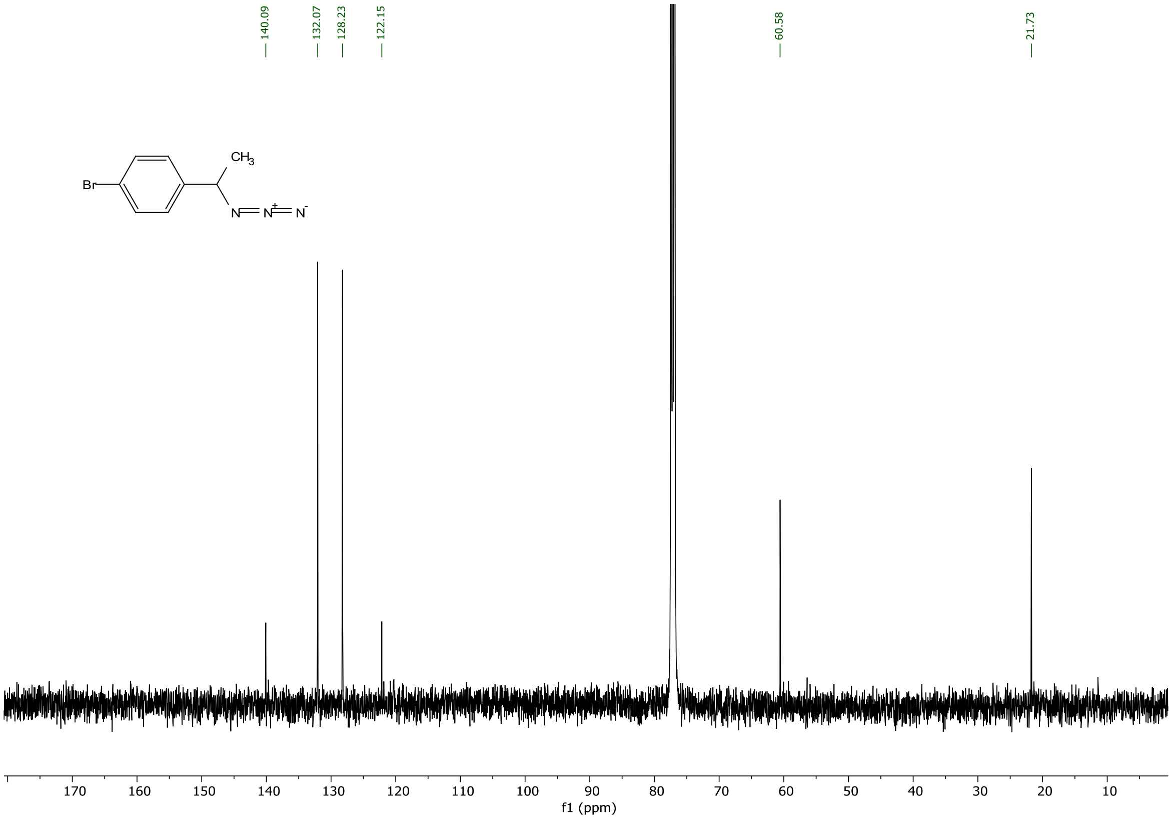

N-(1-Phenylethyl)morpholine (18b).). **^{1}H NMR** (400 MHz, $CDCl_3$)

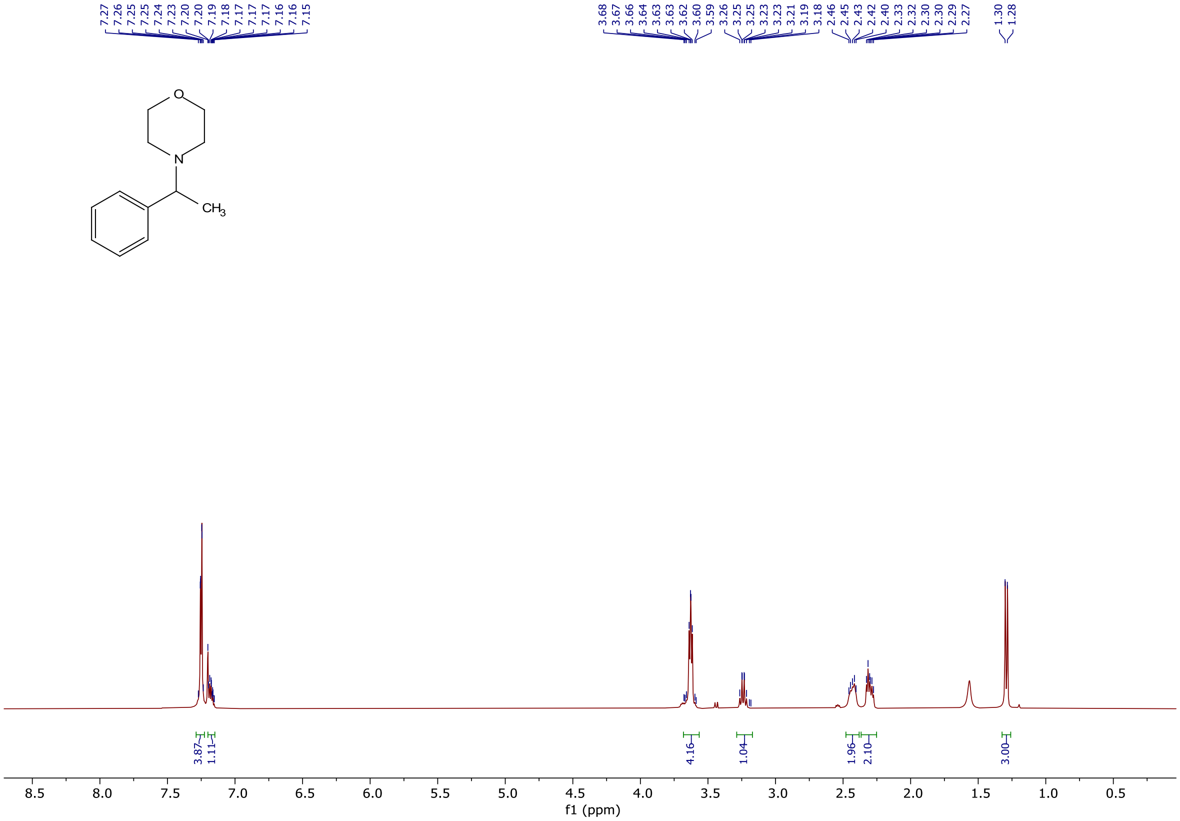

^{13}C NMR (101 MHz, $CDCl_3$)

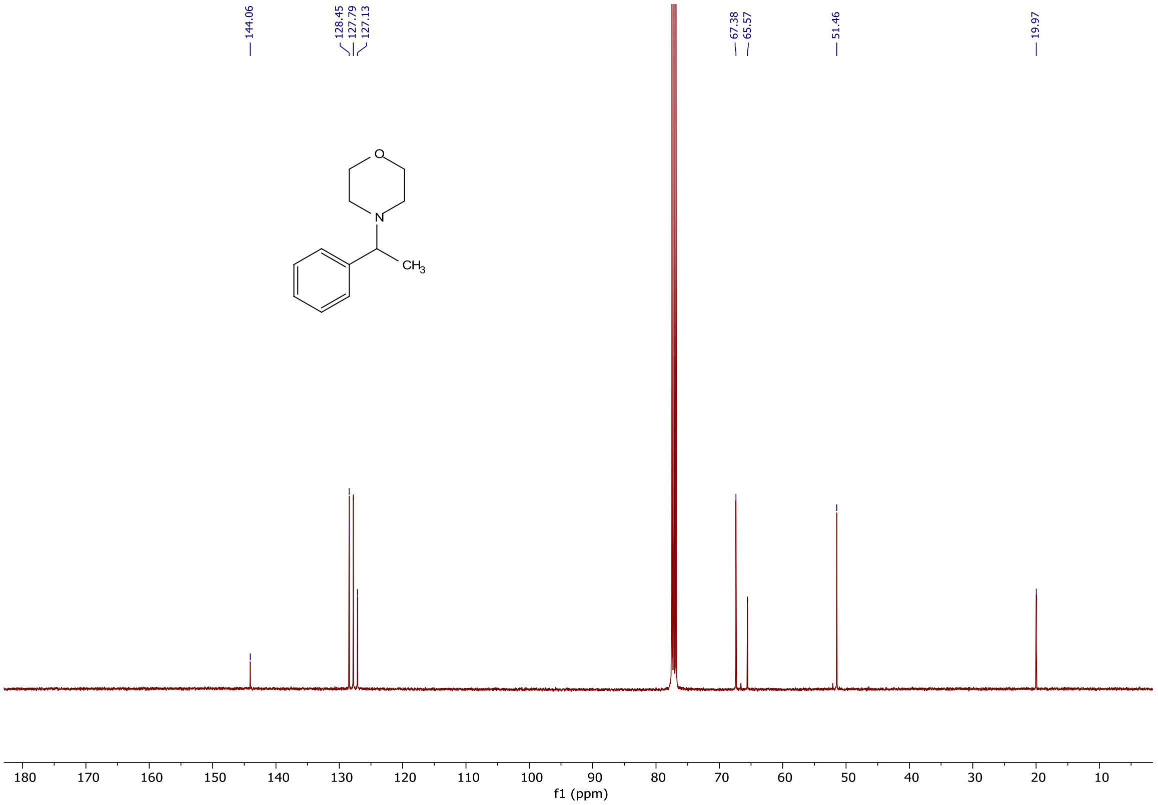

4-(1-([1,1'-Biphenyl]-4-yl)ethyl)morpholine (18c). ^{1}**H NMR** (400 MHz, $CDCl_3$)

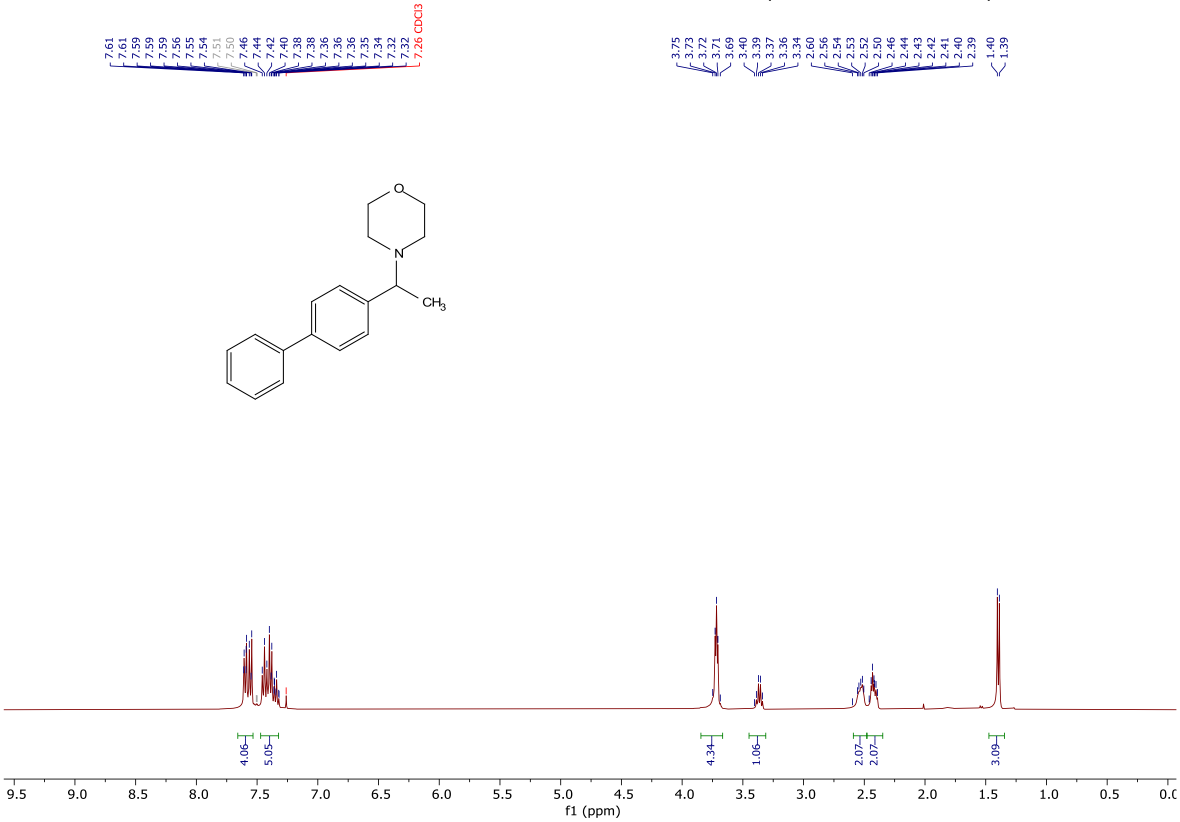

^{13}**C NMR** 101 MHz, $CDCl_3$)

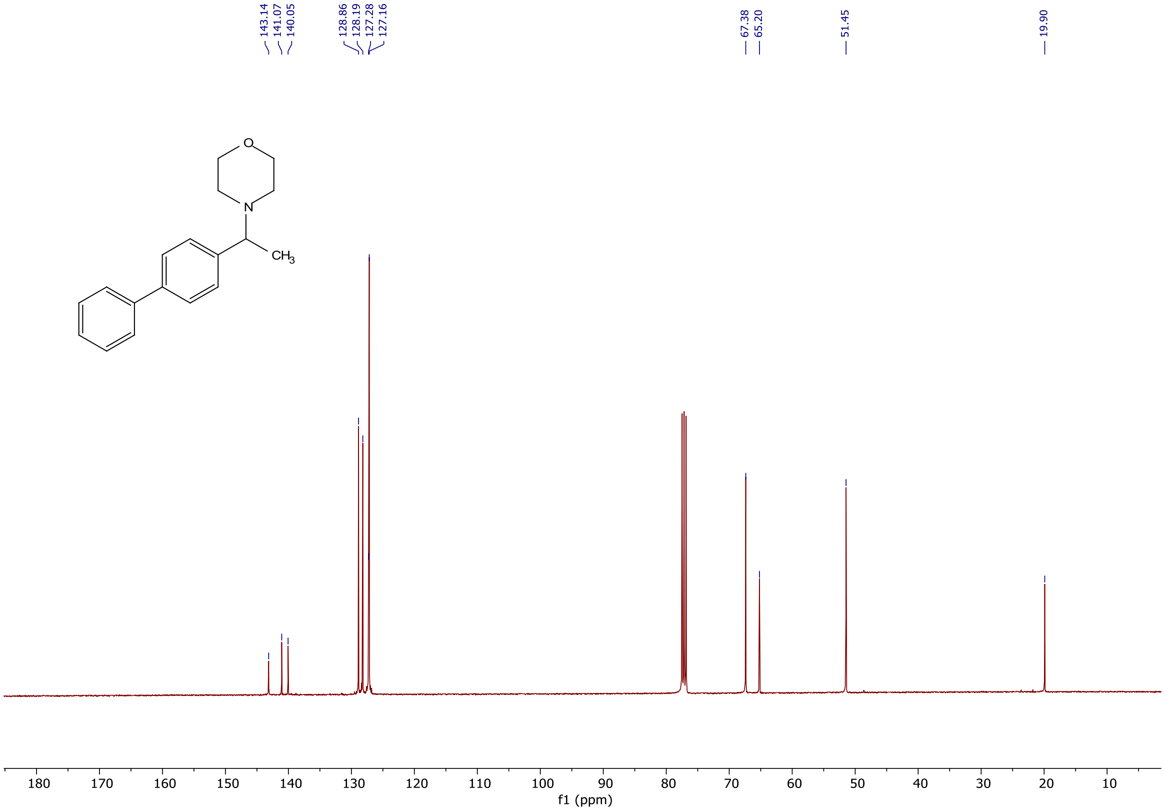

Supplement: Supplementary file 1 [file au5c01226_si_001.pdf]
